# A novel class III endogenous retrovirus with a class I envelope gene in African frogs with an intact genome and developmentally regulated transcripts in *Xenopus tropicalis*

**DOI:** 10.1186/s12977-021-00564-2

**Published:** 2021-07-14

**Authors:** Venkat R. K. Yedavalli, Akash Patil, Janay Parrish, Christine A. Kozak

**Affiliations:** 1 Laboratory of Molecular Microbiology, National Institute of Allergy and Infectious Diseases, Bethesda, MD 20892 USA; 2 Department of Biomedical Engineering, John Hopkins University, Baltimore, MD 21205 USA; 3 Internal Medicine, Northwell Health, Lenox Hill Hospital, New York, NY 10075 USA

**Keywords:** Endogenous retroviruses, Amphibian retroviruses, ERV-L, *Xenopus* retrovirus, African frog retroviruses

## Abstract

**Background:**

Retroviruses exist as exogenous infectious agents and as endogenous retroviruses (ERVs) integrated into host chromosomes. Such endogenous retroviruses (ERVs) are grouped into three classes roughly corresponding to the seven genera of infectious retroviruses: class I (gamma-, epsilonretroviruses), class II (alpha-, beta-, delta-, lentiretroviruses) and class III (spumaretroviruses). Some ERVs have counterparts among the known infectious retroviruses, while others represent paleovirological relics of extinct or undiscovered retroviruses.

**Results:**

Here we identify an intact ERV in the Anuran amphibian, *Xenopus tropicalis.* XtERV-S has open reading frames (ORFs) for *gag*, *pol* (polymerase) and *env* (envelope) genes, with a small additional ORF in *pol* and a serine tRNA primer binding site. It has unusual features and domain relationships to known retroviruses. Analyses based on phylogeny and functional motifs establish that XtERV-S *gag* and *pol* genes are related to the ancient *env*-less class III ERV-L family but the surface subunit of *env* is unrelated to known retroviruses while its transmembrane subunit is class I-like. LTR constructs show transcriptional activity, and XtERV-S transcripts are detected in embryos after the maternal to zygotic mid-blastula transition and before the late tailbud stage. Tagged Gag protein shows typical subcellular localization. The presence of ORFs in all three protein-coding regions along with identical 5’ and 3’ LTRs (long terminal repeats) indicate this is a very recent germline acquisition. There are older, full-length, nonorthologous, defective copies in *Xenopus laevis* and the distantly related African bullfrog, *Pyxicephalus adspersus.* Additional older, internally deleted copies in *X. tropicalis* carry a 300 bp LTR substitution.

**Conclusions:**

XtERV-S represents a genera-spanning member of the largely *env*-less class III ERV that has ancient and modern copies in Anurans*.* This provirus has an *env* ORF with a surface subunit unrelated to known retroviruses and a transmembrane subunit related to class I gammaretroviruses in sequence and organization, and is expressed in early embryogenesis. Additional XtERV-S-related but defective copies are present in *X. tropicalis* and other African frog taxa. XtERV-S is an unusual class III ERV variant, and it may represent an important transitional retroviral form that has been spreading in African frogs for tens of millions of years.

**Supplementary Information:**

The online version contains supplementary material available at 10.1186/s12977-021-00564-2.

## Background

Retroviruses (RVs) are a diverse family of viruses with seven genera. The alpha-, beta-, gamma-, delta-, epsilon-, lenti-, and spumaRVs are distinguished by variations in sequence, genomic organization and life cycle. RVs replicate through a DNA intermediate generated by the virus-encoded reverse transcriptase (RT) [1]. These DNA copies integrate into the genomes of infected cells and can be passed to progeny cells. RVs thus can exist as infectious virions that can be horizontally transmitted through infection, and as endogenous retroviruses (ERVs) that have integrated into the host germline. ERVs represent the relics of past infections, and up to 10% of vertebrate genomes are RV-derived [2]. ERVs are grouped into three clusters largely based on RT sequence relationships to the infectious Retroviridae: class I (gamma- and epsilonRVs) and class II (alpha-, beta-, delta- and lentiRVs) are orthoRVs, and class III is most closely related to spumaRVs [2]. While many ERVs have counterparts among present day infectious RVs, others do not and serve as paleovirological records of extinct, or so far undiscovered, infectious viruses.

After their acquisition, ERVs are inactivated by mutations acquired at the neutral mutation rate of their host genomes. Over extended evolutionary timescales, ERVs accumulate mutations that render them defective, eventually becoming unrecognizable as RVs. Rarely, some ERV domains can be co-opted by their hosts to serve cellular functions, and these sequences are preserved by purifying selection preventing the mutational decay experienced by genetic sequences under neutral selection [3]. Examples of such domesticated genes include viral envelope (*env)* genes co-opted to serve in placenta formation (termed syncytins) [4, 5], *env* and *gag* genes that can serve anti-viral functions like *Fv4* and *Fv1* [6, 7], and regulatory sequences that affect host gene expression [8–10].

As documented through studies on the expanding number of sequenced genomes, ERVs are widely distributed in vertebrates, and genome analyses have catalogued the viral subtypes present in different species [11–13] and have also tracked cross-species transmissions [14–17]. The identification of ancient paleo-retroviruses encountered in this evolutionary record has led investigators to reconstruct the genomes of their progenitors [18, 19], viruses that may not have extant infectious counterparts.

Frogs are a diverse and mainly carnivorous subgroup of amphibians. They are classed in the vertebrate order *Anura* which dates to the Permian, 265 million years ago. Frogs show a wide geographic distribution and occupy diverse habitats ranging from the tropics to subarctic regions, although most species are found in tropical rainforests. There are at least 5424 recorded species, making them one of the five most diverse orders of vertebrates [20].

*Xenopus*, commonly known as the clawed frog, is a genus of aquatic frogs native to sub-Saharan Africa. Of the twenty-nine *Xenopus* species, the most well-studied are *Xenopus laevis* and *Xenopus tropicalis* (formerly *Silurana tropicalis*). *X. laevis* has been extensively used as a vertebrate model in developmental biology, cell biology, toxicology, neuroscience and gene expression, but its usefulness in genetic studies and for genetic manipulation has been complicated by its allotetrapoid genome (2n = 36). *X. tropicalis* offers advantages as an experimental model system as it is a smaller frog with a shorter generation time, and, because it is the only one of the 29 extant *Xenopus* species with a diploid genome (2n = 20), *X. tropicalis* was the first *Xenopus* species selected for genomic sequencing [21]. The subsequent sequencing of *X. laevis* has extended its utility by providing a model for the evolution of vertebrate polyploidy [22]. Analyses of these two genomes found a high diversity of transposable elements, including four superfamilies of LTR retroelements [21, 22].

In the course of screening non-mammalian vertebrates for conserved and functionally important RV domains, we identified an unusual 8.0 kb ERV in *X. tropicalis* that we termed XtERV-S because it has a serine tRNA primer binding site (PBS)*.* This ERV has *gag, pol* and *env* genes with open reading frames (ORFs), one additional ORF in *pol* and identical 5’ and 3’ LTRs, suggesting it is a recent germline acquisition. Older intact but defective and nonorthologous copies are also present in *X. laevis* and the African bullfrog, *Pyxicephalus adspersus*. XtERV-S is expressed during early development, its Gag protein shows expected cellular localization, and its LTR shows some activity in human 293T cells. Phylogenetic and functional motif comparisons indicate that the XtERV-S *pol* and *gag* genes are related to the ancient class III family of ERVs represented by ERV-L. However, XtERV-S, unlike mammalian ERV-Ls, has an *env* with an ORF. The surface subunit of this *env*, SU*env*, is not related to known RVs although its transmembrane subunit, TM*env,* is class I-like. The sequence homologies, presence of viral genus-specific functional motifs, and the distribution of older copies in other African frog species indicates that XtERV-S is a genera-spanning ancestral form that has been circulating in these species for at least 36 million years.

## Results

### Identification of the *X. tropicalis* endogenous retrovirus XtERV-S

**Fig. 1:**
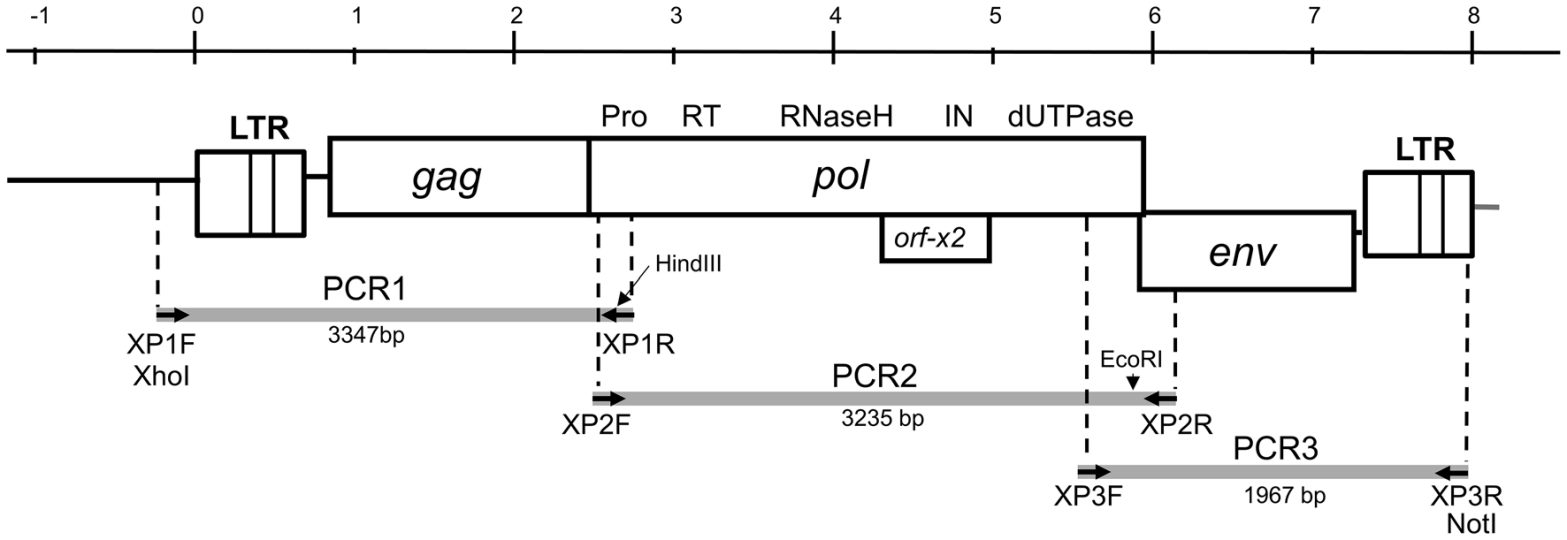
Cloning of XtERV-S. Positions are shown for PCR products PCR1-3 (grey boxes) and the locations of the primers (dashed lines and black arrowheads). Also shown are the PCR product sizes and the positions of restriction sites used for cloning and assembly of the provirus in the pBluescript SK(+) vector

An intact provirus, XtERV-S, was initially identified in an unplaced scaffold in the sequenced genome of *X. tropicalis* (NW_016684263.1:c1706-9791 *X. tropicalis* unplaced genomic scaffold_1181, *X._tropicalis*_v9.1). A molecular clone of the full-length provirus was assembled from three overlapping PCR products (Fig. 1; Table 1). The 5′ PCR fragment includes flanking sequence that corresponds to the scaffold sequence and maps to chromosome 7 (XTR7; NC_030683.2:127894395-127895901) in the most recent assembly (UCB_Xtro_10.0).

**Table 1:** Primers used for cloning the XtERV-S provirus and for expression studies

Primer	Sequence (5′–3′)
Cloning XtERV-S	
XP1 F	CTC GAG GAT TCC CCA AGT CAC ATG AGA TG
XP1 R	GCG GCC GCG ACT TAC TGC TGG CCG AAA TAC TC
XP2 F	CTC GAG CTG TCA ATC TCA AGC AGT ATC GCA TAC
XP2 R	GCG GCC GCC CAC TCA GGG TCG GTT CTG TTT ATC CAA CTC
XP3 F	CTC GAG TAA TGT AAC AAA CTG TTG GAT ATG TG
XP3 R	GCG GCC GCT GTA ATA AAG GGG TTA ACC TTT ATC
LTR cloning into pGL3 basic	
XtERV-S LTR FW	GGT ACC TGA TTT GTA TGA TTT ACA ATT TAT ACA TG
XtERV-S LTR RE	GGA TCC GTA ATA AAG GGG TTA ACC TTT ATC
MoMLV LTR FW	GGA TCC TGA AAG ACC CCA CCT GTA GGT TTG GCA
MoMLV LTR RE	AAG CTT TGA AAG ACC CCC GCT GAC GGG TAG TC
Gag cloning into eGFPC1	
Gag FW	GGA TCC TTA TGT TCT GCT GGT TAA AAA ACA AGG TTA G
Gag Rev	CTC GAG ATC TAT AGG ACT GGG CAC CTC

Underlined regions of the sequence indicate the restriction sites used for cloning

**Fig. 2:**
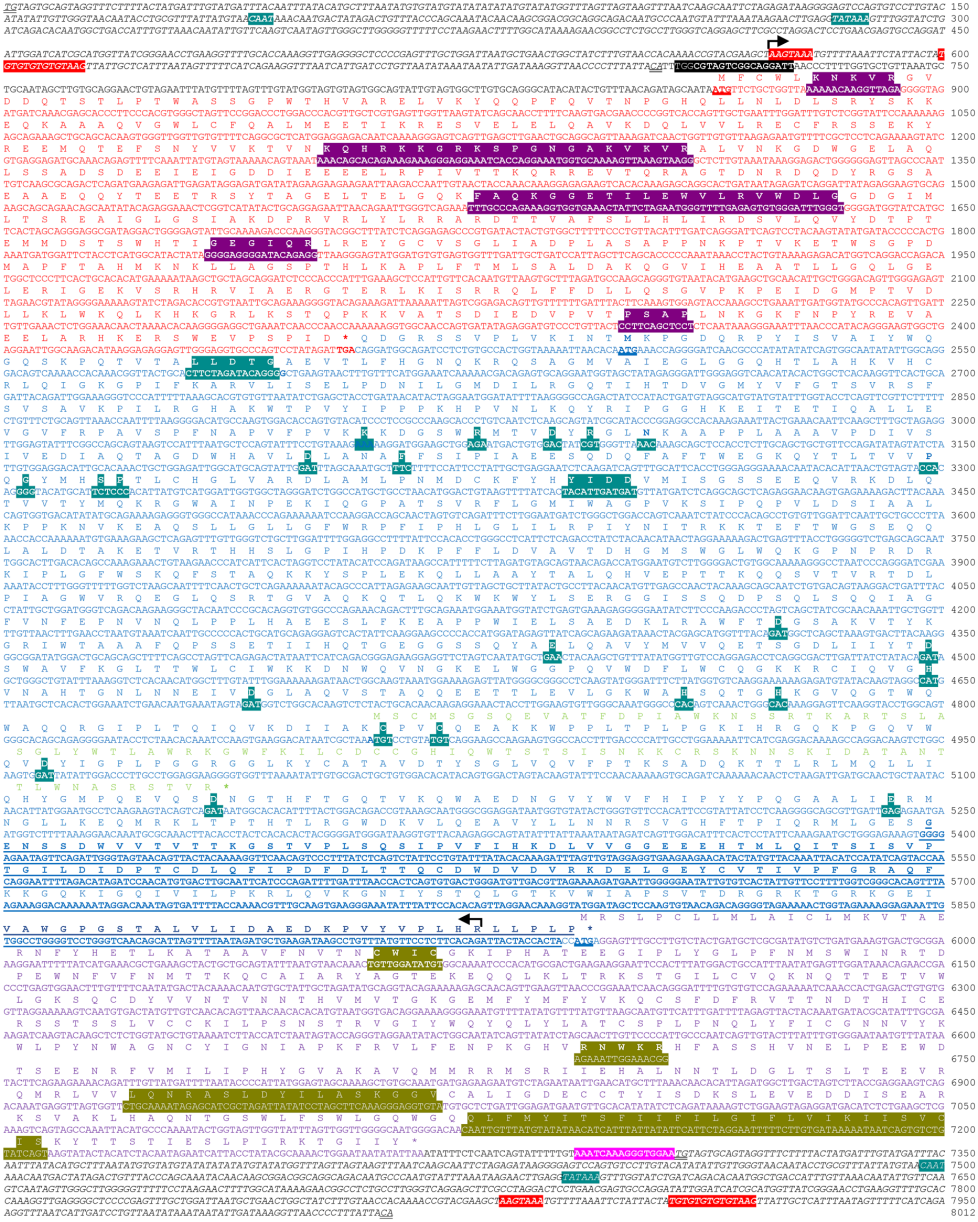
The complete nucleotide and deduced amino acid sequence of the XtERV-S proviral genome. The sequence is shown from the beginning of the 5′ U3 region to the end of the 3′ U5. The LTR sequence is in black italics and its inverse repeats are double underlined. The *gag*, *pol* and *env* ORFs are in red, blue and purple, respectively, and termination codons are marked by an asterisk. The Orf-x2 sequence is in light green. The positions of the functional motifs are bolded and highlighted and include the following in order: PBS (primer binding site); basic regions of Gag; MHR (major homology region); GQR motif; PSAP late domain; PR (protease); RT/RNH (reverse transcriptase/RNase H); CWIC (isomerase domain); furin site; ISD (immunosuppressive domain); MSD (membrane spanning domain); PPT (polypurine tract); polyA (polyadenylation signal). Arrows indicate the splice donor and acceptor sites. The dUTPase region of *pol* is underlined and bolded

XtERV-S is 8012 bp in length predicting a packaged genome of 7597 bp (Fig. 2). The coding regions have no fatal stop codons. XtERV-S has a genomic structure similar to that of simple RVs: LTR-*gag-pro-pol-env*-LTR (Figs. 1, 2), with a novel additional ORF in *pol*. The *gag* and *pol* regions are separated by an in-frame stop codon analogous to the organization found in mammalian gamma- and epsilonRVs, where expression of *pol* occurs through translation suppression of the *gag* termination codon. The Env protein is likely expressed from a spliced transcript from a start site that overlaps the *pol* stop with a -1 frameshift. The genome contains the functional motifs common to all RVs and has some motifs diagnostic of specific RV genera (Fig. 2; Table 2).

**Table 2:** Key functional motifs that are present, absent or variant in XtERV-S and related RVs

Protein	Feature	Consensus sequence	Motif presence, absence or sequence variation (viral genome position)^a^			
			XtERV-S	ERV-L	FV	MLV
Gag						
Matrix (MA)	Myristylation signal	GX_2-3_[S/T]	–	–	–	GQTVT (2–6)
Capsid (CA)	Major homology region (MHR)	QX_3_EX_4_ΦX_2_R	(238–257)	(281–300)	–	(357–376)
Nucleocapsid (NC)	Cys-His motif	CX_2_CX_4_HX_4_C	–	–	–	(503–511)
	glycine-glutamine-arginine (GQR) domain	GX_0-3_QR	GEGIQR (324–331)	GQR (471–473)	–	–
NC or P6	Late domain	PXXP, PPXY or YXXL	PSAP (496–499)	PTAY (520–523)	PSAP (284–287)	PPPY (162–165)
Pol						
Protease (Pro) (*Pfam* 13975)	Catalytic motif	D[T/S]G	DTG (44–46)	DTG (54–56)	DSG (24–26)	DTG (27–29)
Reverse transcriptase (RT) (*cl02808*)		D[YLF]RX_2_NX_66-71_G	DYRGLN_66_G (211–283)	DYRKLN_55_G (210–271)	DYREVNX_64_G (217–287)	DLREVN_71_G (234–311)
	Catalytic center	Y[V/L/I/M]DD	YIDD (312–315)	YIDD (312–315)	YVDD (312–315)	YVDD (342–345)
RNaseH (*cl14782*)	Active site/dead box	DEDD or DEDHD	DEDD (623–745)	DEDD (621–751)	DEDD (599–740)	DEDD (644–773)
dUTPase			(987–1162)	(1018–1105)	–	–
Integrase (IN) (*Pfam* 00665)	Zinc finger domain	HX_3-7_HX_23-32_CX_2_C	HX_4_HX_28_CX_2_C (768–805)	HX_4_HX_28_CX_2_C (774–811)	HX_3_HX_29_CX_2_C (818–850)	HX_3_HX_32_CX_2_C (848–888)
	Catalytic core	DX_39-58_DX_35_E	DX_58_DX_35_E (834–929)	DX_58_DX_35_E (838–933)	DX_58_DX_35_E (916–1011)	DX_56_DX_35_E (879–972)
Env						
Surface unit (SU)	Isomerase domain	CX_2_C	CWIC (40–43)	–	–	CWLC (336–339)
	N-linked glycosylation sites	NX^P[S/T]	8 SU, 1TM	–	2 LP, 10 SU, 3 TM	7 SU, 1 TM
	Furin site	K/R-X-K/R-R	RNWKR (251–255)	–	–	RHKR (466–469)
Transmembrane unit (TM)	“CX6CC”	CX_6_CC	(344–352)	–	–	(555–563)
	Heptad repeats	ΦX_2_ΦX_3_	+	+	+	+
	“Stutter” glycosylation site		306	–	–	–
	Immunosuppressive Domain		(327–341)	–	–	(538–551)
	Membrane spanning domain		(397–417)	–	–	(606–631)
	Cytoplasmic tail		(419–441)	–	–	(633–665)
	Endocytosis signal	YXXΦ	YTTS (424–427)	–	–	YHQL (655 – 658)

^a^Genome positions for most XtERV-S motifs are marked in Fig. 2; positions for the three RV prototypes are based on GenBank numbers Y12713 (ERV-L), Y07725 (FV), J01998 (AKV MLV). MHR sequences provided in Additional file 6: Fig. S6

### LTR

XtERV-S has LTRs of 705 bp that are 100% identical and flanked by the trinucleotides 5′-TGT and 3′-ACA, integrase recognition motifs conserved in class III ERVs [23]. The 3’ cellular flank could not be amplified due to the highly repetitive downstream sequence in scaffold_1181, so we could not identify a target site duplication (TSD). Both LTRs contain recognizable promoter and polyadenylation signals (Fig. 2). The presumptive core promoter has a CAAT box (position 197–200), a GC dinucleotide (position 221–255) and a TATAA box (283–287). The CAAT and TATAA boxes are 78 nt apart. A polyadenylation signal (position 574–579) is followed by a GT-rich sequence stretch (position 601–610) typically required for binding of the CstF (cleavage stimulatory factor), responsible for cleavage of RNA and addition of poly-A tails. Upstream of the 3′ LTR is an AG-rich polypurine tract (PPT). XtERV-S has a short 158 bp leader region downstream of the 5′ LTR. The 3′ end of the LTR is followed by a tRNA-related primer binding site (PBS) complementary to the 3′-terminal 19 nucleotides of tRNA^Ser(AGA/TGA)^.

### *gag*

The XtERV-S *gag* ORF encodes a putative 530 amino acid (aa) protein of approximately 60 kDa (Fig. 2) that is related to ERV-L type *gag* genes (see below). While XtERV-S does not have distinguishable matrix (MA), nucleocapsid (NC) and capsid (CA) proteins, it contains key functional motifs found in ortho- and/or spumaRV Gag proteins (Fig. 2; Table 2). These motifs include the “late” or “L” domain motif, PSAP, required for virus budding and release, and the major homology region (MHR) found in orthoRV but not spumaRV CAs [24, 25].

The 5’ end of the XtERV-S Gag lacks a myristoylation signal that functions in some RVs to target Gag to the plasma membrane [26]; instead, it contains polybasic regions (aa positions 6–10 and 130–149) (Fig. 2), which are also found in various RVs where they mediate MA/plasma membrane interactions [27, 28]. A zinc finger Cys-His box motif present in 1–2 copies in the NC of all orthoRVs functions in RNA binding, but is absent from XtERV-S and spumaRVs [29]. Instead, XtERV-S Gag contains a single glycine-glutamine-arginine (GQR) domain (Fig. 2; Table 2); this motif is also present in fish foamy virus (FV)-like ERVs and is hypothesized to function in nucleic acid binding and nuclear localization analogous to the GR boxes found in infectious FVs [30–32].

### *pro*,* pol*

The organization of the deduced XtERV-S Pol sequence is typical of gamma-, epsilon-, spuma- and lentiRVs with the order: PRO-RT-RNAseH-IN (Fig. 2). *pro* is in the same reading frame as *pol*, which is characteristic of gamma-, epsilon-, spumaRVs and class III ERV-L, but not lentiviruses.

RV Pol proteins can be alternatively produced by readthrough suppression, ribosomal frameshifting, or, in the case of spumaRVs, use of a separate start codon for *pol*, which is in a different reading frame. The XtERV-S *gag* and *pol* genes are in the same frame and are separated by a stop codon, TGA, that can be subject to translational suppression (Fig. 2) [33, 34]. The *pol* ORF is thus predicted to start at or before the *gag* stop codon at position 2457.

*Pro-pol* spans 3489 bp, potentially encoding a 1162 aa polyprotein (Fig. 2). This region contains the conserved and properly spaced key residues for common functional motifs [35–37] (Table 2). Pro contains a catalytic region with the active aspartate site (DTG) and the active site flap (amino acid position 66–76) [38–40]. The *pol* gene encodes, in order: RT, a tether domain derived from a second degenerate RNaseH-related sequence [41], RNAseH and IN. The RT catalytic domain uses YIDD as the active YXDD site, which is typical of class III ERVs like ERV-L, but not FVs (Additional file 1: Fig. S1).

The XtERV-S *pol* includes an additional ORF of 276 bp in the -2 reading frame within IN. This ORF substantially overlaps the position of the *orf-x* sequence first identified in JSRV [42], with comparable ORFs in ERVs of other species like the bat DrERV and armadillo DnERV (Env1.1) [43, 44] (Additional file 2: Fig. S2). However, the XtERV-S ORF, *Orf-x2*, is shorter, with a 5′ end truncated by a stop codon, and has little sequence homology to the others.

A dUTPase gene is found in some RVs, but is located in different positions in four lineages: within *pro* in betaRVs, upstream of IN in nonprimate lentiRVs, after IN in some endogenous ERVs or at the 5′ end of *gag* in some Equid ERVs [45, 46]. Homology modeling using I-TASSER [47] of the XtERV-S Pol identified a segment positioned after IN as having structural similarities to other viral UTPase proteins (PBD 3ZEZ and PBD 5Y5O), and this position is common to class III ERVs, like ERV-L (Fig. 2).

### *env*

**Fig. 3:**
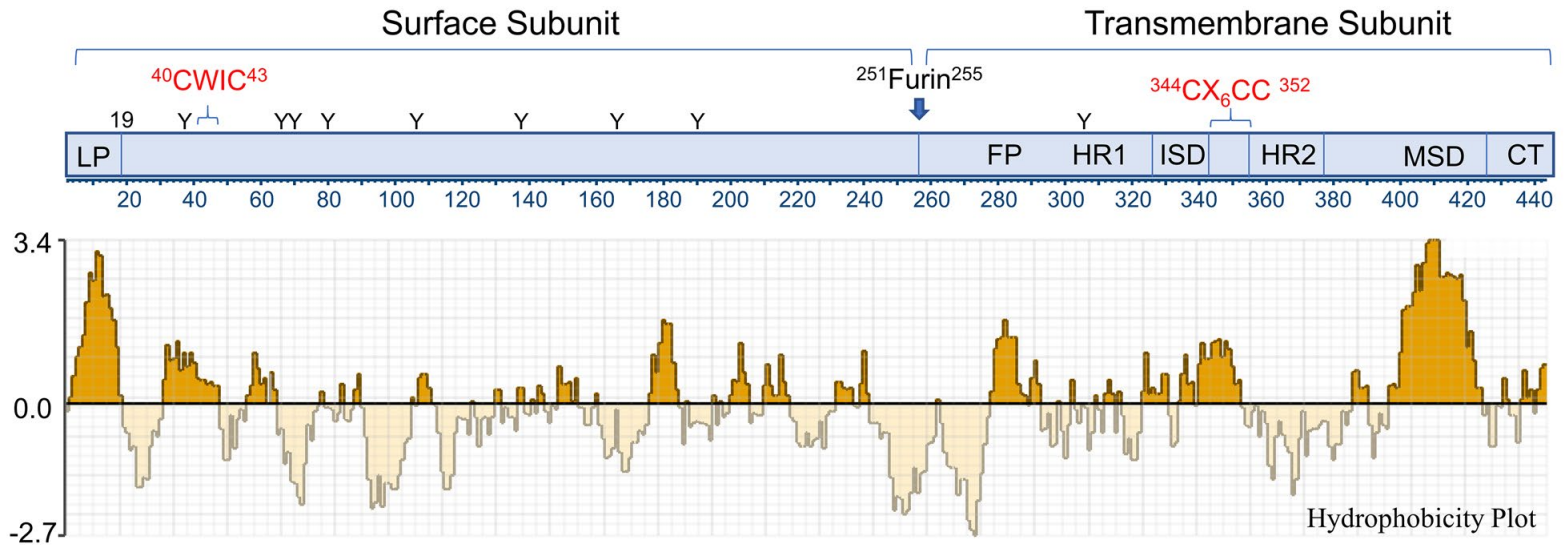
Hydrophobicity plot of the XtERV-S Env. The SU and TM subunits of envelope are separated by a furin site (RNWKR) at position 251–255. The SU CWIC domain (position 40–43) and its interacting TM CX_6_CC (position 344–352) domains are indicated in red. The TM subunit contains the following: FP (fusion peptide), two heptad repeats (HR1, HR2), ISD (immunosuppressive domain), MSD (membrane spanning domain), CT (cytoplasmic tail). N-linked glycosylation sites are marked with a Y

The *env* ORF encodes a putative 441 aa precursor with obvious surface (SU) and transmembrane (TM) domains along with a 19 residue signal peptide (Fig. 3). Based on ESTs such as GenBank # CF222458.1 and Genbank *ab inito* gene prediction bioinformatics tools, there are potential splice donor/acceptor *env* sites at bases 576 and 5928 (Fig. 2). This positioning is unusual for RVs because the resulting transcripts would not contain the PBS; this configuration is typical of spumaRVs but not orthoRVs [48]. The *env* start overlaps the *pol* stop and is in the -1 reading frame. The SU and TM domains of RVs are typically cleaved by the furin protease at the consensus site K/R-X-K/R-R; XtERV-S contains a similar but nonstandard sequence, RNWKR, at the putative N-terminus of TM (Fig. 2).

The XtERV-S SU shows no discernible sequence homology to known RVs or ERVs, but its TM resembles gammaRVs in having an immunosuppressive domain (ISD) and a CX_6_CC motif that functions to establish a covalent disulfide link with a CXXC motif in the Env SU [49, 50]. The XtERV-S SU has a CWIC element positioned near the SU N-terminus (Fig. 2). TM has the domain structure typical of gamma- and alphaRVs (Table 2). A hydrophobic stretch is the likely fusion peptide but is 22 residues downstream of the putative furin site, an organization that is characteristic of alphaRVs, although the the alphaRV peptide is flanked by C residues not present in XtERV-S. The fusion peptide is followed by an N-heptad repeat, an ISD, a chain reversal region containing CX_6_CC, and a C-heptad repeat [50]. The ISD contains the sequence QNRAA/SLD which is typical of nonmammalian gammaRVs [51]. The TM ectodomain is followed by a membrane spanning motif [52] (Fig. 3) and an unusually short cytoplasmic tail of 27 residues [53].

RV *env* genes have 4–30+ potential N-linked glycosylation sites; XtERV-S has eight in SU and one in TM. In some gammaRVs, the first heptad repeat pattern in TM is disrupted by a “stutter” that is associated with the presence of a glycosylation site [51]. This “stutter”-associated glycosylation site is not found in infectious mammalian RVs but is present in XtERV-S and is also present in some fish FVs, some alphaRVs [51], some mammalian syncytins, and some other non-RV virus envelopes [54–57], as also shown below.

### XtERV-S related sequences in *X. tropicalis* and other species

The most recent *X. tropicalis* genome assembly (UCB_Xtro_10.0) contains two different full length XtERV-S-related copies on chromosome XTR4: XtERV-S2(Xt-S2) (8009 bp; NC_030680.2:c12439548-12431540) and XtERV-S3 (Xt-S3) (7961 bp; NC_030680.2:c11872885-11864925). Xt-S2 and Xt-S3 differ from XtERV-S at 28 and 95 nt positions, respectively (Additional file 3: Fig. S3). Xt-S2 carries intact open reading frames for Gag, Pol and Env proteins but has an in-frame deletion in Pol relative to XtERV-S, while Xt-S3 has fatal mutations in *gag*, *pol* and *env*. These XTR4 ERVs have nearly identical flanking sequences including the same target site duplication, CCCTA, consistent with a local genomic duplication. A 5 bp TSD is also characteristic of ERV-L. The LTRs of Xt-S2 and Xt-S3 differ by 1 and 7 nts, respectively indicating their recent acquisition (Additional file 8: Table S1).

**Fig. 4:**
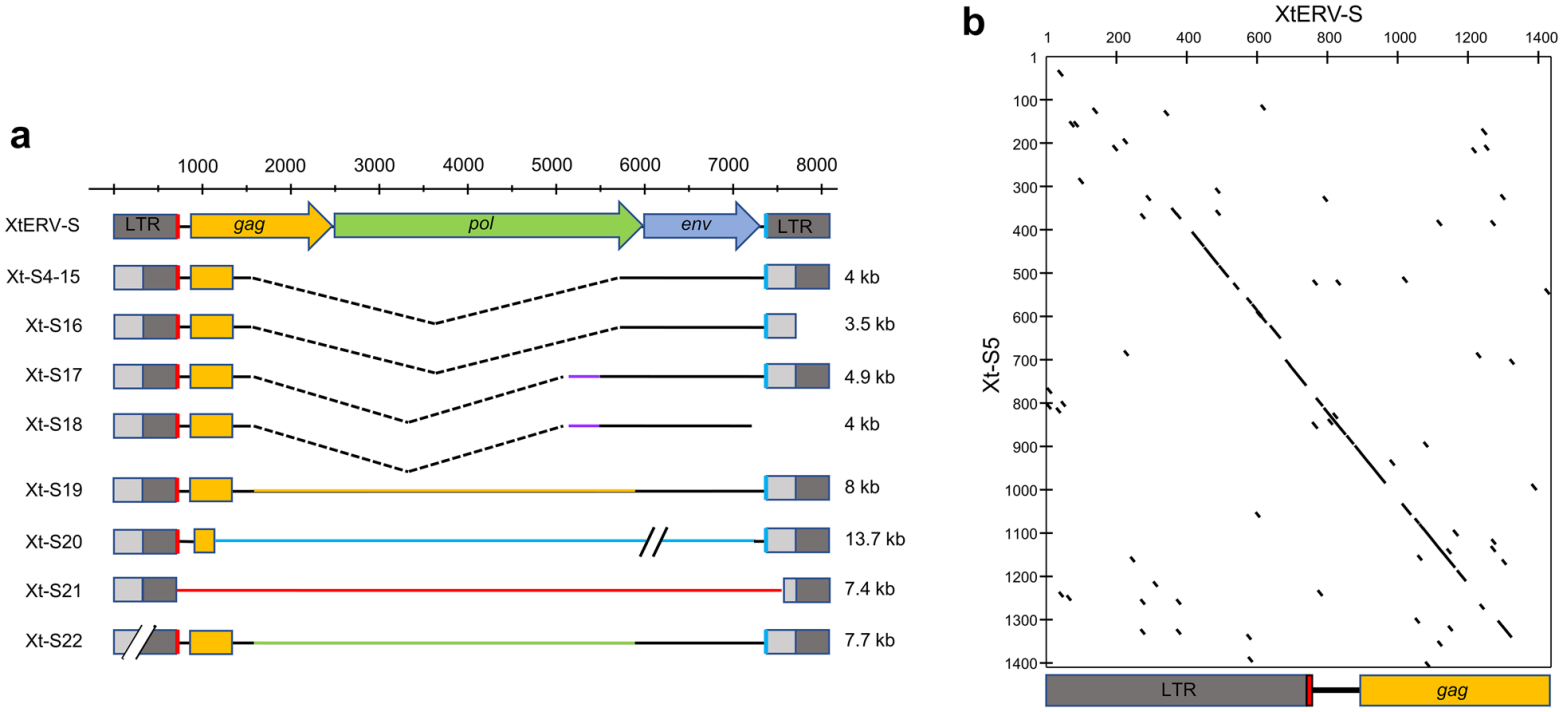
Additional XtERV-S-related copies in *X. tropicalis*. **A** Schematic representation of XtERV-S-related deleted ERVs. Most of the 19 deleted copies are present in a single copy while Xt-S4—Xt-S14 are structurally similar. Identical line and bar colors represent sequence similarities. Dotted lines represent deletions. **B** Dot plot comparison of XtERV-S and the 5′ end of Xt-S5 (~ 1400 nt) shows similarities in the 3′ half of the LTR and the N-terminal region of *gag*

The *X. tropicalis* genome also contains 19 additional related, but deleted copies (Xt-S4–Xt-S22) having at least two LTR sequences and some internal sequence that usually includes the PBS, *gag* leader, the 5’ end of *gag* and the 3’ PPT (Fig. 4A; Additional file 8: Table S1). These insertions are all flanked by a 5 bp TSD (Additional file 8: Table S1). The LTRs in these copies are nearly identical to the XtERV-S LTR in the 3′ half, but have a 5′ 296 bp replacement (Fig. 4B). Based on LTR differences, the oldest of these copies was acquired ~ 25 mya while others are more recent acquisitions (Additional file 8: Table S1). There are also more than 140 solo LTRs with this altered LTR sequence (Additional file 8: Table S1).

**Fig. 5:**
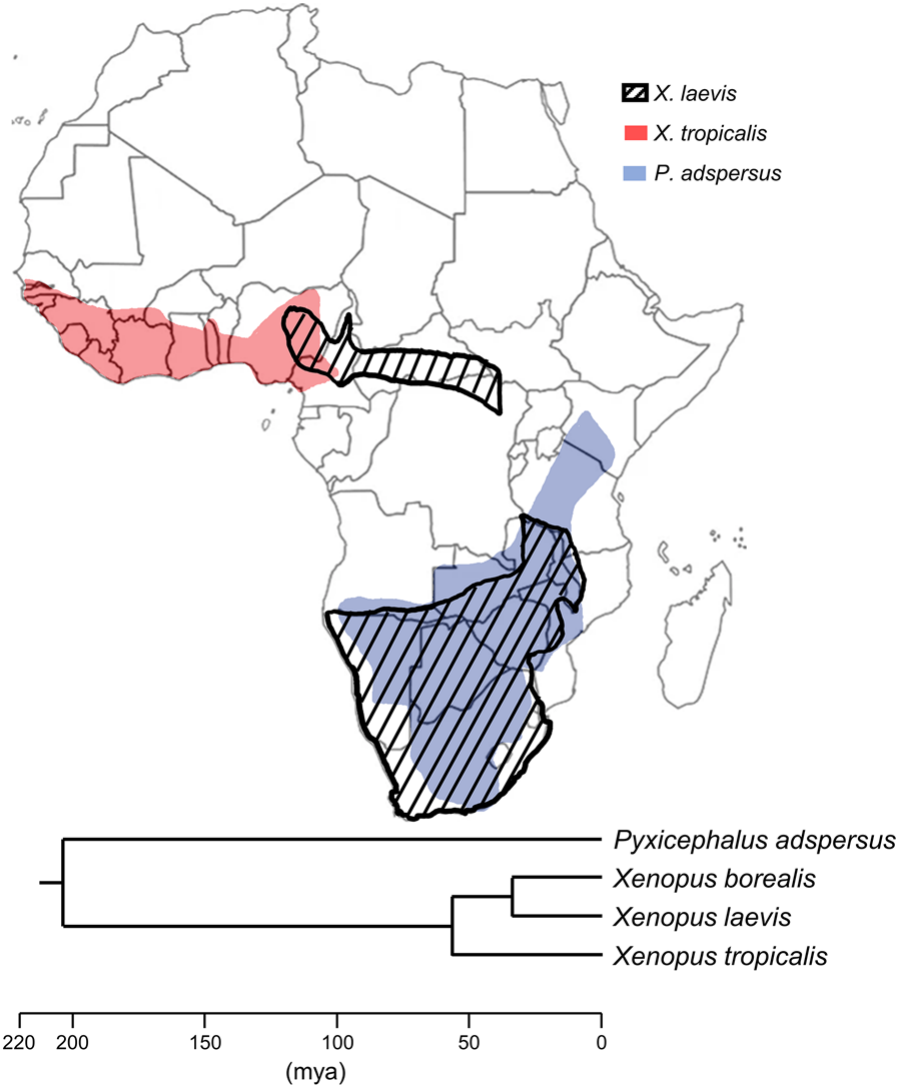
Geographic distribution of *X. tropicalis*, *X. laevis* and *P. adspersus*. The areas represented by red and blue highlighting and black stripes represent the natural habitats of *X. tropicalis*, *P. adspersus* and *X. laevis*, respectively [98]. The phylogenetic tree from Timetree [58] places the divergence of *P. adspersus* and *Xenopus sp*. at ~ 204 mya and the divergence of two *X. tropicalis* and *X. laevis* at 57 mya

BLAST searches identified XtERV-S-related sequences in the *X. laevis* genome [22]. The two *Xenopus* species diverged 57 mya [58], and have minimally overlapping geographic ranges (Fig. 5). The single full-length *X. laevis* copy, XlERV-S (NC_030735:53003809-53021418), is 82% identical to XtERV-S but all three coding regions contain frameshifting deletions and insertions (Additional file 4: Fig. S4). Most notably, there are insertions of 3975 bp in *pol,* and 315 and 1924 bp in *env*. The two LTRs resemble the XtERV-S LTRs in the 3’ half and the 5’ half has no equivalent in *X. tropicalis*. Differences in these LTRs provide an age estimate for XlERV-S of 36 my (Additional file 8: Table S1). *X. laevis* is allotetraploid with two sets of chromosomes, L and S, that are homeologous and co-orthologous to *X. tropicalis* chromosomes and originated from the interbreeding of frogs with distinguishable genomes 34 mya [22]. XlERV-S maps to XLA6S and is therefore not orthologous to XtERV-S or to any of the deleted XtERV-S copies on its chromosome 6, XTR6. The *X. laevis* genome also carries more than 100 related solo LTRs.

**Fig. 6:**
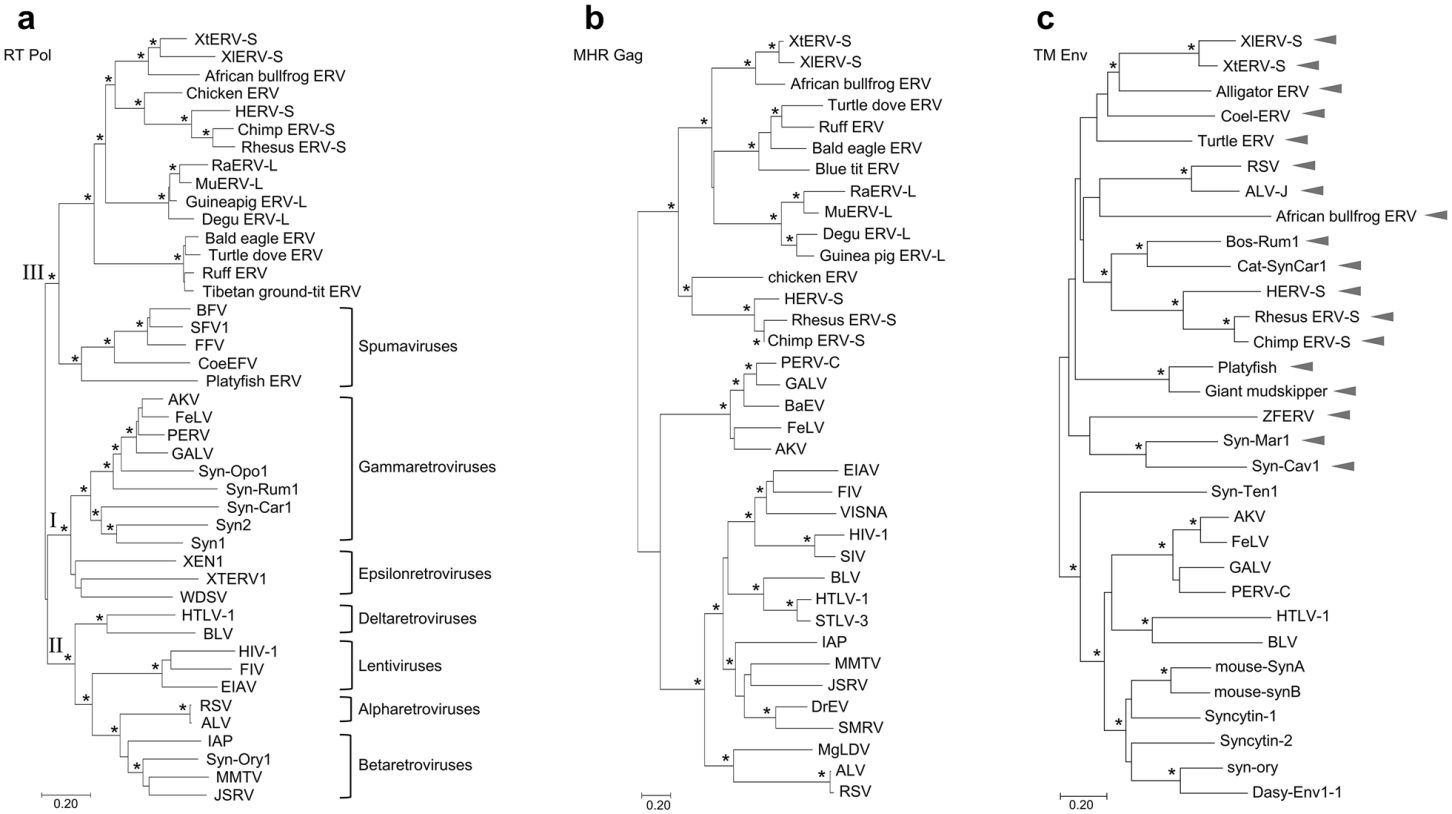
Unrooted phylogenetic trees of representative retroviruses (Additional file 9: Table S2) based on a MUSCLE multiple alignment and neighbor-joining method. Asterisks indicate bootstrap values greater than 70. Horizontal branch lengths are proportional to the degree of amino acid substitutions per site. The three trees represent RTpol (**A**), the MHR region of CA*gag* (**B**) and a segment of TM*env* (**C**). The RT tree identifies the clusters representative of the seven RV genera. The arrowheads in the TM*env* tree identify sequences with the N-linked glycosylation site associated with a heptad “stutter” (Additional file 7: Fig. S7)

BLAST searches of other frog genomes identified an intact related provirus in another African Anuran, the African bull frog (*P. adspersus* isolate 1538 chromosome 4, CM016419:110619499-110628269) (Additional file 5: Fig. S5). This genus diverged from *Xenopus* approximately 200 mya and is sympatric with *X. laevis* (Fig. 5). This provirus has full-length but defective *gag*, *pol* and *env* genes, and the predicted proteins show high similarity to XtERV-S in Gag (51% identical and 71% similar: Blossum90) and Pol (67.4% identical and 84.2 similar: Blossum90). This ERV has a leucine PBS and a dUTPase positioned as in XtERV-S and ERV-L. The Env protein shows no homology to XtERV-S in SU, but contains a CWIC motif in a comparable location near the SU N-terminus. The gammaRV-like TM has a putative ISD, a CX_6_CC motif and a “stutter” in its heptad repeat but is only distantly related to the *Xenopus* ERVs (Fig. 6). The LTRs are 504 bp and show no significant similarity to the LTRs of XtERV-S or ERV-L, and the LTR differences produce an age estimate of 15 mya (Additional file 8: Table S1).

### Phylogenetic analysis of XtERV-S

Segments of RV genomes can have different phylogenetic histories because RV recombination is common and can involve distantly related RVs [16, 59, 60] or can occur between endogenous and exogenous viruses [61]. XtERV-S has a class III *gag-pol* and a class I *env*. We generated phylogenetic trees based on alignments of three regions of the genome: the RT core of *pol,* the MHR-containing region of *gag*, and the TM domain of *env,* including representative members of the seven retroviral genera where possible, and previously described and newly extracted ERVs from the genomes of nonmammalian vertebrates (Fig. 6; Additional file 9: Table S2).

The RT core is the most highly conserved region across all seven RV genera and this tree shows three groupings that correspond to the class I-III ERVs (Fig. 6A). XtERV-S is not closely related to the two previously identified *Xenopus* ERVs, XTERV1 and Xen-1 [62, 63], which are epsilonRVs. The XtERV-S RT clusters with the other African frog ERVs and with class III which includes ERVs from fish, amphibians and birds, FVs, and mammalian ERV-L and ERV-S. RT alignments identify obvious ERV-L lineage specific sequence stretches in XtERV-S (Table 2; Additional file 1: Fig. S1).

The *gag* gene is poorly conserved among RVs, but XtERV-S contains an MHR, shared by most orthoRVs and ERVs, and there are lineage specific sequence patterns in and around the MHR (Additional file 6: Fig. S6). This tree also groups the XtERV-S segment with ERV-L (Fig. 6B).

XtERV-S encodes a TM*env* typical of class I gammaRVs and ERVs [50]. This tree defines two subgroups (Fig. 6C) with XtERV-S grouping with some nonmammalian gammaRVs and several syncytins. The subgroup containing XtERV-S includes all of the TMs with the heptad-stutter associated glycosylation site (Fig. 2; Additional file 7: Fig. S7) [51].

### Expression of XtERV-S in vivo and in cultured cells

**Fig. 7:**
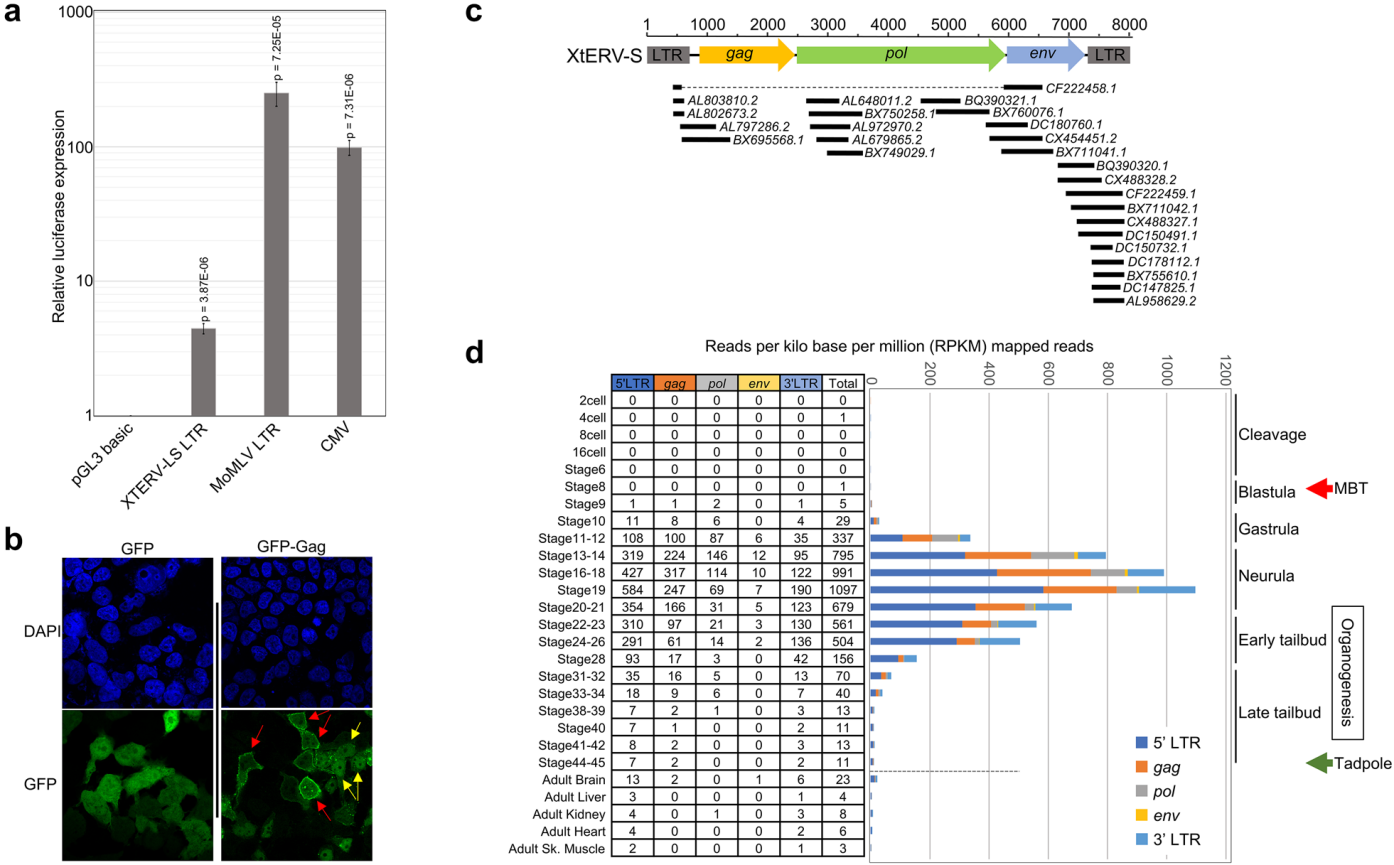
XtERV-S is transcriptionally active. **A** Functional analysis of the XtERV-S LTR cloned into a luciferase reporter vector and transfected into 293T cells. Luciferase expression is compared to the promoter-less vector pGL3 basic and to vectors using the Moloney mouse leukemia virus (MoMLV) LTR and CMV promoter. **B** Confocal examination of GFP-tagged XtERV-S Gag protein in 293T cells shows accumulation in the cytoplasm at the plasma membrane (red arrows) and localization in nucleus (yellow arrows). **C** ESTs mapping to XtERV-S are expressed embryonically in *X. tropicalis*. ESTs map to the *gag*, *pol* and *env* regions. **D** RNAseq reads map mostly to LTR and the *gag*-*pol* ORFs. The chart shows the total number of reads per kilobase per million (RPKM) mapped to the provirus and the reads mapping to LTR, *gag*, *pol*, and *env*. The developmental stages and events are indicated, including MBT (mid-blastula transition); the red arrow represents the transcriptional transition from maternal mRNA to zygote genome transcribed mRNAs; the green arrow represents the beginning of the tadpole stage

The presence of intact and correctly positioned CAAT and TATA boxes along with a polyadenylation signal within the LTRs strongly suggests that XtERV-S can be transcribed. We cloned the XtERV-S LTR into a luciferase reporter vector. In the absence of established cell culture systems to test for *Xenopus* gene expression, we used human 293T cells and found that the XtERV-S LTR increased luciferase expression by four–fivefold compared to promoter-less reporter (Fig. 7A), but was 20–60 fold lower than the MoMLV and CMV promoters (Fig. 7A). This reduced expression directed by the XtERV-S LTR may be due to its partial mutational inactivation or to some incompatibility of this LTR in 293T cells.

The XtERV-S *gag* with a GFP tag can express stable protein under the constitutive CMV promoter in transfected 293T cells (Fig. 7B). This protein accumulated in the cytoplasm at the plasma membrane, but also distributed to the nucleus. The Gag of multiple RVs is primarily found in the cytoplasm of infected cells, but for some RVs can be distributed to the nucleus, and in late stages of the viral life cycle accumulates at the plasma membrane for assembly [64]. ERV-L Gag has been found in the cytoplasm [65].

Expressed sequence tags (ESTs) related to XtERV-S and RNAseq data document the production of XtERV-S *gag, pol* and *env* transcripts (Fig. 7C, D), indicating that the LTR promoter is transcriptionally active in vivo. ESTs mapping to XtERV-S are detected from gastrulation to the tailbud embryo. These ESTs correspond to segments of the *gag, pol, env* and LTR (Fig. 7C). We mapped the RNAseq reads from the publicly available RNAseq datasets of adult tissues and distinct developmental stages to the XtERV-S genome (301–7916 bp) [66] (Fig. 7D). Most reads mapped to *gag*, *pol* or LTR (Fig. 7D).

The RNAseq data shows little or no expression of XtERV-S transcripts in adult *X. tropicalis* tissues including brain, liver, kidney heart, skeletal muscles and in 2 cell to stage 8 embryos (Fig. 7D). Initiation of XtERV-S transcription thus coincides with the maternal-to-zygotic mid-blastula transition (MBT) characterized by large scale activation of the zygotic genome (onset of transcription from the embryonic/zygotic genome) and destabilization of maternal mRNAs (stage 9; Fig. 7D) [67]. There is increasing expression during development stages 9 and 10 (late blastula—early gastrula) and robust expression through stages 11–28 (mid-gastrula, neurula and early tailbud). Expression decreases subsequent to stage 28 with little or no expression by stage 44–45 (late tailbud–tadpole) suggesting a developmental role and/or regulation of these transcripts. Embryonic expression of XtERV-S in mid-stage embryos differs from that of mouse ERV-L which peaks at the 2 cell stage and decreases at the 8 cell stage [65, 68]. A previous genome wide analysis that examined expression of *X. tropicalis* LTR retroelements similarly found that expression of this set of retroelements is activated at mid-blastula [69]. These data taken together show that XtERV-S is likely transcribed, that transcription is particularly active during specific stages of development, and that tagged transfected *gag* can produce protein that shows the expected cellular distribution. We do not however have evidence that infectious XtERV-S is produced.

## Discussion

XtERV-S is a novel, intact RV ERV with unusual domain relationships to known RVs. It shows closest sequence homology in *gag*-*pol* with the ancient class III ERVs, while its *env* gene has an SU*env* subunit that is unrelated to any known RV and a TM*env* characteristic of class I gammaRVs in organization and functional motifs. Recombinant structures are common among RVs, and multiple instances of *env*-swapping have described the acquisition of class I *envs* by class II RVs isolated from multiple species [16, 59, 70] as well as *env*-swapping between different subgroups of class I RVs [71, 72], a phenomenon which occurs regularly during MLV-induced lymphomagenesis [61]. XtERV-S is thus an unusual example of an intact and apparently nondefective ERV genome with a class I *env* in a class III backbone. The TM*env* subunit of this virus has the motifs necessary to establish a covalent disulfide SU/TM bond, an obviously successful and ancient *env* structure that is common in other virus families including filoviruses, influenza and coronaviruses [73–75]. XtERV-S may thus represent an ancient evolutionary RV form with a combination of viral genes not found in extant mammalian RVs but that may still be circulating in African frogs. This RV structure may be prove to be common in ancient ERVs and representative of infectious RVs yet to be discovered.

The ERVs related to this sequence in the genomes of various African frogs include recent and ancient copies. The intactness of the XtERV-S ORFs and its identical 5′ and 3′ LTRs show it to be a recent insert in the early stages of retroviral endogenization. On the other hand, the divergent LTRs of the mutationally damaged nonorthologous copy found in XlERV-S date it to 36 MYA, and similarly ancient ERVs that are related but not identical are found in the African bullfrog (*P. adspersus)*. These data suggest that related infectious RVs have long been spreading in Anuran populations and were fairly recently active in *X. tropicalis,* although we have no evidence that *Xenopus* carries such infectious viruses or that XtERV-S can produce virus. These frogs are all African, but the two *Xenopus* species have a limited shared geographical distribution (Fig. 5). While the distribution of the African bullfrog largely overlaps the territory of sub-Saharan Africa occupied by *X. laevis*, their ecological niches differ as *Xenopus* is fully aquatic whereas the bullfrog resides largely in dry savannas and shrub land; both species, however, reproduce in aquatic settings suggesting the possibility of trans-species transmission.

RV family relationships are determined by sequence identities and by the presence of conserved functional motifs that can be genus specific in their presence/absence, sequence variations and position. These features identify XtERV-S *gag* and *pol* as class III. The class III ERVs most closely related to XtERV-S are largely degenerate human ERVs, mouse ERVs with intact *gag* and *pol* genes, and ERVs found in nonmammalian vertebrates. Class III ERVs most prominently include ERV-L, an *env*-less ancient proviral lineage that entered mammalian genomes more than 100 mya [76] as well as divergent and generally degenerate subtypes like ERV-S [77], which has associated *env* sequences, but notably differs from ERV-L and XtERV-L in *gag-pol*, particularly in the absence of an identifiable dUTPase.

Class III ERVs are most closely related to the infectious spumaRVs, but XtERV-S is not particularly FV-like [30, 78]. While the location of the predicted *env* splice sites excludes the PBS, as is also the case for FVs, the novel XtERV-S SU*env* lacks FV features like a putative gag-interacting WXXW, a second furin site and an internal promoter. Also, while XtERV-S, like FVs, contains an FV-like consensus Gag p3 cleavage site (VXXV) [79], the location of this site downstream of the Gag stop suggests that any possible ancestral link no longer has any functional significance.

While the XtERV-S SU*env* sequence is not closely related to any other RV, it has a gammaRV-like CWIC motif that can potentially establish a covalent bond with its gammaRW-like TM*env.* Gamma-like Envs can be subgrouped on the basis of a “stutter” found in the N-terminal heptad repeat [51]. This motif, present in XtERV-S, is shared by other class III ERVs, some alphaRVs, *env* ERVs in some spiny-rayed fish and some mammalian *envs* domesticated to serve as syncytins. Syncytins are Env-encoding ERVs independently co-opted from different orthoretroviruses for a convergent physiological role in the formation of the syncytial layers at the placental fetal-maternal interface. More than 11 syncytins are found in different mammalian lineages [4], and the heptad stutter cluster in the TM*env* tree includes some but not all of these syncytins, a feature that is not related to taxa or to placenta type. This stutter has a presumed functional role in entry mechanisms involving endocytosis [80]. That this motif has important functionality and ancient origins is supported by its presence in the envelope genes of filoviruses, arenaviruses, influenza and coronaviruses [54–57].

The expression of retroviral LTRs in vertebrates depends on genetic and epigenetic factors including tissue type, ontogenic stages, age and sex [62, 81–84]. The LTR is transcriptionally active and Gag protein in transfected cells duplicates patterns reported for orthoRVs, but we have no evidence that XtERV-S can produce viral proteins or virus in vivo. XtERV-S expression is obviously under regulation as it is largely restricted to development stages 9 to 34. This expression coincides with transcriptional activation of the zygotic genome through the early tailbud stage suggesting these transcripts may have possible role in development. Many other ERVs and ERV-derived genes are expressed during embryogenesis (or in epididymis) including ERV-L [85–87] and while some of this expression has been co-opted to serve host regulatory functions, as for HERV-H [88], the timing of this expression may also represent a strategy to maximize or regulate proliferation in undifferentiated cells in early development to ensure preservation of ERV lineages within the host genome [89]. Further studies focused on these early developmental stages should clarify the extent of XtERV-S expression and uncover possible roles in development.

The different classes and families of ERVs are derived from independent genome invasion events followed by their differential amplification. ERV characterization has long focused on the many invasions that occurred after the divergence of mammalian orders. Most ERV families that have retained function are lineage-specific although important functional motifs have ancient roots and are found in dead ERVs. Here we described a set of ERVs that have ancient members along with recent acquisitions that retain some functionality. Elucidating the ancient origins of Retroviridae benefits from the increasing attention directed to nonmammalian vertebrates.

## Conclusions

We have identified a recently acquired intact ERV in *X. tropicalis*. Characterization of XtERV-S based on phylogenetics and the presence or absence of functional motifs that can be retrovirus or virus subtype specific shows that the *gag* and *pol* genes of XtERV-S are representative of the largely *env*-less class III ERVs. This provirus, however, carries a class I *env* gene with a novel surface subunit and a transmembrane subunit. XtERV-S expression is developmentally regulated with transcripts that are expressed between the mid-blastula maternal—zygotic transition and the tailbud stage. Additional much older defective copies are found in *X. tropicalis* as well as other African frog taxa indicating that this virus subtype has been circulating in these species for at least 36 million years, and may be representative of a yet to be discovered infectious retrovirus. Exploring XtERV-S expression and replication in X. tropicalis and also in vitro cell culture provides us an opportunity to understand the biology of ancient ERV-L and related family of endogenous retroviruses.

## Methods

### Cloning of XtERV-S

The XtERV-S genomic sequence was amplified from the genomic DNA of 20 pooled stage 12 *X. tropicalis* embryos provided by Dr. Frank L. Conlon (University of North Carolina, Raleigh, NC). Primers listed in Table 1 were designed from the XtERV-S proviral sequence identified in *X. tropicalis* v9.1 scaffold 1181, GenBank NW_016684263.1. PCR was performed using TaKaRa LA as per the manufacturer’s instructions (Clontech/TaKaRa, Mountain View, CA) using the strategy indicated in Fig. 1. The viral genome was amplified in three fragments that were cloned separately into the Xho1/Not1 site of the pBluescript SK(+) vector (Agilent Biosciences, Santa Clara, CA, USA). These fragments were sequenced and then ligated to each other to generate the full length XTERV-S proviral clone. The GenBank Accession number for XtERV-S is MW779451.

The XTERV-S and Moloney mouse leukemia virus (MoMLV) proviral LTR sequence was PCR amplified from pNCA [90] using primers listed in Table 1, and the fragment was cloned between the KpnI-BglII and BglII-HindIII sites of the pGL3 basic luciferase reporter plasmid (Promega, Madison, WI). The XtERV-S *gag* gene was amplified using primers listed in Table 1 and cloned into the eGFP-C1 vector (Clontech/TaKaRa) to produce GFP-Gag.

### Homology modeling

The XtERV-S Pol sequence was submitted to the I-TASSER [47] program which identifies homologs based on a multiple threading approach—identifying templates from PDB, iterative structure assembly simulation, model selection and refinement, and structure-based function annotation.

### Cell culture and luciferase assay

293T cells were grown and maintained in DMEM (Lonza, Walkersville, MD) containing 10% fetal bovine serum and supplemented with penicillin–streptomycin and L-Glutamine. 293T cells were transfected separately with the luciferase reporter vector carrying XtERV-S LTR, the promoter less—pGL3 basic control vector, CMV luciferase (Promega) and MoMLV LTR luciferase. Transfections were performed using Lipofectamine 3000 (Thermo Fisher Scientific, Atlanta, GA) and repeated three or more times and normalized to β-galactosidase activity expressed from a cotransfected pCMV-β (Clontech/TaKaRa). Cells transfected with reporter vectors were lysed in luciferase reporter cell lysis buffer and assayed for luciferase and β-galoctosidase activity as described previously [91].

### Confocal imaging

293T cells were cultured on 25-mm coverslips and transfected with 200 ng of either pEGFP-C1 or pEGFP-XtERV-S Gag plasmid in 12 well cell culture plates. One day later, cells were fixed with 3.7% formaldehyde and permeabilized with PBS containing 0.1% Triton X-100. Nuclei were stained with 4,6-diamidino-2-phenylindole (DAPI, Thermo Fisher Scientific). Coverslips were mounted onto glass slides with ProLong antifade kit (Thermo Fisher Scientific) and examined with a Leica laser-scanning microscope.

### Sequence analysis and phylogenetic trees

NCBI Blastn was used to search for additional copies of XtERV-S in the genomes of *X. tropicalis*, *X. laevis* and the African bullfrog, *P. adspersus*. Sequence analysis was performed using Geneious Prime 2021.0.3 (https://www.geneious.com). XtERV-S Env hydrophobicity plots were drawn using DNASTAR Lasergene 17 (DNASTAR Inc., Madison, WI).

Three phylogenetic trees were constructed in MEGA-X [92] using the Neighbor-Joining method [93]. The three trees were based on the RT domain of *pol*, the MHR region of *gag* and a segment of TM*env*; these segments correspond to the following positions in XtERV-S: RT: 3311–3871, gag:1841–2260, TM: 7126–7590. RV sequences used for the trees are listed in Additional file 9: Table S2. The evolutionary distances were computed using the JTT matrix-based method [94]. The rate variation among sites was modeled with a gamma distribution (shape parameter = 1). All positions with less than 95% site coverage were eliminated so fewer than 5% alignment gaps, missing data, and ambiguous bases were allowed at any position.

### Mapping and quantitation of the RNAseq reads to XTERV-S proviral region

Publicly available RNA-seq datasets for adult tissues (Accession No. SRX191164-68, 5 runs (brain, liver, kidney, heart and skeletal muscle, 39 Gbases) and distinct developmental stages from (Accession No. SRA051954—40 runs compromising 92 Gbases [66] were downloaded using the fastq-dump utility of the NCBI SRA Toolkit. Reads were then aligned to the XtERV-S genome using Bowtie2 [95], and the output was converted into indexed BAM files with Samtools [96]. Finally, Bedtools [97] was used to count the reads aligned to each particular region of the XtERV-S genome. The reads were mapped to the proviral sequence between the regions 301–7916 nt positions.

## Supplementary Information

**Additional file 1: Figure S1. **Sequence similarity of the RTs of XtERV-S and ERV-L. The amino acid sequences of the RTs of XtERV-S and other families of RVs were aligned using MUSCLE. Asterisks indicate conserved amino acids. The RT catalytic domain YIDD is shaded in grey.

**Additional file 2: Figure S2. **Schematic diagram locates the XtERV-S Orf-x2 and *orf-x* genes in unrelated ERVs. Relative positions are shown for the *pol* encoded *Orf-x* from XtERV-S and from JSRV, DxERV and DrERV [42–44]. The lightly shaded XtERV-S *Orf-x2* box identifies a short ORF before the in-frame stop codon.

**Additional file 3: Figure S3. **19 deleted XtERV-S like sequences in the *X. tropicalis* genome.

**Additional file 4: Figure S4. **Dot-plot and alignments compare the XtERV-S and XlERV-S genomes. Arrows indicate the site of insertions in the XlERV-S proviral sequence. Dots in the alignment represent identities. Shaded portions represent different regions of the proviruses – LTR (grey), PBS (red), *gag* (yellow), *pol* (green) and *env* (blue).

**Additional file 5: Figure S5**. XtERV-S like sequences in the African bull frog (*P. adspersus*) genome.

**Additional file 6: Figure S6. **Alignment of RV MHR sequences including XtERV-S and mouse ERV-L. MHR is defined by three conserved residues and a fourth site occupied by a hydrophobic residue, all with conserved spacing as shown in the consensus sequence.

**Additional file 7: Figure S7. **Alignment of the TM regions of the ERV and RV envelopes. Some of TMenv genes in the Fig. 6C tree have a heptad repeat stutter marked by N-linked glycosylation site (shaded in grey). The ISD region is shown in red and the CX_6_CC motif is highlighted in green.

**Additional file 8: Table S1**. Genome positions and age of the XtERV-S like elements in *Xenopus tropicalis*.

**Additional file 9: Table S2. **Accession numbers for sequences used for phylogenetic analysis.

## Data Availability

The XtERV-S sequence is deposited in GenBank under accession number: MW779451. Other sequences used and analyzed in the current study are publicly available in GenBank, accession numbers are provided in this article and its Additional files.

## Abbreviations

ERV: Endogenous retrovirus
ORF: Open reading frame
RT: Reverse transcriptase
*env*: Envelope
*gag*: Group specific antigen
XTR: *Xenopus tropicalis* Chromosome
LTR: Long terminal repeat
TSD: Target site duplication
PPT: Polypurine tract
PBS: Primer binding site
MA: Matrix
CA: Capsid
NC: Nucleocapsid
MHR: Major homology region
*pro*: Protease
RNaseH: Ribonuclease H
IN: Integrase
FV: Foamy virus
TM: Transmembrane
SU: Surface subunit
ISD: Immunosuppressive domain
GFP: Green fluorescense protein
MBT: Mid-blastula transition
RV: Retrovirus
HERV: Human endogenous retrovirus
DAPI: 4,6-Diamidino-2-phenylindole
MSD: Membrane spanning domain
CT: Cytoplasmic tail
RPKM: Reads per kilo base per million
JSRV: Jaagsiekte sheep retrovirus
