# Supplementary material for: A novel class III endogenous retrovirus with a class I envelope gene in African frogs with an intact genome and developmentally regulated transcripts in Xenopus tropicalis
Source: Retrovirology. 2021 Jul 14;18:20. doi: 10.1186/s12977-021-00564-2 (PMC8278194; doi:10.1186/s12977-021-00564-2)
Supplement: Supplementary file 1 — Additional file 1: Figure S1. Sequence similarity of the RTs of XtERV-S and ERV-L. The amino acid sequences of the RTs of XtERV-S and other families of RVs were aligned using MUSCLE. Asterisks indicate conserved amino acids. The RT catalytic domain YIDD is shaded in grey. [file 12977_2021_564_MOESM1_ESM.pdf]

```
                         **  *    **** *  ** * *     ********** **    * ******   **  * *    * * *  **********     * **** *
                             10        20        30        40        50        60        70        80        90        100
                      ....|....|....|....|....|....|....|....|....|....|....|....|....|....|....|....|....|....|....|....|...
XtERV-S               QALLEVGVFRPA--VSPFNAPVFPVKKKD-----GSWRMTVDYRGLNKAAPPLAAAVPDI--VSIVEDIAQTAGDWHAVLDLANAFFSIPIAEESQDQFAFTW
African bullfrog ERV  ...M.A.....--..........R.P.-----..Y...I......V.........M--.TL..T....E.................D.K........
MuERV-L               IVNKDA..VV.T--T....S.IW..Q.T.-----.........K..QVVT.I......V--..LL.Q.NTSP.T.Y.AI.......V.VHKDH.K.I..S.
Guineapig ERV-L       IVNKDA..VV.T--T....S.IW..Q.T.-----.........K..QVVT.I......V--..LL.L.NTSP.T.Y.AI..........HKDH.K....S.

Turtle_dove ERV       TVHESQ..ISKT--R....S.IW..R.SS-----DE..L........EVT...S.....M--LELQFELESK.AK.Y.TI.I........V.A.CRP.....
Ruff ERV              TVHEIQ..ISKT--H....S.IW..R.SN-----.E..L........EVT...S.....M--LELQYELESK.AK.Y.TI.I........L.A.CRP.....
Chicken ERV           .E.ER..II...--H..Y.S.IW..R.P.-----.T.......E...VT..IH....N.--A.LMDTLSREIETY.C.............K.........
Chimp ERV-S           VTSK..QIVHGT--H..Y.FL.W..R.P.-----.T-Q.....WK...VT...H....P.--MDLMDRLTMEL.EY.Y.V........D..P...ER.....

BFV                   ---.RQ..L.QQ--N.EM.T..Y..P.A.-----.R...VL...EV..VT.LV.TQNCHS--A..LNTLYR--.PYKST.....G.WAH..KP.DYWIT....
AKV                   PVS.DQ.ILV.C--Q..W.T.LL....PGT----NDY.PVQ.L.EV..RVEDIHPT..NP--YNLLSGLPPS-HR.YT....KD...CLRLHPT..PL...E.
FeLV                  PIS.DQ.ILK.C--Q..W.T.LL....PGT----EDY.PVQ.L.EV..RVEDIHPT..NP--YNLLSTLPPS-HP.YT....KD...CLRLHS...LL...E.
GALV                  PVA.DL..LV.C--R..W.T.LL....PGT----NDY.PVQ.L.EI..RVQDIHPT..NP--YNLLSSLPPS-YT.YS....KD...CLRLHPN..PL...E.
Syn-Opo1              PVIKTA.FLI.C--H..W.I.LL.XXXXWC----KDY.PVQ.L.EV.IWVEDIHPT..NPSIYTLLSTLTPH-LG.YTT...KD...TV.L.LL..PL...E.
Syn-Rum1L             LVRKQA.ILVEC--QTAW.T.IL....EGG----QDY.PVQ.L.LVSQ.TVT.HLS..KP--YTLLSLLPPK-TRIYTC...TE..SR.RL.PA..PI...E.
Syn1                  PYQKAQ.LVK.C--S..C.T.ILG.R.PN-----.Q..LVQ.L.II.E.VF..YP..SSP--YTLLSL.PEE-AE.FT....KD...C..VRPD..FL...ED
XEN1                  ---..N.AIKRQ--S..C.T.L....E.GKAGEPDKY.LVQ.L.AV.E.TVMETPL.SNP--HT.LSG.PPS-ATHFRAV..T...Y...LR.DC.YL....H
HTLV-1                ---..A.HIE.Y--TG.G.N.......AN-----.T..FIH.L.AT.SLTID.SSSS.GP---PDLSSLPT.-LAHLQTI..KD...Q..LPKQF.PY....V
BLV                   ---..A.YIS.W--DG.G.N.....R.PN-----.A..FVH.L.AT.ALTK.IP.LS.GP---PDLTA.PTH-PPHIIC...KD...Q..VEDRFRFYLS..L
HIV-1                 ----.-.KISKIGPEN.Y.T...AI....S----TK..KL..F.E...RTQDFWEVQLG.---PHTAGLKK--KKSVT...VGD.Y..V.LD.SFRKYT...I
EIAV                  ----.-.KISE.SDNN.Y.S.I.VI..RS-----.K..LLQ.L.E...TVQVGTEISRGL---PHPGGLIK--CKHMT...IGD.Y.T..LDP.FRPYT...I
RSV                   PVW.QL.HIE.S--L.CW.T...VIR.AS-----..Y.LLH.L.AV.AKLV.FG.VQQGA---PVLSALPR--.WPLM....KDC.....L..QDREA....L
MMTV                  ---.QL.HLEES--N..W.T...VI...S-----.K..LLQ.L.AV.ATMHDMG.LQ.GL---PSPVAVPK--.WEIIII..QDC..N.KLHP.DCKR...SV
```

```
                          * *** ** ****    ** *** ** ***   *         ***** *  *  **  *    *   ** *  *** *** * **** *** ***  *
                             110       120       130       140       150       160       170       180       190       200
                      .|....|....|....|....|....|....|....|....|....|....|....|....|....|....|....|....|....|....|....|....
XtERV-S               -------EGKQYTLTVVPQGY--MHSPTLCHGLVARDLAMLPN--MDCKFYHYIDDVMISGS-SEEQVRKDLQTVVTYMQKRGWAINPEK-IQGPATSVRFLGMIW
African bullfrog ERV  -------D.R.-AP..L....--L....I....I....EKI.P--IS.QM.....N....T-TQDE.Q....I.MD..KE.V....LS.-V....Q..KI.....
MuERV-L               -------Q.Q...F..L..V.--IN..A...N..R...DR.DLP-QSITLV.....I.LV.P-..QE.ATT.DSL..H.RI...E...T.-....S...K...VQ.
Guineapig ERV-L       -------Q.Q...F..L....--IN..A...N..Q...DRFSLP-Q.ITLV.........LT.P-..QE.ATA.DSL.RHLRA...E...T.-....S-T.K...VQ.

Turtle_dove ERV       -------R.I...WNRL...W--K....I....IQTT.EQGKAP----EHLQ....IIVW.D-TAKE.FEKGERIIQILLDA.F..KRS.-VK...REIQ...IK.
Ruff ERV              -------R.V...WNRL...W--K....I....IQTA.EKGEAP----EHLQ....II.W.N-TA.E.FEKGHKIIQILL.A.F..RRG.-VK...QEIQ...VK.
Chicken ERV           -------..R.W.FQ.L....--V....F..N...S...NWNKP-STV.MF.....L.LTSD-.I.ALE.TVPSLI..L.EK......Q.-V...GL..K...VV.
Chimp ERV-S           ----------EQ.F.ML..-----A.HHLSWYCCHGFGC.EMS-KRVH.F.....I.LISD-.LADLEVVAPFWRQHLAAC...V.ES.-V...GL.TK...V..

BFV                   -------G..T.CW..L...F--LN..A.FTAD.VDI.KDI----PNVQV--.V...YV.SA-T.QEHLDI.E.IFNRLSTA.YIVSLK.-SKLAKET.E...FSI
AKV                   RDPG-MGISG.L.W.RL...F--KN....FDEALH....DFRIQHP.LILLQ.V..ILLAAT-..LDCQQGTRALLLTLGNL.YRASAK.-A.LCQKQ.KY..YLL
FeLV                  RDPE-IGLSG.L.W.RL...F--KN....FDEALHS...DFRVRYPALVLLQ.V..LLLAAA-TRTECLEGTKALLETLGNK.YRASAK.-A.ICLQE.TY..YSL
GALV                  KDPE-KGNTG.L.W.RL...F--KN....FDEALH....PFRALNPQVVLLQ.V..LLVAAP-TY.DCK.GT.KLLQELS.L.YRVSAK.-A.LCQRE.TY..YLL
Syn-Opo1              YDED-TQATVKIMW.RL...F--KN...IFREAL.A..RDFCIDHPQVTLLQ.V.NLLLTA.-.Q.LCESATLSLLITENEL.YKVSGK.-ASIAQQE.TY..YKL
Syn-Rum1L             EDPI-GGNKQ.L.W.HLS..F--KNT.NIFGEAL.S..EPFQPERYG.WLLQ.V...LLAAE-PWVECCEGTPGSS.CG.RQDPECRGK.EA.ICKEE..Y..FVL
Syn1                  P----LNPTS.L.W..L...F--RD..H.FGQAL.Q..SQF--SYL.TLVLQ.V..LILATH-..TLCHQAT.ELLNFLTTC.YKVSKP.-ARLCSQQIKYX.LKL
XEN1                  -------.RQ..VW..L...A--QN...HFSLALTSI.DSWISSHPEITLLQ.V..LLLCAP-DLPTCEASSTDLLSFLADQ.CKASK..-L.WCQ.T.V...QCI
HTLV-1                PQQCNYGP.TR.AWR.L...F--KN....FEMQL.HI.QPIRQAFPQ.TILQ.M..ILLASP-.HADLQLLSEATMASLISH.LPVSEN.-T.QTPGTIK...Q.I
BLV                   PSPGGLQPHRRFAWR.L...F--IN..A.FERALQEP.RQVSAAFSQSLLVS.M..ILYASP-T...RSQCY.ALAARLRDL.FQVAS..-TSQTPSP.P...QMV
HIV-1                 PSINNETP.VR.QYN.L...W--KG..SIFQSSMTKI.EPFRSQHP.IVI.Q.M..LYVGSDLEIG.H.AKIEELRAHLLSW.FITPDK.-H.KEP-PFLWM.YEL
EIAV                  PSINHQEPD.R.VWNCL...F--VL..YIYQKTLQEI.QPFRERYPEVQL.Q.M..LFVGSNG.KK.HKELIIELRAILLEE.FETPDD.-L.EVP-PYSW..YQL
RSV                   PSVNNQAPARRFQWK.L...M--TC...I.QLV.GQV.EP.RLKHPSLCML..M..LLLAA.-.HDGLEAAGEE.ISTLERA.FT.S.D.-V.REP-G.QY..YKL
MMTV                  PSPNFKRPYQRFQWK.L...M--KN.....QKF.DKAILTVRDKYQ.SYIV..M..ILLAHP-.RSI.DEI.TSMIQALN.H.LVVST..-..KYD-NLKY..THI
```
