# Supplementary figures and images for: A novel class III endogenous retrovirus with a class I envelope gene in African frogs with an intact genome and developmentally regulated transcripts in Xenopus tropicalis
Source: Retrovirology. 2021 Jul 14;18:20. doi: 10.1186/s12977-021-00564-2 (PMC8278194; doi:10.1186/s12977-021-00564-2)

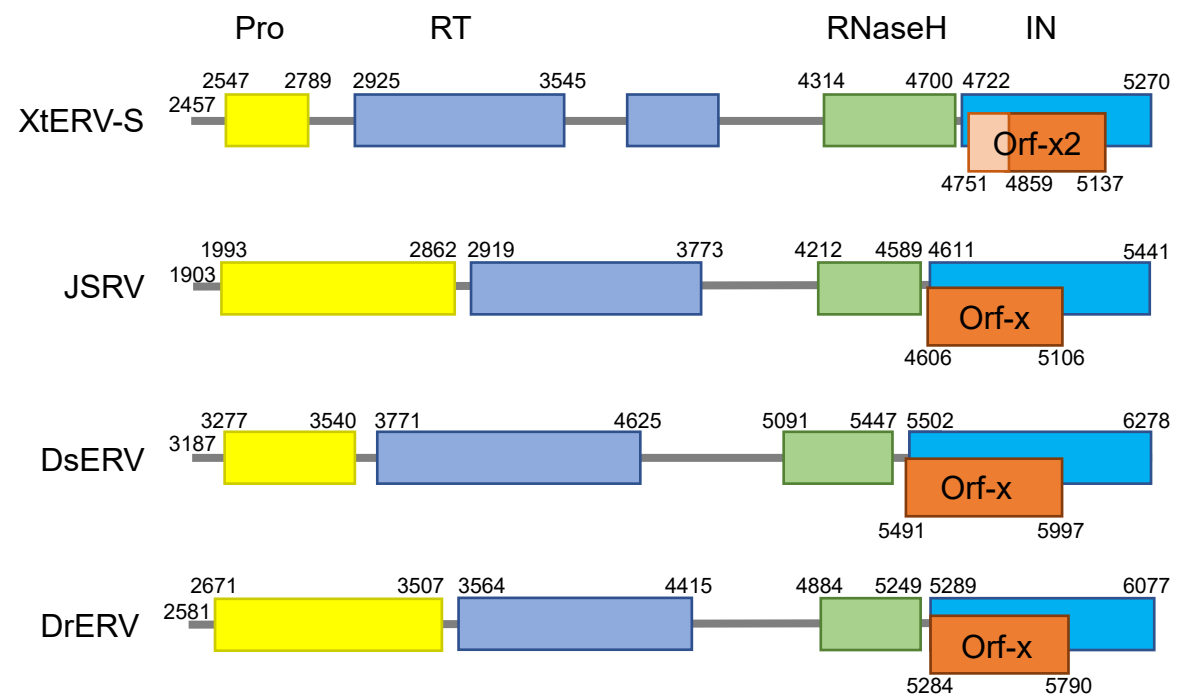

Supplement: Supplementary file 2 — Additional file 2: Figure S2. Schematic diagram locates the XtERV-S Orf-x2 and orf-x genes in unrelated ERVs. Relative positions are shown for the pol encoded Orf-x from XtERV-S and from JSRV, DxERV and DrERV [42–44]. The lightly shaded XtERV-S Orf-x2 box identifies a short ORF before the in-frame stop codon. [file 12977_2021_564_MOESM2_ESM.pdf]
