# Supplementary material for: A novel class III endogenous retrovirus with a class I envelope gene in African frogs with an intact genome and developmentally regulated transcripts in Xenopus tropicalis
Source: Retrovirology. 2021 Jul 14;18:20. doi: 10.1186/s12977-021-00564-2 (PMC8278194; doi:10.1186/s12977-021-00564-2)
Supplement: Supplementary file 3 — Additional file 3: Figure S3. 19 deleted XtERV-S like sequences in the X. tropicalis genome. [file 12977_2021_564_MOESM3_ESM.pdf]

>XtERV-S2(Xt-S2)NC_030680.2:c12439548-12431540 Xenopus tropicalis strain Nigerian chromosome 4, UCB_Xtro_10.0, whole genome shotgun sequence
TGTAGTGCAGTAGGTTTCTTTTACTATGATTTGTATGATTTACAATTTATACATGCTTTAATATGTGTAT
GTATATATATATATATATATGGTTTAGTTAGTAAGTTTAATCAAGCAATTCTAGAGATAAGGGGAGTCCA
GTGTCCTTGTACATATATTGTTGGGTAACAATACCTGCGTTTATTATGTAACAATAAACAATGACTATAG
ACTGTTTACCCAGCAAATACAACAAGCGGACGGCAGGCAGACAATGTCCAATGTATTTAAATAAGAACTT
GAGGTATAAAGTTTGGTATCTGATCAGACACAATGGCTGACCATTTGTTAAACAATATTGTTCAAGTCAA
TAGTTGGGCTTGGGGGTTTTTCCTAAGAACTTTTGGCATAAAAGAACGGCCTCTGCCTTGGGTCAGAAGC
TTCGCCTAGGACTCCTGAACGAGTGCCAGGATATTGGATCATCGCATGGTTATCGGGAACCTGAAGGTTT
TGCACCAAAGGTTGAGGGGCTCCCCGAGTTTGCTGGATTAATGCTGAACTGGCTATCTTTGTAACCACAA
AACCGTACGAAGCTAAATAAATGTTTTAAATTCTATTACTATGTGTGTGTGTAAGTTATTGCTCATTTAA
TAGTTTTTCATCAGAAGGTTTAATCATTGATCCTGTTAATATAAATAATATTGATAAAGGTTAACCCCCT
TTATTACATTTGGCGTAGTCGGCAGGATTAACCCTTTTGGTGCTGTTAAATGCTGCAATAGCTTGTGCAG
GAATTGTAGAATTTATGTTTTAGTTTGTATGGTAGTGTAGAAGCAGTATTGTAGTGGCTTGTGCAGGGCA
TACATACTGTTTAACAGATAGCAATAATGTTCTGCTGGTTAAAAAACAAGGTTAGAGGGGTAGATGATCA
AACGAGCACCCTTCCCACGTGGGCTAGTTCCGGACCCTGGACCCACGTTGCTCGTGAGTTGGTTAAGTAT
CAGCAACCTTTTCAAGTGACGAACCCCGGTCACCAGTTGCTGAATTTGGATTTGTCTCGGTATTCCAAAA
AAGAGCAGAAAGCTGCAGCACAAGTGGGTTGGTTGTGTTTTCAGGCGCTCATGGAGGAGACAATCAAAAG
GGAGTCAGTTGAGCTTGAACTGCAGGCAGTTAAAGATCAACTGGTTGTGTTAAGAGAATGTTTTCGCTCC
TCAGAAAAGTATCGTGAGGAGATGCAAACAGAGTTTTCAAATTATGTAGTAAAAACAGTAAATAAACAGC
ACAGAAAGAAAGGGAGGAAATCACCAGGAAATGGTGCAAAAGTTAAAGTAAGGGCTCTTGTAAATAAAGG
AGACTGGGGGGAGTTAGCCCAATTGTCAAGCGCAGACTCAGATGAAGAGATTGAGATAGGAGATGATATA
GAAGAAGAAGAATTAAGACCAATTGTAACTACCAAACAAAGGAGAGAAGTCACACAAAGAGCAGGCACTG
ATAATAGAGATCAGGATTATAGAGGAAGTGCAGAAGCAGCAGAACAGCAATATACAGAGGAAACTCGGTC
ATATACTGCAGGAGAATTAACAGAATTGGGTCAGAAATTTGCCCAGAAAGGTGGTGAAACTATTCTAGAA
TGGGTTTTGAGAGTGTGGGATTTGGGTGGGGATGGTATCATGCTCACTAGCAGGGAGGCAATAGGACTGG
GGAGTATTGCAAAAGACCCAAGGGTACGGCTTTATCTCAGGAGAGCCCGTGATACTACTGTGGCTTTTTC
CCTGTTACATTTGATCAGGGATTCAGTCCTACAAGTATATGATACCCCCACTGAAATGATGGATTCTACC
TCATGGCATACTATAGGGGAGGGGATACAGAGGTTAAGGGAGTATGGATGTGTGAGTGGTTTGATTGCTG
ATCCATTAGCTTCAGCACCCCCAAATAAACCTACTGTAAAAGAGACATGGTCAGGACCAGACATGGCTCC
CTTCACTGCACACATGAAAAATAAGCTGCTAGCAGGATCTCCCACCCATTTGAAAGCTCCATTGTTCACA
ATGTTAAGTGCTTTAGATGCCAAGCAGGGTGTAATACATGAAGCTGCAACATTGCTGGGACAGTTGGGAG
AGTTAGAACGTATAGGGGAAAAAGTATCTAGACACCGTGTAATTGCAGAAAGGGGTACAGAAAGATTAAA
AATTAGTCGGAGACAGTTGTTTTTTGATTTACTTCAAAGTGGAGTACCAAAGCCTGAAATTGATGGTATG
CCCACAGTTGATTTGTTGAAACTCTGGAAACAACTAAAACACAAGGGGAGGCTGAAATCAACCCAACCAA
AAAAGGTGGCAACCAGTGATATAGAGGATGTCCCTGTTACTCCTTCAGCTCCTCCTAATAAAGGGAAATT
TAACCCATACAGGGAAGTGGCTGAGGAATTGGCAAGACATAAGGAGAGGAGTTGGGAGGTGCCCAGTCCT
ATAGATTGACAGGATGGCAGATCCTCTGTGCCACTGGTAAAAATTAACACAATGAAACCAGGGGATCAAC
GCCCATATATATCAGTGGCAATATATTGGCAGGGACAGTCAAAACCACAAACGGTTACTGCACTTCTAGA
TACAGGGGCTGAAGTAACTTTGTTTCATGGAAATCAAAAACGACAGAGTGCAGGAATAGTAGCTATAGAG
GGATTGGGAGGTCAACATACACTGGCTCACAAGGTTCACTGCAGATTACAGATTGGAAAGGGTCCCATTT
TTAAAGCACGTGTGTTAATATCTGAGCTACCTGATAACATACTAGGAATGGATATTTTAAGGGGCCAGAC
TATCCATACTGATGCAGGCATGTATGTATTTGGTACCTCAGTTCGTTCTTTTTCTGTTTCTGCAGTTAAA
CCAATTTTAAGGGGACATGCCAAGTGGACACCAGTGTACATCCCTCCGCCCAAGCACCCTGTCAATCTCA
AGCAGTATCGCATACCCGGAGGCCACAAAGAAATTACTGAAACAATTCAAGCTTTGCTAGAGGTTGGAGT
ATTTCGGCCAGCAGTAAGTCCATTTAATGCTCCAGTAAAGAAAAAGGATGGAAGCTGGAGAATGACTGTG
GACTATCGTGGGTTAAACAAAGCAGCTCCACCTCTTGCAGCTGCTGTTCCAGATATAGTATCTATTGTGG
AGGACATTGCACAAACTGCTGGAGATTGGCATGCAGTATTGGATTTAGCAAATGCTTTCTTTTCCATTCC
TATTGCTGAGGAATCTCAAGATCAGTTTGCATTCACCTGGGAGGGAAAACAATACACATTAACTGTAGTA
CCACAGGGGTACATGCATTCTCCCACATTATGTCATGGATTGGTGGCTAGGGATCTGGCCATGCTGCCTA
ACATGGACTGTAAGTTTTATCACTACATTGATGATGTTATGATCTCAGGCAGCTCAGAGGAACAAGTGAG
AAAAGACTTACAAACAGTGGTGACATATATGCAGAAAAGAGGGTGGGCTATAAACCCAGAAAAAATCCAA
GGACCAGCAACTAGTGTCAGATTTCTTGGAATGATCTGGGCTGGACCAGTCAAATCTATCCCACAGCCTG
TGTTGGATTCAATTGCTGCCCTTAAACCACCAAAAAATGTGAAAGAAGCTCAGAGTTTGTTGGGTCTGCT
TGGATTTTGGAGGCCTTTTATTCCACACCTGGGCCTCATTCTCAGACCTATCTACAATATAACTAGGAAA
AAGACTGAGTTTACCTGGGGGTCTGAGCAGCAATTGGCACTTGACACAGCCAAAGAAACTGTAAGAACCC
ATCATTCACTAGGTCCTATACATCCAGATAAGCCATTTTTCTTAGATGTAGCAGTAACAGACCATGGAAT

GTCTTGGGGACTGTGGCAAAAAGGGCCTAATCCCAGGGATCGAAAAATACCTTTGGGTTTTTGGTCTAAG
CAATTTTCAACTGCTCAGAAAAAAATACAGCCCATTAGAGAAGCAATTGTTAGCTGCTTATACTGCCTTAC
AACATGTTGAGCCAACTACAAAGCAGCAATCTGTGACAGTAAGGACTGATTTACCTATTGCTGGATGGGT
CAGACAAGAAGGGCTACAATCCCGCACAGGTGTGGCCCAGAAACAGACTTTGCAGAAATGGAAATGGTAT
CTGAGTGAAAGAGGGGGAATATCTTCCCAAGACCCTAGTCAGCTATCGCAACAAATTGCTGGTTTTGTTA
ACTTTGAATCTAATGTAAATCAATTGCCCCCACTGCATGCAGAGGAGTCACTATTCAAGGAAGCCCCACC
ATGGATAGAGTTATCAGCAGAAGATAAACTACGAGCATGGTTTACAGATGGCTCAGCTAAAGTGACTTAC
AAGGGGCGGATATGGACTGCAGCAGCTTTTCAGCCTAGTTCAGAGACTATAATTCATCAGACGGGAGAAG
GAGGTTCTAGTCAATATGCTGAACTACAAGCTGTTTATATGGTTGTCCAAGAGACCTCAGGCGACTTGAT
TATCTATACAGATAGCTGGGCTGTATTTAAAGGTCTCACAACATGGCTTTGTATTTGGAAAAAAGATAAC
TGGCAAGTAAATGGAAAAGAGTTATGGGGCGGGCCTCAAGTATGGGATTTCTTATGGTGTCAAGGAAAAA
AGAGATGTATACAAGTAGGCCATGTTAATGCTCACACTGGAAATCTGAACAATGAAATAGTAGATGGTCT
GGCACAAGTCTCTACTGCACAACAAGAGGAAACTACCTTGGAAGTGTTGGGCAAATGGGCCCACAGTCAA
ACTGGGCACAAAGGAGTTCAAGGTACCTGGCAGTGGGCACAGCAGAGGGGAATACCTCTAACACAAATCC
AAGTGAAGGACATAATTGCTAAATGTCCTGTATGTCAGGAAGCCAAGAAGTGGCCACCTTTGACCCCATT
GCCTGGAAAAATTCATCGAGGACAAAAGCCAGGACAAGTCTGGCAAGTGGATTATATTGGACCCTTGCCT
GGAGGAAGGGGTGGTTTAAAATATTGTGCGACTGCTGTGGACACATACAGTGGACTAGTACAAGTATTTC
CAACAAAAAGTGCAGATCAAAAAACAACTCTAAGATTGATGCAACTGCTAATACAACATTATGGAATGCC
TCAAGAAGTACAGTCAGATAATGGCACACATTTTACTGGACAGACCGTAAAGCAATGGGCGGAGGATAAT
GGTGTATACTGGGTGTTCCACATTCCGTATTATCCTCAAGGGGCAGCGTTGATTGAGAGAATGAATGGTC
TCTTAAAGGAACAAATGCGCAAACTTACACCTACTCACACACTACGGGGATGGGATAAGGTGTTACAAGA
GGCAGTATATTTATTAAATAATAGATCAGTTGGACATTTCACTCCTATTCAAAGAATGCTGGGAGAAAGT
GGGGAGAATAGTTCAGATTGGGTAGTAACAGTTACTACAAAAGGTTCAACAGTCCCTTTATCTCAGTCTA
TTCCTGTATTTATACACAAAGATTTAGTTGTAGGAGGTGAAGAAGAACATACTATGTTACAAATTACATC
CATATCAGTACCAACAGGAATTTTAGACATAGATCCAACATGTGACTTGCAATTCATCCCAGATTTTGAT
TTAACCACTCAGTGTGACTGGGATGTTGACGTTAGAAAAGATGAATTGGGGGAATATTGTGTCACTATTG
TTCCTTTTGGTCGGGCACAGTTTAAGAAAGGACAAAAAATAGGACAAATAGTGATTTTACCAAAACGTTT
GCAAGTGAAGGGAAATATTTATTCCACACAGTTAGGAACAAAGGTATGGATAGCTCCAAGTGTAACAGAC
AGGGGTAGAAAAACAGGTAGAAAAGGAGAAATTGTGGCCTGGGGTCCTGGGTCAACAGCATTAGTTTTAA
TAGATGCTGAAGATAAGCCTGTTTATGTTCCTCTTCACAGATTATTACCACTACCATGAGGAGTTTGCCT
TGTCTACTGATGCTCGCGATATGTCTGATGAAAGTGACTGCGGAAAGGAATTTTTATCATGAAACGCTGA
AAGCTACTGCTGCAGTATTTAATGTAACAAACTGTTGGATATGTGGCAAAATCCCACATGCGACTGAAGA
AGGAATTCCACTTTATGGACTGCCATTTAATATGAGTTGGATAAACAGAACCGACCCTGAGTGGAACTTT
TTTTTCAATATGACTACAAAACAATGTGCTATTGCTAGATATGCAGGTACAGAAAAAGAGCAACAGTTGA
AGTTAACCCGGAAATCAACAGGGATTTTGTGTGTCCAGAAAAATCAAACCACTGAGACTGTGTGGTTAGG
AAAAAGTCAATGTGACTATGTTGTCAACACAGTTAACAACACACATGTAATGGTGACAGGAAAAGGGGAA
ATGTTTTATATGTTTTATGTTAAGCAATGTTCATTTGATTTTAGAGTTACTACAAATGATACGCATATTT
GCGAAAGATCAAGTACAAGCTCTCTGGTATGCTGTAAAATCTTACCATCTAATAGTACCAGGGTAGGAAT
ATACTGGCAATATCAGTTATATCTAGCAACTTGTTCCCCATTGCCCAATCAGTTGTACTTTATTTGTGGG
AATAATGTTTATAAGTGGCTACCATATAATTGGGCAGGAAATTGTTATATAGGAAATATAGCACCAAAAT
TTAGAGTATTATTTGAGAACCCAAAAGGGCATGTGAGAAATTGGAAACGGCATTTTGCCTCTTCTCATGT
TAATGAATTACCCGAAGAGTGGGATACTTCAGAAGAAAACAGATTTGTTATGATTTTAATACCCCATTAT
GGAGTAGCAAAAGCTGTGCAAATGATGAGAAGAATGTCTAGAATAATTGAACATGCTTTAAACAACACAT
TAGATGGCTTGACTAGTCTTACCGAGGAAGTCAGACAAATGAGGTTAGTGGTTCTGCAAAATAGAGCATC
GCTAGATTATATCCTAGCTTCAAAGGGAGGTGTATGTGCTCTGATTGGAGATGAATGTTGCACATATATC
TCAGATAAAAGTCTGGAAGTAGAGGATGACATCTCTGAAGCTCGAAAGTCAGTAGCCAAATTACATGCCC
AAAATACTGGTAGTTGGTTATTTAGTTGGTTGGGGCAATGGGGACAACAATTGTTTATGTATATAACATC
ATTTATTATATTCATTCTAGGAATTTTTCTTGTGATAAAAATAATCAGTGTCTGTATCAGTAAGTATACT
ACATCTGCAATAGAATCATTACCTATACGCAAAACTGGAATAATATATTAAATATTTCTCAATCAGTATT
TTTGTAAATCAAAGGGTGGAATGTAGTGCAGTAGGTTTCTTTTACTATGATTTGTATGATTTACAATTTA
TACATGCTTTAATATGTGTATGTATATATATATATATATATGGTTTAGTTAGTAAGTTTAATCAAGCAAT
TCTAGAGATAAGGGGAGTCCAGTGTCCTTGTACATATATTGTTGGGTAACAATACCTGCGTTTATTATGT
AACAATAAACAATGACTATAGACTGTTTACCCAGCAAATACAACAAGCGGACGGCAGGCAGACAATGTCC
AATGTATTTAAATAAGAACTTGAGGTATAAAGTTTGGTATCTGATCAGACACAATGGCTGACCATTTGTT
AAACAATATTGTTCAAGTCAATAGTTGGGCTTGGGGGTTTTTCCTAAGAACTTTTGGCATAAAAGAACGG
CCTCTGCCTTGGGTCAGAAGCTTCGCCTAGGACTCCTGAACGAGTGCCAGGATATTGGATCATCGCATGG
TTATCGGGAACCTGAAGGTTTTGCACCAAAGGTTGAGGGGCTCCCCGAGTTTGCTGGATTAATGCTGAAC

TGGCTATCTTTGTAACCACAAAACCGTACGAAGCTAAGTAAATGTTTTAAATTCTATTACTATGTGTGTG
TGTAAGTTATTGCTCATTTAATAGTTTTTCATCAGAAGGTTTAATCATTGATCCTGTTAATATAAATAAT
ATTGATAAAGGTTAACCCCCTTTATTACA

>XtERV-S3(Xt-S3)NC_030680.2:c11872885-11864925 Xenopus tropicalis strain Nigerian chromosome 4, UCB_Xtro_10.0, whole genome shotgun sequence
TGTAGTGCAGTAGGTTTCTTTTACTATGATTTGTATGATTTACAATTTATACATGCTTTAAATATGTGTA
TGTATATATATATATATATATGGTTTAGTTAGTAAGTTTAATCAAGCAATTCTAGAGATAAGGGGAGTCC
AGTGTCCTTGTACATATATTGTTGGGTAACAATACCTGCGTTTATTATGTAACAATAAACAATGACTATA
GACTGTTTACCCAGCAAATACAACAAGCGGACGGCAGGCAGACAATGTCCAATGTATTTAAATAAGAACT
TGAGGTATAAAGTTTGGTATCTGATCAGACACAATGGCTGACCATTTGTTAAACAATATTGTTCAAGTCA
ATAGTTGGGCTTGGGGGTTTTTCCTAAGAACTTTTGGCATAAAAGAACGGCCTCTGCCTTGGGTCAGAAG
CTTCGCCTAGGACTCCTGAACGAGTGCCAGGTATTGGATCATCGCATGGTTATCGGGAACCTGAAGGTTT
TGCACCAAAGGTGAGGGGCTCCCCGAGTTTGCTGGATTAATGCTGAACTGGCTATCTTTGTAACCACAAA
ACCGTACGAAGCTAAATAAATGTTTTAAATTCTATTACTATGTGTGTGTGTAAGTTATTGCTCATTTAAT
AGTTTTTTCATCAGAAGGTTTAATCATTGATCCTGTTTAATATAAATAATATTGATAAAGGTTAACCCCC
TTTATTACATTTGGCGTAGTCGGCAGGATTAACCCTTTTGGTGCTGTTAAATGCTGCAATAGCTTGTGCA
GGAATTGTAGAATTTATGTTTAGTTTGTATGGTAGTGTAGAAGCAGTATTGTAGTGGCTTGTGCAGGGCA
TACATACTGTTTAACAGATAGCAATAATGTTCTGCTGGTTAAAAAACAAGGTTAGAGGGGGTAGATGATC
AAACGAGCACCCTTCCCACGTGGGCTAGTTCCGGACCCTGGACCCACGTTGCTCGTGAGTTGGTTAAGTA
TCAGCAACCTTTTCAAGTGACGAACCCCGGTCACCAGTTGCTGAATTTGGATTTGTCTCGGTTTCCAAAA
AAGAGCAGAAAGCTGCAGCACAAGTGGGTTGGTTGTGTTTTCAGGCGCTCATGGAGGAGACAATCAAAAG
GGAGTCAGTTGAGCTTGAACTGCAGGCAGTTAAAGATCAACTGGTTGTGTTAAGAGAATGTTTTCGCTCC
TCAGAAAAGTATCGTGAGGAGATGCAAACAGAGTTTTCAAATTATGTAGTAAAAACAGTAAATAAACAGC
ACAGAAAGAAAGGGAGGAAATCACCAGGAAATGGTGCAAAAGTTAAAGTAAGGGCTCTTGTAAATAAAGG
AGACTGGGGGAGTTAGCCCAATTGTCAAGCGCAGACTCAGATGAAGAGATTGAGATAGGAGATGATATAG
AAGAAGAAGAATTAAGACCAATTGTAACTACCAAACAAAGGAGAGAAGTCACACAAAGAGCAGGCACTGA
TAATAGAGATCAGGATTATAGAGGAAGTGCAGAAGCAGCAGAACAGCAATATACAGAGGAAACTCGGTCA
TATACTGCAGGAGAATTAACAGAATTGGGTCAGAAATTTGCCCAGAAAGGTGGTGAAACTATTCTAGAAT
GGGTTTTGAGAGTGTGGGATTTGGGTGGGGATGGTATCATGCTCACTAGCAGGGAGGCAATAGGACTGGG
GAGTATGCAAAAGACCCAAGGGTACGGCTTTATCTCAGGAGAGCCCGTGATACTACTGTGGCTTTTTTTC
CCTGTTACATTTGATCAGGGATTCAGTCCTACAAGTATATGATACCCCCACTGAAATGATGGATTCTACC
TCATGGCATACTATAGGGAGGGGATACAGAGGTTAAGGGAGTATGGATGTGTGTGGTTTGATTGCTGATC
CATTAGCTTCAGCACCCCCAAATAAACCTACTGTAAAAGAGACATGGTCAGGACCAGACATGGCTCCCTT
CACTGCACACATGAAAAATAAGCTGCTAGCAGGATCTCCCACCCATTTGAAAGCTCCATTGTTCACAATG
TTAAGTGCTTTAGATGCCAAGCAGGGTGTAATACATGAAGCTGCAACATTGCTGGGACAGTTGGGAGAGT
TAGAACGTATAGGGGAAAAAGTATCTAGACACCGTGTAATTGCAGAAAGGGGTACAGAAAGATTAAAAAT
TAGTCGGAGACAGTTGTTTTTTGATTTACTTCAAAGTGGAGTACCAAAGCCTGAAATTGATGGTATGCCC
ACAGTTGATTTGTTGAAACTCTGGAAACAACTAAAACACAAGGGGGAGGCTGAAATCAACCCAACCAAAAA
AGGTGGCAACCAGTGATATAGAGGATGTCCCTGTTACTCCTTCAGCTCCTCCTAATAAAGGGAAATTTAA
CCCATACAGGGAAGTGGCTGAGGAATTGGCAAGACATAAGGAGAGGAGTTGGGAGGTGCCCAGTCCTATA
GATTGACAGGATGGCAGATCCTCTGTGCCACTGGTAAAAATTAACACAATGAAACCAGGGGATCAACGCC
CATATATATCAGTGGCAATATATTGGCAGGGACAGTCAAAACCACAAACGGTTACTGCACTTCTAGATAC
AGGGGCTGAAGTAACTTTGTTTCATGGAAATCAAAAACGACAGAGTGCAGGAATAGTAGCTATAGAGGGA
TTGGGAGGTCAACATACACTGGCTCACAAGGTTCACTGCAGATTACAGATTGGAAAGGGTCCCATTTTTA
AAGCACGTGTGTTAATATCTGAGCTACCTGATAACATACTAGGAATGGATATTTTAAGGGGCCAGACTAT
CCATACTGATGCAGGCATGTATGTATTTGGTACCTCAGTTCGTTCTTTTTCTGTTTCTGCAGTTAAACCA
ATTTTAAGGGGACATGCCAAGTGGACACCAGTGTACATCCCTCCGCCCAAGCACCCTGTCAATCTCAAGC
AGTATCGCATACCCGGAGGCCACAAAGAAATTACTGAAACAATTCAAGCTTTGCTAGAGGTTGGAGTATT
TCGGCCAGCAGTAAGTCCATTTAATGCTCCAGTAAAGAAAAAGGATGGAAGCTGGAGAATGACTGTGGAC
TATCGTGGGTTAAACAAAGCAGCTCCACCTCTTGCAGCTGCTGTTCCAGATATAGTATCTATTGTGGAGG
ACATTGCACAAACTGCTGGAGATTGGCATGCAGTATTGGATTTAGCAAATGCTTTCTTTTCCATTCCTAT
TGCTGAGGAATCTCAAGATCAGTTTGCATTCACCTGGGAGGGAAAACAATACACATTAACTGTAGTACCA
CAGGGGTACATGCATTCTCCCACATTATGTCATGGATTGGTGGCTAGGGATCTGGCCATGCTGCCTAACA
TGGACTGTAAGTTTTATCACTACATTGATGATGTTATGATCTCAGGCAGCTCAGAGGAACAAGTGAGAAA
AGACTTACAAACAGTGGTGACATATATGCAGAAAAGAGGGGTGGGCTATAAACCCAGAAAAAAATCCAAGG
ACCAGCAACTAGTGTCAGATTTCTTGGAATGATCTGGGCTGGACCAGTCAAATCTATCCCACAGCCTGTG

TTGGATTCAATTGCTGCCCTTAAACCACCAAAAAATGTGAAAGAAGCTCAGAGTTTGTTGGGTCTGCTTG
GATTTTGGAGGCCTTTTATTCCACACCTGGGCCTCATTCTCAGACCTATCTACAATATAACTAGGAAAAA
GACTGAGTTTACCTGGGGGTCTGAGCAGCAATTGGCACTTGACACAGCCAAAGAAACTGTAAGAACCCAT
CATTCACTAGGTCCTATACATCCAGATAAGCCATTTTTCTTAGATGTAGCAGTAACAGACCATGGAATGT
CTTGGGGACTGTGGCAAAAAGGGCCTAATCCCAGGGATCGAAAAATACCTTTGGGTTTTTGGTCTAAGCA
ATTTTCAACTGCTCAGAAAAAATACAGCCCATTAGAGAAGCAATTGTTAGCTGCTTATACTGCCTTACAA
CATGTTGAGCCAACTACAAAGCAGCAATCTGTGACAGTAAGGACTGATTTACCTATTGCTGGATGGGTCA
GACAAGAAGGGCTACAATCCCGCACAGGTGTGGCCCAGAAACAGACTTTGCAGAAATGGAAATGGTATCT
GAGTGAAAGAGGGGGAATATCTTCCCAAGACCCTAGTCAGCTATCGCAACAAATTGCTGGTTTTGTTAAC
TTTGAATCTAATGTAAATCAATTGCCCCCACTGCATGCAGAGGAGTCACTATTCAAGGAAGCCCCACCAT
GGATAGAGTTATCAGCAGAAGATAAACTACGAGCATGGTTTACAGATGGCTCAGCTAAAGTGACTTACAA
GGGGCGGATATGGACTGCAGCAGCTTTTCAGCCTAGTTCAGAGACTATAATTCATCAGACGGGAGAAGGA
GGTTCTAGTCAATATGCTGAACTACAAGCTGTTTATATGGTTGTCCAAGAGACCTCAGGCGACTTGATTA
TCTATACAGATAGCTGGGCAAAAGTGGCTAAATGGAAAAGAGTTATGGGGCGGGCCTCAAGTATGGGATT
TCTTATGGTGTCAAGGAAAAAAGAGATGTATACAAGTAGGCCATGTTAATGCTCACACTGGAAATCTGAA
CAATGAAATAGTAGATGGTCTGGCACAAGTCTCTACTGCACAACAAGAGGAAACTACCTTGGAAGTGTTG
GGCAAATGGGCCCACAGTCAAACTGGGCACAAAGGAGTTCAAGGTACCTGGCAGTGGGCACAGCAGAGGG
GAATACCTCTAACACAAATCCAAGTGAAGGACATAATTGCTAAATGTCCTGTATGTCAGGAAGCCAAGAA
GTGGCCACCTTTGACCCCATTGCCTGGAAAAATTCATCGAGGACAAAAGCCAGGACAAGTCTGGCAAGTG
GATTATATTGGACCCTTGCCTGGAGGAAGGGGTGGTTTAAAATATTGTGCGACTGCTGTGGACACATACA
GTGGACTAGTACAAGTATTTCCAAAAAAAAGTGCAGATCAAAAAACAACTCTAAGATTGATGCAACTGCT
AATACAACATTATGGAATGCCTCAAGAAGTACAGTCAGATAATGGCACACATTTTACTGGACAGACCGTA
AAGCAATGGGCGGAGGATAATGGTGTATACTGGGTGTTCCACATTCCGTATTATCCTCAAGGGGCAGCGT
TGATTGAGAGAATGAATGGTCTCTTAAAGGAACAAATGCGCAAACTTACACCTACTCACACACTACGGGG
ATGGGATAAGGTGTTACAAGAGGCAGTATATTTATTAAATAATAGATCAGTTGGACATTTCACTCCTATT
CAAAGAATGCTGGGAGAAAGTGGGGAGAATAGTTCAGATTGGGTAGTAACAGTTACTACAAAAGGTTCAA
CAGTCCCTTTATCTCAGTCTATTCCTGTATTTATACACAAAGATTTAGTTGTAGGAGGTGAAGAAGAACA
TACTATGTTACAAATTACATCCATATCAGTACCAACAGGAATTTTTAGACATAGATCCAACATGTGACTT
GCAATTCATCCCAGATTTTGATTTAACCACTCAGTGTGACTGGGATGTTGACGTTAGAAAAGATGAATTG
GGGGAATATTGTGTCACTATTGTTCCTTTTGGTCGGGCACAGTTTAAGAAAGGACAAAAATAGGACAAAT
AGTGATTTTACCAAAACGTTTTGCAAGTGAAGGGAAATATTTATTCCACACAGTTAGGAACAAAGGTATG
GATAGCTCCAAGTGTAACAGACAGGGGTAGAAAAACAGGTAGAAAAGGAGAAATTGTGGCCTGGGGTCCT
GGGTCAACAGCATTAGTTTTAATAGATGCTGAAGATAAGCCTGTTTATGTTCCTCTTCACAGATTATTAC
CACTACCATGAGGAGTTTGCCTTGTCTACTGATGCTCGCGATATGTCTGATGAAAGTGACTGCGGAAAGG
AATTTTTATCATGAAACGCTGAAAGCTACTGCTGCAGTATTTAATGTAACAAACTGTTGGATATGTGGCA
AAATCCCACATGCGACTGAAGAAGGAATTCCACTTTATGGACTGCCATTTAATATGAGTTGGATAAACAG
AACCGACCCTGAGTGGAACTTTTTTTTCAATATGACTACAAAACAATGTGCTATTGCTAGATATGCAGGT
ACAGAAAAAGAGCAACAGTTGAAGTTAACCCGGAAATCAACAGGGATTTTGTGTGTCCAGAAAAATCAAA
CCACTGAGACTGTGTGGTTAGGAAAAAGTCAATGTGACTATGTTGTCAACACAGTTAACAACACACATGT
AATGGTGACAGGAAAAGGGGAAATGTTTTATATGTTTTTATGTTAAGCAATGTTCATTTGATTTTAGAGT
TACTACAAATGATACGCATATTTGCGAAAGATCAAGTACAAGCTCTCTGGTATGCTGTAAAATCTTACCA
TCTAATAGTACCAGGGTAGGAATATACTGGCAATATCAGTTATATCTAGCAACTTGTTCCCCATTGCCCA
ATCAGTTGTACTTTATTTGTGGGAATAATGTTTATAAGTGGCTACCATATAATTGGGCAGGAAATTGTTA
TATAGGAAATATAGCACCAAAATTTAGAGTATTATTTGAGAACCCAAAAGGGCATGTGAGAAATTGGAAA
CGGCATTTTGCCTCTTCTCATGTTAATGAATTACCCGAAGAGTGGGATACTTCAGAAGAAAACAGATTTG
TTATGATTTTAATACCCCATTATGGAGTAGCAAAAGCTGTGCAAATGATGAGAAGAATGTCTAGAATAAT
TGAACATGCTTTAAACAACACATTAGATGGCTTGACTAGTCTTACCGAGGAAGTCAGACAAATGAGGTTA
GTGGTTCTGCAAAATAGAGCATCGCTAGATTATATCCTAGCTTCAAAGGGAGGTGTATGTGCTCTGATTG
GAGATGAATGTTGCACATATATCTCAGATAAAAGTCTGGAAGTAGAGGATGACATCTCTGAAGCTCGAAA
GTCAGTAGCCAAATTACATGCCCAAAATACTGGTAGTTGGTTATTTAGTTGGTTGGGGCAATGGGGACAA
CAATTGTTTATGTATATAACATCATTTATTATATTCATTCTAGGAATTTTTCTTGTGATAAAAATAATCA
GTGTCTGTATCAGTAAGTATACTACATCTGCAATAGAATCATTACCTATACGCAAAACTGGAATAATATA
TTAAATATTTCTCAATCAGTATTTTTGTAAATCAAAGGGTGGAATGTAGTGCAGTAGGTTTCTTTTACTA
TGATTTGTATGATTTACAATTTATACATGCTTTAATATGTGTATGTATATATATTATATATATGGTTTAG
TTAGTAAGTTTAATCAAGCAATTCTAGAGATAAGGGGAGTCCAGTGTCCTTGTACATATATTGTTGGGTA
ACAATACCTGCGTTTATTATGTAACAATAAACAATGACTATAGACTGTTTACCCAGCAAATACAACAAGC
GGACGGCAGGCAGACAATGTCCAATGTATTTAAATAAGAACTTGAGGTATAAAGTTTGGTATCTGATCAG

ACACAATGGCTGACCATTTGTTAAACAATATTGTTCAAGTCAATAGTTGGGCTTGGGGGTTTTTCCTAAG
AACTTTTGGCATAAAAGAACGGCCTCTGCCTTGGGTCAGAAGCTTCGCCTAGGACTCCTGAACGAGTGCC
AGGATATTGGATCATCGCATGGTTATCGGGAACCTGAAGGTTTTGCACCAAAGGTTGAGGGGCTCCCCGA
GTTTGCTGGATTAATGCTGAACTGGCTATCTTTGTAACCACAAAACCGTACGAAGCTAAGTAAATGTTTT
AAATTCTATTACTATGTGTGTGTGTAAGTTATTGCTCATTTAATAGTTTTTCATCAGAAGGTTTAATCAT
TGATCCTGTTAATATAAATAATATTGATAAAGGTTAACCCCCTTTATTACA

>Xt-S4_CM004449.2:75962155-75966160_ Xenopus tropicalis strain Nigerian chromosome 7, whole genome shotgun sequence
TGTAGCACGGTAGATTTTCTATTGATTTTATGATTTACAATATATACATCTGCTTTGACATATGAATCAAATC
TTAGGTTTGAGTCAAGTAAAAGCAGCAGATTTTGGGACAGCTATTGTCTAGTGTTCAAGGTACAATGTTTTTG
TTCTATGTATATTGCTGGGTAAACAGTAAACACAGGTATTGTTACCCAGCAAATATCCTTGGAATAAACAAAC
AGACAGCATATAGACAATACACTTTATGTACAACATATTTGAGCAACAAATAAGGCCTTCTAAGTCTAGTTAA
TAGGTTTTGTTCATGACAAAAGTTTGTACACAAGTCAGCACTTGGTGTTGGGGGTTTGCTAAGAACTCTACTA
TAAAAAGGAGCCTCCACCATGTGTCGGGAGCTTCGCCTAGGACTCCTGAATGAGTGCCGGGACATTTGGATCA
TCGCTTGGTTATCGGGAACCTGAAGGTCTTGCGCCAAAGGTTGTGGGGCTCCCCGATTGTGCTGAATTGTGCA
GAACTGGCTATACTTGTAACTAATCATACTAAGATAAGTAAATCTATTTAATTCTATTATTACTTGTGTGTGT
GTAAGTTATTGCTCACTAACAGTCTATCAGAAGGTTTAATCATTGATCCTGCATATTTATTAATATTTGTCAT
TAATGTCAATTAATACAAATTATTAATATTAATAAAGGTTCACCCCCTTTATTACATTTGGTGTAGTCGGCAG
GATTAACCCTTTTGATGCTGGGAAGTGCAGTAATAACTTGTATAGAAATTGTAGAATTTATAGTGCAGCTTTG
TATGGTAGTGTAGTGGCAGTATTGTAGTGGCTTGTGCAGGGCAAACATACTGTTGAAAGTTAGCAATAATGTT
CTGTTGGTTGAAAAACAAAGTTAGAGGGGTAGATGATCAAACGAGCAACCTTCCCACATGGGCTAGTTCCGGA
CCCTGGACCCATGTTGCTCGGGATGTGATAGGAGATCAGCAACTCTCTCAGGTGACTCAACCCCATCACCTGT
TGCTGAATTTGGATTTGTCTATGTGTTCCAAGGAGCTGAAGTCCTCAGCACAATTGGGTTGGTTGTGTTTTCA
GGCGCTCAGGGAGGAGACAGTCCAAAGGGAGGCAGTTGAGCTTGAGCTGCAGGCAGTTAAAGATCAACTAGCC
ACAATGAGAGAATGTTTGCTCTCCTCATTAGCACACAATGAGGAGTTGGAGATAGAATTTACAAAATATGTGG
TAAGAACTATGAGTAAAAAGCACCATAGAAAAGGGACAGAACAAGCAGAAAATGGTGTAAGAGTAAGAACTCT
TGTAAGTAAAAGAGACTAAGGGTGTGATTTATTTCTCAAGATAGAGAAACAAAATAAATGTTACATTGTGTTA
AATTGTACTGTATGTTAATGTCATATGTTTTTCTGTTTCAGGATTTAAGAATCTGATTATTTTGAGTTAATAT
GGTTTTTGATAACAGGCATCAAGAGTTGATGGATCCATTATTTTTGACACTTTTTGTTTTGTTTTTTAAGTGC
ACAAATTTGTGCGTAAAGGCCTTAAGACATGTCTGTTTTTGTTAAAAAAAAAAGTTTAACAATGAAACTTGAA
AGTTTTTAACACTTTTTGTTGTATGTTTCTGAGAATGTGTGGGCCCACATGCATGTTGTTATGGTTTGTCTTG
TCTTAATTGTGACAATGACTTAAATGTGACAGTGACTGAAAAATTCTTGACAAAGTAATACAAAATTAAGAAA
GTTGTTAAAGTTGACTAAACAGATGTTAAATGCTAAGAACTGATGTTGCCTTGATCTTTTGTTTTATAGATGT
TTATCAAGTGCATTAACAAATGGGTTAATAGCAAAACAATTGACAGCAAACAAGGATTTGGACATTGTTAATC
TTGAATGGACATCAGTACAAAGTATTGAAGAACCTGGAATTCAGTTGAATGGTGACTTGGAAACTTAAATGGA
CATTGGTTTTGGACTTTATAAATGAATACTTATGTTTTGTTCAACTGTTTCTACAGACTGAAATTAGCATTGC
ATACACTATTGTACTAATGGTAATAGGAATGTTACATGAACTGTAAAGTGCTAGTTAGTGGGTTTTAGAGCAC
AGGCAGCACAGCTGAACTGTATATCTCCTATGGTATATAATATTGTGCAGTGAACCTGCAGTATAGAGCAGGC
CCTGGCTGACAATCTGTAGATTTAGGCAAATGCCAGAGGTGCTGCTGTAAGATGCCATAGACAGTCACTATTT
ATTGGGCTGGTGGGGTCTGTTTGGGTCTCTGTGTATTTGAAATGCCAGGGTCTATTTTGAAATTAGTGTCAAC
AAAACTCTTTGATAAAACAGAAAGAGTGAAATTTATTATTCTGAGGGAGAAATTGTTATGTGAAACTAACAAT
GTCTGTAGACAAGCAATATGGTATAATTGTTGTAAGTGTGAATAGGGTAATGTCATTTTATGAGTTAGTACTA
TGATATCAATGGAGTTAGCTGAATGTTGGCTGTAAGAGAGTTTGGACAACTCTATATGATTGAAGTGAAAGCT
TTTTAAAGACTACAAGTTAATACACAATTCTGCTGAGGGACACAGTTGTTTATAAGAAGTCACTATTAGACTT
GATAGTATCATCCCAAGTGATTTGAATGCCCTGGGTGATTTGTGGCCTTATGGGTGGAATTTGTGGCTACTAG
TGATAGACTGATATTTTAGAAAAGCATTGGCAGAATGTGTATTGAGGTTAAAACTCTGCTGAAAGGAATTCAG
TTGTCATTTGGTCTATAAGAGGGGGGTAAGGTAGAAACAAAGTGCATGGCAGATACATAATATAGTAAACATA
TTGCTTATCAAAGAGGCACTGTTTAACTATGTGAGCTGTAAAGCCTGCAATGAAACCTGCCCTTGGCCTAATT
GATTTGAGCAGCTGATAGCCTATAATGAAAATGCAGAGCAAGAGGGATGCCTATACATGTTTGCCTCATGTTT
GCCCAAGATATGTATCTACAAGTAGACACAGATTTTCTCACTGGACAGAAATCATTATAGAGGTGTTTTATGA
CTTGGGATGTAGGTGAGATTTGTTTAATCTTTTGTTAAATGTAGTGTCCTTCAGTCAGAATTACAGTAATTAA
TTGTTTATTTGTTTAGGGTAATGGGGACAACCAGTGAATGCATATATTGCATTGAATGCAGCATTGTATGTTA
TCTGTATTAGAGGACTTACCAATTTGTATACTGTTGGTTATTATTATCAAATCAGTATATTTTGTAAATCAAG

GGGTGGAATGTAGCACGGTGGATTTTCTATTGATTTTATGATTTACAATATATACATCTGCTTTGACATATGA
ATCAAATCTTAGGTTTGAGTCAAGTAAAAGCAGCAGATTTTGGGACAGCTATTGTCTAGTGTTCAAGGTACAA
TGTTTTTGTTCTATGTATATTGCTGGGTAAACAGTAAACACAGGTATTGTTACCCAGCAAATATCCTTGGAAT
AAACAAACAGACAGCATATAGACAATACACTTTATGTACAACATATTTGAGCAACAAATAAGGCCTTCTAAGT
CTAGTTAATAGGTTTTGTTCATGACAAAAGTTTGTACACAAGTCAGCACTTGGTGTTGGGGGTTTGCTAAGAA
CTCTACTATAAAAAGGAGCCTCCACCATGTGTCGGGAGCTTCGCCTAGGACTCCTGAATGAGTGCCGGGACAT
TTGGATCATCGCTTGGTTATCGGGAACCTGAAGGTCTTGCGCCAAAGGTTGTGGGGCTCCCCGATTGTGCTGA
ATTGTGCAGAACTGGCTATACTTGTAACTAATCATACTAAGATAAGTAAATCTATTTAATTCTATTATTACTT
GTGTGTGTGTAAGTTATTGCTCACTAACAGTCTATCAGAAGGTTTAATCATTGATCCTGCATATTTATTAATA
TTTGTCATTAATGTCAATTAATACAAATTATTAATATTAATAAAGGTTCACCCCCTTTATTACA

>Xt-S5_CM004447.2:144579405-144575357_ Xenopus tropicalis strain Nigerian chromosome 5, whole genome shotgun sequence
TGTAGCACGGTGGATTTTATTTTGATTTTATAATGATTTACATTATATATCTGCTTTGACATATGAATCAAAT
CTTAGGTTTGAGTCAAGTAAAAGCAGCAGATTTTGGGACAGTTATTGTCCAGTGTTCAAGGTACAATGTCTTT
GTTCTATGTATATTGCTGGGTAAACAGTAAACACAGGTATTGTTAGCAAATATCCTTGGAATAAACAAACAGA
CAGCATATAGACAATACACTTTATGTACAACTTATTTGAGAAACAAATAAGGCTTTCTACAAAGTTAGTAAAC
TGTCTAGTTAATAGGTTTTGTTCATGACAAAAGTTTGTACACAAGTCAGCACTTGGTGTTGGGGGTTTTGCTA
AGAACTCTAGTATAAAAGGGAGCCTCCACCATATGTCAGTAGCTTCGCCTAGGACTCCTGAACGAGTGCCGGG
ACAATTGGATCATCGCTTGGTTATCGGGAACCTGAAGGTCTTGCGCCAAAGGTTGTGGGGCTCCCCGATTGTG
CTGAATTGTGCAGAACTGGCTATACTTGTAACTAATCATACTAAGATAAGTAAATCTATTTAATTCTATTATT
ACTTGTGTGTGTGTAAGTTATTGCTCACTAACAGTCTATCAGAAGGCTTAATCATTGATCCTGCATATTTATT
AATATTTGTCATTAATGTCAATAAATACAAATTATTAATATTAATAAAGGTTCACCCCCTTTATTACACTTGG
CGTAGTCGGCAGGATTAACCCTTTTGATGCTGGGAAGTGCAGTAATAACTTGTATAGAAATTGTAGAATTTAT
AGTGCAGCTTTGTATGGTAGTGTAGTGGCAGTATTGTAGTGGCTTGTGCAGGGCAAACATACTGTTGAAAGTT
AGCAATAATGTTCTGTTGGTTGAAAAACAAAGTTAGAGGGGTAGATGATCAAACGAGCAACCTTCCCACATGG
GCTAGTTCCGGACCCTGGACCCATGTTGCTCGGGATGTGATAGGAGATCAGCAACTCTCTCAGGTGACTCGCC
CCCATCACCTGTTGCTGAATTTGGATTTGTCTATGTGTTCCAAGGAGCAGAAGTCCTCAGCACAATTGGGTTG
GTTGTGTTTTCAGGCGCTCAGGGAGGAGACAGTCCAAAGGGAGGCAGTTGAGCTTGAGCTGCAGGCAGTTAAA
GATCAACTAGCCACAATGAGAGAATGTTTGCGCTCCTCATTAGCACACAATGAGGAGTTGGAGATAGAATTTA
CAAAATATGTGGTAAGAACTATGAGTAAAAAGCACCATAGAAAAGAGACAGAACAAGCAGAAAATGGTGTATT
AGTTAAAGTAAGAACTCTTGTAAGTAAAAGAGACTAAGGGAGTGATTTATTTCTCAAGATAGAGAAACAAAAT
AAATGTTACATTGTGTTTAATTGTACTGTATGTTAATGTCATATGTTTTCTGTTTCAGGATTTAAGAATCTGA
TTATTTTGAGTTAATATGGTTTTTAATAACAGGCATCAAGAGTTGATGGATCCATTATTTTTGACACTTTTTG
TTTTGTTTTTTTTAGTGCACAAATTTGTGCGTAAAGGCCTTAAGACATGTCTGTTTTTGTTAAAAAAAAAAGT
TTAACAATGAAACTTGAAAGTTTTTAACACTTTTTGTTGTATGTTTCTGAGAATGTGTGGGCCCACATGCATG
TTGTTATGGTTTGTCTTGTCTTAATTGTGACAATGACTTAAATGTGACAGTGACTGAAGAATTCTTGACAAAG
TAATACAAAATTAAGAAAGTTGTTAAAGTTGACAAAAACAGATCTTAAATGCTAAGAACTGATGTTGCCTTGA
TCTTTTGTTTTATAGATGTTTATCAAGTGCATTAACAAATGGGTTAATAGCAAAACAATGGACAGCAAACAAG
GATTTGGACATTGTTTATCTTCAATGGACATCAGTACAAAGTATTGAAGAACCTGGAATTCATTTGAATGGTG
ACCTGGAAACTTAAATGGACATTGGTTTTGGACTTTATAAATGAATATTTATGTTTTGTTCAACTGTTTCTAC
CGGCTGAAATTAGCATTGCATACACTATTGTACTAATGGTAATAGGAATGTTATGGCTTGTTACATGAACTGT
AAAGTGCTAGTTAGTGGGTTCTAGAGCACAGGCAGCACAGCTGAACTGTATATCTCTTATGGTATATGATATT
GTGCAGTGAACCTGCAGTATAGAGCAGGCCCTGGCTGACAATCTGTAGATTTAGGCAAATGCCAGAGGGGCTG
CTGTAAGATGCCATAGACAGTCACTATTTATTGGGCTGGTGGGGTCTGTTTGGGTCTCTGTGTATTTGAAATG
CCAGGGTCTATTTTGAAATTAGTGTCAACAAAACTCTTTGATAAAACAGAAAGAGTGAAATTTAATATTCTGA
GGGAGAAATTGTTATGTGAAACTAACAATGTCTGTAGACAAGCATTATGGTATAATTGTTGTAAGTGTGAATA
GGGTAATGTAATTTTATGAGTTAGTACTATGATATCAATGGAGTTAGCTGAATGTTGGCTGTAAGAGAGTTTG
GACAACTCTATATGATTGAAGTGAAAGCTTTTTAAAGACTACAAGTTAATACACAATTCTGCTGAGGGACACG
TTAGTTTACAAGAAGTCACTGTTAGACTTGATAGTATCATCCCAAGTGATTTGAATGCCCTGGGTGATTTGTG
GCCTTATGGGTGGAATTTGTGGCTACTGGTGATAGACATATTTTGGAAAAGCATTGGCAGAATGTGTACTGAG
GCTAAAACTCTGCTGAAAGGAATTCAGTTGCCATTTGGTCTATAAGAGGGGGGTTAGGTAGAAACAAAGTGCA
TGGCAGATACATAATATAGTAAACATATTGCTTATCAAAGAAGCACTGTTTAACTATGTGAGCTGTAAAGCCT
GCAATGAAACCTGCCCTTGGCCTAATTGTTTTGAGCAGCTGATAGTCTGTAATGAAAATGCAGAGCAAGAGGG

ATTCCTATACATGTTTGCCACATGATTGCCCATGATATGTATCTACAAGTAGACACAGATTTTCTCACTGGAC
AGAAATCATTATAGAGATGTTTTATGACTTGGGATGTAGGTGAGATTTGTTTAATCTTTTGTTAAATCTAGTG
TCCTTCAGTCAGTATTACAGTCATTAATTGTTTATTTGTTTATGGTAATGGGGACAACCAGTGAATGCATATA
TTGCATTGAATGCAGCATTGTATGTTATCTGTATTAGAGGACTTACCAATTTGTATACTGTTGGTTATTATTA
TCAAATCAGTATATTTTGTAAATCAAGGGGTGGAATGTAGCACGGTGGATTTTATTTTGATTTTATAATGATT
TACATTATATATCTGCTTTGACATATGAATCAAATCTTAGGTTTGAGTCAAGTAAAAGCAGCAGATTTTGGGA
CAGTTATTGTCCAGTGTTCAAGGTACAATGTCTTTGTTCTATGTATATTGCTGGGTAAACAGTAAACACAGGT
ATTGTTACCCAGCAAATATCCTTGGAATAAACAAACAGACAGCATATAGACAATACACTTTATGTACAACTTA
TTTGAGAAACAAATAAGGCTTTCTACAAAGTTAGTAAACTGTCTAGTTAATAGGTTTTGTTCATGACAAAAGT
TTGTACACAAGTCAGCACTTGGTGTTGGGGGTTTTGCTAAGAACTCTAGTATAAAAGGAAGCCTCCACCATGT
GTCAGTAGCTTCGCCTGGGACTCCTGAACGAGTGCCGGGACAATTGGATCATCGCTTGGTTATCGGGAACCTG
AAGGTCTTGCGCCAAAGGTTGTGGGGCTCCCCGATTGTGCTGAATTGTGCAGAACTGGCTATACTTGTAACTA
ATCATACTAAGATAAGTAAATCTATTTAATTCTATTATTACTTGTGTGTGTGTAAGTTATTGCTCACTAACAG
TCTATCAGAAGGCTTAATCATTGATCCTGCATATTTATTAATATTTGTCATTAATGTCAATTAATACAAATTA
TTAATATTAATAAAGGCTCACCCCCTTTATTACA

>Xt-S6_CM004450.2:74753455-74757497_ Xenopus tropicalis strain Nigerian chromosome 8, whole genome shotgun sequence
TGTAGCACGGTGGATTTTATTTTTATTTTATAATGATTTACATTATATATCTGCTTTGACATATGAATCAAAT
CTTAGGTTTGAGTCAAGTAAAACCAGCAGATTTTGGGACATCTATTGTCCAGTGTTCAAGGTACAATGTCTTT
GTTCTATGTATATTGCTGGGTAAACAGTAAACACAGGTATTGTTACCCAGCAAATATCCTTGGAATAAACAAA
CAGACAGCATATAGACAATACACTTTATGTACAACTTATTTGAGAAACAAATAAGGCTTTCTACAAAGTTAGT
AAACTGTCTAGTTAATAGGTTTTGTTCATGACAAAAGTTTGTACACAAGTTAGCACTTGGTGTTGGGGGTTTG
CTAAGAACTCTAGTATAAAAAAGGAGCCTCCACCATGGGTCAGTAGCTTCGCCTAGGACTCCTGAACGAGTGC
CGGGATATTTGGATCATCGCTTGGTTATCGGGAACCTGAAGGTCTTGCGCCAAAGGTTGTGGGGCTCCCCGAT
TGTGCTGAATTGTGCAGAACTGGCTACACTTGTAACTAATCATACTAAGATAAGTAAATCTATTTGATTCTAT
TATTACTTGTGTGTGTGTAAGTTATTGCTCACTAACAGTCTATCAGAAGGCTTAATCATTGATCCTGCATATT
TATTAATATTTGTCATTAATGTCAATTAATACAAATTATTAATATTAATAAAGGTTCACCCCCTTTATTACAT
TTGGCGTAGTCGGCAGGATTAACCCTTTTGATGCTGGGAAGTGCAGTAATAACTTGTATAGAAATTGTAGAAT
TTATAGTGCAGCTTTGTATGGTAGTGTAGTGGCAGTATTGTAGTGGCTTGTGCAGGGCAAACATACTGTTGAA
AGTTAGCAATAATGTTCTGTTGGTTGAAAAACAAAGTTAGAGGGGTAGATGATCAAACGAGCAACCTTCCCAC
ATGGGCTAGTTCCGGACCCTGGACCCATGTTGCTCGGGATGTGATAGGAGATCAGCAACTCTCTCAGGTGACT
CACCCCCATCACCTGTTGCTGAATTTGGATTTGTCTATGTGTTCCAAGGAGCTGAAGTCCTCAGCACAATTGG
GTTGGTTGTGTTTTCAGGCGCTCAGGGAGGAGACAGTCCAAAGGGAGGCAGTTGAGCTTGAGCTGCAGGCAGT
TAAAGATCAACTAGCCACAATGAGAGAATGTTTGCGCTCCTCATTAGCACACAATGAGGAGTTGGAGATAGAA
TTTACAAAATATGTGGTAAGAACTATGAGTAAAAAGCACCATAGAAAAGGGACAGAACAAGCAGAAAATGGTG
TATTAGTTAAAGTAAGAACTCTTGTAAGTAAAAGAGACTAAGGGAGTGATTTATTTCTCAAGATAGAGAAACA
AAGTAAATGTTACATTGTGTTAAATTGTACTGTATGTTAATGTCATATGTTTTTCTGTTTCAGGATTTAAGAA
TCTGATTATTTTGAGTTAATATGGTTTTTGATAACAGGCATCAAGAGTTGATGGATCCATTATTTTTGACACT
TTTTGTTTTGTTTTTTAAGTGCACAAATTTGTGCGTAAAGGCCTTAAGACATGTCTGTTTTTGTTAAAAAAAA
AGTTTAACAATGAAACTTGAAAGTTTTTAACACTTTTTGTTGTATGTTTCTGAGAATGTGTGGGCCCACATGC
ATGTTGTTATGGTTTGTCTTGTCTTAATTGTGACAATGACTTAAATGTGACAGTGACTGAAAAATTCTTGACA
AAGTAATACAAAATTAAGAAAGTTGTTAAAGTTGACAAAACAGATCTTAAATGCTAAGAACTGATGTTGCCTT
GATCTTTTGTTTTATAGATGTTTATCATGGATATAACAAGTGCATTAACAAATGGGTTAATAGCAAAACAATG
GACAGCAAACAAGGATTTGGACATTGTTAATCTTGAATGGACATCAGTACAAAGTATTGAAGAACCTGGAATT
CATTTGAATGGTGACCTGGAAACTTAAATGGACATTGGTTTTGGACTTTATAAATGAATACTTATGTTTTGTT
CAACTGTTTCTACAGACTGAAATTAGCATTGCATACACTATTGTACTAATGGTAATAGGAATGTTATGGCTTG
TTACATGAACTGTAAAGTGCTAGTTAGTGGGTTTTAGAGCACAGGCAGCACAGCTGAACTGTATATCTCCTAT
GGTATATAATATTGTGCAGTGAACCTGCAGTATAGAGAAGGCCCTGGCTGACAATCTGTAGATGCAGGCAAAT
GCCAGAGGTACTGATGTAAGATGCCAGACAGTCACTATCTATTGGGCTGGTGGGGGCTGTTTGGGTCTTTGTG
TATTTGAAATGCCAGGGCCTATTTTGAAATTAGTGTCAACGAAACTCTTTGATAAAACAGAAAGAGTGAAATT
TATTATTCTGAGGGAGAAATTATTATGTGAAACTGACAATGTCTGTAGACAAGTATTATGGTATAAGTAATTA
TTGTAAGTGTGAATACGGTAATGTTATTTTGTGAGTTAGTACTGTGATGTCAATGGGGTTGGCTGAAGGAGAG
TTTAGACAACTCTATATGATTGAAGTTAAAGACTACAACTTAATATACAATTCTGTTGAGGGACACAGTAGTT

TATAAGAAGTCACTGTTAGACTTGATAGTATCATCCCAAGTGATTTGAATGCCCTGTGTGATTTGTGGCCTTA
TGGGTGGAATTTGTGGCTACTAGTGATAGACATATTTTGGAAAAGCATTGGCAGAATGTGTACTGAGGCTAAA
ACTCTGCTGAAAGGAATTCAGTTGCCATTTGGTCTATAAGAGGGGGGTTAGGTAGAAACAAAGTGCATGGCAG
ATACATAATATAGTAAACATATTGCTTATCAAAGAAGCACTGTTTACCTAAGTGAGCTGTAAAGCCTGCAATG
AAACCTGCCCTTGGCCTAATTGTTTTGAGCAGCTGATAGTCTGTAATGAAAATGCAGAGCAAGAGGGATTCCT
ATACATGTTTGCCACATGATTGCCCATGATATGTATCTACAAGTAGACACAGATTTTCTCACTGGACAGAAAT
CATTATAGAGATGTTTTATGACTTGGGATGTAGGTGAGATTTGTTTAATCTTTTGTTAAATCTAGTGTCCTTC
AGTCAGAATTACAGTCATTAATTGTTTATTTGTTTATGGTAATGGGGACAACCAGTGAATGCATATATTGCAT
TGAATGCAGCATTGTATGTTATCTGTATTAGAGGACTTACCAATTTGTATACTGTTGGTTATTATTATCAAAT
CAGTATATTTTGTAAATCAAGGGGTGGAATGTAGCACGGTGGATTTTATTTTTATTTTATAATGATTTACATT
ATATATCTGCTTTGACATATGAATCAAATCTTAGGTTTGAGTCAAGTAAAACCAGCAGATTTTGGGACATCTA
TTGTCCAGTGTTCAAGGTACAATGTCTTTGTTCTATGTATATTGCTGGGTAAACAGTAAACACAGGTATTGTT
ACCCAGCAAATATCCTTGGAATAAACAAACAGACAGCATATAGACAATACACTTTATGTACAACTTATTTGAG
AAACAAATAAGGCTTTCTACAAAGTTAGTAAACTGTCTAGTTAATAGGTTTTGTTCATGACAAAAGTTTGTAC
ACAAGTTAGCACTTGGTGTTGGGGGTTTGCTAAGAACTCTAGTATAAAAAAGGAGCCTCCACCATGGGTCAGT
AGCTTCGCCTAGGACTCCTGAACGAGTGCCGGGATATTTGGATCATCGCTTGGTTATCGGGAACCTGAAGGTC
TTGCGCCAAAGGTTGTGGGGCTCCCCGATTGTGCTGAATTGTGCAGAACTGGCTACACTTGTAACTAATCATA
CTAAGATAAGTAAATCTATTTGATTCTATTATTACTTGTGTGTGTGTAAGTTATTGCTCACTAACAGTCTATC
AGAAGGCTTAATCATTGATCCTGCATATTTATTAATATTTGTCATTAATGTCAATTAATACAAATTATTAATA
TTAATAAAGGTTCACCCCCTTTATTACA

>Xt-S7_CM004451.2:78972808-78976805_ Xenopus tropicalis strain Nigerian chromosome 9, whole genome shotgun sequence
TGTAGCACGGTGGATTTTATTTTGATTTTATAATGATTTACAATATACATCTGCTTTGACATATGAATCAAAT
CTTAGGTTTGAGTCAAGTTAAAGCAGCAGATTTTGGGACAGCTATTGTCCAGTGTTCAAGGTACAATGTCTTT
GTTTTTTTGTATATTGCTGGGTAAACAGTAAACACAGGTATTGTTACCCAGCAAATATCCTTGGAATAAACAA
ACAGACAGCATATAGACAATACACTTTAGGTACAACTTATTTGAGCAACAAATAAGGCCTTAGTTAATAGATT
TTGTTCATGACAAAAGTTTGTACACAAGTCAGCACTTGGTGTTGGGGGTTTTGCTAAGAACTCTAGTATAAAA
AGGAGCCTCCACCATGGGTCAGTAGCTTCGCCTAGGACTCCTGAACGAGTGCCGGGACAATTGGATCATCGCT
TGGTTATCGGGAACCTGAAGGTCTTGCGCCAAAGGTTGTGGGGCTCCCCGATTGTGCTGAATTGTGCAGAACT
GGCTATACTTGTAACTAATCATACTAAGATAAGTAAATCTATTTGATTCTATTATTACTTGTGTGTGTGTAAG
TTATTGCTCTATCAGAAGGCTTAATCATTGATCCTGCATATTTATTAATATTTGTCATTAATGTCAATTAATA
CAAATTATTAATATTAATAAAGGTTCACCCCCTTTATTACATTTGGCGTAGTCGGCAGGATTAACCTTTTTGA
TGCTGGGAAGTGCAGTAATAATTTGTATAGAAATTGTAGAATTTATAGTGTAGCTTTGTATGGTAGTGTAGTG
GCAGTATTGTAGTGGCTTGTGCAGGGCAAACATACTGTTGAAAGTTAGCAATAATGTTTTGTTGGTTGAAAAA
CAAAATTAGAGGGGTAGATGATCAAACGAGCAACCTTCCCACATGGGCTAGTTCCGGACCCTGGACCCATGTT
GCTCGGGATGTGATAGGAGATCAGCAACTCTCTCAGGTGACTCACCCCCATCACCTGTTGCTGAATTTGGATT
TGTCTATGTGTTCCAAGGAGCTGAAGTCCTCAGCACAATTGGGTTGGTTGTGTTTTCAGGCGCTCAGGGAGGA
GACAGTCCAAAGGGAGGCAGTTGAGCTTGAGCTGCAGGCAGTTAAAGATCAACTAGCCACAATGAGAGAATGT
TTGCGCTCCTCATTAGCACACAATGAGGAGTTGGAGATAGAATTTACAAAATATGTGGTAAGAACTATGAGTA
AAAAGCACCATAGAAAAGGGACAGAACAAGCAGAAAATGGTGTATTAGTTAAAGTAAGAACTCTTGTAAGTAA
AAGAGACTAAGGGAGTGATTTATTTCTCAAGATAGAGAAACAAAATAAATGTTACATTGTGTTAAATTGTACT
GTATGTTAATGTCATATGTTTTTCTGTTTCAGGATTTAAGAATCTGATTATTTTGAGTTAATATGGTTTTTGA
TAACAGGCATCAAGAGTTGATGGATCCATTATTTTTGACACTTTTTGTTTTGTTTTTTAAGTGCACAAATTTG
TGCGTAAAGGCCTTAAGACATGTCTGTTTTTGTTAAAAAAAAGTTTAACAATGAAACTTGAAAGTTTTAAACA
CTTTTTGTTGTATGTTTCTGAGAATGTGTGGGCCCACATGCATGTTGTTATGGTTTGTCTTGTCTTAATTGTG
ACAATGACTTAAATGTGACAGTGACTGAAGAATTCTTGACAAAGTAATACAAAATTAAGAAAGTTGTTAAAAT
TGACAAAACAGATCTTAAATGCTAAGAATTGATGTTGCCTTGATCTTTTGTTTTATAGATGTTTATCATGGAT
ATAACAAGTGCATTAACAAATGGGTTAATAGCAAAACAATGGACAGCAAACAAGGATTTGGACATTGTTAATC
TTGAATGGACATCAGTACAAAGTATTGAAGAACCTGGAATTCATTTGAATGGTGACCTGGAAACTTAAATGGA
CATTGGTTTTGGACTTTATAAATTAATATTTATGTTTTGTTCAATTGTTTCTACCGGCTGAAATTAGCATTGC
ATACACTATTGTACTAATGGTAATAGGAATGATATGGCTTGTTACATGAACTGTAAAGTGCTAGTAAGTGGGT
TTTAGAGCACAGGCAGCACAGCTGAACTGTATATCTCCTATGGTATATAATATTGTGCAGTGAACCTGCAGTA
TAGAGCAGGCCCTGGCTGACAATCTATAGATTTAGGCAAATGCCAGAGGGGCTGCTGTAAGATGCCATAGACA

GTCACTATTTATTGGGCTGGTGGGGTCTGTTTGGGTCTCTGTGTATTTGAAATGCCAGGGTCTATTTTGAAAT
TAGTGTCAACAAAACTCTTTGATAAAACAGAAAGAGTGAAATTTATTATTCTGAGGGAGTAATTGTTATGTGA
AACTAACAATGTCTGTAGACAAGCATTATGGTATAATTGTTGTAAGTGTGAATAGGGTAATGTAATTTTATGA
GTTAGTACTATGATATCAATGGAGTTAGCTGAATGTTGGCTGTAAGAGAGTTTGGACAATGCTATATGATTGA
ATTGAAAGCCTTTTAAAGACTACAAGTTAATATACAACTCTGCTGAGGGACACGTTAGTTTACAAGAAGTCAC
TGTTAGACTTGATAGTATCATCCCAAGTGATTTGAATGCCCTGGGTGATTTGTGGCCTTATGGGTGGAATTTG
TGGCTACTGGTGATAGACATATTTTGGAAAAGCATTGGCAGAATGTGTACTGAGGCTACAATTCTGCTGAAAG
GAATTCAGTTGCCATTTGGTATATAAAAGGGGGTTAGGTACAAACAAAGTGCATGGCAGATACATAATATAGT
AAACATTTTGCTTATCAAAGAAGCACTGTTTAACTATGTGAGCTGTAAAGCCTGCAAGGTAACCTGCCCTTGG
CCTAATTGTTTTGAGCAGCTGATGGTCTATAATGAAAATGCAGAGCAAGAGGGGTGCCTATACATGTTTGCCA
CATGTTTGCCCAAGATATGTATCTACAAGTAGACACAGATTTTCTCACTGGACAGAAATCATTTTAGAGGTGT
TTTATGACTTGGGATGTAGGTGAGATTTGTTTAATCTTTTGTTAAATGTAGTGTCCTTCAGTCAGAATTACAG
TAATTAATTGTTTATTTGTTTAGGGTAATGGGGACAACCAGTAAATGCATATATTGCATTGAATGCAGCATTG
TATGTTATCTGTATTAGAGGACTTACCAATTTGTATACTGTTGGTTATTATTATCAAATCAGTATATTTTGTA
AATCAAGGGGTGGAATGTAGCACGGTGGATTTTATTTTGATTTTATAATGATTTACAATATACATCTGCTTTG
ACATATGAATCAAATCTTAGGTTTGAGTCAAGTTAAAGCAGCAGATTTTGGGACAGCTATTGTCCAGTGTTCA
AGGTACAATGTCTTTGTTTTTTTGTATATTGCTGGGTAAACAGTAAACACAGGTATTGTTACCCAGCAAATAT
CCTTGGAATAAACAAACAGACAGCATATAGACAATACACTTTAGGTACAACTTATTTGAGCAACAAATAAGGC
CTTAGTTAATAGATTTTGTTCATGACAAAAGTTTGTACACAAGTCAGCACTTGGTGTTGGGGGTTTTGCTAAG
AACTCTAGTATAAAAAGGAGCCTCCACCATGGGTCAGTAGCTTCGCCTAGGACTCCTGAACGAGTGCCGGGAC
AATTGGATCATCGCTTGGTTATCGGGAACCTGAAGGTCTTGCGCCAAAGGTTGTGGGGCTCCCCGATTGTGCT
GAATTGTGCAGAACTGGCTATACTTGTAACTAATCATACTAAGATAAGTAAATCTATTTGATTCTATTATTAC
TTGTGTGTGTGTAAGTTATTGCTCTATCAGAAGGCTTAATCATTGATCCTGCATATTTATTAATATTTGTCAT
TAATGTCAATTAATACAAATTATTAATATTAATAAAGGTTCACCCCCTTTATTACA

>Xt-S8_CM004452.2:5826453-5822436_ Xenopus tropicalis strain Nigerian chromosome 10, whole genome shotgun sequence
TGTAGCACGGTGGATTTTATTTTGATTTTATAATGATTTACAATATACATCTGCTTTGACATATGAATCAAAT
CTTAGGTTTGAGTCAAGTTAAAGCAGCAGATTTTGGGACAGCTATTGTCCAGTGTTCAAGGTACAATGTCTTT
GTTTTTTTGTATATTGCTGGGTAAACAGTAAACACAGGTATTGTTACCCAGCAAATATCCTTGGAATAAACAA
ACAGACAGCATATAGACAATACACTTTAGGTACAACTTATTTGAGCAACAAATAAGGCCTTAGTTAATAGATT
TTGTTCATGACAAAAGTTTGTACACAAGTCAGCACTTGGTGTTGGGGGTTTTGCTAAGAACTCTAGTATAAAA
AGGAGCCTCCACCATGGGTCAGTAGCTTCGCCTAGGACTCCTGAACGAGTGCCGGGACAATTGGATCATCGAT
TGGTTATCGGGAACCTGAAGGTCTTGCGCCAAAGGTTGTGGGGCTCCCCGATTGTGCTGAATTTATACAGAAC
TGGCTATACTTGTAACTAATCATACTAAGATAAGTAAATCTATTTGATTCTATTATTACTTGTGTGTGTGTAA
GTTATTGCTCACTAACAGTTTATCAGAAGGTTTAATCATTGATCCTGCATATTTATTAATATTTGTCATTAAT
GTCAATTAATACAAATTATTAATATTAATAAAGGTTCCCCCCTTTATTACATTTGGCGTAGTCGGCAGGATTA
ACCTTTTTGATGCTGGGAAGTGCAGTAATAATTTGTATAGAAATTGTAGAATTTATAGTGTAGCTTTGTATGG
TAGTGTAGTGGCAGTATTGTAGTGGCTTGTGCAGGGCAAACATACTGTTGAAAGTTAGCAATAATGTTCTGTT
GGTTGAAAAACAAAATTAGAGGGGTAGATGATCAAACGAGCAACCTTCCCACATGGGCTAGTTCCGGACCCTG
GACCCATGTTGCTCGGGATGTGATAGGAGATCAGCAACTCTCTCAGGTGACTCACCCCCATCACCTGTTGCTG
AATTTGGATTTGTCTATGTGTTCCAAGGAGCTGAAGTCCTCAGCACAATTGGGTTGGTTGTGTTTTCAGGCGC
TCAGGGAGGAGACAGTCCAAAGGGAGGCAGTTGAGCTTGAGCTGCAGGCAGTTAAAGATCAACTAGCCACAAT
GAGAGAATGTTTGCGCTCCTCATTAGCACACAATGAGGAGTTGGAGATAGAATTTACAAAATATGTGGTAAGA
ACTATGAGTAAAAAGCACCATAGAAAAGGGACAGAACAAGCAGAAAATGGTGTATTAGTTAAAGTAAGAACTC
TTGTAAGTAAAAGAGACTAAGGGAGTGATTTATTTCTCAAGATAGAGAAACAAAATAAATGTTACATTGTGTT
AAATTGTACTGTATGTTAATGTCATATGTTTTTCTGTTTCAGGATTTAAGAATCTGATTATTTTGAGTTAATA
TGGTTTTTGATAACAGGCATCAAGAGTTGATGGATCCATTATTTTTGACACTTTTTGTTTTGTTTTTTAAGTG
CACAAATTTGTGCGTAAAGGCCTTAAGACATGTCTGTTTTTGTTAAAAAAAAGTTTAACAATGAAACTTGAAA
GTTTTAAACACTTTTTGTTGTATGTTTCTGAGAATGTGTGGGCCCACATGCATGTTGTTATGGTTTGTCTTGT
CTTAATTGTGACAATGACTTAAATGTGACAGTGACTGAAGAATTCTTGACAAAGTAATACAAAATTAAGAAAG
TTGTTAAAATTGACAAAACAGATCTTAAATGCTAAGAATTGATGTTGCCTTGATCTTTTGTTTTATAGATGTT
TATCATGGATATAACAAGTGCATTAACAAATGGGTTAATAGCAAAACAATGGACAGCAAACAAGGATTTGGAC
ATTGTTAATCTTGAATGGACATCAGTACAAAGTATTGAAGAACCTGGAATTCATTTGAATGGTGACCTGGAAA

CTTAAATGGACATTGGTTTTGGACTTTATAAATTAATATTTATGTTTTGTTCAATTGTTTCTACCGGCTGAAA
TTAGCATTGCATACACTATTGTACTAATGGTAATAGGAATGATATGGCTTGTTACATGAACTGTAAAGTGCTA
GTAAGTGGGTTTTAGAGCACAGGCAGCACAGCTGAACTGTATATCTCCTATGGTATATAATATTGTGCAGTGA
ACCTGCAGTATAGAGCAGGCCCTGGCTGACAATCTATAGATTTAGGCAAATGCCAGAGGTGCTGCTGTAAGAT
GCCATAGACAGTCACTATTTATTGGGCTGGTGGGGTCTGTTTGGGTCTCTGTGTATTTGAAATGCCAGGGTCT
ATTTTGAAATTAGTGTCAACAAAACTCTTTGATAAAACAGAAAGAGTGAAATTTATTATTCTGAGGGAGTAAT
TGTTATGTGAAACTAACAATGTCTGTAGACAAGCATTATGGTATAATTGTTGTAAGTGTGAATAGGGTAATGT
AATTTTATGAGTTAGTACTATGATATCAATGGAGTTAGCTGAATGTTGGCTGTAAGAGAGTTTGGACAATGCT
ATATGATTGAATTGAAAGCCTTTTAAAGACTACAAGTTAGTATACAACTCTGCTGAGGGACACGTTAGTTTAC
AAGAAGTCACTGTTAGACTTGATAGTATCATCCCAAGTGATTTGAATGCCCTGGGTGATTTGTGGCCTTATGG
GTGGAATTTGTGGCTACTGGTGATAGACATATTTTGGAAAAGCATTGGCAGAATGTGTACTGAGGCTACAATT
CTGCTGAAAGGAATTCAGTTGCCATTTGGTATATAAAAGGGGGTTAGGTACAAACAAAGTGCATGGCAGATAC
ATAATATAGTAAACATTTTGCTTATCAAAGAAGCACTGTTTAACTATGTGAGCTGTAAAGCCTGCAAGGTAAC
CTGCCCTTGGCCTAATTGTTTTGAGCAGCTGATGGTCTATAATGAAAATGCAGAGCAAGAGGGGTGCCTATAC
ATGTTTGCCACATGTTTGCCCAAGATATGTATCTACAAGTAGACACAGATTTTCTCACTGGACAGAAATCATT
TTAGAGGTGTTTTATGACTTGGGATGTAGGTGAGATTTGTTTAATCTTTTGTTAAATGTAGTGTCCTTCAGTC
AGAATTACAGTAATTAATTGTTTATTTGTTTAGGGTAATGGGGACAACCAGTAAATGCATATATTGCATTGAA
TGCAGCATTGTATGTTATCTGTATTAGAGGACTTACCAATTTGTATACTGTTGGTTATTATTATCAAATCAGT
ATATTTTGTAAATCAAGGGGTGGAATGTAGCACGGTGGATTTTATTTTGATTTTATAATGATTTACAATATAC
ATCTGCTTTGACATATGAATCAAATCTTAGGTTTGAGTCAAGTTAAAGCAGCAGATTTTGGGACAGCTATTGT
CCAGTGTTCAAGGTACAATGTCTTTGTTTTTTTGTATATTGCTGGGTAAACAGTAAACACAGGTATTGTTACC
CAGCAAATATCCTTGGAATAAACAAACAGACAGCATATAGACAATACACTTTAGGTACAACTTATTTGAGCAA
CAAATAAGGCCTTAGTTAATAGATTTTGTTCATGACAAAAGTTTGTACACAAGTCAGCACTTGGTGTTGGGGG
TTTTGCTAAGAACTCTAGTATAAAAAGGAGCCTCCACCATGGGTCAGTAGCTTCGCCTAGGACTCCTGAACGA
GTGCCGGGACAATTGGATCATCGATTGGTTATCGGGAACCTGAAGGTCTTGCGCCAAAGGTTGTGGGGCTCCC
CGATTGTGCTGAATTTATACAGAACTGGCTATACTTGTAACTAATCATACTAAGATAAGTAAATCTATTTGAT
TCTATTATTACTTGTGTGTGTGTAAGTTATTGCTCACTAACAGTTTATCAGAAGGTTTAATCATTGATCCTGC
ATATTTATTAATATTTGTCATTAATGTCAATTAATACAAATTATTAATATTAATAAAGGTTCCCCCCTTTATT
ACA

>Xt-S9_CM004445.2:116537647-116541655_ Xenopus tropicalis strain
Nigerian chromosome 3, whole genome shotgun sequence
TGTAGCACGGTGGATTTTATTTTGATTTTATAATGATTTACAATATACATCTGCTTTGACATATGAATCAAAT
CTTAGGTTTGAGTCAAGTTAAAGCAGCAGATTTTGGGACAGCTATTGTCCAGTGTTCAAGGTACAATGTCTTT
GTTTTTTTGTATATTGCTGGGTAAACAGTAAACACAGGTATTGTTACCCAGCAAATATCCTTGGAATAAACAA
ACAGACAGCATATAGACAATACACTTTAGGTACAACTTATTTGAGCAACAAATAAGGCCTTAGTTAATAGATT
TTGTTCATGACAAAAGTTTGTACACAAGTCAGCACTTGGTGTTGGGGGTTTTGCTAAGAACTCTAGTATAAAA
AGGAGCCTCCACCATGGGTCAGTAGCTTCGCCTAGGACTCCTGAACGAGTGCCGGGACAATTGGATCATCGAT
TGGTTATCGGGAACCTGAAGGTCTTGCGCCAAAGGTTGTGGGGCTCCCCGATTGTGCTGAATTGTGCAGAACT
GGCTATACTTGTAACTAATCATACTAAGATAAGTAAATCTATTTAATTCTATTATTACTTGTGTGTGTGTAAG
TTATTGCTCACTAACAGTCTATCAGAAGGCTTAATCATTGATCCTGCATATTTATTAATATTTGTCATTAATG
TCAATTAATACAAATTATTAATATTGATAAAGGTTAACCCCCTTTATTACATTTGGCGTAGTCGGCAGGATTA
ACCCTTTTGATGCTGGGAAGTGCAGTAATAACTTGTATAGAAATTGTAGAATTTATAGTGCAGCTTTGTATGG
TAGTGTAGTGGCAGTATTGTAGTGGCTTGTGCAGGGCAAACATACTGTTGAAAGTTAGCAATAATGTTCTGTT
GGTTGAAAAACAAAGTTAGAGGGGTAGATGATCAAACGAGCAACCTTCCCACATGGGCTAGTTCCGGACCCTG
GACCCATGTTGCTCGGGATGTGATAGGAGATCAGCAACTCTCTCAGGTGACTCACCCCGATCACCTGTTGCTG
AATTTGGATTTGTCTATGTGTTCCAAGGAGCAGAAGTCCTCAGCACAATTGGGTTGGTTGTGTTTTCAGGCGC
TCAGGGAGGAGACAGTCCAAAGGGAGGCAGTTGAGTTTGAGCTGCAGGCAGTTAAAGATCAACTAGCCACAAT
GAGAGAATGTTTGCGCTCCTCATTAGCACACAATGAGGAGTTGGAGATAGAATTTACAAAATATGTGGTAAGA
ACTATGAGTAAAAAGCACCATAGAAAAGAGACAGAACAAGCAGAAAATGGTGTATTAGTTAAAGTAAGAACTC
TTGTAAGTAAAAGAGACTAAGGGAGTGATTTATTTCTCAAGATAGAGAAACAAAATAAATGTTACATTGTGTT
TAATTGTACTGTATGTTAATGTCATATGTTTTCTGTTTCAGGATTTAAGAATCTGATTATTTTGAGTTAATAT
GGTTTTTAATAACAGGCATCAAGAGTTGATGGATCCATTATTTTTGACACTTTTTGTTTTGTTTTTTTTAAGT
GCACAAATTTGTGCGTAAAGGCCTTAAGACATGTCTGTTTTTGTTAAAAAAAAAGTTTAACAATGAAACTTGA

GAGTTTTTAACACTTTTTGTTGTATGTTTCTGAGAATGTGTGGGCCCACATGCATGTTGTTATGGTTTGTCTT
GTCTTAATTGTGACAATGACTTAAATGTGACAGTGACTGAAAAATTCTTGACAAAGTAATACAAAATTAAGAA
AGTTGTTAAAGTTGACAAAACAGATCTTAAATGCTAAGAACTGATGTTGCCTTGATCTTTTGTTTTATAGATG
TTTATCAAGTGCATTAACAAATGGGTTAATAGCAAAACAATTGACAGCAAACAAGGATTTGGACATTGTTAAT
CTTCAATGGACATCAGTACAAAGTATTGAAGAACCTGGAATTCAGTTGAATGGTGACTTGGAAACTTAAATGG
ACATTGGTTTTGGACTTTATAAATGAATACTTATGTTTTGTTCAATTGTTTCCACAGACTGAAATTAGCATTG
CATACACTATTGTACTAATGGTAATAGGAATGTTATGGCTTGTTACATGAACTGTAAAGTGCCAGTTAGTGGG
TTTTATAGCACAGGCAGCACAGCTGAACTGTATATCTCCTATGGTATATAATATTGTGCAGTGAACCTGCAGT
ATAGAGCAGGCCCTGGCTGACAATCTGTAGATTTAGGCAAATGCCAGAGGTGCTGCTGTAAGATGCCATAGAC
AGTCACTATTTATTGGGCTGGTGGGGTCTGTTTGGGTCTCTGTGTATTTGAAATGCCAGGGTCTATTTTGAAA
TTAGTGTCAACAAAACTCTTTGATAAAACAGAAAGAGTGAAATTTATTATTCTGAGGGAGTAATTGTTATGTG
AAACTAACAATGTCTGTAGACAAGCATTATGGTATAATTGTTGTAAGTGTGAATAGGGTAATGTAATTTTATG
AGTTAGTACTATGATATCAATGGAGTTAGCTGAATGTTGGCTGTAAGAGAGTTTGGACAATGCTATATGATTG
AATTGAAAGCCTTTTAAAGACTACAAGTTAGTATACAACTCTGCTGAGGGATACGTTAGTTTACAAGAAGTCA
CTGTTAGACTTGATAGTATCATCCCAAGTGATTTGAATGCCCTGGGTGATTTGTGGCCTTATGGGTGGAATTT
GTGGCTACTGGTGATAGACATATTTTGGAAAAGCATTGGCAGAATGTGTACTGAGGCTACAATTCTGCTGAAA
GGAATTCAGTTGCCATTTGGTATATAAAAGGGGGTTAGGTACAAACAAAGTGCATGGCAGATACATAATATAG
TAAACATTTTGCTTATCAAAGAAGCACTGTTTAACTATGTGAGCTGTAAAGCCTACAAGGTAACCTGCCCTTG
GCCTAATTGTTTTGAGCAGCTGATGGTCTATAATGAAAATGCAGAGCAAGAGGGGTGCCTATACATGTTTGCC
ACATGTTTGCCCAAGATATGTATCTACAAGTAGACACAGATTTTCTCACTGGACAGAAATCATTTTAGAGGTG
TTTTATGACTTGGGATGTAGGTGAGATTTGTTTAATCTTTTGTTAAATGTAGTGTCCTTCAGTCAGAATTACA
GTAATTAATTGTTTATTTGTTTAGGGTAATGGGGACAACCAGTAAATGCATATATTGCATTGAATGCAGCATT
GTATGTTATCTGTATTAGAGGACTTACCAATTTGTATACTGTTGGTTATTATTATCAAATCAGTATATTTTGT
AAATCAAGGGGTGGAATGTAGCACGGTGGATTTTATTTTGATTTTATAATGATTTACAATATACATCTGCTTT
GACATATGAATCAAATCTTAGGTTTGAGTCAAGTTAAAGCAGCAGATTTTGGGACAGCTATTGTCCAGTGTTC
AAGGTACAATGTCTTTGTTTTTTTGTATATTGCTGGGTAAACAGTAAACACAGGTATTGTTACCCAGCAAATA
TCCTTGGAATAAACAAACAGACAGCATATAGACAATACACTTTAGGTACAACTTATTTGAGCAACAAATAAGG
CCTTAGTTAATAGATTTTGTTCATGACAAAAGTTTGTACACAAGTCAGCACTTGGTGTTGGGGGTTTTGCTAA
GAACTCTAGTATAAAAAGGAGCCTCCACCATGGGTCAGTAGCTTCGCCTAGGACTCCTGAACGAGTGCCGGGA
CAATTGGATCATCGATTGGTTATCGGGAACCTGAAGGTCTTGCGCCAAAGGTTGTGGGGCTCCCCGATTGTGC
TGAATTGTGCAGAACTGGCTATACTTGTAACTAATCATACTAAGATAAGTAAATCTATTTAATTCTATTATTA
CTTGTGTGTGTGTAAGTTATTGCTCACTAACAGTCTATCAGAAGGCTTAATCATTGATCCTGCATATTTATTA
ATATTTGTCATTAATGTCAATTAATACAAATTATTAATATTGATAAAGGTTAACCCCCTTTATTACA

>Xt-S10_CM004447.2:151155029-151151033_ Xenopus tropicalis strain Nigerian chromosome 5, whole genome shotgun sequence
TGTAGCACGGTGGATTTTATTTTGATTTTATAATGATTTACAATATACATCTGCTTTGACATATGAATCAAAT
CTTAGGTTTGAGTCAAGTTAAAGCAGCAGATTTTGGGACAGCTATTGTTCAAGGTACAATGTCTTTGTTTTTT
TGTATATTGCTGGGTAAACAGTAAACACAGGTATTGTTACCCAGCAAATATCCTTGGAATAAACAAACAGACA
GCATATAGACAATACACTTTTTAGGTACAACTTATTTGAGCAACAAATAAGGCCTTCTACAAAGTCTAGTTAA
TAGATTTTGTTCATGACAAAAGTTTGTACACAAGTCAGCACTTGGTGTTGGGGGTTTGCTAAGAACTCTAGTA
TAAAAAGGAGCCTCCACCATGGGTCAGTAGCTTCGCCTAGGACTCCTGAACGAGTGCCGGGACAATTGGATCA
TCGATTGGTTATCGGGAACCTGAAGGTCTTGCGCCAAAGGTTGTGGGGCTCCCCGATTGTGCTGAATTGTGCA
GAACTGGCTATACTTGTAACTAATCATACTAAGATAAGTAAATCTATTTAATTCTATTATTACTTGTGTGTGT
GTAAGTTATTGCTCACTAACAGTCTATCAGAAGGCTTAATCATTGATCCTGCATATTTATTAATATTTGTCAT
TAATGTCAATTAATACAAATTATTAATATTGATAAAGGTTAACCCCCTTTATTACATTTGGCGTAGTCGGCAG
GATTAACCCTTTTGATGCTGGGAAGTGCAGTAATAACTTGTATAGAAATTGTAGAATTTATAGTGCAGCTTTG
TATGGTAGTGTAGTGGCAGTATTGTAGTGGCTTGTGCAGGGCAAACATACTGTTGAAAGTTAGCAATAATGTT
CTGTTGGTTGAAAAACAAAGTTAGAGGGGTAGATGATCAAACGAGCAACCTTCCCACATGGGCTAGTTCCGGA
CCCTGGACCCATGTTGCTCGGGATGTGATAGGAGATCAGCAACTCTCTCAGGTGACTCACCCCGATCACCTGT
TGCTGAATTTGGATTTGTCTATGTGTTCCAAGGAGCAGAAGTCCTCAGCACAATTGGGTTGGTTGTGTTTTCA
GGCGCTCAGGGAGGAGACAGTCCAAAGGGAGGCAGTTGAGTTTGAGCTGCAGGCAGTTAAAGATCAACTAGCC
ACAATGAGAGAATGTTTGCGCTCCTCATTAGCACACAATGAGGAGTTGGAGATAGAATTTACAAAATATGTGG
TAAGAACTATGAGTAAAAAGCACCATAGAAAAGAGACAGAACAAGCAGAAAATGGTGTATTAGTTAAAGTAAG

AACTCTTGTAAGTAAAAGAGACTAAGGGAGTGATTTATTTCTCAAGATAGAGAAACAAAATAAATGTTACATT
GTGTTTAATTGTACTGTATGTTAATGTCATATGTTTTCTGTTTCAGGATTTAAGAATCTGATTATTTTGAGTT
AATATGGTTTTTAATAACAGGCATCAAGAGTTGATGGATCCATTATTTTTGACACTTTTTGTTTTGTTTTTTT
AAGTGCACAAATTTGTGCGTAAAGGCCTTAAGACATGTCTGTTTTTGTTAAAAAAAAAGTTTAACAATGAAAC
TTGAGAGTTTTTAACACTTTTTGTTGTATGTTTCTGAGAATGTGTGGGCCCACATGCATGTTGTTATGGTTTG
TCTTGTCTTAATTGTGACAATGACTTAAATGTGACAGTGACTGAAAAATTCTTGACAAAGTAATACAAAATTA
AGAAAGTTGTTAAAGTTGACAAAACAGATCTTAAATGCTAAGAACTGATGTTGCCTTGATCTTTTGTTTTATA
GATGTTTATCAAGTGCATTAACAAATGGGTTAATAGCAAAACAATTGACAGCAAACAAGGATTTGGACATTGT
TAATCTTCAATGGACATCAGTACAAAGTATTGAAGAACCTGGAATTCAGTTGAATGGTGACTTGGAAACTTAA
ATGGACATTGGTTTTGGACTTTATAAATGAATACTTATGTTTTGTTCAATTGTTTCCACAGACTGAAATTAGC
ATTGCATACACTATTGTACTAATGGTAATAGGAATGTTATGGCTTGTTACATGAACTGTAAAGTGCTAGTTAG
TGGGTTTTATAGCACAGGCAGCACAGCTGAACTGTATATCTCCTATGGTATATAATATTGTGCAGTGAACCTG
CAGTATAGAGCAGGCCCTGGCTGACAATCTGTAGATTTAGGCAAATGCCAGAGGTGCTGCTGTAAGATGCCAT
AGACAGTCACTATTTATTGGGCTGGTGGGGTCTGTTTGGGTCTCTGTGTATTTGAAATGCCAGGGTCTATTTT
GAAATTAGTGTCAACAAAACTCTTTGATAAAACAGAAAGAGTGAAATTTATTATTCTGAGGGAGAAATTGTTA
TGTGAAACTAACAATGTCTGTAGACAAGCATTATGGTATAATTGTTGTAAGTGTGAATAGGGTAATGTAATTT
TATGAGTTAGTACTGTGATGTCAATGGGGTTGGCTGAAGGAGAGTTTAGACAACTCTATATGATTGAAGTTAA
AGACTACAACTTAATACACAATTCTGTTGAGGGACACAGTAGTTTACAAGAAGTCACTGTTAGACTTGATAGT
ATCATCCCAAGTGATTTGAATGCCCTGGGTGATTTGTGGCCTTATGGGTGGAATTTGTGGCTACTGGTGATAG
ACATATTTTGGAAAAGCATTGGCAGAATGTGTACTGAGGCTACAATTCTGCTGAAAGGAATTCAGTTGCCATT
TGGTATATAAAAGGGGGTTAGGTACAAACAAAGTGCATGGCAGATACATAATATAGTAAACATTTTGCTTATC
AAAGAAGCACTGTTTAACTATGTGAGCTGTAAAGCCTGCAAGGTAACCTGCCCTTGGCCTAATTGTTTTGAGC
AGCTGATGGTCTATAATGAAAATGCAGAGCAAGAGGGATGCCTATACATGTTTGCCACATGTTTGCCCGAGAT
ATATATCTACAAGTAGACACAGATTTTCTCACTGGACAGAAATCATTATAGAGGTGTTTTATGACTTGGGATG
TAGGTGAGATGTGTTTAATCTTTTGTTAAATGTAGTGTCCTTCAGTCAGAATTACAGTCATTAATTGTTTATT
TGTTTAGGGTAATGGGGACAACCAGTAAATGCATATATTGCATTGAATGCAGCATTGTATGTTATCTGTATTA
GAGGACTTACCAATTTGTATACTGTTGGTTATTATTATCAAATCAGTATATTTTGTAAATCAAGGGGTGGAAT
GTAGCACGGTGGATTTTATTTTGATTTTATAATGATTTACAATATACATCTGCTTTGACATATGAATCAAATC
TTAGGTTTGAGTCAAGTTAAAGCAGCAGATTTTGGGACAGCTATTGTTCAAGGTACAATGTCTTTGTTTTTTT
GTATATTGCTGGGTAAACAGTAAACACAGGTATTGTTACCCAGCAAATATCCTTGGAATAAACAAACAGACAG
CATATAGACAATACACTTTTTAGGTACAACTTATTTGAGCAACAAATAAGGCCTTCTACAAAGTCTAGTTAAT
AGATTTTGTTCATGACAAAAGTTTGTACACAAGTCAGCACTTGGTGTTGGGGGTTTGCTAAGAACTCTAGTAT
AAAAAGGAGCCTCCACCATGGGTCAGTAGCTTCGCCTAGGACTCCTGAACGAGTGCCGGGACAATTGGATCAT
CGATTGGTTATCGGGAACCTGAAGGTCTTGCGCCAAAGGTTGTGGGGCTCCCCGATTGTGCTGAATTGTGCAG
AACTGGCTATACTTGTAACTAATCATACTAAGATAAGTAAATCTATTTAATTCTATTATTACTTGTGTGTGTG
TAAGTTATTGCTCACTAACAGTCTATCAGAAGGCTTAATCATTGATCCTGCATATTTATTAATATTTGTCATT
AATGTCAATTAATACAAATTATTAATATTGATAAAGGTTAACCCCCTTTATTACA

>Xt-S11_CM004447.2:28728451-28724434_ Xenopus tropicalis strain Nigerian chromosome 5, whole genome shotgun sequence
TGTAGCACGGTGGATTTTATTTTGATTTTATAATGATTTACAATATACATCTGCTTTGACATATGAATCAAAT
CTTAGGTTTGAGTCAAGTTAAAGCAGCAGATTTTGGGACAGCTATTGTTCAAGGTACAATGTCTTTGTTTTTT
TTGTATATTGCTGGGTAAACAGTAAACACAGGTATTGTTACCCAGCAAATATCCTTGGAATAAACAAACAGAC
AGCATATAGACAATACACTTTAGGTACAACTTATTTGAGCAACAAATAAGGCCTTCTACAAAGTCTAGTTAAT
AGATTTTGTTCATGACAAAAGTTTGTACACAAGTCAGCACTTGGTGTTGGGGGTTTTGCTAAGAACTCTAGTA
TAAAAAGGAGCCTCCACCATGGGTCAGTAGCTTCGCCTAGGACTCCTGAACGAGTGCCGGGACAATTGGATCA
TCGCTTGGTTATCGGGAACCTGAAGGTCTTGCGCCAAGGTTGTGGGGCTCCCCGATTGTGCTGAATTGTGCAG
AACTGGCTATACTTGTAACTAATCATACTAAGATAAGTAAATCTATTTAATTCTATTATTACTTGTGTGTGTG
TAAGTTATTGCTCACTAACAGTCTATCAGAAGGCTTAATCATTGATCCTGCATATTTATTAATATTTGTCATT
AATGTCAATTAATACAAATTATTAATATTAATAAAGGTTCACCCCCTTTATTACATTTGGCGTAGTCGGCAGG
ATTAACCCTTTTGATGCTGGGAAGTGCAGTAATAACTTGTATAGAAATTGTAGAATTTATAGTGTAGCTTTGT
ATGGTAGTGTAGTGGCAGTATTGTAGTGGCTTGTGCAGGGCAAACATACTGTTGAAAGTTAGCAATAATGTTC
TGTTGGTTGAAAAACAAAGTTAGAGGGGTAGATGATCAAACGAGCAACCTTCCCACATGGGCTAGTTCCGGAC
CCTGGACCCATGTTGCTCGGGATGTGATAGGAAATCAGCAACTCTCTCAGGTGACTCACCCCCATCACCTGTT

GCTGAATTTGGATTTGTCTATGTGTACCAAGGAGCTGAAGTCCTCAGCACAATTGGGTTGGTTGTGTTTTCAG
GCGCTCAGGGAGGAGACAGTCCAAAGGGAGGCAGTTGAGCTTGAGCTGCAGGCAGTTAAAGATCAACTAGCCA
CAATGAGAGAATGTTTGCGCTCCTCATTAGCACACAATGAGGAGTTGGAGATAGAATTTGCAAAATATGTGGT
AAGAACTATGAGTAAAAAGCACCATAGAAAAGGGACAGAACAAGCAGAAAATGGTGTATTAGTTAAAGTAAGA
ACTCTTGTAAGTAAAAGAGACTAAGGGAGTGATTTATTTCTCAAGATGGAGAAACAAAATAAATGTTACATTG
TGTTAAATTGTACTGTATGTTAATGTCATATGTTTTTCTGTTTCAGGATTTAAGAATCTGATTATTTTGAGTT
AATATGGTTTTTGATAACAGGCATCAAGAGTTGATGGATCCATTATTTTTGACACTTTTTGTTTTGTTTTTTA
AGTGCACAAATTTGTGCGTAAAGGCCTTAAGACATGTCTGTTTTTGTTAAAAAAAAAAAGTTTAACTATTAAA
CTTGAAAGTTTTTAACACTTTTTGTTGTATGTTTTTGAGAATGTGTGGGCCCACATGCATGTTGTTATGGTTT
GTCTTGTCTTAATTGTGACAATGATTTAAATGTGACAGTGACTGAAAAATTCTTGACAAAATAATACAAAATT
AAGAAAGTTGATAAAGTTGACAAAACAGATCTTAAATGCTAAGAACTGATGTTGCCTTGATCTTTTGTTTTAT
AGATGTTTATCAAGTGCATTAACAAATGGGTTAATAGCAAAACAATGGACAGCAAACAAGGATTTGGACATTG
TTATCTTCAATGGACATCAGTACAAAGTATTGAAGAACCTGGAATTCATTTGAATGGTGACCTGGAAACTTAA
ATGGACATTGGTTTTGGACTTTATAAATGAATACTTATGTTTTGTTCAACTGTTTCTACAGACTGAAATAAGC
ATTGCATACACTATTGTACTAATGGTAATAGGAATGTTATGGCTTGTTACATGAACTGTAAAGTGCTAATAAG
TGGGTTTTAGAGCACAGGCAGCACAGCTGAACTGTATATCTCCTATGGTATATAATATTGTGCAGTGAACCTG
CAGTATAGAGCAGGCCCTGGCTGACAATCTAAAGATTTAGGCAAATGCCAGAGGTGCTGCTGTAAGATGCCAT
AGACAGTCACTATTTATTGGGCTGGTGGGGTCTGTTTGGGTCTCTGTGTATTTGAAATGCCAGGGTCTATTTT
GAAATTAGTGTCAACAAAACTCTTTGATAAAACAGAAAGAGTGAAATTTATTATTCTGAGGGAGAAATTGTTA
TGTGAAACTAACAATGTCTGTAGACAAGCATTATGGTATAATTGTTGTAAGTGTGAATAGGGTAATGTAATTT
TATGAGTTAGTACTATGATATCAATGGAGTTAGCTGAATGTTGGCTGTAAGAGAGTTTGGACAACTCTATATG
ATTGAAGTGAAAGCCTTTTAAAGACTACAAGTTAATACACAATTCTGCTGAGGGACACGTTAGTTTACAAGAA
GTCACTGTTAGACTTGATAGTATCATCCCAAGTGATTTGAATGCCCTGGGTGATTTGTGGCCTTATGGGTGGA
ATTTGTGGCTACTGGTGATAGACATATTTTGGAAAAGCATTGGCAGAATGTGTACTGAGGCTACAATTCTGCT
GAAAGGAATTCAGTTGCCATTTGGTATATAAAAGGGGGTTAGGTACAAACAAAGTGCATGGCAGATACATAAT
ATAGTAAACATTTTGCTTATCAAAGAAGCACTGTTTAACTATGTGAGCTGTAAAGCCTGCAAGGTAACCTGCC
CTTGGCCTAATTGTTTTGAGCAGCTGATGGTCTATAATGAAAATGCAGAGCAAGAGGGATTCCTATACATGTT
TGCCACATGTTTGCCCAAGATATGTATCTACAAGTAGACACAGATTTTCTCACTGGACAGAAATCATTATAAA
GGTGTTTTATGACTTGGGATGTAGGTGAGATTTGTTTAATCTTTTGTTAAATGTAGTGTCCTTCAGTCAGAAT
TACAGTCATTAATTGTTTATTTGTTTAGGGTAATGACAACCAGTAAATGCATATATTGCATTGAATGCAGCAT
TTTATGTTATCTGTATTAGAGGACTTACCAATTTGTATACTGTTGGTTATTATTATCAAATCAGTATATTTTG
TAAATCAAGGGGTGGAATGTAGCACGGTGGATTTTATTTTGATTTTATAATGATTTACAATATATATACATCT
GCTTTGACATATGAATCAAATCTTAGGTTTGAGTCAAGTTAAAGCAGCAGATTTTGGGACAGCTATTGTTCAA
GGTACAATGTCTTTGTTTTTTTTGTATATTGCTGGGTAAACAGTAAACACAGGTATTGTTACCCAGCAAATAT
CCTTGGAATAAACAAACAGACAGCATATAGACAATACACTTTAGGTACAACTTATTTGAGCAACAAATAAGGC
CTTCTACAAAGTCTAGTTAATAGATTTTGTTCATGACAAAAGTTTGTACACAAGTCAGCACTTGGTGTTGGGG
GTTTTGCTAAGAACTCTAGTATAAAAAGGAGCCTCCACCATGGGTCAGTAGCTTCGCCTAGGACTCCTGAACG
AGTGCCGGGACAATTGGATCATCGCTTGGTTATCGGGAACCTGAAGGTCTTGCGCCAAGGTTGTGGGGCTCCC
CGATTGTGCTGAATTGTGCAGAACTGGCTATACTTGTAACTAATCATACTAAGATAAGTAAATCTATTTAATT
CTATTATTACTTGTGTGTGTGTAAGTTATTGCTCACTAACAGTCTATCAGAAGGCTTAATCATTGATCCTGCA
TATTTATTAATATTTGTCATTAATGTCAATTAATACAAATTATTAATATTAATAAAGGTTCACCCCCTTTATT
ACA

>Xt-S12_CM004445.2:51361566-51357539_ Xenopus tropicalis strain Nigerian chromosome 3, whole genome shotgun sequence
TGTAGCACAGTGGATTTTATTTTGATTTTATAATGATTTACAATATACATCTGCTTTGACATATGAATCAAAT
CTTAGGTTTGAGTCAAGTTAAAGCAGCAGATTTTGGGACATCTATTGTCCAGTGTTCAAGGTACAATGTCTTT
GTTTTTTGAATATTGCTGGGTAAACAGTAAACACAGGTATTGTTACCCAGCAAATATCCTTGGAATAAACAAA
CAGACAGCATATAGACAATACACTTTAGGTACAACTTATTTGAGCAACAAATAAGGCCTTCTACAAAGTCTAG
TTAATAGATTTTGTTCATGACAAAAGTTTGTACACAAGTCAGCACTTGGTGTTGGGGGTTTTGCTAAGAACTC
TAGTATAAAAAGGAGCCTCCACCATGGGTCAGTAGCTTCGCCTAGGACTCCTGAAAGAGTGCCGGGACAATTG
GATCATCGCTTGGTTATCGGGAACCTGAAGGTCTTGCGCCAAAGGTTGTGGGGCTCCCCGATTGTGCTGAATT
GTGCAGAACTGGCTATACTTGTAACTAATCATACTAAGATAAGTAAATCTATTTAATTCTATTATTACTTGTG
TGTGTGTAAGTTATTGCTCACTAACAGTCTATCAGAAGGTTTAATCATTGATCCTGCATATTTATTAATATTT

GTCATTAATGTCAATTAATACAAATTATTAATATTAATAAAGGTTCACCCCCTTTATTACATTTGGCGTAGTC
GGCAGGATTAACCTTTTTGATGCTGGGAAGTGCAGTAATAATTTGTATAGAAATTGTAGAATTTATAGTGTAG
CTTTGTATGGTAGTGTAGTGGCAGTATTGTAGTGGCTTGTGCAGGGCAAACATACTGTTGAAAGTTAGCAATA
ATGTTCTGTTGGTTGAAAAACAAAATTAGAGGGGTAGATGATCAAACGAGCAACCTTCCCACATGGGCTAGTT
CCGGACCCTGGACCCATGTTGCTCGGGATGTGATAGGAGATCAGCAACTCTCTCAGGTGACTCACCCCCATCA
CCTGTTGCTGAATTTGGATTTGTCTATGTGTTCCAAGGAGCTGAAGTCCTCAGCACAATTGGGTTGGTTGTGT
TTTCAGGCGCTCAGGGAGGAGACAGTCCAAAGGGAGGCAGTTGAGCTTGAGCTGCAGGCAGTTAAAGATCAAC
TAGCCACAATGAGAGAATGTTTGCGCTCCTCATTAGCACACAATGAGGAGTTGGAGATAGAATTTACAAAATA
TGTGGTAAGAACTATGAGTAAAAAGCACCATAGAAAAGGGACAGAACAAGCAGAAAATGGTGTATTAGTTAAA
GTAAGAACTCTTGTAAGTAAAAGAGACTAAGGGAGTGATTTATTTCTCAAGATAGAGAAACAAAATAAATGTT
ACATTGTGTTAAATTGTACTGTATGTTAATGTCATATGTTTTTCTGTTTCAGGATTTAAGAATCTGATTATTT
TGAGTTAATATGGTTTTTGATAACAGGCATCAAGAGTTGATGGATCCATTATTTTTGACACTTTTTGTTTTGT
TTTTTAAGTGCACAAATTTGTGCGTAAAGGCCTTAAGACATGTCTGTTTTTGTTAAAAAAAAGTTTAACAATG
AAACTTGAAAGTTTTAAACACTTTTTGTTGTATGTTTCTGAGAATGTGTGGGCCCACATGCATGTTGTTATGG
TTTGTCTTGTCTTAATTGTGACAATGACTTAAATGTGACAGTGACTGAAAAATTCTTGACAAAGTAATACAAA
ATTAAGAAAGTTGATAAAGTTGACAAAACAGATCTTAAATGCTAAGAACTGATGTTGCCTTGATCTTTTGTTT
TATAGATGTTTATCAAGTGCATTAACAAATGGGTTAATAGCAAAACAATGGACAGCAAACAAGGATTTGGACA
TTGTTATCTTCAATGGACATCAGTACAAAGTATTGAAGAACCTGGAATTCATTTGAATGGTGACCTGGAAACT
TAAATGGACATTGGTTTTGGACTTTATAAATGAATACTTATGTTTTGTTCAACTGTTTCTACAGACTGAAATT
AGCATTGCATACACTATTGTACTAATGGTAATAGGAATATTATGGCTTGTTAAATGAACTGTTAAGTGCTAGT
TAGTGGGTTTTATAGCACAGGCAGCACAGCTGAACTGTATATCTCCTATGGTATATGATATTGTGCAGTGAAC
CTGCAGTATAGAGCAGGCCCTGGCTGACAATCTGTAGATTCAGGCAAATGCCAGAGGGGCTGCTGTAAGATGC
CATAGACAGTCACTATTTATTGGGCTGGTGGGGTCTGTTTGGGTCTCTGTGTATTTGAAATGCCAGGGTCTAT
TTTGAAATTAGTGTCAACAAAACTCTTTGATAAAACAGAAAGAGTGAAATTTATTATTCTGAGGGAGAAATTG
TTATGTGAAACTTAACAATGTCTGTAGACAAGCATTATGGTATAATTGTTGTAAGTGTGAATAGGGTAATGTA
ATTTTATGAGTTAGTACTATGATATCAATGGAGTTAGCTGAATGTTGGCTGTAAGAGAGTTTGGACAACGCTA
TATGATTGAAGTGAAAGCCTTTTAAAGACTACAAGTTAATACACAATTCTGCTGAGGGACACAGTTGTTTACA
AGAAGTCACTGTTAGACTTGATAGTATCATCCCAAGTGATTTGAATGCCCTGGGTGATTTGTGGCCTTATGGG
TGGAATTTGTGGCTACTGGTGATAGACATATTTTGGAAAAGCATTGGCAGAATGTGTACTGAGGCTAAAACTC
TGCTGAAAGGAATTCAGTTGCCATTTGGTCTATAAGAGGGGGGTAAGGTAGAAACAAAGTGCATGGCAGATAC
ATAATATAGTAAACATATTGCTTATCAAAGAAGCATTGTTTAACTATGTGAGCTGTAAAGCCTGCAAGGTAAC
CTGCCCTTGGCCTAATTGTTTTGAGCAGCTGATGGTCTATAATGAAAATGCAGAGCAAGAGGGGTGCCTATAC
ATGTTTGCCTCATGTTTGCCCAAGATATGTATCTATAAGTAGACACAGATTTTCTCACTGGACAGAAATCATT
ATAGAGGTGTTTTATGACTTGGGATGTAGGTGAGATTTGTTTAATCTTTTGTTAAATCTAGTGTCCTTCAGTC
AGAATTACAGTCATTAATTGTTTATTTGTTTAGGGTAATGGGGACAACCAGTGAATGCATATATTGCATTGAA
TGCAGCATTGTATGTTATCTGTATTAGAGGACTTACCAATTTGTATACTGTTGGTTATTATTATCAAATCAGT
ATATTTTGTAAATCAAGGGGTGGAATGTAGCACAGTGGATTTTATTTTGATTTTATAATGATTTACAATATAC
ATCTGCTTTGACATATGAATCAAATCTTAGGTTTGAGTCAAGTTAAAGCAGCAGATTTTGGGACATCTATTGT
CCAGTGTTCAAGGTACAATGTCTTTGTTTTTTGAATATTGCTGGGTAAACAGTAAACACAGGTATTGTTACCC
AGCAAATATCCTTGGAATAAACAAACAGACAGCATATAGACAATACACTTTAGGTACAACTTATTTGAGCAAC
AAATAAGGCCTTCTACAAAGTCTAGTTAATAGATTTTGTTCATGACAAAAGTTTGTACACAAGTCAGCACTTG
GTGTTGGGGGTTTTGCTAAGAACTCTAGTATAAAAAGGAGCCTCCACCATGGGTCAGTAGCTTCGCCTAGGAC
TCCTGAAAGAGTGCCGGGACAATTGGATCATCGCTTGGTTATCGGGAACCTGAAGGTCTTGCGCCAAAGGTTG
TGGGGCTCCCCGATTGTGCTGAATTGTGCAGAACTGGCTATACTTGTAACTAATCATACTAAGATAAGTAAAT
CTATTTAATTCTATTATTACTTGTGTGTGTGTAAGTTATTGCTCACTAACAGTCTATCAGAAGGTTTAATCAT
TGATCCTGCATATTTATTAATATTTGTCATTAATGTCAATTAATACAAATTATTAATATTAATAAAGGTTCAC
CCCCTTTATTACA

>Xt-S13_CM004444.2:89086660-89090623_ Xenopus tropicalis strain Nigerian chromosome 2, whole genome shotgun sequence
TGTAGCACGGTGGATTTTCTATTGATTTTATGATTTACAATATATACATCTGCTTTGACATATGAATCAAATC
TTAGGTTTGAGTCAAGTAAAAGCAGCAGATTTTGGGACAGCTATTGTCTAGTGTTCAAGGTACAATGTTTTTG
TTCTATGTATATTGCTGGGTAAACAGTAAACACAGGTATTGTTACCCAGCAAATATCCTTGGAATAAACAAAC
AGACAGCATATAGACAATACACTTTAGGTACAACTTATTTGAGCAACAAATAAGGCCTTCTACAAAGTCTAGT

TAATAGATTTTGTTCATGACAAAAGTTTGTACACAAGTCAGCACTTGGTGTTGGGGGTTTTGCTAAGAACTCT
AGTATAAAAAGGAGCCTCCACCATGGGTCAGTAGCTTCGCCTAGGACTCCTGAACGAGTGCCGGGACAATTGG
ATCATCGCTTGGTTATCGGGAACCTGAAGGTCTTGCGCCAAAGGTTGTGGGGCTCCCCGATTGTGCTGAATTG
TGCAGAACTGGCTATACTTGTAACTAATCATACTAAGATAAGTAAATCTCTTTGATTCTATTATTACTTGTGT
GTGTGTAAGTTATTGCTCACTAACAGTCTATCAGAAGGTTTAATCATTGATCCTGCATATTTATTAATATTTG
TCATTAATGTCAATTAATACAAATTATTAATATTAATAAAGGTTCACCCCCTTTATTACATTTGGCGTAGTCG
GCAGGATTAACCCTTTTGATGCTGGGAAGTGCAGTAATAACTTGTATAGAAATTGTAGAATTTATAGTGCAGC
TTTGTATGGTAGTGTAGTGGCAGTATTGTAGTGGCTTGTGCAGGGCAAACATACTGTTGAAAGTTAGCAATAA
TGTTCTGTTGGTTGAAAAACAAAGTTAGAGGGGTAGATGATCAAACGAGCAACCTTCCCACATGGGCTAGTTC
CGGACCCTGGACCCATGTTGCTCGGGATGTGATAGGAAATCAGCAACTCTCTCAGGTGACTCACCCCCATCAC
CTGTTGCTGAATTTGGATTTGTCTATGTGTTCCAAGGAGCTGAAGTCCTCAGCACAATTGGGTTGGTTGTGTT
TTCAGGCGCTCAGGGAGGAGACAGTCCAAAGGGAGGCAGTTGAGCTTGAGCTGCAGGCAGTTAAAGATCAACT
AGCCACAATGAGAGAATGTTTGCGCTCCTCATTAGCACACAATGAGGAGTTGGAGATAGAATTTGCAAAATAT
GTGGTAAGAACTATGAGTAAAAAGCACCATAGAAAAGGGACAGAACAAGCAGAAAATGGTGTATTAGTTAAAG
TAAGAACTCTTGTAAGTAAAAGAGACTAAGGGAGTGATTTATTTCTCAAGATGGAGAAACAAAATAAATGTTA
CATTGTGTTAAATTGTACTGTATGTTAATGTCATATGTTTTTCTGTTTCAGGATTTAAGAATCTGATTATTTT
GAGTTAATATGGTTTTTGATAACAGGCATCAAGAGTTGATGGATCCATTATTTTTGACACTTTTTGTTTTGTT
TTTTAAGTGCACAAATTTGTGCGTAAAGGCCTTAAGACATGTCTGTTTTTGTTAAAAAAAAAAAATGTAACTA
TTAAACTTGAAAGTTTTTAACACTTTTTGTTGTATGTTTTTGAGAATGTGTGGGCCCACATGCATGTTGTTAT
GGTTTGTCTTGTCTTAATTGTGACAATGACTTAAATGTGACAGTGACTGAAAAATTCTTGACAAAGTAATACA
AAATTAAGAAAGTTGTTAAAGTTGACAAAACAGATCTTAAATGCTAAGAACTGATGTTGCCTTGATCTTTTGT
TTTATAGATGTTTATCAAGTGCATTAACAAATGAGTTAATAGCAAAACAATTGACAGCAAACAAGGATTTGGA
CATTGTTAATTTTGAATGGACATCAGTAACAAAGTCAACAAACGAAGTATTGAAGAACATGGAATTCATTTGA
ATGGTGACCTGGAAACTTAAATGGACATTGGTTTTGGACTTTATAAATTAATACTTATGTTTTGTTCAACTGT
TTCTACAGGCTGAAATTAGCATTGCATACATTATTGTACTAATGGTAATAGGAATGTAATGGCTTGTTACATG
AACTGTAAAGTGCTAGTTAGTGGGTTTTATAGCACAGGCAGCACAGCTGAACTGTATATCTCCTATGGTATAT
AATATTGTGCAGTGAACCTGCAGTATAGAGCAAGCCACTGGCAATCTGTGAATTTAGGTAAATGCCAGAGGGG
CTGCTGTAAGATACCACAGACACTCACTATTTATTGGCCGGGTGGGGGCTGTTTGGGGCTCTGTGTACTTGAA
ATATCAGGGCCTATTTTGATAAAACAGAAAGAGTGAAATTTATTATTCTGAGGGAGAAATTATTATGTGAAAC
TGACAATGTCTGTAGACAAGTATTATGGTATAAGTAATTATTGTAAGTGTGAATACGGTAATGTTATTTTGTG
AGTTAGTACTGTGATGTCAATGGGGTTGGCTGAAGGAGAGTATAGACAACTCTATATGATTGAAGTTAAAGAC
TACAACTTAATATACAATTCTGTTGAGGGACACAGTAGTTTATAAGAAGTCACTGTTAGACTTGATAGTATCA
TCCCAAGTGATTTGAATGCCCTGTGTGATTTGTGGCCTTATGGGTGGAATTTGTGGCTACTGGTGATAGACAT
ATTTTGGAAAAGCATTGGCAGAATGTGTACTGAGGCTAAAACTCTGCTGAAAGGAATTCAGTTGCCATTTGGT
CTATAAGAGGGGGGTTAGGTAGAAACAAAGTGCATGGCAGATACATAATATAGTAAACATATTGCTTATCAAA
GAAGCACTGTTTAACTATGTGAGCTGTAAAGCCTGCAATGAAACCTGCCCTTGGCCTAATTGTTTTGAGCAGC
TGATAGTCTGTAATGAAAATGCAGAGCAAGAGGGATTCCTATACATGTTTGCCACATGATTGCCCATGATATG
TATCTACAAGTAGACACAGATTTTCTCACTGGACAGAAATCATTATAGAGATGTTTTATGACTTGGGATGTAG
GTGAGATTTGTTTAATCTTTTGTTAAATGTAGTGTCCTTCAGTCAGAATTACAGTCATTAATTGTTTATTTGT
TTAGGGTAATGGGGACAACCAGTGAATGCATATATTGCATTGAATGCAGCATTGTATGTTATCTGTATTATTA
TCAAATCAGTATATTTTGTAAATCAAGGGGTGGAATGTAGCACGGTGGATTTTCTATTGATTTTATGATTTAC
AATATATACATCTGCTTTGACATATGAATCAAATCTTAGGTTTGAGTCAAGTAAAAGCAGCAGATTTTGGGAC
AGCTATTGTCTAGTGTTCAAGGTACAATGTTTTTGTTCTATGTATATTGCTGGGTAAACAGTAAACACAGGTA
TTGTTACCCAGCAAATATCCTTGGAATAAACAAACAGACAGCATATAGACAATACACTTTAGGTACAACTTAT
TTGAGCAACAAATAAGGCCTTCTACAAAGTCTAGTTAATAGATTTTGTTCATGACAAAAGTTTGTACACAAGT
CAGCACTTGGTGTTGGGGGTTTTGCTAAGAACTCTAGTATAAAAAGGAGCCTCCACCATGGGTCAGTAGCTTC
GCCTAGGACTCCTGAACGAGTGCCGGGACAATTGGATCATCGCTTGGTTATCGGGAACCTGAAGGTCTTGCGC
CAAAGGTTGTGGGGCTCCCCGATTGTGCTGAATTGTGCAGAACTGGCTATACTTGTAACTAATCATACTAAGA
TAAGTAAATCTCTTTGATTCTATTATTACTTGTGTGTGTGTAAGTTATTGCTCACTAACAGTCTATCAGAAGG
TTTAATCATTGATCCTGCATATTTATTAATATTTGTCATTAATGTCAATTAATACAAATTATTAATATTAATA
AAGGTTCACCCCCTTTATTACA

>Xt-S14_CM004452.2:20863848-20859955_ Xenopus tropicalis strain Nigerian chromosome 10, whole genome shotgun sequence

TGTAGCACGGTGGATTTTATTTTGATTTTATAATGATTTACAATATACATCTGCTTTGACATATGAATCAAAT
CTTAGGTTTGAGTCAAGTAAAAGCAGCAGATTTTGGGACAGCTATTGTCCAGTGTTCAAGGTATATTGCTGGG
TAAACAGTAAACACAGGTATTGTTACCCAGCAAATATCCTTGGAATAAACAAACAGACAGCATATAGACAATA
CACTTTATGTACAACTTATTTGAGCAACAAATAAGGCCTTCTACAAAGTCTAGTTAATAGGTTTTGTTCATGA
CAAAAGTTTGTACACAAGTCAGCACTTGGTGTTGGGGGTTTTGCTAAGAACTCTAGTATAAAAGGGAGCCTCC
ACCATGGGTCAGTAGCTTCGCCTAGGACTCCTGAACGAGTGCCGGGACATTTGGATCATCGCTTGGTTATCGG
GAACCTGAAGGTCTTGCGCCAAAGGTTGTGGGGCTCCCCAATTGTGCTGAATTGTGCAGAACTGGCTACACTT
GTAACTATAATCATACCAAGATAAGTAAATCTATTTGATTCTATTATTACTTGTGTGTTATTGCTCACTAACA
GTTTATCAGAAGGCTTAATCATTGATCCTGCATATTTATTAATATTTGTCATTAATGTCAATTAATTATTAAT
ATTAATAAAGGTTCACCCCCTTTATTACATTTGGCGTAGTCGGCAGGATTAACCCTTTTGATGCTGGGAAGTG
CTGTAATAAATTGTATAGAAATTGTAGAATTTATAGTGCAGCTTTGTATGGTAGCGTAGTGGCAGTATTGTAG
TGGCTTGTGCAGGGCAAACATACTGTTGAAAGTTAGCAATAATGTTCTGTTGGTTGAAAAACAAAGTTAGAGG
GATAGATGATCAAACGAGCAACCTTCCCACATGGGCTAGTTCCGGACCCTGGACCCATGTTGCTCGGGATGTG
ATAGGAGATCAGCAACTCTCTCAGGTGACTCACCCCGATCACCTGTTGCTGAATTTGGATTTGTCTATGTGTT
CCAAGGAGCAGAAGTCCTCAGCACAATTGGGTTGGTTGTGTTTTCAGGCGCTCAGGGAGGAGACAGTCCAAAG
GGAGGCAGTTGAGCTTGAGCTGCAGGCAGTTAAAGATCAACTAGCCACAATGAGAGAATGTTTGCGCTCCTCA
TTAGCACACAATGAGGAGTTGGAGATAGAATTTACAAAATATGTGAGCAGAAAATGGTGTATTAGTTAAAGTA
AGAACTCTTGTAAGTAAAAGAGACTAAGGGAGTGATTTATTTCTCAAGATAGAGAAACAAAATAAATGTTACA
TTTTGTTTAATTGTACTGTATGCTAATGTCATATGTTTTTCTGTTTCAGGATCTAAGAATCTGATTATTTTGA
GTTAATATGGTTTTTAATTACAGACATCAAGAGTTGAAGGATCCATTATTCTTGACAAATTTTTGTTCTGTTT
TTAGTGCACAGATTTCTGTGTAAAGGCCTTAAGACATGTCTGTTTTTGTTAAAAGAAAGTTTAACAATGAAAC
TTGAAAGTTTTTAACACTTTTTGTTGTATGTTTCTGAGAATGTGTGGGCCCACATGCATGTTGTTATGGTTTG
TCTTGTCTTAATTGTGACAATGACTTAAATGTGACAGTGACTGAAAAATTCTTGACAAAGTAATACAAAATTA
AGAAAGTTGTTAAAAGTTGACAAAAACAGATCTTAAATGCTAAGAACTGATGTTGCCTTGATCTTTTGTTTTA
TAGATGTTTATCAAGTGCATTAATAAATGGGTTAATAGCAAAACAATTGACAGCAAACAAGGATTTGGACATT
GTTAATCTTGAATGGACATCAGTACAAAGTATTGAAGAACCTGGAATTCAGTTGAATGGTGACTTGGAAACTT
AAATGGACATTGGTTTTGGACTTTATAAATGAATACTTATGTTTTGTTCAACTGCTTCTACAGACTGAAATTA
GCATTGCATACACTATTGTACTAATGGTAATAGGAATGTTATGGCTTGTTACATGAACTGTAAAGTGCTAGTT
AGTGGGTTTTATAGCACAGGCAGCACAGCTGAACTGTATATCTCCTATGGTATATAATATTGTGCAGTGAACC
TACAGTGTAGAGCAGGCCCTGGCTGACAATCTGTAGATTCAGGCAAATGCCAGAGGTGCTGATGTAAGATGCC
ATAGACAGTCACTATTTATTGGGCTGGGGGGGGGCTGTTTGGGTCTCTGTGTATTTGAAATGCCAGGGCCTAT
TTTGAAATTAGTGTCAACGAAACTCTTTGATAAAACAGAAAGAGTGAGGGAGAAATTGTTATGTGAAACTAAC
AATGTCTGTAGACAAGTATTATGGTATAAGTAATTATTGTAAGTGTGAATAGGGTAATGTTATTTTGTGAGTT
AGTACTGTGATGTCAATGGGGTTGGCTGAAGGAGAGTTTAGACAACTCTATATGATTGAAGTTAAAGACTACA
ACTTAATATACAATTCTGTTGAGGGACACAATAGTTTATAAGAAGTCACTGTTAGACTTGATAGTATCATCCC
AAGTGATTTGAATGCCCTGGGTGATTTGTGGCCTTATGGGTGGAATTTGTGGTTACTGGTGATAGACATATTT
TGGAAAAGCATTGGCAGAATGTGTATTGAGACTAAAATTCTGCTGAAATGAATTCAGTTGCCATTTGGTCTAT
AAGAGGGAGGTAAGGTAGAAACAAAGTGCATGGCAGATACATAATATAGTAAACATATTGCTTATCAAAGAAG
CACTGTTTAACTATGTGAGCTGTAAAGCCTGCAATGAAACCTGCCCTTGGCCTAATTGTTTTGAGCAGCTGAT
AGCCTATAATGAAAATGCAGAGCAAGAGGGGTGCCTATACATGTTTGCCACATGATTGCCCAAGATATGTATC
TACAAGTGGACACAGATTTTTCTCACTGGACAGAAATCATTATAGAGGTGTTTTATGACTTGGGATGTAGGTG
AGATTTGTTTAATCTTTTGTTAAATGTAGTGTCCTTCAGTCAGAATTTCAGTCATTAATTGTTTATTTGTTTA
GGGTAATGACAACCAGTAAATGCATATATTGCATTGAATGCAGCATTTTATGTTATCTGTATTAGAGGACTTA
CCAGTTTGTATACTGTTGGTTATTATTATCAAATCAGTATATTTTGTAAATCAAGGGGTGGAATGTAGCACGG
TGGATTTTATTTTGATTTTATAATGATTTACAATATACATCTGCTTTGACATATGAATCAAATCTTAGGTTTG
AGTCAAGTAAAAGCAGCAGATTTTGGGACAGCTATTGTGCAGTGTTCAAGGTTTATTGCTGGGTAAACAGTAA
ACACAGGTATTGTTACCCAGCAAATATCCTTGGAATAAACAAACAGACAGCATATAGACAATACACTTTATGT
ACAACTTATTTGAGCAACAAATAAGGCCTTCTACAAAGTCTAGTTAATAGGTTTTGTTCATGACAAAAGTTTG
TACACAAGTCAGCACTTGGTGTTGGGGGTTTTGCTAAGAACTCTAGTATAAAAGGGAGCCTCCACCATGGGTC
AGTAGCTTCGCCTAGGACTCCTGAACGAGTGCCGGGACATTTGGATCATCGCTTGGTTATCGGGAACCTGAAG
GTCTTGCGCCAAAGGTTGTGGGGCTCTCCAATTGTGCTGAATTGTGCAGAACTGGCTACACTTGTAACTATAA
TCATACCAAGATAAGTAAATCTATTTGATTCTATTATTACTTGTGTGTTATTGCTCACTAACAGTTTATCAGA
AGGCTTAATCATTGATCCTGCATATTTATTAATATTTGTCATTAATGTCAATTAATACAAATTATTAATATTA
ATAAAGGTTCACCCCCTTTATTACA

>Xt-S15_CM004451.2:15854700-15850673_ Xenopus tropicalis strain Nigerian chromosome 9, whole genome shotgun sequence
TGTAGCACGGTGGATTTTCTATTGATTTTATGATTTACAATATATATACATCTGCTTTGACATATGAATCAAA
TCTTAGGTTTGAGTCAAGTAAAAGCAGCAGATTTTGGGACAGCTATTGTCTAGTGTTCAAGGTATAATGTCTT
TGTTCTATGTATATTGCTGGGTAAACAGTAAACACAGGTATTGTTACCCAGCAAATATCCTTGGAATAAACAA
ACAGACAGCATATAGACAATACACTTTATGTACAACTTATTTGAGCAACAAATAAGGCCTTCTAAGTCTAGTT
AATAGGTTTTGTTCATGACAAAAGTTTGTACACAAGTCAGCACTTGGTGTTGGGGGTTTGCTAAGAACTCTAC
TATAAAAAGGAGCCTCCACCATGGGTCGGTAGCTTCGCCTAGGACTCCTGAACGAGTGCCGGGACATTTGGAT
CATCGCTTGGTTATCGGGAACCTGAAGGTCTTGCGCCAAAGGTTGTGGGGCTCCCCGATTGTGCTGAATTGTG
CAGAACTGGCTATACTTGTAACTAATCATACTAAGATAAGTAAATCTATTTGATTCTATTATTACTTGTGTGT
GTGTAAGTTATTGCTCACTAAAAGTCTATCAGAAGGTTTAATCATTGATCCTGCATATTTATTAATATTTGTC
ATTAATGTCAATTAATACAAATTATTAATATTAATAAAGGTTCACCCCCTTTTTTACATTTGGCGTAGTTGGC
AGGATTAACCCTTTTGATGCTGGGAAGTGCAGTAATAACTTGTATAGAAATTGTAGAATTTATAGTGCAGCTT
TGTATGGTAGTGTAGTGGCAGTATTGTAGTGGCTTGTGCAGGGCAAACATACTGTTGAAAGTTAGCAATAATG
TTCTGTTGGTTGAAAAACAAAATTAGAGGGGTAGATGATCAAACGAGCAACCTTCCCACATGGGCTAGTTCCG
GACCCTGGACCCATGTTGCTCGGGATGTGATAGGAGATCAGCAACTCTCTCAGGTGACTCACCCCCATCACCT
GTTGCTGAATTTGGATTTGTCTATGTGTTCCAAGGAGCTGAAGTCCTCAGCACAATTGGGTTGGTTGTGTTTT
CAGGCGCTCAGGGAGGAGACAGTCCAAAGGGAGGCAGTTGAGCTTGAGCTGCAGGCAGTTAAAGATCAACTAG
CCACAATGAGAGAATGTTTGCTCTCCTCATTAGCACACAATGAGGAGTTGGAGATAGAATTTACAAAATATGT
GGTAAGAACTATGAGTAAAAAGCACCATAGAAAAGGGACAGAACAAGCAGAAAATGGTGCATTAGTTAGAATA
AGAACTCTTGTAAGTAAAAGAGACTAAGGGAGTGATTTATTTCTCAAGATAGAGAAACAAAATAAATGTTACA
TTGTGTTAAATTGTACTGTATGTTAATGTCATATGTTTTTCTGTTTCAGGATTTAAGAATCTGATTATTTTGA
GTTAATATGGTTTTTGATAACAGGCATCAAGAGTTGATGGATCCATTATTTTTGACACTTTTTGTTTTGTTTT
TTAAGTGCACAAATTTGTGCGTAAAGGCCTTAAGACATGTCTGTTTTTGTTAAAAAAAAGTTTAACAATGAAA
CTTGAAAGTTTTTAACACTTTTTGTTGTATGTTTCTGAGAATGTGTGGGCCCACATGCATGTTGTTGGTTTGT
CTTGTCTTAATTGTGACAATGACTTAAATGTGACAGTGACTGAAAAATTCTTGACAAAGTAATACAAAATTAA
GAAAGTTGATAAAGTTGACAAAACAGATCTTAAATGCTAAGAACTGATGTTGCCTTGATCTTTTGTTTTATAG
ATGTTTATCATGGATATAACAAGTGCATTAACAAATGGGTTAATAGCAAAACAATTGACAGCAAACAAGGATT
TGGACATTGTTAATCTTGAATGGACATCAGTACAAAGTATTGAAGAACCTGGAATTCATTTGAATGGTGACCT
GGAAACTTAAATGGACATTGGTTTTGGACTTTATAAATGAATACTTATGTTTTGTTCAACTGTTTCTACAGAC
TGAAATTAGCATTGCATACACTATTGTACAAAAGGTAATAGGAATGTTATGGCTTGTTACATTCACTGTAAAG
TGCTAGTTAGTGGGTTTTATAGCACAGGCAGCACAGCTGAACTGTATATCTCCTATGGTATATAATATTGTGC
AGTGAACCTGCAGTATAGAGCAGGCCCTGGCTGACAATCTGTAGATTCAGGCAAATGCCAGAGGGGCTGCTGT
AAGGTGCCATAGACAGTCACTATCTATTGGGCTGGTGGGGGCTGTTTGGGTCTCTGTATTTGAAATGCCAGGG
TCTATTTTGAAATTGGTGTCAACGAAACTCTTTGATAAAACAGAAAGAGTGAAATATATTATTCTGAGGGAGA
AATTGTTATGTGAAACTAACAATGTCTGTAGACAAGCATTATGGTATAATTGTTGTAAGTGTGAATAGAGTAA
TGTAATTTTATGAGTTAGTACTATGATATCAATGGAATTAGCTGAATGTTGGCTGTAAGAGAGTTTGGACAAC
TCTATATGATTGAAGCAAAAGCCTTTTAAAGACTACAAGTTAATACACAATTCTGCTGAGGGACACAGTTGTT
TATAAGAAGTCACTATTAGACTTGATAGTATCATCCCAAGTGATTTGAATGCCCTGGGTGATTTGTGGCCTTA
TGGGTGGAATTTGTGGATACTAGTGATAGACTGATATTTTGGAAAAGCATCGTCAGAATGTGTATTGAGGTTA
AAACTCTGCTGAAAGGAATTCAGTTGTCATTTGGTCTATAAGAAGGGGGTAAGAAACAAAGTGCATGGCAGAT
ACATAATATAGTAAACATATTGCTTATCAAAGAAGCACTGTTTAACTATGTGAGCTGTAAAGCCTGCAATGAA
ACCTGCCCTTGGCCTAATTGTTTTGAGCAGCTGATAGCCTATAATGAAAATGCAGAGTAAGAGGGATGCCTAT
ACATGTTTGCCACATGTTTGTCTAAGATATGTATCTACAAGTAGACACAGATTTTCTCACTGGACAGAAATCA
TTATAGAGGTGTTTTATGATTTGGGATGTAGGTGAGATTTGTTTAATCTTTTGTTAAATGTAGTGTCCTTCAG
TCAGAATTACAGTCATTAATTGTTTATTTGTTTAGGGTAATGGGGACAACCAGTGAATGCATATATTGCATTG
AATGCAGCGTTGTATGTTATCTGTATTAGAGGACTTACCAATTTGTATACTGTTGGTTACTATTATCAAATCA
AGTATATTTTGTAAATCAAGGGGTGGAATGTAGCACGGTGGATTTTCTATTGATTTTATGATTTACAATATAT
ATACATCTGCTTTGACATATGAATCAAATCTTAGGTTTGAGTCAAGTAAAAGCAGCAGATTTTGGGACAGCTA
TTGTCTAGTGTTCAAGGTATAATGTCTTTGTTCTATGTATATTGCTGGGTAAACAGTAAACACAGGTATTGTT
ACCCAGCAAATATCCTTGGAATAAACAAACAGACAGCATATAGACAATACACTTTATGTACAACTTATTTGAG
CAACAAATAAGGCCTTCTAAGTCTAGTTAATAGGTTTTGTTCATGACAAAAGTTTGTACACAAGTCAGCACTT
GGTGTTGGGGGTTTGCTAAGAACTCTACTATAAAAAGGAGCCTCCACCATGGGTCGGTAGCTTCGCCTAGGAC

TCCTGAACGAGTGCCGGGACATTTGGATCATCGCTTGGTTATCGGGAACCTGAAGGTCTTGCGCCAAAGGTTG
TGGGGCTCCCCGATTGTGCTGAATTGTGCAGAACTGGCTATACTTGTAACTAATCATACTAAGATAAGTAAAT
CTATTTGATTCTATTATTACTTGTGTGTGTGTAAGTTATTGCTCACTAAAAGTCTATCAGAAGGTTTAATCAT
TGATCCTGCATATTTATTAATATTTGTCATTAATGTCAATTAATACAAATTATTAATATTAATAAAGGTTCAC
CCCCTTTTTTACA

>Xt-S16_CM004448.2:73921067-73917599 Xenopus tropicalis strain Nigerian
chromosome 6, whole genome shotgun sequence
TGTAGCACGGTGGATTTTCTATTGATTTTATGATTTACAATATATACATCTGCTTTGACATATGAATCAAATC
TTAGGTTTGAGTCAAGTAAAAGCAGCAGATTTTGGGACAGCTATTGTCTAGTGTTCAAGGTACAATGTTTTTG
TTCTATGTATATTGCTGGGTAAACAGTAAACACAGGTATTGTTACCCAGCAAATATCCTTGGAATAAACAAAC
AGACAGCATATAGACAATACACTTTATGTACAACTTATTTGAGCAACAAATAAGGCCTTCTAAGTCTAGTTAA
TAGGTTTTGTTCATGACAAAAGTTTGTACACAAGTCAGCACTTGGTGTTGGGGGTTTGCTAAGAACTCTACTA
TAAAAAGGAGCCTCCACCATGGGTCGGTAGCTTCGCCTAGGACTCCTGAACGAGTGCCGGGACAATTGGATCA
TCGCTTGGTTATCGGGAACCTGAAGGTCTTGCACCAAAGGTTGTGGGGCTCCCCGATTGTGCTGAATTGTGCA
GAACTGGCTATACTTGTAACTAATCATACTAAAGATAAGTAAATCTATTTAATTCTATTATTACTTGTGTGTG
TGTAAGTTATTGCTCACTAACAGTCTATCAGAAGGCTTAATCATTGATCCTGCATATTTATTAATATTTGTCA
TTAATGTCAATTAATACAAATTATTAATATTAATAAAGGTTCACCCCCTTTATTACATTTGGCGTAGTCGGCA
GGATTAACCCTTTTGATGCTGGGAAGTGCAGTAATAACTTGTATAGAAATTGTAGAATTTATAGTGCAGCTTT
GTATGGTAGTGTAGTGGCAGTATTGTAGTGGCTTGTGCAGGGCAAACATACTGTTGAAAGTTAGCAATAATGT
TCTGTTGGTTGAAAAACAAAGTTAGAGGGGTAGATGATCAAACGAGCAACCTTCCCACATGGGCTAGTTCCGG
ACCCTGGACCCATGTTGCTCGGGATGCGATAGGAGATCAGCAACTCTCTCAGGTGACTCACCCCGATCACCTG
TTGCTGAATTTGGATTTGTCTATGTGTTCCAAGGAGCAGATGTCCTCAGCACAGTTGGGTTGGTTGTGTTTTC
AGGCGCTCAGGGAGGAGACAATCCAAAAGGAGGCGGTTGAGCTTGAGCTGCAGGCAGTTAAAGATCAGCTAGC
CACAATGAGAGAATGTTTGGGCACCTCATTAGCACACAATGAGGAGATGGAGATAGAATTTACAAAAATATGT
GGTAAGAACTCTGAGTAAAAAGCACCATAGAAAAGGGACAGAACAAGAAGAAAATGGTGTATTAGTTAAAGTA
AGAACTCTTGTAAGTAAAAGAGACTAAGGGAGTGATTTATTTCTCAAGATAGAGAAACAAAATAAATGTTACA
TTGTGTTAAATTGTACTGTATGTTAATGTCATATGTTTTTCTGTTCCAGGATTTAAGAATCTGATTATTTTGA
GTTAATATGGATTTTAATAACAGGCATCAAGAGTTGATGGATCCATTATTTTTGACACTTTTTGTTTTGTTTT
TTTAAGTGCACAAATTTGTGCGTAAAGGCCTTAAGACAGGTCTGTTTTTGTTAAAAAAAAAGTTTAACAATGA
AACTTGAAAGTTTTTAACACTTTTTGTTGTATGTTTCTGAGAATGTGTGGGCCCACATGCATGTTGTTATGGT
TTGTCTTGTCTTAATTGTGACAATGACTTAAATGTGACAGTGACTGAAAAATTCTTGACAAAGTAATACAAAA
TTAAGAAAGTTGTTAAAGTTGACAAAACAGATCTTAAATGCTAAGAACTGATGTTGCCTTGATCTTTTGTTTT
ATAGATGTTTATCAAGTGCATTAACAAATGAGTTAATAGCAAAACAATTGACAGCAAACAAGGATTTGGACAT
TGTTAATTTTGAATGGACATCAGTAACAAAGTCAACAAACAAAGTATTGAAGAACATGGAATTCATTTGAATG
GTGACCTGGAAACTTAAATGGACATTGGTTTTGGACTTTATAAATTAATACTTATGTTTTGTTCAACTGTTTC
TACAGGCTGAAATTAGCATTGCATACATTATTGTACTAATGGTAATAGGAATGTAATGGCTTGTTACATGAAC
TGTAAAGTGCTAGTTAGTGGGTTTTATAGCACAGGCAGCACAGCTGAACTGTATATCTCCTATGGTATATAAT
ATTGTGCAGTGAACCTGCAGTATAGAGCAAGCCACTGGCAATCTGTGAATTTAGGTAAATGCCAGAGGGGCTG
CTGTAAGATACCACAGACACTCACTATTTATTGGCCGGGTGGGGGCTGTTTGGGGCTCTGTGTACTTGAAATA
TCAGGGCCTATTTTGAAAAAACAGAAAGAGTGTAATTTATCATCCTGAGGGAAAAATTGCTATGTGAAACTAA
CAATGTCTGTAGACAAGCATTATGGTATAATTGTTGTAAGTGTGAATAGAGTAATGTAATTTTATGAGTTAGT
ACTATGATATCAATGGAGTTAGCTGAATGTTGGCTGTAAGAGAGTTTGGACAACTCTATATGATTGAAGTGAA
AGCCTTTTAAAGACTACAAGTTAATACACAATTCTGCTGAGGGACACGTTAGTTTACAAGAAGTCACTGTTAG
ACTTGATAGTATCATCCCAAGTGATTTGAATGCCCTGGGTGATTTGTGGCCTTATGGGTGGAATTTGTGGCTA
CTGGTGATAGACATATTTTGGAAAAGCATTGGCAGAATGTGTACTGAGGCTACAATTCTGCTGAAAGGAATTC
AGTTGCCATTTGGTATATAAAAGGGGGTTAGGTAGAAACAAAGTGCATGGCAGATACATAATATAGTAAACAT
ATTGCTTATCAAAGAAGCACTGTTTAACTATGTGAGCTGTAAAGCCTGCAATGAAACCTGCCCTGGGCCTAAT
TGTTTTGAGCAGCTGATAGCCTATAATGAAAATGCAGAGCAAGAGGGGTGCCTATACATGTTTGCCACATGAT
TGCCCAAGATATGTATCTACAAGTAGACACAGATTTTCTCACTGGACAGAAATCATTATAGAGGTGTTTTATG
ACTTGGGATGTAGGTGAGATTTGTTTAATCTTTTGTTAAATGTAGTGTCCTTCAGTCAGAATTACAGTCATTA
ATTGTTTATTTGTTTAGGGTAATGGGGACAACCAGTGAATGCATATATTGCATTGAATGCAGCATTGTATGTT
ATCTGTATTAGAGGACTTACCAATTTGTATACTGTTGGTTATTATTATCAAATCAGTATATTTTGTAAATCAA
GGGGTGGAATGTAGCACGGTGGATTTTCTATTGATTTTATGATTTACAATATATACATCTGCTTTGACATATG

AATCAAATCTTAGGTTTGAGTCAAGTAAAAGCAGCAGATTTTGGGACAGCTATTGTCTAGTGTTCAAGGTACA
ATATTTTTGTTCTATGTATATTGCTGGGTAAACAGTAA

>Xt-S17_CM004448.2:c1899534-1894643 Xenopus tropicalis strain Nigerian chromosome 6, whole genome shotgun sequence
GATTTTCTATGATTTACAATATATACATCTGCTTTGACATATGAATCAAATCTTAGGTTTGAGTCAAGTAAAA
GCAGCAGATTTTGGGACAGCGATTGTCCAGTGTTCAAGGTACAATGTCTTTGTTCTATGTATATTGCTGGGTA
AACAGTAAACACAGGTATTGTTACCCAGCAAATATCCTTGGAATAAACAGACAGACAGCATATAGACAATACA
TTTTATGTACAACTTATTTGAGAAACAAATAAGGCTTTCTACAAAGTTAGTAAATTGTCTAGTTAGGTTTTGT
TCATGACAAAAGTTTGTACACAATATTGCACAATAAGTCAGCACTTGGTGTTGGGGGTTTGCAAAGACCTCTG
GTATAAAAGGGAGCCTCCACCATGGGTCAGTAGCTTCGCCTAGGACTCCTGAATGAGTGCTGGGACATTTGGA
TCATCGCTTGGTTATCGGGAACCTGAAGGTCTTGCGCCAAAGGTTGTGGGGCTCCCCGATTGTGCTGAATTGT
GCAGAACTGGCTACACTTGTAACTATAATCATACCAAGATAAGTAAATCTATTTGATTCTATTATTACTTGTG
TGTGTGTAAGTTATTGCTCACTAATAGTCTATCAGAAGGTTTAATCATTGATCCTGCATATGTATTAATATTT
GTTATTAATGTCAATTAATACAAATTATTAATATTAATAAAGGTTCACCCCCCTTTATTACATTTGGCGTAGTC
GGCAGGATTAACCTTTTTGATGCTGGGAAGTGCAGTAATAACTTGTATAGAAATTGTAGAATTTATAGTGCAG
CTTTGTATGGTAGTGTAGTGGCAGTATTGTAGTGGCTTGTGCAGGGCAAACATACTGTTGAAAGTTAGCAATA
ATGTTCTGTTGGTTGAAAAACAAAATTAGAGGGGTAGATGATCAAACGAGCAACCTTCCCACATGGGCTAGTT
CCGGACCCTGGACCCATGTTGCTCGGGATGTGATAGGAGATCAGCAACTCTCTCAGGTGACTCACCCCGATCA
CCTGTTGCTGAATTTGGATTTGTCTATGTGTTCCAAGGAGCAGAAGTCCTCAGCACAATTGGGTTGGTTGTGT
TTTCAGGCGCTTAGGGAGGAGGCAGTCCAAAGGGAGGCAGTTGAGCTTGAGCTGCAGGCAGTTAAAGATCCAC
TAGCCACAATGAGAGAATGTTTGCGCTCCTCATTAGCACACAATGAGGAGTTGGAGATAGAATTTACAAAATA
TGTGGTAAAAACTATGAGTAACAATCACCATAGAAAAGAGACAGAACAAGAAGAAAATGGTGCATTAGTTAGA
ATAAGAGCTCTTGTAAGTAAAGGAGACTGGGGGGAGTTAGCCCAGTGGTCAGGAGCACATGAGGAAGTTCAAG
AAGACATGATAAGACCTCTTGTGACTACAAGACAAGTCAGTCAAAGTGCAGATATTGTTGACAGATCTTCAGG
AAATACAGAAGCAGCAGATCAACAGTACATTGAGCAACCAAGTTTATCTGCTGCAGTAGTTAAGTTGGGTCAG
CCAATTTCCCAGGTAGGAGGAGAAAGTGGTGTGCTTACAGATCCATTAACTTCAGTACTCCCAAACCCACATA
CAGTAAAAGAAGAATGGGAAAGGCCAGACAAAGTTCCATTTACTGCACATATGAAGAATACATTGCTAGTAGG
ATCTCCCACCCATTTAAAAGCCCCATTGTTACATAAAACTGACATATTGTTGGGCAGATTAGAATGTGCAGAG
GAAAACATTCCTAAGCAAAGGGTAGTAAAAGTCTGGGGTACAAAAAGGTTAAAAAACAGTCGCAGGCAGATGT
TTTTAGATTTGCTTCAATATGGTGTACCAAAGCCTGAAATTGATGGTATTCCCACTGCTGATTTGTTGAAGCG
CTGGAAAAAACTGAGACAAAAGGTGAGGTTGAAATTACCACAACCACAGAATGTGGCAACCTGTAATGTAGAG
GACCTATCTTCCCTTTTAGCCCATACATAGAAAAGGAAATGGCTAAACATAAGGGGAACATCTAGGAGGTTAT
ATGAGAGGATAACTAGTGATAGTTTAGGAAAAGTACAGAAGCAAAACAATGTTGAGAATGGTATAATTCTACT
GGTTTAGAAATAGAACAGCTGTATATTGTAAAATGTGTTTCTCTTTGTATGTAAAGAATTGCTGCCCCCTCCC
TTTAGACACAGACTAGTTCAACTGAGGGAGTGATTTATTTCTCAAGATAGAGAAACAAAATAAATGTTACATT
GTGTTTAATTTTACTGTATGTTAATGTCATATGTTTTTCTGTTTCAGGATTTAAGAATCTGATTATTTTGAGT
TAATATGGTTTTTAATTACAGGCATCAAGATTTGATCAAGAGTTGATTTACGCACAAATTTGTGCGTAAAGGC
CTTAAGACATTCCTATTTTGTTAAAAAGTTTTTTTAAAACTGTAGTACTACAATTAACAATGAAACTTGAAA
GTTTTTAACACTTTTTTTGTTGTATGTTTCTGAGAATGTGTGGGCCCACATGCATGTTGTTATGGTTTGTCTT
GTCTTAATTGTGACAATGACTTAAATGTGACAGTGACTGAAAAAATCTTGACAAAATAATAGAAAATTAAGAA
ACTTGATAAAGTTGACAAAACAGATGTTAAATGCTAACAACTGATGTTGCCTTGATATTTTGTTTTATAGATG
TTTATCAAGTGCATTAACAAATGGGTTAATAGCAAAACAATGGACAGCAAACAAGGATTTGGACATTGTTTAT
CTTCAATGGACATCAGTACAAAGTATTGAAGAACCTGGAATTCATTTGAATGGTGACCTGGAAACTTAAATGG
ACATTGGTTTTGGACTTTATAAATGAATATTTATGTTTTGTTCAATTGTTTCTACTGGCTGAAATTAGCATTG
CATACACTATTGTACTAATGGTAATAGGAATGTTATGGCTTGTTACATGAACTGTAAAGTGCTAGTTAGTGGG
TTTTAGAGCACAGCTGAACTGTATATCTCCTATGGTATACAGTATAATATTGTGCAGTGAACCTGCAGTATAG
AGCAGGCCCTGGCTGACAATCTGTAGATTCTGGCAAATGCCAGAGGGGCTGATGTAAGATGCTATAGACAGTC
ACTATTTATTGGGATTGTGGGAGCTGTTTGGGTCACTGTGTATTTGAAATGCCAGGGTCTATTTTGAAATTAG
AGTCTATGAAACTCTTTGATAAAACACTCTTTGAGTGAAATTTATTATTCTGAGGGAGAAATTGTTATGTGAA
ACTAACAATGTCTGTAGACAAGTACTGTATTATGGTATAAGTAATTATTGTAAGTGTGAATACGGTAATGTCA
TTTTGTGAGTTAGTACTGTGATATCAATGGGGTTGGCTGAAGGAGAGTTTGGACAACTCTATATGAAGTTAAA
GACTACAACTTAATATACAATTCTGTTGAAGGACACAGTAGTTTATAAGAAGTCACTGTTAGACTTGATAGTA
TCATCCCAAGTGATTTGAATGCCCTGGGTGATTTGTGGCCTTATGGGTGGAATTTGTGGCTACTAGTGATAGA

CTGATATTTTGGAAAAGCATTGGCAGAATGTGTATTGGGACTAAAACTCTGCTGAAAGGAATTCAGTTGCCAT
TTGGTCTATAAGAGGGGGGTTAGGTAGAAACACAGTGCATGGCAGATACATAATATAGTAAACATATTGCTTA
TCAAAGAAGCACTGTTTAACTATGTGAGCTGTAAAGCCTGCAATGAAACCTGCCCTTGGCCTGATTGTTTTGA
GCAGCTGATAGATTATAATGAAAATGCAGAGCAAGAGGGATTCCTATACATGTTTGCAACATGTTTGCCCAAG
ATATGTGTCTACAAGTAGACACAGATTTTCTCACTGGACAGAAAGCATTATAGAGATGTTTTATGACTTGGGA
TGTAGGTGAGATTTGTTTAATCTTTTGTTAAATGTAGTGTCCTTCAGTCAGAATTACAGTAATTAATTGTTTA
TTTGTTTAGGGTAATGGGGACAACCAGCGAATGCATATATTGCATTGAACACAGCATTGTATGTTATCTGTAT
TAGAGGACTTACCAATTTGTAAACTGTTGGTTATTATTATCAAATTAGTATATTTTGTAAATCAAGGGGTGGA
ATGTAGCACGGTGGATTTTCTATGATTTACAATATATACATCTGCTTTGACATATGAATCAAATCTTAGGTTT
GAGTCAAGTAAAAGCAGCAGATTTTGGGACAGCTATTGTCCAGTGTTCAAGGTACAATGTCTTTGTTCTATGT
ATATTGCTGGGTAAACAGTAAACACAGGTATTGTTACCCAGCAAATATCCTTGGAATAAACAGACAGACAGCA
TATAGACAATACATTTTATTTACAACTTATTTGAGAAACAAATAAGGCTTTCTACAAAGTTAGTAAAGTGTCT
AGTTAGGTTTTGTTCATGACAAAAGTTTGTACACAATATTGCACAATAAGTCAGCACTTGGTGTTGGGGGTTT
GCAAAGACCTCTGGTATAAAAGGGAGCCTCCACCATGGGTCAGTAGCTTCGCCTAGGACTCCTGAATGAGTGC
CGGGACATTTGGATCATCGCTTGGTTATCGGGAACCTGAAGGTCTTGCGCCAAAGGTTGTGGGGCTCCCCGAT
TGTGCTGAATTGTGCAGAACTGGCTATACTTGTAACTATAATCAAACAAAGTTAAGTAAATCTATTTAATTCT
ATTATTACTTGTGTGTGTGTAAGTTATTGCTCATTAATAGTCTATCAGAAGGTTTAATCATTGATCCTGCATA
TGTATTAATATTTGTTATTAATGTCAATTAATACAAATTATTAATATTAATAAAGGTTCACCCCCTTTATTAC
A

>Xt-S18_CM004449.2:c61019258-61015158 Xenopus tropicalis strain Nigerian chromosome 7, whole genome shotgun sequence
TGTAGCACGGTGGATTTTCTATTGATTTTATGATTTACAATATATACATCTGCTTTGACATATGAATCAAATC
TTAGGTTTGAGTCAAGTAAAAGCAGCAGATTTTGGGACAGCTATTGTCTAGTGTTCAAGGTACAATGTCTTTG
TTCTATGTATATTGCTGGGTAAACAGTAAACACAGGTATTGTTACCCAGCAAATATCCTTGGAATAAACAAAC
AGACAGCATATAGACAATACACTTTATGTACAACTTATTTGAGCAACAAATAAGGCCTTCTAAGTCTAGTTAA
TAGGTTTTGTTCATGACAAAAGTTTGTACACAAGTCAGCACTTGGTGTTGGGGGTTTGCTAAGAACTCTACTA
TAAAAAGGAGCCTCCACCATGGGTCGGGAGCTTCGCCTAGGACTCCTGAACGAGTGCCGGGACATTTGGATCA
TCGCTGGGTTATCGGGAACCTGAAGGTCTTGCGCCAAAGGTTGTGGGGCTCCCTGATTGTGCTGAATTGTGCA
GAACTGGCTATACTTGTAACTAATCATACTAAGATAAGTAAATCTATTTAATTCTATTATTACTTGTGTGTAA
GTTATTGCTCACTAACAGTCTATCAGAAGGTTTAATCATTGATCCTGCATATTTATTAATATTTGTCATTAAT
GTCAATTAATACAAATTATTAATATTAATAAAGGTTCACCCCCTTTATTACATTTGGCGTAGTCGGCAGGATT
AACCCTTTTGATGCTGGGAAGTGCAGTAATAACTTGTATAGAAATTTATAGTGCAGCTTGTATGGTAGTGTAG
TGGCAGTATTGTAGTGGCTTGTGCAGGGCAAACATACTGTTGAAAGTTAGCAATAATGTTCTGTTGGTTGAAA
AACAAAGTTAGAGGGGTAGATGATCAAACGAGCAACCTTCCCACATGGGCTAGTTCCGGACCCTGGACCCATG
TTGCTCGGGATGTGATAGGAGATCAGCAACTCTCTCAGGTGACTCACCCCGATCACCTGTTGCTGAATTTGGA
TTTGTCTATGTGTTCCAAGGAGCAGAAGTCCTCAGCACAATTGGGTTGGTTGTGTTTTCAGGCGCTCAGGGAG
GAGACAGTCCAAAGGGAGGCAGTTGAGCTTGAGCTGCAGGCAGTTAAAGATCAACTAGCCATAATGAGAGAAT
GTTTGCGCTCCTCATTAGCACACAATGAGGAGTTGGAAATAGAATTTACAAAATATGTGGTAAAAACTATGAG
TAACAATCACCATAGAAAAGAGACAGAACAAGAAGAAAATGCTGCATTAGTTAGAGTAAGAGCTCTTGTAAGT
AAAGGAGACAGGGGGGAGTTAGCCCAGTGGTCAGGAGCACATGAGGAAGTTCAAGAAGACATGATAAGACCTA
TTGTGACTACAAGACAAGTCAGTCAAAGTGCAGGTATTGTTGACAGATCTTCAGGAAATACAGAAGCAGCAGA
TCAACAGTACATTGAGCAACCAAGTTTATCTGCTGCAGTAGTTAAGTTGGGTCAGCCAATTTCCCAGGTAGGA
GGAGAAAGTGGTGTGCTTACAGATCCAATAACTTCAGTACTCCCAAACCCACATACAGTAAAAGAAGAATGGG
AAAGGCCAGACAAAGTTCCATTTACTGCACATATGAAGAATACATTGCTAGTAGGTTCTCCCACCCATTTAAA
AGCCCCGTTGTTACATAAAACTGACATATTGTTGGGTAGATTAGAATGTGCAGAGGAAAACATTCCTAAGCAA
AGGGTAGTAAAAGTCTGGGGTACAAAAAGGTTAAAAAACAGTCGCAGGCAAATGTTTTTAGATTTGCTTCAAT
ATGGTGTACCAAAGCCTGAAATTGATGGTATTCCCACTGCTGATTTGTTGAAGCGCTCACAAAAGGTGAGTTT
GAAATTACCACAACCACAGAATGCGGCAACCTGTAATGTAGAGGACCTATCTTTCCTTTTATCCCATACATAG
AAAAGGAAATGGCTAAACATATGGGAGGAGGACATCTAGGAGGTTATATGAGAGGATAACTAGTGATAGTTTA
GGAAAAATACAGAAGCAAAACAATGTTGAGAATGGTACAATTCTACTGGTTTAGTAATAGAACAGCTGTATAT
CGTAAAAGGTGTTTCTCTTTGTATGTAAAGAATTGCTGCCCCCTCCCTTTAGACACAGACTAGTTCAACTGAG
GGAGTGATTTATTGCTCAAGATAAGAGAAACAAAAACTAAATGTTACAGTGTGTTTAATTGTACTGTATGTTA
ATGTCATATGTTTTTTTGTTTCAGGATTTAAGAATCTGATTATTTTGAGTTAATATGTTTTTTAATAACAGGC

ATCAAGAGTTGATGGATCCATTATTTTTTACACTTTATTTGTTTTGTTTTTAAGTGCACAAATTTGTGCGTAA
AGGCCTTAAGACATGTCTATTTTGTTAAAAAGTTTTTTAAAACTGTAGTACTACAATTAACAATGAAACTTG
AAAGTTTTTAACACTTTTTGTTGTATGTTTCTGAGAATGTGTGGGCCCACATGTATGTTGTTATAGTTTGTCT
TGTCTTAATTTGACAATGACTTAAATGTGACAGTGACTGAAAAAAAGTCTTGACAAAATAATACAAAATTAAG
AAAGTTGTTAAATTTGACAAAAACAGATCTTAAATGCTAAGAACTGATGTTGCCTTGATCTTTTGTTTTATAG
ATGTTTATCAAGTGCATTAACAAATGGGTTAATAGCAAAACAATGGACAGCAAACAAGGATTTGGACATTGTT
TATCTTCAATGGACATCAGTACAAAGTATTGAAGAACCTGGAATTCATTTGAATGGTGACCTGGAAACTTAAA
TGGACATTGGTTTTGGACTTGGACTTTGGATAAATGAATATTTATGTTTTGTTCAATTGTTTCTACCGGCTGA
AATTAGCATTGCATACACTATTGTACTAATGGTAATAGGAATGTTATGGCTTGTTACATGAACTGTAAAGTGC
TAGTTAGTGGGTTTTAGAGCACAGGCAGCACAGCTGAACTGTATATCTCCTATGGTATATAATATTGTGCAGT
GAACCTGCAGTATAGAGCAGGCCCTGGCTGACAATCTGTAGATTTAGGCAAATGCCAGAGGTGCTGCTGTAAG
ATGCCATAGACAGTCACTATTTATTGGGCTGATGGGGTCTGTTTGGGTCTCTGTGTATTTGAAATGCCAGGGT
CTGTTTTGAAATTAGTGTCAACAAAACTCTTTGATAAAACAGAAAGAGTGAAATTTATTATTCTGAGGGAGAA
ATTGTTATGTGAAACTAACAATGTCTGTAGACAAGCATTATGGTATAATTGTTGTAAGTGTGAATAGGGTAAT
GTAATTTTATGAGTTAGTACTATGTTATCAATGGAGTTAGCTGAATGTTGGCTGTAAGAGAGTTTGGACAACT
CTATATGATTGAAGTGAAAGCCTTTTAAAGACTACAAGTTAATATACAATTCTGTTGAGGGACACGTTAGTTT
ACAAAAAGTCACTGTTAGACTTGATAGTATCATCCCAAGTGATTTGAATGCCCTGGGTGATTTGTGGCCTTAT
GGGTGGAATTTGTGGCTACTAGTGATCGACTGATATTTTGGAAAAGCATTGGCAGAATGTGTATTGGGACTAA
AACTCTGCTGAAAGGAATTCAGTTGCCATTTGGTCTATAAGAGGGGGGTTAGGTAGAAACAAAGTGCATGGCA
GATACATAATATAGTAAACATATTGCTTATCAAAGAAGCATTGTTTAACTATGTGAGCTGTAGAGCCTGCAAT
GAAACCTGCTCTTGGCCTAATTGTTTTAAGCAGCTGATAGCCTATAATGAAAATGCAGAGCAAGAGGGATGCC
TATACATGTTTGCCACATGATTGCCCAAGATATGTATCTACAAGTAGACACAGATTTTCTCACTGGACAGAAA
TCATTATAGAGGTGTTTTATGACTTGGGATGTAGGTGAGATTTGTTTAATCTTTTGTTAAATGTAGTGTCCTT
CAGTCAGAATTACAGTCATTAATTGTTTATTTGTTTAGGGTAATGGGGACAACCAGTGAATTCATATATTGCA
TTGAATGCAGCAT

>Xt-S19_CM004446.2:1821190-1829189 Xenopus tropicalis strain Nigerian chromosome 4, whole genome shotgun sequence
TGTAACACGGTGGATTTTCTATTGATTTTATTTTACAATATATACATCTGCTTTGACATATGAATCAAATCTT
AGGTTTGAGTCCAGTAAAAGCAGCAGATTTTGGGACAACTATTGTCCAGTGTTCAAGGTACAATGTCTTTGTT
CTGTGTATATTGCTGGGTAAACAGTAAACACAGGTATTGTTACCCAGCAAATATCTTTGGAATAAACAAACAG
ACAGCATATAGACAATACACACAATGTACAACTTATTTGAGCAACAAATAAGGCTTTCTACAAAGTCTAGTTA
ATAGGTTTTGTTCATGACAAAAGTTTGTGCACAAGTCAGCACTTGATGTTGGGGGTTTTGCTAAGAACTCTGG
TATAAAAGGGGCCCCCACCCTGTGTCAGTAGCTTCGCCTGGGGCTCCTGAACCAGTGCTGGGACATTTGGATC
ATCGCTTGGTTATCGGGAACCTGAAGGTCTTACACCAAAGGCTGTGGGGCTCCCGATTAGGCTGAATTGTGCA
GAACTGGCTGTACTTGTAACTATAATCATACAAAGATAAGTAAATCTATTTGATTCTATTATTACTTGTGTGT
GTGTAAGTTATTGCTCACTAACAGTCTATCAGAAGGTTTAATCATTGATCCTGCATATTTATTAATATTTGTC
ATTAATGTCAATTATTACAAATTATTAATATTAATAAAGGTTCACCTCTTTATAACATTTGGCGTAGTCAGCA
GGATTAACCCTTTTGATGCTGGGAAGTGCAGTAATAACTTGTATAGAAATTGTAGAATTTATAGTGCAGCTTT
GTATGGTAGTGTAGTGGCAGTACTGTAGTGGCTTGTGCAGGGCAAAGATACTGTTGAAAGTTAGCAATAATGT
TCTGTTGGTTGAAAAACAAAGTTAGAGGGGTAGATGATCAAACGAGCAACCTTCCCACGTGGGCTAGTTCCGG
GTCCCTGGACCCATGTTGCTCGGGATGTGATAGGAGATCAGCAACTCTCTCAGGTGACTCACCCCCATCACCT
GTTGCTGAATTTGGATTTGTCTATGTGTTCCAAGGAGCAGAATCCTCAGCACAATTGGGTTGGTTGTGTTTTC
AGGCGCTCAGGGAGGAGACAGTCCAAAGGGAGGCAGTTGAGCTTGAGCTGCAGGCAGTTAAAGATCAACTAGC
CACAATGAGAGTATGTTTGCGCTCCTCATTAGCACACAATGAGGAGTTGGAGATAGAATTTACAAAATATGTG
GTAAGAACTATGAGTAAAAAGCACCATAGAAAAGGGACAGAACAAGCAGAAAATGGTGTATTAGTTAAAGTAA
GAACTCTTGTAAGTAAGGGCTCTGGTACACGGGGAGATTAGTCGCCCGCGGCAAAACTCCCTACTCGCGGGCG
ACTAATCTCCCCGAGTTGCCTTCCCTCTGCCATCCCTGGCAGAGGGAAGGCAACTCGGGGAGATTAGTCGCCC
GCGAGCAGGGAGTTTTGCCGCGGGCGACTAATCTCCCCGTGTACCAGAGCCCTAAAAGAGACTAAGGGAGTGA
TTTATTTCTCAAGATAGAGAAACAAAATAAATGTTACATTGTGTTTAATTGTACTGTATGTTAATGTCATATG
TTTTCTGTTTCAGGATTTAAGAATCTGATTATTTTGAGTTAATATGGTTTTTAATAACAGGCATCAAGAGTTG
ATGGATCCATTATTTTTGACACTTTTTGTTTTGTTTTTTAAGTGCACAAATTTGTGCGTAAAGGCCTTAAGAC
ATGTCTGTTTTTGTTAAAAAAAAAAAAAAGTTTAACAATGAAACAACTTTTTGTTGTATGTTTCTGAGAATGT
GTGGGCCCACATGCATGTTGTTATGGTTTGTCTTGTCTTAATTGTGACAATGACTTAAATGTGACAGTGACTG

AAAAAGTCTTGACAAAATAATACAAAATTAAGAAAGTTGATAAAGTTGACAAAACAGATCTTAAATGCTAAGA
ACTGATGTTGCCTTGATCTTTTGTTTTATAGATGTTTATCAAGTGCATTAACAAATGGGTTAATAGCAAAACA
ATGGACAGCAAACAAAGATTTTGACATTGTTAATCTTGAATGGACATCAGTACAAAGTATTGAAGAACCTGGA
ATTCATTTGAATGGTGACCTGGAAACTTAAATGGACATTGGTTTTGGACTTCATAAATGAATACTTATGTTTT
GTTCAACTGTTTCTACAGACTGAAATTAGCATTGCATACACTATTGTACTAATGGTAATAGGAATGTTAGGGC
TTGTTACATGAACTGTAAAGTGCTAGTTAGTGGGTTTTATAGCACAGGCAGCACAGCTGAACTGTATATCTCC
TATGGTATATAATATTGTGCAGTGAACCTGCAGTATAGAGCAGGCCCTGGCAGACAATCTGTAGATTCAGGCA
AATGCCAGAGGTGCTGATGTAAGATGCCATAAACAGTCACTATTTATTGGGCTGGGGGGGCTGTTTGTGCCTC
TGGGTACTGGGAATGCCAGGGGGGCTGTAAGGTGCCACAGACAGTCACTATTTATTGGGCTGGGGGGGCTGTT
TGTGCCTCTGGGTACTGGGAATGCCAGGGGGCTGTAAGGTGCCACAGACAGTCACTATTTATTGGGCTGGGGG
GGGGCTGTTTGTGCCTCTGGGTACTGGGAATGCCAGGGGGGCTGTAAGGTGCCACAGACAGTCACTATTTATT
GGGCTGGGGGGGGGGCTGTTTGTGCCTCTGGGTACTGGGAATGCCGGGGGGGCTTTAAGGTGCCACAGACAGT
CACTATTTATTGGGATGGGGGGGCTGTTTGTGCCTCTGGGTACTGGGAATGCCAGGGGGGCTGTAAGGTGCCA
CAGACAGTCACTATTTATTGGGCTGGGGGGGGGGCTGTTTGTGCCTCTGGGTACTGGGAATGCCGGGGGGGCT
TTAAGGTGCCACAGACAGTCACTATTTATTGGGATGGGGGGGCTGTTTGTGCCTCTGGGTACTGGGAATGCCA
GGGGGGCTGTAAGGTGCCACAGACAGTCACTATTTATTGGGCTGGGGGGGGGCTGTTTGTGCCTCTGGGTACT
GGGAATGCCGGGGGGGCTGTAAGGTACCACAGACAGTCACTATTTATTGGGCTGGGGGGGCTGTTTGTGCCTC
TGGGTACTGGGAATGCCAGGGGGGCTGTAAGGTGCCACAGACAGTCACTATTTATTGGGCTGGGGGGGGGCTGT
TTGTGCCTCTGGGTACTGGGAATGCCAGGGGGGCTGTAAGGTGCCACAGACAGTCACTATTTATTGGGCTGGG
GGGGGGCTGTTTGTGCCTCTGGGTACTGGGAATGCCGGGGGGGCTTTAAGGTGCCACAGACAGTCACTATTTA
TTGGGATGGGGGGGCTGTTTGTGCCTCTGGGTACTGGGAATGCCAGGGGGGCTGTAAGGTGCCACAGACAGTC
ACTATTTATTGGGCTGGGGGGGGGGCTGTTTGTGCCTCTGGGTACTGGGAATGCCGGGGGGGCTTTAAGGTGC
CACAGACAGTCACTATTTATTGGGATGGGGGGGCTGTTTGTGCCTCTGGGTACTGGGAATGCCAGGGGGGCTG
TAAGGTGCCAACAGACAGTCACTATTTATTGGGCTGGGGGGGGGCTGTTTGTGCCTCTGGGTACTGGGAATGC
CGGGGGGGCTGTAAGGTACCACAGACAGTCACTATTTATTGGGCTGGGGGGGGGCTGTTTGTGCCTCTGGGTA
CTGGGAATGCCCGGGGGGCTGTAAGGTGCCACAGACAGTCACTATTTATTGGGCTGGGGGGGCTGTTTGTGCC
TCTGGGTACTGGGAATGCCAGGGGGGCTGTAAGGTGCCACAGTCACTATTTATTGGGCTGGGGGGGGGGGCTG
TTTGTGCCTCTGGGTACTGGGAATGCCAGGGGGGCTGTAAGGTGCCACAGACAGTCACTATTTATTGGGCTGG
GGGGGCTGTTGTGCCTCTGGGTACTGGGAATGCCGGGGGGGCTGTAAGGTGCCACAGACAGTCACTATTTATT
GGGGTGGGGGGGGCTGTTTGTGGCCTCTGGGTCTGGGAGTGCCAGGGGGGCTGTAAGGTGCCACAGACAGTCA
CTATTTATTGGGCTGGGGGGGGCTGTTTGTGCCTCTGGGTACTGGGAGTGCCAGGGGGGCTGTAAGGTGCCAC
AGACAGTCACTATTTATTGGGCTGGGGGGGCTGTTTGTGCCTCTGGGTACTGGGAATGCCAGGGGGCTGTAAG
GTGCCACAGACAGTCACTATTTATTGGGCTGGGGGGGGGCTGTTTGTGCCTCTGGGTACTGGGAATGCCAGGG
GGGCTGTAAGGTGCCACAGACAGTCACTATTTATTGGGCTGGGGGGGGGCTGTTTGTGCCTCTGGGTACTGGG
AATGCCAGGGGGGCTGTAAGATGCCATAAACAGTCACTATTTATTGGGCTGGGGGGGGCTGTTTGTGCCTCTG
GGTACTGGGAATGCCGGGGGGCTTTAAGGTGCCACAGACAGTCACTATTTATTGGGATGGGGGGGCTGTTTGT
GCCTCTGGGTACTGGGAATGCCAGGGGGGCTGTAAGGTGCCACAGACAGTCACTATTTATTGGGCTGGGGGGG
GCTGTTTGTGCCTCTGGGTACTGGGAATGCCAGGGGGGCTGTAAGGTGCCACAGACAGTCACTATTTATTGGG
CTGGGGGGGCTGTTTGTGCCTTTGGGTACTGGGAATGCCAGGGGGGCTGTAAGGTGCCACAGACAGTCACTAT
TTATTGGGCTGGGGGGGGCTGTTTGTGCCTCTGGGTACTGGGAATGCCAGGGGGGCTGTAAGGTGCCACAGTC
ACTATTTATTGGGCTGGGGGGGGCTGTTTGTGCCTCTGGGTACTGGGAATGCCAGGGGGGCTGTAAGGTGCCA
CAGACAGTCACTATTTATTGGGCTGGGGGGGGCTGTTTGTGCCTCTGGGTACTGGGAATACCAGGGGGGGCTG
TAAGGTGCCACAGACAGTCACTATTTATTGGGCTGGGGGGGCTGTTTGTGCCTGTGGGTACTGGGAATGCCAG
GGGGGCTGTAAGGTGCCACAGACAGTCACTATTTATTGGGCTGGGGGGGCTGTTTGTGCCTCTGGGTACTGGG
AATGCCGGGGGGGCTGTAAGGTGCCACAGACAGTCACTATTTATTGGGCTGGGGGGGGCTGTTTGTGCCTCTG
GGTACTGGGAATGCCAGGGGGGCTGTAAGGTGCCACAGACAGTCACTATTTATTGGGCTGGGGGGGGCTGTTT
GTGCCTCTGGGTACTGGGAATGCCAGGGGGGCTGTAAGGTGCCACAGACAGTCCACTATTTATTGGGCTGGGG
GGGGCTGTTTGTGCCTCTGGGTACTGGAATGCCAGGGGGGCTGTAAGGTGCCACAGACAGTCACTATTTATTG
GGCTGGGGGGGGCTGTTTGTGCCTCTGGGTACTGGGAATGCCGGGGGGGCTGTAAGGTGCCACAGACAGTCAC
TATTTATTGGGCTGGGGGGGCTGTTTGTGCCTCTGGGTACTGGAATGCCAGGGGGCTGTAAGGTGCCACAGAC
AGTCACTATTTATTGGGCTGGGGGGGCTGTTTGTGCCTCTGGGTACTGGGAATGCTAGGGGGGCTGTAAGGTG
CCACAGACAGTCACTATTTATTGGGCTGGGGGGGCTGTTTGTGCCTCTGGTACTGGGAATGCCGGGGGGGCTG
TAAGGTGCCACAGACACTCACTATTTATTGGGCTGGGGGGGCTGTTTGTGCCTCTGGGTACTGGGAATGCCAG
GGGGGCTGTAAGGTGCCACAGACAGTCACTATTTATTGGGCTGGGGGGGGCTGTTTGTGCCTCTAGGTACTGG

GAATGCCGGGGGGGCTGTAAGGTGCCACAGACAGTCACTATTTATTGGGCTGGGGGGGGCTGTTTGTGCCTCT
GGGTACTGGGAATGCCGGGGGGGCTGTAAGGTGCCACAGACAGTCACTATTTATTGGCTGGGGGGGGGGCTGT
TTGTGCCTCTGGGTACTGGGAATGCCAGGGGGGCTGTAAGGTGCCACAGACAGTCACTATTTATTGGGCTGGG
GGGGCTGTTTGTGCCTCTGGGTACTGGGAATGCCAGGGGGGCTGTAAGGTGCCACAGACAGTCACTATTTATT
GGGCTGGGGGGGGGGCTGTTTGTGCCTCTGGGTACTGGGAATGCCAGGGGGGCTGTAAGGTGCCACAGACAGT
CACTATTTATTGGGCTGGGGGGGGGCTGTTTGTGCCTCTGGGTACTGGGAATGCCAGGGGGGCTGTAAGGTGC
CACAGACAGTCACTATTTATTGGGCTGGTGGGGGCTGTTTGGGCCTCTGTGTATTTGAAATGCCAGGGTCTAT
TTTGAAATTAGTTTGAAACGAAACTCTTTGATAAAACAGAAAGAGTGAAATGTATTATTCTGAGGGAGAAATT
GTTATGTGAAACTGACAATGTCTGTAGACAAGTATTATGGTATAAGTAATTATTGTAAGTGTGAATAGGGTAA
TGTTATTTTGTGAGTTAGAACTGTGATGTCAATGGGGTTGGCTGAAGGAGAGTTTGGACAACTCTATATGATT
GAAGTTAAAGACTACAACTTAATATGCAATTCTGTTGAAGGACACAGTTGTTTATAAGAAGTCACTATTAGAC
TTGATAGTATCATCCCAAGTGATTTGAATGCCCTGGCTGATTTGTGGTCTTATGGGTTGAATTTGTGGCTACT
GGTGATAGACATATTTTGGAAAAGCATTGGCAGAATGTGTACTGAGACTAAAACTCTGCTGAAAGGAATTCAG
TTGTCATTTGGTCTATAAGAGGGGGGTTAGGTACAAACAAAGTGCATGGCAGATACATAATATAGTAAACATA
TTGCTTATCAAAGAAGCACTGTTTAACTATGTGAGCTGTAAAGCCTGCAATGAAACCTGCCCTTGGCCTAATT
GTTTTGAGCAGCTGATGGTCTATATTGAAAATGCAGAGCAAGAGGGATTCCTATACATGTTTGCCCAAGATAT
GTATCTACAAGTAGACACAGATTTTCTCACTGGACAGAAATCATTATAGAGGTGTTTTATGACTTGGGATGTA
GGTGAGATTTGTTTAATCTTTTGTTAAATGTAGTGTCCTTCAGTCAGAATTACAGTCATTAATTGTTTATTTG
TTTAGGGTAATGGGGACAACGAGTGTATGCATATATTGCATTGAATGCAGCATTGTATGTTATCTGTATTAGA
GGACTTACCAATTTGTATACTGTTGGTTATTATTATCAAATCAGTATATTTTGTAAATCAAGGGGTGGAATGT
AGCACGGTGGATTTTCTATTGATTTTATAATGATTTATAATATATACATCTGCTTTGACATATGAATTAAATC
TTAGGTTTGAGTCCAGTAAAAGCAGCAGATTTTGGGACAGCGATTGTCCAGTGTTCAAGGTACAATGTCTTTG
TTCTATGTATATTGCTGGGTAAACAGTAAACACAGGTATTGTTACCCAGCAAATATCCTTGGAATAAACAAAC
AGACAGCATATAGACAATACATTTTATGTACAACTTATTTGAGCAACAAATGGGCCTTCTACAAAGTCTAGTT
AATAGGTTTTGTTCATGACAAAAGTTTGTACACAATTCAGCACTTGGTGTTGGGGGGTTTGCTAAGAACTCTG
GTATAAATGGGGCCCCCACCCTGTGTCAGTAGCTTCGCCTGGGGCTCCTGAACCAGTGCCGGGACATTTGGAT
CATCGCTTGGTTATCGGGAACCTGAAGGTCTTGCACCAAAGGTTGTGGGGCCCCCGATTGTGCTGAATTGTGC
AGAACTGGCTGTGCTTGTAGATAAGTAAATCTATTTAATTCTATTAGTACTTGTGTGTATGTAAGTTATTGCC
CACTAACAGTCTATCAGAAGGTTTAATCATTGATCCTGCATATGTATTACTATTTGTCATTAATGTCAATTAA
TACAAATTATTAATATTAATAAAAGGTTCACCCCTTTATTACA

>Xt-S20_CM004448.2:4091567-4105257 Xenopus tropicalis strain Nigerian
chromosome 6, whole genome shotgun sequence
TGTAGCACCGTGGATTTTATTTTGATTTTATAATGATTTACAATAATATATACATCTGCTTTGACATATGAAT
CAAATCTTTGAGTCAAGTAAAAGCAGCAGATTTTGGGACAAATACTGTCCAGTGTTCAAGGTACAATGTCTTT
GTTCTATGTATATTGCTGGGTAAACAGTAAACACAGGTATTGTTACCCAGCAAATATCCTTGGAATAAACAAA
CAGACAGCATATAGACAATACACTCTATGTACAACTTATTTGAGAAACAAATAAGGCTTTCTACAAAGTTAGT
AAATTGTCTAGTTAGGTTTTGTTCATGACAAAAGTTTGCACACAATATTGCACAAGTCAGCACTTGGGGTTGG
GGGTTTGCTAAGAACTCTAGTATAAAAGGGAGCCCCCACCATGGGTCAGTAGCTTCGCCTAGGGCCCCTCAAC
GAGTGCCGGGACATTTGGATCATCGCTTGGTTATTGGGAACCTGAAGGTCTTGGACCAAAGGCTGTGGGGCTC
CCCGATTGGGGTGATTTGTGCAGAACTGGCTGTGCTTGTAACTATAATCATACAAAGATAAGTAAATCTATTT
AATTCTATTATTACTTGTGTGCGTGTAAGTTATTGCTCACTAACAGTCTATCAGAAGGCTTAATCATTGATCC
TGCATATGTATTAATATTTGTCATTAATGTCAATTAATACAAATTATTAATATTAATAAAGGTTCACCCCCTT
TATTACATTTGGCGTAGTCGGCAGGATTAACCCTTTTGATGCTGGGAAGTGTAGTAATAACTTGTAAAGAAAT
TATAGAATTTATAGTGCAGCTTTGTATGGTAGTGTAGTGGCTTGTGCAGGGCAAACATACTGTTGAAAGTTAG
CAATAATGTTCTGTTGGCTGAAAAACAAAGTTAGAGGGGTAGATGATCAAACGAGCAACCTTCCCACATGGGC
TAGTTCCGGACCCTGGACCCATGTTGCTCGGGATGTGATAGAAAATCAGTAGAGATGTAGCGAACCTCACAAA
AAAAGTTCGCGAACCCGTTCGCGAACTTCCGCCAAAAAGTGCGAACTTTTGCGAACTTTGCGAACCCCATAGA
CTTCAATGGGAAGGCGAACTTTAAAACCTAGAAAAGCCATTTCTGGCCAGAAAACTGATTTTAAAGTTGTTTA
AAGGGTGTCACGACCTGGGCAGTGATATGCAGGAGGGGGATCAAGGGCAAAAATTTCTCTGAAAAATACGTTG
TTGACACAGCGTTGCGTTTTGTGCTGTAAAGGGCAGAAATCACACTACATTCCTAAACTTGTGTAATAAACTG
CTTTAAAACGTCCGGCGCCTACATGCCAATCAAGTCGTGTAAAGGTTACGGCCGGTTCCCACGCAAAGACAAA
ACGCCGCAGTAGTGGATACGGAATATATTATTGCTGGTGGAAAAACATCGCTCAGGTGATTTTATTGCAATGC
AATGTCACTACTATAATGTTATTTCATTTAATGTTTGGTGCAGGAAACAAATTTACACGGGGGCAACAAAGAC

AATGAATTTTAGGAAGGCAAATTTTAGCTCCTTAAGGGCAGCGCTTCAGGGCATAGATTGGGGCATTATGTTT
TCTGATAAAAATACAGAGCAGAAATGGTTGTCATTTAAAATGATATTAAATCATTACTGTTCTCAATTCATTC
CATTAATAAGAAAAAGTAGAAGTGTTAAGAATCACCCTATGGGGCTTAACTCTGAGGGAAAGAAGTTAATAGG
GAGAAAAAGGAAAGCTTTTAAGAAATATAAGTCAGAGGGGACAGTAGCTGCGTTTAATGAATATAAACACTAT
AACAAGTGTGTAAAACAGCAATCCGGAAGGCAAAGATAGAAAATGAGGAGCGCATCGCGGCCGAGGCCAAGAC
TAACCCCAAAAAGTTTTTTAAGTATATTAATAGTAAAAAGATGCAGGTTGAGGGTGTGGCCCCATTGAGTTAT
AATAACAATATGGTTACAGCGGATACAGAAAAGGCAGATGTGCTTAACCAGTTCTTTTCTTCTGTGTATACAG
TAGGGGGAGCCAGAGGGCCAAGTCCCACCCAATAGCTGCACTGTTGCCTCAGCTCCAACTACACAGTGGTTGG
CACAGGATATGGTGCTTAAAGGGTTACACACGATAAATGTAAACAAGGCACCTGGGCCAGATGGAATACACCC
TCGGGTACTGAGAGAGCTAGGGGCAGAATTGCAGTGGCCCTTGTTTCTGATATTCTCAGACTCTCTTTCATCA
GGTATGGTACCTAGGGATTGGAAGAAGGCGAATGTCATTCCCATATTTAAAAAGGGAGTAAGATCTCAGCCTG
GCAATTATAGGCCTGTAAGTCTGACATCCGTGGTGGGCAAGTTATTTGAAGGCTTGTTAAGGGATCACATTCA
AAATTATGTAGTGGAGAATGCCATTATGAGCAGTAATCAGCATGGCTTTATGAAGGACAGGTCATGTCAGACC
AATTTAATTGCTTTTTATGATGAGGTAAGTAAGAAGCTGGACAGTGGGGATGCAGTAGATATCATCTATTTGG
ATTTTGCGAAAGCATTTGATACCGTTCCCCACAAACGACTGCTTTCTAAGCTAAGGTCTATTGGTCTTAGTGA
AGCCGTTTGCACATGGATAGAAAACTGGCTACAGGATCGGGTACAGAGGGTGGTTGTTAATGGTACATTCTCT
ACTTGGAATAAGGTCCTCAGTGGGGTCCCTCAGGGTTCTGTACTGAGTCCACTTTTGTTTAATTTGTTCATAA
ATGACTTAGGGGAGGGTATTATGAGTAATGTATCAGTGTTTGCAGATGACACAAAACTCTGCAGACCAGTCAA
TTCTATCCAGGATGTGACATCCCTGCAGCAGGATCTTGGCCAACTGGCAATCTGGGCAGCTAAGTGGCAGATG
AGATTTAATGTGGATAAATGTAAGGTCATGCACCTGGGATGTAAAAATATGCAAGCCCCGTATACCCTTAATG
GGACTGCACTAGGCAAATCCCTAATGGAGAAGGACCTTGGAGTCCTTGTAGATAATAAACTTGGCTGTAGCAA
GCAATCCCAGGCAGCAGCTGCAAGGGCAAACAAGGTTTTGAGCTGTATTAAAAGGGGTATAGATTCACGGGAG
GAGGGGGTTATTCTTCCCCTTTACAGAGCGCTGGTAAGGCCCCATCTAGAATATGCTGTTCAGTTTTGGTCTC
CAGTGCTCAAACGGGACATTACTGAGTTAGAGAGGGTCCAGAGAAGGGCAACTAAGCTGGTAAAGGGTATGGA
AAGTCTCAGTTATGGGGAAAGACTGGCCCAGTTGGGTCTGTTTACACTGGAGAAGAGGCGCTTAAGAGGTGAC
ATGATAACTATGTATAAATATATAAAGGGATCATATAATAACCTTTCTAATGTTTTATTTACCAGTAGGTCCT
TCCAACGGACACGAGGGCACCCACTCCGTTTAGAAGAAGGGAGGTTCCATTTAAACATTCGGAAAGGATTTTT
TACAGTGAGAGCTGTGAGGTTCTGGGGTCCCCTCCCCGAATCAGTCGTGCTGGCTGATACATTATATAACTTT
ATATAGGGGCTGGATGGATTCTTAGCAAGTGAGGGAATACAGGGGTTATGGGAGATAGCTCTCAGTACAAGTT
GATCCAGGGACTGGTCCGATTGCCATCTTGGAGTCAGGAAGGAATTTTTTCCCCTCTGTGGCAAATTAGAGAG
GCTTCAGATGGGGTTTTTTGCCTTCCTCTGGATCAACTAGTAGTTAGGCAGGTTATATATAGGCATTATGGTT
GAACTTGATGGACGTATGTCTTTTTTCAACCCAACTTACTATGTTACTATGTTACTATGTTACTATGTGTAAT
GTGTTTGGGGCACTACTATAAATAACAGCAAACAGCACTGGACACGTTAAAGAACAGTTTAAATTAAAAAAAA
ATAAAAATTAATAATAAAAAAAAAGTGATCTCTGGTTGGTGCTGGGGGTGAACTACTAGGAGCAGCACACCAG
TCCCCCACAGCTAGACTAATAGCACTCAGCTCTATAAATTACAGTAGCAAAGTAAAAAAACACAAATAAAAAA
AAATTATGTGAATGTGTGGTTGGTGCTTAGTGCACTACTATGAGCAGCACACCTGTCTCCAACACACACAGAC
GGAGCTGCAGTACACAATGAAAAGAACACTAGAGTAACAGTAATCAGAAAATAAAAGCAGTCCTTACAAGGAC
TATTGGGTTACAGCAGATGAGATCAGCAGGACAGCTGTCCCCAGCAGCTACATACAGAGCAGTAGAAAGTAGA
TTACTAGTCAGCAAAGCTACCTAAACTGTCCCTCAAACCCCTGCACAGCTCTCTCCCTATGCTAACTCATCAA
GCACACACAGGCAGAATGTAAAATGGCTGCTGGGCTTCGGTTTATATATGGAAGGGAGTGGTCCGGGGGTGGT
CCAGGAGGGAGAGCTGCCTGATTGGCTGCCATGTATCTGCTGGCTCTGGGGTGAGAGGTCAAAATTTGGCTCC
AGCTAAGGCGAACCCAAAATTGCGAACTTCACTAAAAGTTCGCGAACTTGCAAACTTGCGAACACCCGATTTT
CGTGCGAATTAGTTCTCCAGCGAACAGTTCGCTACATCTCTAGAAATCAGCAACTCTCTCAGGTGACTCACCC
CGATCACCTGTTGCTGAATTTGGATTTGTCTATGTGTTCCAAGGAGCTGAAGTCCTCAGCACAATTGGGTTGG
TTGTGTTTTCAGGCGCTTAGGGAGGAGGCAGTCCAAAGGGAGGCAGTTGAGCTTGAGCTGCAGGCAGTTAAAG
ATCAACTAGCCACAATGAGAGAATGTTTGCGCTCCTCATTAGCACACAATGAAAAGTTGGAGATAGAATTTAC
AAAATATGTGGTAAAAACTATGAGTAACAATCACCATAGAAAAGAGACAGAACAAGAAGAAAATGGTGCATTA
GTTAGAATAAGAGCTCTTATAAGTAAAGGAGACTGGGGGGAGACTGGTCAGGAGCACATGAGGAAGTTCAAGA
AGACATGATAAGACCTATTGTGACTACAAGACAAGTCTGTCAAAGTGCAGATTTTGCTGACAGATCTTCAGGA
AATACAGAAGCAGCAGATCAACAGTACACTGAGCAACCAAGTTTATCTGCTGCAGTAGTTAAGTTGGGTCAGC
TAATTTCCCAGTTAGGAGGAGAAAGTGGTGTGCTTACAGATCCATTAACTTCAGTACTCCCAAACCCACATAC
AGTAAAAGAAGAATGGGAAAGGCCAGACAAAGTTCCATTTACTGCACATATGAAGAATACATTGCTAGTAGGA
TCTCCCACCCATTTAAAAGCCCCATTGTTACATAAAACTGAAATATTGTTGGGCAGATTAGAATGTGGAGAGG
AAAACATTCCTAGGCAAAGGGTAGTAAAAGTCTGGGGTACAAAAAGGTTAAAAAACATTTGAAGGCAGATGCT

TTTAGATTTGCTTCAATATGGTGTACCAAAGCCTGAAATTGATGGTATTCCTAACACTGATTTGTTGAAGCGC
TGGAAAAAACTGAGACAAAAGGTGAGGTTGAAATTACCACAACCACAGAATGTGGCAACCTGTAATGTAGAGG
ACCTATCTTCCCTTTTAGCCCATACATAGAAAAGGAAATGGCTAAACATAAGGGGAACATCTAGGAGGTTATA
TGAGAGGATAACTAGTGATAGTTTAGGAAAAATACAGAAGCAAAACAATGTTGAGAATGGTACAATTCTACTG
GTTTAGTAATAGAACAGCTGTATATCGTAAAAGGTGTTTCTCTTTGTATGTAAAGAATTGCTGCCCCCTCCCT
TTATACACAGACTAGTTCAACTGAGGGAGTGATTTATTGCTCAAGATAGAGAAACAAAATACATTTTACATTG
TGTTAAATTGTACTGTATGTTAATGTCATTTGTTTTTCTGTTTCAGGATTTAAGAATCTGATTATTTTGAGTT
AATATGGTTTTTAATTACAGACATTAAGAGTTGATGGATCCATTATTTTTGACAAATTTTTGTTCTGTTTTTA
GATTTCTGTGTAAAGGCCTTAAGACATGTCTGTTTTTTTTGTTAAAAAGTTTTTAAAGAACTGTGGTACTACA
AATAAGAATGAAACTGGAAACACTTTGGGCCTGATTCACTAAAGTGCGATAAAATTTATCGCATGTTTTGCGT
TAAATTAGTCGCGATAATTAGCGCTCGATTCACCATAGTATTATCGCGTGCGTTAACTCGCCATATCGCATGC
GTTAATTTCCGCGCTGAAAAGAATACAATCGCATGATTCACAAACACTTAGGCGCACTAAATATCGCATTGGT
CTATGCAAAAATTAACCCATACTACAGGCAGGTGATAAATTCTAGAAAAGTGCAGTAAATGAGTTTTTGGCAA
TAAAATATGGACTTACAGTGTTATTTATTCAGTCTGTGTCTTTTTACTCAATTTTACTAAAGTCTTGGGATGT
ATGGAATTTCCCTACTAATATACAGTACTATAGTGGTAAATATTTAGTCGCCATAATATACAGTACTATAGCG
GTGAATATTTAGTCGCCATAATATACAGTACTGTAGCGGTGAATATTTAGTCGCCATAATATACAGTACTGTA
GCGGTGAATATGTTGTCGCCAAAATATACAGTACTATAGCGGTAAATATTTAGTCGCACAAAATACATTACTA
GGTATAATAATTATAGAAAAGTACAGTAAATGAGCTTTTGCCAATAAAATATGGACTTTGCAGTGTTATTTAT
TCAGTCTGTGTTTCCCCCAGAGTGACGCAGCCGCCAGTTTGCAGCGAAATGGGCATTTTTAATACAGTAATTT
TCTGCAAGTATTGGCGTGTATGGCTAACATGGCGTGCGTTTATTCGCGCGACTATTTATATGCAGCTACAAGT
GATGAAATGTTTCGCCAGGCATGGATTCGCAGCAAATTTTTGGACGTACAGCAAATTTTTCCGCGGCGAATTT
TTTCATGCGTTTCGCAAAACAATCTGCCAATGGCAAAACGCATAAAAAAATTTGCCACTCAAAAATTCACCGC
ACGTCCAAAAATTTACACAGGCGTCAAAAAATAATAGTTGCGGGCAACAATTTTTTTGCCCGCACAACATTTT
TGCCGTTTTGTGGATCTTTCGAAAGATTTGCTAATTTTTCATAAAAGATAACCAGAACACATTCACTCATCAC
TAGTGGCTACTATTTATATGTGGCTAATATTTATATACACTATTTATATGCGGCGACAATTTATATACACTAT
TTAAAGCTACCATTTATATCCGGCGACTAACCGCGAGAGTTATTTAGCGCATGTACCGGCAAATACCGCATTT
AAATTGTCTTCGCGAGTTAAATAACGCATGCAATATCGCGCGTAAAATAGCGCAAGTATGCTTATAGTGAATC
GTGCGAAAAGTCGCGAAAATTAATACGCAATAAAATTTTTACCGCATGCTAAACAGATCTTAAATGCTAATTT
TTATCAAGTGCATTAACAAATGGGTTAATAGCAAAACAATGGACAGCAAACAAGGATTTGGACATTGTTAATC
TTCAATGGACATCAGTACAAAGTATTGAAGAACCTGGAATTCATTTGAATGGTGACCTGGAAACTTAAATGGA
CATTGGTTTTGGACTTTATAAATGAATATTTATGTTTTGTTCAATTGTTTCTACCGGCTGAAATTAGCATTGC
ATACACTATTGTACTAATGGTAATAGGAATGTTATGGCTTGTTACATGAACTGTAAAGTGCTAGTTAGTGGAT
TTTAGAGCACAGGCAGCACAGCTGAACTGTATACCTCCAATGGTATATAATATTGTGCAGTGAACCTGCAGTA
TAGAGAAGACCCTGGCTGACAATCTGTAGATTCTGGCAAATACCAGAGGGGCTGCTGTAAGATGCCATAGACA
CTCACTATTTATTGGGCTGGTGAAAGGATGTTTGGGGCTCTGTGTACTTGAAATGCCAGGGCCTATTTTGAAC
TTATCCCTTATGAAGTCAGTCTATGAAATTCTTTGATAAAAAAGAAAGATAGTGTAATTTATTATCCTGAGGG
AAAAATTGTATGTGAAACTAGAAAGGGAAGTTTGAGAACAAACTTCATGTTGGGTTGAAAAACATGAAGTCAC
TGAATGGGCTCCGCCCACTTTCTCTGACCATGGACCACAGTTATATAGTAAAAACCACTGTGCAAAGTTTGGG
GACCCTGGTTTTAATAGTGTCTGCATGGCAGCAACTTAAATTTCCCCACTGAAAGTCAACGACATCTGATTGG
TGGTTGGTGGCTCCACCCCCTTTTTCTAACCTGGAACTGCAGTTACCCAGTGACTAACTCTGCGAAGTTTAGG
GACCCTAGGATTAATAGTTAAAGAACGGCAGCAATTACACCAATAAAAGTCAATAGGTGAATTGTGATTGGTG
GTTCCGCCCACTTTTTCTAACCTTGAGTAGAAGTCACCCAGTGACAAACAGTGGGCACCCTGGCATAAATAGT
TTGAGAATGACAGCATTTTAAATTTAAACCAATAAAATTCCATGGTTGAAATCTGATTGGCTGTTAGTGGCCC
AACCTTCTTTTCCTATTTTTGAACTGCAGTCCCCCAGTGACCAACATTGCAAATTTTGGGGACTCAGGCATTC
ATTGTGAGGCTGAGAGCATTTTACACTTCACCATTAAAAGTAAATAGGTGAAATGTTATTGGCTGTTGGTGGC
TCCACCCACTTTTTTCCTAACTTTGAATGGAAAATACCCAGTGACTAACTCTGGAAACTTTAACAACCCTGGA
ATTAATATTTAAATAATGGCATCAGTTTAAATATAAACCAAGGAAATCTAATGGGTGGAAATGGATTGGCCGT
TGGTGGCTCCTCCCACTTGTTTGGGAACCCTGATATAAATAGTGTGAGCAAAGCAACATTTTAAATATAAACC
AAAAAAATTTAATAGGTGAAGTCTGATTGGCTGTTGGTGGCTCCACCCACGTTTTAAAACCTAAAAATGCAGT
TCCCTAGTGACCCTGGTGTTAATACTGTGAGAATGGCAGCAGGTTGAATTTCCCATTGAAAATCAATAGGTAA
AATCTGATTGGCTGTTGTTGGCTCCACCCACTTTTTCTAACTCTGAACTGCATTTACCAGGTGACTAGCTCTG
CAAATTTTGGAGACCTTAGTATTAATATTTAAAGAATGGCTGCAGTTTAAATTTAAACATATGAAGTCTATAG
GTGAAATCTAATTGGTTGTTGTTGGCCCCACCCACTTTTCTAAACTTGGAACATAGTCACCTAGTGACAAACT
GTGCAAAGTTTGGGGACCCTGACATTAAACATGTGAGAATGGCAACAGTTAAAATTTTCCCACTGAAAACAAT

GAAAGAAATGTGATTGGCTTTTAGTGGCCCTGCCCACTTTTTCTAACCTTGGGTACAAAGTCACGCAGTGACT
GACTGTGCAAAGTTTGGGAACTCTGGCATCAAGCCAATAAAATTTAATGGGTGAAATCTGATTGGCTGTTGGT
GGCCCCGCCCACTTTTTCCAAAACTAAAACTGTAGTCCTCTAGTGACCAACTGTGAAAAGTTTGGGGACCCTG
GTGTTAATACTGTCAGAATGGCAGCAGGTTGAATTTCCCCATTTAGAGTCAATAGGTAAAATCTGATTGGCTG
TTGGTGGCTCCACCCACTTACAAAAATACAAAAAAACTTAAATGCGTAGTCACCCAGTGTCTGACTATGCAAA
GTTTGGGAAGCCTGGCAACAAACCAATAAAAATCAATAGGTGAAATCTGATTGGTTGTTGGTGGCTCCGCCCA
CTTTTCAAAACTAAAATTGCAGTCCCCTAGTGACCAACTGTGAAAAGTTTGGGGACCCTGGTGTTAGTACTGT
GAGAATGGCAGCAGGTTGGATTTCCCCATTGAAAGTCAATAGGTAAACTCTGATTGGCTGTTGGTGGCTCCGC
CCACTTTTTCTTACCTTGAATGCGTAGTCATCCAGTGACTGACTGTGCAAATTTTGGAAAATCTGGCATCAAA
CCAATAAAAATCAATAGGTGAAATCTAATTGGTTGTTGTTGGCCCCACCCACTTTTCTAAACTTGGAACATAG
TCACCTAGTGACATACTGTGCAAAGTTTGGGGACCCTGACATTAAACATGTGAGAATGGCAGCAGTTAAAATT
TTCCCACTGAAAACAATGAAAGAAATATGATTGGCTTTTGGTGGCCCCGCCTACTTTTTCTAACCTTGAGTAC
AAAGTCACGCAGGGACTGACTGTGCAAAGTTTGGGAAGCCTGGCATCAAACCAATAAAAATCAATAGGTGAAA
TCTGATTGGTTGTTGGTGGCTCCGCCCACTTTTCAAAACTAAAATTGCAGTCCCCTAATGACCAACTGTGAAA
AGTTTAGGGACCCTGGTGTTAATTCTGTGAGAATGGCAGCAGATTGAATTTCCCCATTGAAAGTCAAAAGGTA
AACTCTGATTGGCTGTTGGTGGCTTCGCCCACTTTTTCTTACCTTGAACCACAGTTACCTGGTGCCTAACCCT
GTAAAGTCTGGGGTCCCCGGTGTTATTACTGTGAGACTGGCAGCAGGTTGGATTTCTGCCAAGTTAATAGGTA
AAATCTGATTGGCTGTTCACATCTCCGCCCACTTTTGGGCATCCAACAATCATCATATTTTCATTCAGGCTGA
ACCCATGACTGTGTGATTCAAGTTTGGGGGTGTGGCCTCAAAGCTGTAAGTTTGGCAGCAGTTTCAATTTCCC
CATAAAAGTCAAAGGTACAATATGATTGGCTGCTGTTGGCCCCTCCCACTTTGTGGGGTTTTTTTAAAAAAA
AAAAAAACCTGAATGTGTAGTCACCCAGTGACTGACTGTGCAAAGTTTGGAAAATCTGGCATCAAACCAATAA
AAATCAATAGGTGAAATCTGATTGGCTGTTGGTGGCTCCGCCCACTTTTCAAACCTTAAACTGCAGTCCCCTA
GTGACCAAGTGTAAAAAGTTTGGGGACCCCGACGTTATTACTGTGAGACTGGCAGCAGGTTGGATTTTCGCCA
AATCAATAGGTAAAATCTGATTGGCTGTTTGGGCATCCAATAATCATCCTATTTTCATTCAGGCTGACCCCAT
GACTGTGTGATTCAAGTTTGGGGAGTGAAGCCTCAAAGCTGTAAGATTGGCAGCAGTTTCAATTTCCCCATTA
AAGTCAATGATTAAAATTTGATTGGCTGTTGTTGGCCCCTCCCACTTTGGGGTCATCCAACAAATGTTGCTGC
TTCATTCGGGGTGACCCCATTATTATATTATTCAAGTTTGGGGGGTGTAGCTTCAAAGCTGTAAGTGTAGCAG
CAGTTTGAAAATCTTCCCTGTCAAAGTCAATGGGAAAATTGGGGGGTTCGGAGCGGCGCCACAAAAAGACGGG
GGGCCGGATCGCTTAGAAAAGCACAAGCAACCTGCTCTGCTATAGGGCGAAGATGTGTGGGGAGTTTGGGTGT
TGTACCCCTAAAACTGTAGGAGGAGTGGCATTTAGAAAATGGGGGGCACTAAGAAGAAGAAGAAGAAGAAGAA
GCGGAAGAATAAGCCAAAGTCGAAGAACAGTCTGTGGGGTTTTCAACCCAACACAACTAACAATGTCAGTAGC
ATTATTGTATAAGTAATTATTATAAGTGTGAAGAGGGTCATGTCATGTCATTTTGTGAGTTAGTACTGTGATG
TCAATGGGGTTGGCTGAAAGAGATTTTGGACAACTCTATATAACTGAAGCAAAAGCCTTTAAAAAAACTGCAA
ATAATACACAATTCTGTTGAGGGACACGGTTGTTTATAAGAAGTCACTGTTAGACTTGATAGTATCATCCCAA
GTGATTTGAATGCCCTGGCTGATTTGTGGCCTTATGGGTGGAATTTGTGGCTACTAGTGATAGACATATTTTG
GAAAAGCATTGGCAGAATGTGTACTGAGACTAAAACTCTGCTAAAAGGAATTCAGTTGCCATTTGGTCTATAA
GAGGGGGGTTAGGTAGAAACAAAGTGCATGGCAGATACATAATATAGTAAACATATTGCTTATCAAAGAAGCA
CTGTTTAACTATGTGAGCTGTAAAGCCTGCAATGAAACCTGCCCTTGGCCTAATTGTTTTGAGCAGCTGATGG
TCTGTAATGAAAGTGCAGAGCAAGAGGGATTCCTATACATGTTTGCCCAAGATATATATCTATAAGTAGACAC
AGATTTTCTCACTGGACAGAAATCATTATAGAGGTGTTTTATGACTTGGGATATAGTTGAGATTTGTTTATTG
TTTTATTAAATGTCATTAATTGTTTATTTGTTTAGGGCAGGGGTCAGGAACCTTTTTGGCCGAGTGAGCCATA
AACACCACATATTTTAAAATGTAATTCCGTGAGAGCCATACAATATGTTTGGCCACAGATGCTGCGGGGCAAG
GTAGGGTGGGTCCCAAGGATGCCGGATGTGATGCAGAGGAGGGCGTGGCCTGTGCTCGGCCAATAGAAGACGT
GTTCTAAGGCTTAGAACACGTCTTCTATCCGCCAAGTGCAGGGGCAAGGTAGGGTGGGTGCGAAGGATACCGG
ATGCGGATGTGATGTGGAGGAGAGCTTGGCCTGCGCTCAGCGGATAGAAGACGTGTTCAAGAGCTTAGAACAC
GTCTTAGAACGTCTTAGAACACGTCCTCTATCCACCAAGAGCAGGCCACGCCCCCCGTTATTTTTTTTATTAA
AGATTTGGCAGCGAGCCAGATGCAGCCATCAAAAGAGCCACATTTGGCTCCCGAGCCATAGGTTCCCTACCCC
TGGTTTAGGGTAATGGGGACAACCAGTGAATGCATATATTGCATTGAATGCAGCATTGTATGTTATCTGTATT
AGAGGACTTACCAATTTGTATACTGTTGGTTATTATTATCAAATCAGTATATTTTGTAAATCAAGGGGTGGAA
TGTAGCACGGTGGATTTTATTTTGATTTTGTAATGATTAACAATAATATATGCATCTGCTTTGACATATGAAT
CAAATCTTAGGTTTGAGTCCAGTGTTCAAGGTACAATGTCTTTGTTCTATGTATATTGTTGTGTAAACAGTAA
ACACAGGTATTGTTACCCAGCAAATAACCTTGGAATAAACAAACAGACAGCATATAGACAATACACTCTATGT
ACAACTTATTTCAGAAACAAATAAGGCTTTCTACAAAGTCTAGTTAATAGGTTTTGTTCATGACAAAAGTTTA
TACACAATATTGCACAAGTCAGCACTTGGTGTTGGGGGTTTGCTAAGAACTCTAGTATAAAAGGGGCCCCCAC

CCTGTGTCAGTAGCTTCGCCTAGGACTCCTGAACGAGTGCCGGGACATTTGGATCATCGCTTGGTTATCGGGA
ACCTGAAGGTCTTGCACCAAAGGTTGTGGGGCTCCCCGATTGGGCTGAATTGATGCAGAACTGGCTGTGCTTG
TAACTAATCATACAAAGATAAATAAATCTATTTGATTCTATTATTACTTGTGTGAGTGTAAGTTATTGCTCAC
TAACAGTCTATCAGAAGGCTTAATCGTTGATCCTGCATGTTTATTAATATTTGTCATTAATGTCAATTATTAC
AAATTATTAATATTAATAAAGGTTCATCCCCTTTATTACA

>Xt-S21_CM004451.2:754654-762085 Xenopus tropicalis strain Nigerian
chromosome 9, whole genome shotgun sequence
TATACATCTGCTTTGATATATGAATTAAATCTTAGGTTTGAGTCAAGTAAAAGCAGCAGATTGTCCAATGTTC
CAGGTACAATGTGTTTGTTCTGTGTATATTGCAGGGTAAACAGTAAACACAGTATTGTTACCCAGCAAATATC
CTTGGAATAAACAAACAGCAGATAGACAATACACTTATGTACAACTTATTTGAGCAACAAATGGGCCTGCTAC
AAAGCTTATAAATTGTATAGTTAGATGTTTTGTTCATGACAAAAGTTTGTATACAATATTGCACAATAAGTCA
GCACTTGGTGTTGGGGGGTTTGCTAAGAATTCTGGTATAAAAGGGGCCCCCACCCTGTGTCAGTAGGTTCCCC
TGGGGCCCCTGAACCAGTGCCGGGACATTGGGATCATCGCTTGGTTATCGGGAACCTGAAGGTCTTGCACCAA
AGGCTGTGGGGCCCCCGATTGGGCTGAATTGTGCAGAACTGGCTGAGCTTGTAACTATAATCATACAGAGATA
AGTAAATCTATTTGATTCTATTATTACTTGTGTGTGTGTAAGTTATTGCTCACTAACAGTCTATCAGAAGGTT
TAATCATTGATCCTGCATATGTATTAATATTTGTTATTAATTTCAATTAATACAAATTATTAATATTAGTAAA
GGTTCACCCTCTTAATTACAGTCCCATAGGGATATTACCAAACTGTATCATATATATATATATAAACACACCC
CTCATGTATAATGTCCCTGTGCCCCTCTATCCCCTCCCCCAGGCTGGGATGTGCCACTCTGTAGGTCAGTGGG
TGCCAGTGGGTTATTAGGGGGTACTCACCCCTCACATATAATGGTGGCTGTGCCCCTCTATCCCCCCCAGGCT
GGGATGTACCACTCTGTTGGTCCAGTGGGTGCCAGTGGGTTATTAGGGGGTACTCACCCCTCACATATAATGG
TGCTGTGCCTCTCTATCCCCCCCCCCCCAGGCTGGGATGTGCCACTCTGTAGGTCAGTGGGGTGCCAGTGGGT
TATTAGGGGTACTCACCCCTCACATATAATGGTGCTGTGCCTCTCTATCCCCCCCAGGCTGGGATGTGCCACT
CTGTAGGTCAGTGGGTGCCAGTGGGTTATTAGGGGTACTCACCCCTCACATATAATGGTACTGTGCCTTCTCT
ATCCCCCCCAGGCTGGGATGTGCCACTCTGTAGGTCAGTGGGTGCCAGTGGGTTATTAGGGGGTACTCACCCC
TCACATATAATGGTGCTGTGCCTCTCTATCCCCCCCCCCAGGCTGGGATGTACCACTCCTGTAGGTCAGTGTG
GTGCCAGTGGGTTATTAGGGGGTACTCACCCCTCACATATAATGGTGCTGTGCCTCTCTATCCCCCCCCCCAGG
CTGGGATGTGCCACTCTGTAGGTCAGTGGGTGCCAGTGGGGTTATTAGGGGGTACTCACCCCTCACATATAAT
GGTACTGTGCCTCTCTATCCCCCCCAGGCTGGGATGTGCCACTCTGTAGGTCAGTGGGTGCCAGTGGGTTATT
AGGGGGTACTTCACCCCTCACATATAATGGTACTGTGCCTCTCTATCCCCCCCCAGGCTGGGATGTGCCACTC
TGTAGGTCAGTGGGTGCCAGTGGGTTATTAGGGGGTACTCACCCCTCACATATAATGGTGCTGTGCCTCTCTA
TCCCCCCCCCAGGCTGGGATGTGCCACTCTGTAGGTCAGTGGGTGCCAGTGGGTTATTAGGGGGTACTCACCC
CTCACATATAATGGTGCTGTGCCTCTCTATCCCCCCCCCAGGCTGGGATGTGCCACTCTGTAGGTCAGTGGGT
GCCAGTGGGTTATTAGGGGGTACTCACCCCTCACATATAATGGTGCTGTGCCTCTCTATCCCCCCCCCCAGGC
TGGGATGTGCCACTCTGTAGGTCAGTGGGTGCCAGTGGGTTATTAGGGGGTACTCACCCCTCACATATAATGG
TGCTGTGCCTCTCTATCCCCCCCCCAGGCTGGGATGTGCCACTCTGTAGGTCAGTGGGTGCCAGTGGGTTATT
AGGGGGTACTCACCCCTCACATATAATGGTGCTGTGCGCCTCTCTATCCCCCCCCCAGGCTGGGATGTGCCAC
TCTGTAGGTCAGTGGGTGCCAGTGGGTTATTAGGGGGTACTCACCCCTCACATATAATGGTGCTGTGGCCTCT
CTATCCCCCCCCCAGGCTGGGATGTGCCACTCTGTAGGTCAGTGGGTGCCAGTGGGTTATTAGGGGGTACTCAC
CCCTCACATATAATGGTGCTGTGCCTCTCTATCCCCCCCCCAGGCTGGGATGTGCCACTCTGTAGGTCAGTGG
GTGCCAGTGGGTTATTAGGGGGTACTCACCCCTCACATATAATGGTGCTGTGCCTCTCTATCCCCCCCCCAGG
CTGGGATGTGCCACTCTGTAGGTCAGTGGGTGCCAGTGGGTTATTAGGGGGTACTCACCCCTCACATATAATG
GTGCTGTGCTCTCTATCCCCCCCCCCAGGCTGGGATGTGCCACTCTGTAGGTCAGTGGGTGCCAGTGGGTTAT
TAGGGGGTACTCACCCCTCACATATAATGGTGCTGTGCCTCTTTATCCCCCCCAGGCTGGATGTGCCACTCTG
TAGGTCAGTGGGTGCCAGTGGGTTATTAGGGGTACTCACCCCTCACATATAATGGTGCTGTGCCTCTTTATCC
CCCCCAGGCTGGGATGTGCCACTCTGTAGGTCAGTGGGTGCCAGTGGGTTATTAGGGGGTACTCACCCCTCAC
ATATAATGGTACTGTGCCTCTCTATCCCCCCCAGGCTGGGATGTACCCACTCTGTAGGTCAGTGGGTGCCAGT
GGGTATTAGGGGGTACTCACCCCTCACATATAATGGTGCTGTGCCTCTCTATCCCCCCCCCAGGCTGGGATGT
ACCACTCTGTAGGTCAGTGGGTGCCAGTGGTATTAGGGGGTACTCACCCCTCACATATAATGTACTGTGCCTC
TCTATCCCCCCCAGGCTGGGATGTGCCACTCTGTAGGTCAGTGGGTGCCAGTGGGTTATTAGGGGGTACTCAC
CCCTCACATATAATGGTGCTGTGCCTCTCTATCCCCCCCCCAGGCTGGGATGTGCCACTCTGTAGGTCAGTGG
GTGCCAGTGGGTTATTAGGGGGTACTCACCCCTCACATATAATGGTGCTGTGCCTCTCTATCCCCCCCCAGGC
TGGGATGTACCACTCTGTAGGTCAGTCGGTGCCAGTGGGTTATTAGGGGGTACTCACCCCTCACATATAATGG
TGCTGTGCCTCTCTATCCCCCCCCCCCCAGGCTGGGATGTGCCACTCTGTAGGTCAGTGGGTGCCAGTGGGTTA

TTAGGGGGTACTCACCCCTCACATATAATGGTGCTGTGCCTCTCTAGCTCCTCCCCCAGGCTGGGATGTGCCA
CTCTGTAGGTCAGTGGGTGCCAGTGGGTTATTAGGGGGTACTCACCCCTCACATATAATGGTGCTGTGCCCCT
CTATCCCCCCCCCCAGGCTGGGATGTGCCACTCTGTAGGTCAGTGGGTGCCAGTGGGTTATTAGGGGGTACTC
ACCCCTCACATATAATGGTACTGTGCCTCTCTATCCCCCCCAGGCTGGGATGTGCCACTCTGTAGGTCAGTGG
GTGCCAGTGGGTTATTAGGGGGTACTCACCCCTCACATATAATGGTGCTGTGCCTCTCTATCCCCCCCCCCAG
GCTGGGATGTGCCACTCTGTAGGTCAGTGGGTGCCAGTGGGTTATTAGGGGGTACTCACCCCTCACATATAAT
GGTGCTGTGCGCCTCTCTATCCCCCCCCAGGCTGGGATGTGCCACTCTGTAGGTCAGTGGGTGCCAGTGGGTT
ATTAGGGGGTACTCACCCCTCACATATAATGGTGCTGTGCCCCTCTATCCCCCTCCCCAGGCTGGGATGTGCC
ACTCTGTAGGTCAGTGGGTGCCAGTGGGTTATTAGGTGGGACACTATGCCCCCAGTAACAGGAGGGGCTCCCA
GTGTGGGCGGCCATGTTGTGGCACTAACATGCAAATGGTACTTAGTGCCCCAGTTTGCTCATTATGTGCCTGT
GTGTGAGAGAGCCCCGCCCTCCAGGGCCAATTAATATGCAAAAGAATCCTCCAATGAGAATCCCAGCTGATTG
GGCTCAAACACTGTTTTGGGACCTGCTGCATTGTTCCTCCAGAAACAAGCCCAGATGTTTATAGATGATTATA
GCTCCCGGATTGTCACAGATTTTTTTTTGGGGGGGCACAGTTTTGTTATATTATGCCACTGAATGCCATCTAT
ACACCATCAATTCTGGTTCTACAATTCTGTATGCAGACAGACAGATAAACCAGTAATGTCCCACCATGACTGT
ATCTTTCTACAACCAACTTGTCTCTATGCCCAGACCAACCTAGCAACACACACATTATAGTGTAAGGGACTCA
CCTGTTATCTGTAAGGCCCCAGTGCCAATCTCTATGGGCTGTGCCAGGCTGGGATGTGCCACTCTGCACACAA
TTTCTGCCCCTTGGTGGGAAAGTATAGATGGGATGAAGTGCAGATACTCAGTACTGATATATGTGCCACATGG
GGCCCTTCCTGGTTTATTCTCAGCCCTGTAATTCTGTGTGTGGGGAGGAGAGATCGGCTCTTCATGCCCAGTC
TTCCTTAGTGCCCAGGTAACTGTCAGGGCATTGGGGAAATAGCCAGAGATATCCCACCGCAATGTCGCCTTTG
TGCCCTGTGTAAGGGTTGGTATGGGCTGCACTGCCACCTGGGGGCGCCATGGGAAACCTGCCAATAGGAGGAA
AGGGAAAGCTTAGAACTGTACATTTCTTTGGGGTTCTGTAGTGATGGGCAGGTCAGCAAATTTACAACCTGCA
CTTGGGCGCTGCCAGCTTGGACCCACCATCAACCCTGGCACCACCATGACTTTTAACCCTCAGTGAACTCAAA
CGTGGGCATAGAATTTCAGGTACATCACCAGAAGGTAGTGAATCTACCTGACTGCCAGGTAAACCCATTGACC
CCATAGGGTTGTGCCGTGTAGTGTAAGGAACTATATGAATAAGGGATTTCTATGGATTGAAATCTAACACCAA
AGATACAAAAGACTGAGTCTCCTCCTAATTGCACTTAATCATATCCCAAATATAATTTATATAGGGACAAATG
TTCTAATTATAATGATAGAATATGATTGCCTATTAGCACTTCTCATAAGTAACAAATAAAACATCTAATAACA
GAGGGCTGCAAGGAATTCAAAGCAGCCATGAACATAGCAATTTTTAACCCACCTTTCAAACCATTGTTTATCA
CTGCCAATAATATAGAGATGCTGAAGTGGGGTTACCTGGGTCTCTGGGGCAGATCTCCCTGGATTCAGATGCC
CCCATGGCCGGGTGGTTCCAGGTGACATGGATCTTACAGTCTGGGAGTCGGAGCAGATTCTCAGGTACCGAAC
ATGAACTTGTAACATTAAAGGTTTGGTCAGAATTATTCTGGTACTGAGGTTCTGAGTGTAGGTTTGTTTGCTG
TGACCCAGTCAGGCAGGTCCATGTGAGTTCTATGGGTTTGGGATAAAAGTTCTGCAGGCTGAGGGTACAGAGA
GCTTCCCCTGTATCAGACAGAGACCATTGCACTGGGATTGGCTGCTTGGGGGCCGCTGGGAGGGGAAAGAAAA
GAAATGTAGGTTACAGGGCAAATCTCCCCCCTTATTATATCAGTGCCACTCACTCACCTCTACTCATATAATA
ACTCTTATTCATTAAATATATATTTCTATTTACTGCACATTCACTGATAGGGGGGTTATATGTGTGCGTCTCA
TACCGACATATATATAATAAATAAAGTGCCCCCTATTGTAAAATATAGAGATATTATAAGTCACCAAGGAGTT
CCATGACCATATAAAGGGACGAGGCTGAAGGCCGAGTGCTTTTACACAGGTCATGGAAGTAAGAGTTGACTGC
TAATATCCTTATATTTTACAATAGGGGGTACTTTACTATACTATTACTATAATATACAAGTTTTAGGGGTGTA
TTTATTATGCTGTGTAAAACGAATTCGCCAAAAAAACGGAGTAAAAAGCTGTGTAAAATAAATGGAAAAGATC
GGTATCTGAACGCCGGATATTACACCGTTATTTTCAATAAGTTCCGGTGGAAGAAAAAACGTGTACAAAAATT
ACGCCGATTTTACGCCGTTTTTACATGGAAAAGCCTGGCGATGTGTGGCGAATTTTCTCGCCGTTTTTGACAC
AGCATAATAAATATGTCCCTTAGTGAGTCATGTGACAGAAATGACATCACTAAGCTCCGATTATAACTGATGA
CATCACTACTCACAGTTTATAATTTACGGTTTGGTCCCGGTAGCATAGTGGATTTTCTATTGATTTTATCATT
TTTAGCAAATTATTAGCAAAATTCCCGGTGAGAGGTGTAAGAAGCTTATTGGTGGTTATAGGAAGCGACTGAT
TTCAGTTATTTTTTCCAAAGGGGGTGCAACCAAATATTAAGGTAAGGGGGCCCAGTAATTCTGTCCGGCCCAT
TTTTGGAGTTCTGTGTGACATTATGTCCAATTTGCTTTTTTCCTCCCTATTTTGTTCTGTTCCAATACCCACA
AAGGGAATAAACACGTGTATAGCAAAACATGCGTTACTGCAATACTTTTCTGTGAGAAATACTTGATTTTCTG
GAAAAATTTCTGGGGTGCCAACAATTTGGGCCATGACTGTATACATCTGCTTTGATATATGAATTAAATCTTA
GGTTTGAGTCAAGTAAAAGCAGCAGATTGTCCAATGTTCCAGGTACAATGTGTTTGTTCTGTGTATATTGCAG
GGTAAACAGTAAACACAGTATTGTTACCCAGCAAATATCCTTGGAATAAACAAACAGCAGATAGACAATACAC
TTATGTACAACTTATTTGAGCAACAAATGGGCCTGCTACAAAGCTTATAAATTGTATAGTTAGATGTTTTGTT
CATGACAAAAGTTTGTATACAATATTGCACAATAAGTCAGCACTTGGTGTTGGGGGGTTTGCTAAGAATTCTG
GTATAAAAGGGGCCCCCACCCTGTGTCAGTAGGTTCCCCTGGGGCCCCTGAACCAGTGCCGGGACATTGGGAT
CATCGCTTGGTTATCGGGAACCTGAAGGTCTTGCACCAAAGGCTGTGGGGCCCCCGATTGGGCTGAATTGTGC
AGAACTGGCTGAGCTTGTAACTATAATCATACAGAGATAAGTAAATCTATTTGATTCTATTATTACTTGTGTG

TGTGTAAGTTATTGCTCACTAACAGTCTATCAGAAGGTTTAATCATTGATCCTGCATATTTATTAATATTTGT
TATTAATTTCAATTAATACAAATTATTAATATTAGTAAAGGTTCACCCTCTTAATTACA

>Xt-S22_CM004444.2:119481285-119488999 Xenopus tropicalis strain Nigerian chromosome 2, whole genome shotgun sequence
TGTAGCACGGTGGATTTTCTATTGATTTTATAATGATTTACAATATATACATCTGCTTTGACATATGAATCAA
ATCTTAGGTTTGAGTCAAGTAAAAGCAGCAGATTTTGTGACAGCTATTGTCCAGTGTTCAATGTACAATGTCT
TTGTTCTATTTATATTGCTGGGTAAACAGTAAACACAGGTATTGTTACCCAGCATGTAACTTATTTGAGAAAC
AAATAAGGTCTGCTACAAAGCTAATAAATTGTATAGTTAGATCTTTTGTTCATGACAAAAATTTGCACATAAT
ATTGCACAAGTCAGAACTTGGTGTTGGGGGTTTTGCTAAGAACTCTGGTATAAAAGGGAGCCTCCACCATGTG
TCAGCAGCTTCGCCTAGGACTCCTGAACGAGTGCCAGGACATTTGGATCATCGCTTGGTTATTGGGAACCTGA
AGGTCTTGCACCAAAGGTTGTGGGGCTCCCCGATTGTGCTGCATTGATGCAGAACTGGCTATGCTTGTAACTA
TAATCATACTAAGATACCGTATATACTCGAGTATAAGCCGATCTGAATATAAGCTGAGGTCCCTAATTTTACC
TAAAAAAACTGGAAAAACTTATTGACTCAAGTATAAGCCTAGGGTGGGAAATGTAGCCTCTACTGCTAAGTTT
CAATAATCAAATAAATAATAAAAGGACACTCAGCATGACCCCCTGTGAACTCTTCATAAATATATCATGTTGG
CAGCTGCAGATTAGGGAATCTAATTATCAAGTACTTGCCATGCTGCTGTGTGCACCCCCCTGTGTGCGCTCCA
ATCGGATCATTTGGTGCTTTAATCATATGCAGTGTGCAATCCCTTCATGTTTGTGCTGTGTGCGCATCCAATC
GCATAGTTGTGCCATTATTTTGTACCATTTGTCACCACTACTTTTCCACATTAGCACCCTTGTGCCACATCTC
TGGCAGTATTACGGGGGTATGTAATAACACATTTATGTGACCACAAATTTGTTGTAAATTGATAGTTAAGTGA
TAGTTGCCACCAATGACCCCCCCCTTGAGAATTAAAGGGTAAACAGCATGGGCCAGCTATTTGCTAGGATTAG
GTGCTGGGTGCAGGTATTAGCACCTGGGTGCAGTTCAGTGAATTAGCCCCATATCAGGAACGCACTAATTACT
TAGCAAAACTGCACGCACCCCCCCCCGTGCCCCGTGGACGTGCGTGCGCGTATGCATCTTCTTTCTACCCCC
CCTACCCTTATACCCCCATGTGTCACTGACATAAACTGATCAGGGAATATAAATGTTCCCCGTACCCCCAGCC
TGATAGTTTGGTTCCCCCACAAATAAGGTAAGCGCAGCGGCCCCGCCATTACTATAAGGGAGAGATTACCATT
GCTGCCTGTCAATGAGAGATGCACTAATGCTGATGGGTCTATCTCTCTCTGTCTCTCTCTCTGCCGACTGTCT
AGGTGGCTGTGGGTGCCGGATTGGGCCGCTCCCTGTGTACCCAGCAAATCCCCCCACCCCCCCGTGGACGTGC
GTGCATGGACAGACGTCCAGGAGGGGGGGTTGCAATCAAGGAATATGAATGCCAGCAAGGTACATCCATATAC
TCGAGTATAAGCCAAGGCTTACTTTTTCAGCACATTTTTGGTGCTAAAAAACTCGGCTTATACTCGAGTATGT
ACGGTAAGTAAATCTATTTAATTCTATTATTATTTGTGTGCGTGTAAGTTATTGCTCACTAACAGTCTATCAG
AAGGTTTAATCATTGATCCTGCATATGTATTAATATTTGTCATTAATGTCAATTAATACAAATTATTATTATT
AATAAAGGTTCACCCCCTTTATTACATTTGGCGTAGTTGGCAGGATTAACCCTTTGGAAGCTGGGAAGTGCAG
TAATAACTTGTAAAGAAATTGTAGAATTTATAGCGCAGCTTTGTATGGTAGTGTAGTGGCAGTATTGTAGTGG
CTTGTGCAGGGCAAACATACTGTTGAAAGTTAGCAATAATGTTCTGTTGGTTGAAAAACAAAGTTAGAGGGGT
AGATGATCAAACGAGCAACCTTCCCACGTAGGCTAGTTCCGGACCCCACGTTGCTCGGGATATGATTGGAGAT
CAGCAACTCTCTCAGGTGACTCACCCCAATCACCTATTGCTGAATTTGGAATTGTCTATGTGTTCCAAGGAGC
AGAGGTCCTAAGCACAATTGGGTTTGTTGTGTTTTCAGGCGCTCAGGGAGGAGACAATCCAAAAGGAGGCAGT
TGAGCTTGAGCTGCAGGCAGTTAAAGATCAGCTAGCCACAATGAGAGAATGTTTGGGCTCCTCATTAGCACAC
AATGAGGAGATAGAGACAGAATTTACAAAAATATGTGGTAAGAACTATGAGTAAAAAGCACCATAGAAAAGGG
ACAGAACAAAAAGGAAATGGTGTATTAGTTAGATTAAGAGCTCTTGTAAGTAAAAGAGACTGAGGGAGTGATT
TATTTCTCAAGAAACAAAATAAATGTTACTGTCACGGTCAGTATCCCAAAACTCAGAACAGTTCCCAAGGGCC
TGGTCTCGGCTCACGCTTCTGCCTATAACCGCCGCCTTTCACGTCGGGAGGAGCCCTTCGCTACTCGGATGCC
GCCAGGTCTTATAGTGAGGAGCCCAGGGAAAGAGTTCTGAGCAGGCAAGGGAACCAGAACCGTTCTATAGGGA
GAATGGGCACAAGAAGACAAAGTCGGAGATCAGGCCGGGGTCAGCAACGTTTACAGAGAGTAAAGTACCAAAT
CAGAGTCCAAAAGGGTAGTCAGAGGTCAGGCCGGGGTCAACAACAATAAGGCAGCGCGGTACCAAACAGAATC
CGGAAGAGTAGTCAAAATTCAGGCAAAGGTCGGTTCAGGCAGCAGTACAGGAAGGTCAAAGACAGGCAGAGGT
CAAAACAGATCAGCAGAACAGGAATAACACACCCAGGAACTCACAGGAGGAAAACCTACGTTGGGCAACCATT
ATGCTGCAGAAACCACTTAAATATTCAAATTTGGCGCCATAGTGACGTCACACGCAGCGCCGGATGATCGATG
CAAGAGCGCATGCGCCGGACGCGCGTATGGCTCAGTGTTTAAAGGGCTGCGCGCCGAGCGTGCGCACCCCTAG
GAAGAGTCGGCGGGCGTCCCCGCAACGGAGGAGGCCCCACTAGACCGCCAGGTACCCTTACAGTTACATTGTG
TTAAATTGTACTGTATATTAATGTCATATGTTTTTCTGTTTCAGGATTTAAGAATCTGATTATTTTGAGTTAA
TATGGTTTTTAATTACAGCTATCAAGAGTTGATGGATCCATTATTTTTGACACTTTTTGTTCTGTTTTTTAAG
TGCACAAATTTGTGCGTAAAGGCCTTAAGACATGTCTGTTTTTGTAAAATAAAAAAGTTAAAATGAAACTTGA
AAGTTTTTAACACTTTTTGTTGTATGTTTCTGAGAATGTGTGGGCCCACATGCATGTTGTTATGGTTTGTCTT

GTTTTAAATGTGACAATGACTAAAGGTGGCCATACACGGGCCGACTATAGCTGCCGATATCGGTCCCTTGGAC
CGATTCGGCGGCTAATCGGCCCGTGTATGGGGAGAGCAGAGCGGCCTGGCCGACCGATATCTGGCCTGAAATT
GGCCAGATCTCGATCGGGCAGGTTAGAAAATCTGGTCGGATCGGGGACCGCATCGGCTCGTTGATGCGGTCCC
CGATCCGACTGCCCCATTGCCGCCCTGGGGCCAAACGATCGGATTATTATTTTTTTTACCTAAATGGTCCCCG
ATATCGCCCACCCGTAGGTGGGGATATCGGGGGAAGATCCGCTCGCTTGGCGACATCGCCAAGCGAGCGGATC
TGCTCGTGTATGGCCACCTTTAAATGTGACAGTGACTTAAAAGGTTTTTGACAATTATGTAAAAATTAAGAAA
GTTGATAAAGTTGACAAAACAGATCTTAAATGCTAAGAACTGATGTTGCCTTTATCTTTTTTTTTTTCCATAG
ATGTTTATCGCGGATATAACAAGCGCATTGTTGATAGCAAAACAATGGACAGCAAACAAGGATTTGGACATTG
TTAATCTTCAATGGACATCAGTTCAGAGTATAAAGAACCTGGAATTAATTTTAGTGGTGACCTGGAAACTTAA
ATGGACATTGGTTTTGGGACTTAATGAGTAATATTTATGTTTTGTTCAATTGTTTCTACAGACTGAAGATAGA
TTTGCATACATTATTATACCAATGGTAATTGGAATGTTAGGGCTTGTCACATGAGCTTTAAAGTGCTAGTTAG
TGAGTTTTTGAGCACAGGCAGCATGGATGAGTTTATAAAAAAAAAAAAAAAAAAAAAAGCAAACCACTGGCAAT
CTGTGAATTTTGGTAAATGCCAGAGGGGCTGCTGTAAGATGCCACAGACAGTCACTATTTAGTGGGCTGGTGG
GGGGCTGGTTGGGGCTCTGTGTACTTGAAATGCTAGGGCCTATTTTGAAATTATGTAAGTCTGTGAAACACAC
TGATAAAACAGAAAGAGTGTAATTTATTATCCTGAGGGAAAAAGTGCTATGTGAAACTAACAATGTATGTAGA
CAGGGCCGCCATCAGAAATCATGGGGCCTCTCACAACAAAATTTTCTGGGCCCTCCTCCTCCCACGCCCTGCC
CCCCAATTCCATTCCCCCCCAGTGATCCCTCCCATCAGCACAAAGGACACACAAACATTGGTAGCCAGGGCCC
CCTAATACATTGGTGCCCTGCAGCAGTGCCCCCTAATACATTGGTGCCAAGAGCAGTGCCCCCTAATACATTG
GTGCCAAGAGCAGTGCCCCCTAATACATTGGTGCCCTGCAGCAGTGCCCCCATAATACATTGGTGCCCTGCAG
CAGTGCCCCCATAATACATTGGTGCCCTGCAGCAGTGCCCCTAATACATTGGTGCCAAGGGCAGTGCCCCCAT
AATACATTGGTGCCCTGCAGCAGTGCCCCCATAATACATTGGTGCCCTGCAGCAGTGCCCCCATAATACATTG
GTGCCCTGCAGCAGTGCCCCTAATACATTGGTGCCAAGGGCAGTGCCCCCTAATACATTGGTGCCAAGGGCAG
TGCCCCCTCTAATACATTGGTGCCAAGAGCAGTGCCCCCCTAATACATTGGTGCCCAGCAGCAGTGCCCCCCT
AATACATTGGTGCCAAGGGCAGTGCCCCCTAATACATTGGTGCCAAGAGCAGTGCCCCCTCTAATACATTGGT
GCCAAGAGCAGTGCCCCCCTAATACATTGGTGCCCAGCAGCAGTGCCCCCCTAATACATTGGTGCCCAGCAGC
AGTGCCCCCATAATACATTGGTGCCAAGAGCAGTGCCCCCTCTAATACATTGGTGCCAAGAGCAGTGCCCCCC
TAATACATTGGTGCCCAGCAGCAGTGCCCCCATAATACATTGGTGCCAAGAGCAGTGCCCCCATAATACACTG
GTGCCAAGAGCAGTGCCCCCCTAATACATTGGTGCCCAGCACCAGTGCCCCCATAATACATTGGTGATCAGTG
TGCCCTTCAGCCCCCCCTAATACATTGGTGCCAAGAGCAGTGCCCCCCTAATACATTGGTACCCAGCAGCAGT
GCCCCCATAATACATTGGTGCCCAGCAGCAGTGCCCCCATAATACATTGGTGCCCAGCAGCAGTGCCCCCATA
ATACATTGGTGCCCAGCAGCAGTGCCCCCATAATACATTGGTGCCCAGCAGAAGTGCCCCCATAATACATAGG
TGATCAGTGTGCCCTTCAGCCCCCCCTAATACATTGGTGCCAAGAGCAGTGCCCCCATAATAAATTGGTGGTC
AGTGTGTCCCGCAGCCCCCCCTAATACATTGGCGACCTGCAGCAGTGCTCTTCGGCTCCTCCAGCATCTTCAG
CAGCACCCTTAGATCCTTCCCGGGCGACGCCTCTGCACGTCTGTCTCCACGCGAATGGAGACAGGCCCTTTTA
AAAGGTTCCAACCAACCAATGACAGGGCCCGTTTTTTATTAAAGGGGGCCCAAGCGACTAAGAAAACTGTAGC
ACCGCCGGGCCCCCTTTAAAAGTAAAAATTGCCCGGGCCAGGTACAGCTGTACCCGCTGTGCCCCCCTGATGG
CGGCCCTGTATGTAGACAAGCATTATGGTATAATTATTGTAAGTGTGAATAGGGTAATGTAATTTTATGAGTT
AGTACTGTGATATCAATGGGGACAACTCTATATGATTGTGAAGTGAAAGCCTTTTAAAGACTACAAGTTAATA
TACAATTCTGTTGAGAGACACGTTAGTTTATAAGAAGTCACTGTTAGACTTGATAGTATCATCCCAAGTGATT
TGAATGCCCTGGGTGATTTGTGCCCTTATGGGGTGGAATTTGTGGCTACTAGTGATACACTGATATTTTGGAA
AAGCATTGGCAGAATGTGTACTGAGACTAAAACTCTGCTGAAATGAATTCAGTGGCTATTGCTATATATAAAA
GGTGGTTAGGTACAAACAAAGTGCATGGCAGATACATAATATAGTAAACATTTTGCTTATCAAAGAAGCACTG
TGTGAGCTGTAAAGCCTGCAATGTAACCTGCCTTTGGTCTAATTGTTTTGAGCAGCTGATAGCTTATAATGAA
AATTCAGAGCAAGAGGGGTGCCTATACATGTTTGCCCAAGATATATATCTACAAGTAGACACAGATTTTTCTC
ACTGGACAGAAATCATTACAGAGGTGTTTTATGACTTGGGATGCAGGTGAGATTTGTTTAATCTTTTGTTAAA
TGTAGTGTCCTTCAGTCAGAATTACAGTCATTAATTGTTTATTTGTTTAGGGTAATGGGGACAACCAGTGAAT
GCATATATTGCATTGAACGCAGCATTGTATGTTATCTGTATTAGAGGACTTACCAATTTGTAAACTGTTGGTT
ATTATTATCAAATCAGTATATTTTGTAAATCAAGGGGTGGAATGCAGCACAGTGGATTTTCTATTGATTTTAT
AATGATTTACAATATATACATCTGCTTTGACATATGAATCAAATCTTAGGTTTGAGTCAAGTAAAAGCAGCAG
ATTTTGTGACAGCTATTGTCCAGTGTTCCATGTACTATGTCTTTGTTCTATTTATATTGCTGGGTAAACAATA
AACACAGGTATTGTTACCCAGCAAATATCCTTGGAATAAACAGACAGCATATAGACAATACACTCTATGTACA
ACTTATTTGAGAAACAAATAAGGCCTGCTACAAAGCTAATAAATTGTATAGTTAGATCTTTTGTTCATGACAA
AAATTTGCACATAATATTGCACAAGTCAGCACTTGGTGTTGGGGGTTTTGCTAAGAACTCTGGTATAAAATGG
AGCCTCCACCATGTTTCAGCAGCTTCGCCTAGGACTCCTGAACGAGTGCCGGGACATTTGGATCATCGCTTGG

TTATTGGGAACCTGAAGGTCTTGCACCAAAGGTTGTGGGGCTCCCCGATTGTGCTGAATTGATGCAGAACTGG
CTATGCTTGTAACTATAATCATACTAAGATAAGTAAATCTATTTAATTCTATTATTACTTGTGTGCATGTAAG
TTATTGCTCACTAACAGTCTATCAGAAGGTTTAATCATTGATCCTGCATATGTATTCATATTTGTCATTAATG
TCAATTAATACAAATTATTAATGTTAATAAAGGTTCACCCCCTTTATTAC
