# Supplementary material for: A novel class III endogenous retrovirus with a class I envelope gene in African frogs with an intact genome and developmentally regulated transcripts in Xenopus tropicalis
Source: Retrovirology. 2021 Jul 14;18:20. doi: 10.1186/s12977-021-00564-2 (PMC8278194; doi:10.1186/s12977-021-00564-2)
Supplement: Supplementary file 4 — Additional file 4: Figure S4. Dot-plot and alignments compare the XtERV-S and XlERV-S genomes. Arrows indicate the site of insertions in the XlERV-S proviral sequence. Dots in the alignment represent identities. Shaded portions represent different regions of the proviruses – LTR (grey), PBS (red), gag (yellow), pol (green) and env (blue). [file 12977_2021_564_MOESM4_ESM.pdf]

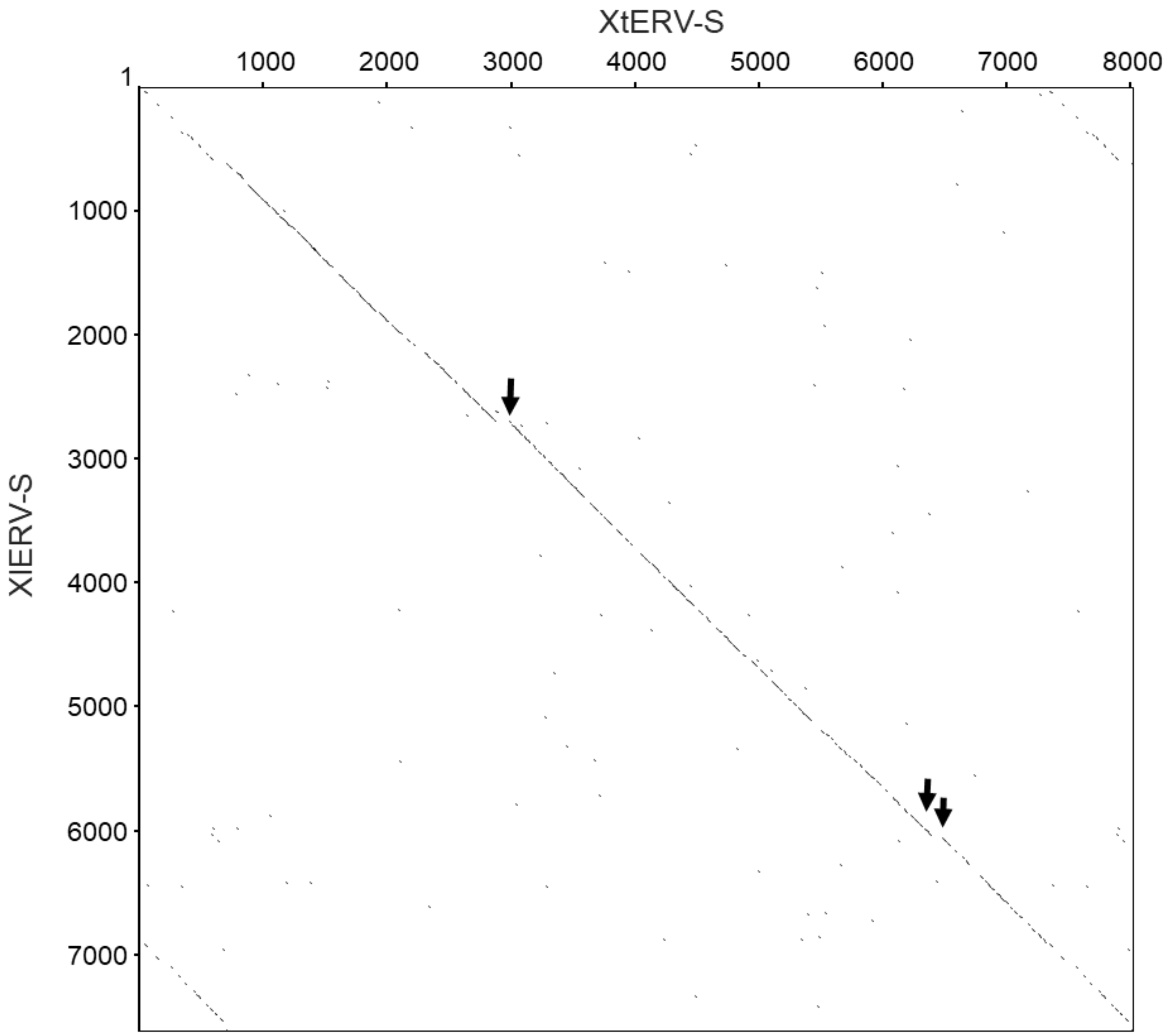

XtERV-S
1000
2000
3000
4000
5000
6000
7000
8000
1
XlERV-S

```
                  10        20        30        40        50        60        70        80        90       100
          ....|....|....|....|....|....|....|....|....|....|....|....|....|....|....|....|....|....|....|....|
XtERV-S   TGTAGTGCAGTAGGTTTCTTTTACTAtgatttgt-aTGATTTACAATTTATACATGCTTTAATATGTGTATGTATATATATATGTATATGGTTTAGTTAG
XlERV-S   -----------.ATG.AA.ACA.GGG.T....T.T.................A...TC.GTT.TAA.A...-....C......AC....ATA.......A
                 110       120       130       140       150       160       170       180       190       200
          ....|....|....|....|....|....|....|....|....|....|....|....|....|....|....|....|....|....|....|....|
XTERV-S   TAAGTTTAATCAAGCAATTC-TAGAGATA-AGGGGAGTCCAGTGTCCTTGTACATATATTGTTGGGTAACAATACCTGCGTTTATTATGTAACAATAAAC
XLERV-S   .GGA..AGTCAGTA.G...AA....A.CCT.CT.AG................TG.GCC...C.A.....--.C.GTCATAGCC...G.T..TTGT..T.T
                 210       220       230       240       250       260       270       280       290       300
          ....|....|....|....|....|....|....|....|....|....|....|....|....|....|....|....|....|....|....|....|
XTERV-S   AATGACTATAGAC-TGTTTACCCAGCAAATA----------CAACAAGCGGACGGCAGGCAGACAATGCCCAATGTATTTAAATAAGAACTTGAGGTATA
XLERV-S   .G.A.AC....GTA.TG.......C......GCCCTAATAAT.C..G..A...AAT..C.TC.T..-A......T.....G.T.....C..G.CT.A.-G
                 310       320       330       340       350       360       370       380       390       400
          ....|....|....|....|....|....|....|....|....|....|....|....|....|....|....|....|....|....|....|....|
XTERV-S   AAGTTTGGTATCTGATCAGACACAATGGCTGACCATTTGTTAAACAATATTGTTCAAGTCAATAGTTGGGCTTGGGGGTTTTTCCTAAGAACTTTTGGCA
XLERV-S   .G.CA.AAAC.T..C.G..---T.T.T....T---...T.G.......T...CCT.T....GC.......G.....T...AAA.--.....A...G....
                 410       420       430       440       450       460       470       480       490       500
          ....|....|....|....|....|....|....|....|....|....|....|....|....|....|....|....|....|....|....|....|
XTERV-S   TAAAAGAACGGCCTCTGCCTTGGGTCAGGAGCTTCGCCTAGGACTCCTGAACGAGTGCCAGGATATTGGATCATCGCATGGTTATCGGGAACCTGAAGGT
XLERV-S   ......C..A....T......A.............A.T.T.A.........A..C....G...A....A.C...T..T..A...............G.T.
                 510       520       530       540       550       560       570       580       590       600
          ....|....|....|....|....|....|....|....|....|....|....|....|....|....|....|....|....|....|....|....|
XTERV-S   TTTGCACC-AAAGGTTGAGGGGCTCCCCGAGTTTGCTG---GATTAATGCTGAACTGGCTATCTTTGTAACCACAAAACCGTACGAAGCTAAGTAAATGT
XLERV-S   CA..T...C.....C..CA.A.........T.G.....ATT....G..............T.TCC.....TT..C.--T.A...........A.......

                 610       620       630       640       650       660       670       680       690       700
          ....|....|....|....|....|....|....|....|....|....|....|....|....|....|....|....|....|....|....|....|
XTERV-S   TTT-AAATTCTATTACTATGTGTGTGTGTAAGTTATTGCTCATTTAATAGTTTTTCATCAGAAGGTTTAATCATTGATCCTGTTAATATAAATAATATTG
XLERV-S   ...T.C......CCTTC.C--------------------------------------------------------------------------------T
                 710       720       730       740       750       760       770       780       790       800
          ....|....|....|....|....|....|....|....|....|....|....|....|....|....|....|....|....|....|....|....|
XTERV-S   ATAAAGGTTAACCCCTTTATTACATTTGGCGTAGTCGGCAGGATTAACCCTTTTGGTGCTGTTAAATGCTGCAATAGCTTGTGCAGGAACTGTAGAATTT
XLERV-S   .........C...............A....A.....T....................A....G..G...A.T....A...A.-----------.......

                 810       820       830       840       850       860       870       880       890       900
          ....|....|....|....|....|....|....|....|....|....|....|....|....|....|....|....|....|....|....|....|
XTERV-S   ATGTTTTAG-TTTGTATGGTAGTGTAGTGGCAGTATTGTAGTGGCTTGTGCAGGGCATACATACTGTTT-AACAGATA-------GCAATAATGTTCTGC
XLERV-S   ..AG.AC.AC............--.............A..........G........A.T.........T..AG....TATTTAA.G.........A...

                 910       920       930       940       950       960       970       980       990      1000
          ....|....|....|....|....|....|....|....|....|....|....|....|....|....|....|....|....|....|....|....|
XTERV-S   TGGTTAAAAAACAAGGTTAGAGGGGTAGATGATCAAACGAGCACCCTTCCCACGTGGGCTAGTTCCGGACCCTGGACCCACGTTGCTCGTGAGTTGGTTA
XLERV-S   .................A.....................G....................................................A.......

                1010      1020      1030      1040      1050      1060      1070      1080      1090      1100
          ....|....|....|....|....|....|....|....|....|....|....|....|....|....|....|....|....|....|....|....|
XTERV-S   AGTATCAGCAACCTTTTCAAGTGACGAACCCCGGTCACCAGTTGCTGAATTTGGATTTGTCTCGGTATTCCAAAAAAGAGCAGAAAGCTGCAGCACAAGT
XLERV-S   .A.................G.....T....................A.....A.................T.G.C..........G..............

                1110      1120      1130      1140      1150      1160      1170      1180      1190      1200
          ....|....|....|....|....|....|....|....|....|....|....|....|....|....|....|....|....|....|....|....|
XTERV-S   GGGTTGGTTGTGTTTTCAGGCGCTCATGGAGGAGACAATCAAAAGGGAGTCAGTTGAGCTTGAACTGCAGGCAGTTAAAGATCAACTGGTTGTGTTAAGA
XLERV-S   ....................T..................T.....A...A..A..........................A..........G..--.....

                1210      1220      1230      1240      1250      1260      1270      1280      1290      1300
          ....|....|....|....|....|....|....|....|....|....|....|....|....|....|....|....|....|....|....|....|
XTERV-S   GAATGTTTTCGCTCCTCAGAAAAGTATCGTGAGGAGATGCAAACAGAGTTTTCAAATTATGTAGTAAAAACAGTAAATAAACAGCACAGAAAGAAAGGGA
XLERV-S   ........C...............C.CT...........G...-------------..................T.........TG..A...........

                1310      1320      1330      1340      1350      1360      1370      1380      1390      1400
          ....|....|....|....|....|....|....|....|....|....|....|....|....|....|....|....|....|....|....|....|
XTERV-S   GGAAATCACCAGGAAATGGTGCAAAAGTTAAAGTAAGGGCTCTTGTAAATAAAGGAGACTGGGGGGAGTTAGCCCAATTGTCAAGCGCAGACTCAGATGA
XLERV-S   ..T.............G.T.....................A.....G.............A...............G........T..............

                1410      1420      1430      1440      1450      1460      1470      1480      1490      1500
          ....|....|....|....|....|....|....|....|....|....|....|....|....|....|....|....|....|....|....|....|
XTERV-S   AGAGATTGAGATAGGAGATGATATAGAAGAAGAAGAA------TTAAGACCAATTGTAACTACCAAACAAAGGAGAGAAGTCACACAAAGAGCAGGCACT
XLERV-S   .........A...........................GAAGAA.........................................C.T.......T...A.

                1510      1520      1530      1540      1550      1560      1570      1580      1590      1600
          ....|....|....|....|....|....|....|....|....|....|....|....|....|....|....|....|....|....|....|....|
XTERV-S   GATAATAGAGATCAGGATTATAGAGGAAGTGCAGAAGCAGCAGAACAGCAATATACAGAGGAAACTCGGTCATATACTGCAGGAGAATTAACAGAATTGG
XLERV-S   .T...C...................C..A...............G.......................T...........C.....T........GC...

                1610      1620      1630      1640      1650      1660      1670      1680      1690      1700
          ....|....|....|....|....|....|....|....|....|....|....|....|....|....|....|....|....|....|....|....|
XTERV-S   GTCAGAAATTTGCCCAGAAAGGTGGTGAAACTATTCTAGAATGGGTTTTGAGAGTGTGGGATTTGGGTGGGGATGGTATCATGCTCACTAGCAGGGAGGC
XLERV-S   ....A........T........A........A.G.T.T..........................CA....A.................C.A.........

                1710      1720      1730      1740      1750      1760      1770      1780      1790      1800
          ....|....|....|....|....|....|....|....|....|....|....|....|....|....|....|....|....|....|....|....|
XTERV-S   GATAGGACTGGGGAGTATTGCAAAAGACCCAAGGGTACGGCTTTATCTCAGGAGAGCCCGTGATACTACTGTGGCTTTTTCCCTGTTACATTTGATCAGG
XLERV-S   A......A.A.....C..............TCA...GA.A.................AT.........A....A.............G........A...

                1810      1820      1830      1840      1850      1860      1870      1880      1890      1900
          ....|....|....|....|....|....|....|....|....|....|....|....|....|....|....|....|....|....|....|....|
XTERV-S   GATTCAGTCCTACAAGTATATGATACCCCCACTGAAATGATGGATTCTACCTCATGGCATACTATAGGGGAGGGGATACAGAGGTTAAGGGAGTATGGAT
XLERV-S   ........TT.........................................C.....TG.TT............A.................A.......
```

```
                1910      1920      1930      1940      1950      1960      1970      1980      1990      2000
          ....|....|....|....|....|....|....|....|....|....|....|....|....|....|....|....|....|....|....|....|
XTERV-S   GTGTGAGTGGTTTGATTGCTGATCCATTAGCTTCAGCACCCCCAAATAAACCTACTGTAAAAGAGACATGGTCAGGACCAGACATGGCTCCCTTCACTGC
XLERV-S   ......................C..........TG........T....C.------------.............................T........

                2010      2020      2030      2040      2050      2060      2070      2080      2090      2100
          ....|....|....|....|....|....|....|....|....|....|....|....|....|....|....|....|....|....|....|....|
XTERV-S   ACACATGAAAAATAAGCTGCTAGCAGGATCTCCCACCCATTTGAAAGCTCCATTGTTCACAATGTTAAGTGCTTTAGATGCCAAGCAGGGTGTAATACAT
XLERV-S   ............C......................TT.........T.C........T..T...........---------...A...............

                2110      2120      2130      2140      2150      2160      2170      2180      2190      2200
          ....|....|....|....|....|....|....|....|....|....|....|....|....|....|....|....|....|....|....|....|
XTERV-S   GAAGCTGCAACATTGCTGGGACAGTTGGGAGAGTTAGAACGTATAGGGGAAAAAGTATCTAGACACCGTGTAATTGCAGAAAGGGGTACAGAAAGATTAA
XLERV-S   ...............T.............C..C..........C........G...TC.......A.........T....G...................

                2210      2220      2230      2240      2250      2260      2270      2280      2290      2300
          ....|....|....|....|....|....|....|....|....|....|....|....|....|....|....|....|....|....|....|....|
XTERV-S   AAATTAGTCGGAGACAGTTGTTTTTTGATTTACTTCAAAGTGGAG-TACCAAAGCCTGAAATTGATGGTATGCCCACAGTTGATTTGTTGAAACTCTGGA
XLERV-S   ....C...-------------...A......G...........G.G........TA......A........T---------------------------.

                2310      2320      2330      2340      2350      2360      2370      2380      2390      2400
          ....|....|....|....|....|....|....|....|....|....|....|....|....|....|....|....|....|....|....|....|
XTERV-S   AACAACTAAAACACAAGGGGAGGCTGAAATCAACCCAACCAAAAAA-GGTGGCAACCAGTGATATAGAGGATGTCCCTGTTACTCCTTCAGCTCCTCTCA
XLERV-S   .....T.T.....A.........T.A...-................A...A.......................T.....C.......T---------T.

                2410      2420      2430      2440      2450      2460      2470      2480      2490      2500
          ....|....|....|....|....|....|....|....|....|....|....|....|....|....|....|....|....|....|....|....|
XTERV-S   ATAAAGGGAAATTTAACCCATACAGGGAAGTGGCTGAGGAATTGGCAAGACATAAGGAGAGGAGTTGGGAGGTGCCCAGTCCTATAGATTGACAGGATGG
XLERV-S   CA..........C..C..........A.............C.....T...G...C........................C..C.................

                2510      2520      2530      2540      2550      2560      2570      2580      2590      2600
          ....|....|....|....|....|....|....|....|....|....|....|....|....|....|....|....|....|....|....|....|
XTERV-S   CAGATCCTCTGTGCCACTGGTAAAAATTAACACAATGAAACCAGGGGATCAACGCCCATATATATCAGTGGCAATATATTGGCAGGGACAGTCAAAACCA
XLERV-S   .....................T..........................C........T...GC.....---...................A.....G...

                2610      2620      2630      2640      2650      2660      2670      2680      2690      2700
          ....|....|....|....|....|....|....|....|....|....|....|....|....|....|....|....|....|....|....|....|
XTERV-S   CAAACGGTTACTGCACTTCTAGATACAGGGGCTGAAGTAACTTTGTTTCATGGAAATCAAAAACGACAGAGTGCAGGAATGGTAGCTATAGAGGGATTGG
XLERV-S   ....A.........TG.....A.----.CA..A............................C......................................

                2710      2720      2730      2740      2750      2760      2770      2780      2790      2800
          ....|....|....|....|....|....|....|....|....|....|....|....|....|....|....|....|....|....|....|....|
XTERV-S   GAGGTCAACATACACTGGCTCACAAGGTTCACTGCAGATTACAGATTGGAAAGGGTCCCATTTTTAAAGCACGTGTGTTAATATCTGAGCTACCTGATAA
XLERV-S   ...T.T........T...................A......................A.............T....A.........C.............

                2810      2820      2830      2840      2850      2860      2870      2880      2890      2900
          ....|....|....|....|....|....|....|....|....|....|....|....|....|....|....|....|....|....|....|....|
XTERV-S   CATACTAGGAATGGATATTTTAAGGGGCCAGACTATCCATACTGATGTAGGCATGTATGTATTTGGTACCTCAGTTCGTTCTTTTTCTGTTTCTGCAGTT
XLERV-S   ....T......................T...................C...A.........................A..A.........C.........

                2910      2920      2930      2940      2950      2960      2970      2980      2990      3000
          ....|....|....|....|....|....|....|....|....|....|....|....|....|....|....|....|....|....|....|....|
XTERV-S   AAACCAATTTTAAGGGG----------ACATGCCAAGT---------GGACACCAGTGTACATCCCTCCGCCCAAGCACCC-TGTCAATCTCAAGCAGTA
XLERV-S   .....CCCC.AGG....TTTCCCTTCAG.G...TGG..CTCGGAGCA..GG..TG.G....-.GGAG.A.G.TGG...GT.GCAC..GGTCT.G.AGCCG

                3010      3020      3030      3040      3050      3060      3070      3080      3090      3100
          ....|....|....|....|....|....|....|....|....|....|....|....|....|....|....|....|....|....|....|....|
XTERV-S   TCGCATACCCGGAGGCCACAAAGAAATTACTGAA------------------------------------------------------------------
XLERV-S   GTCTGGGAG.A......G..GG.GGGA..GCAGTCATCGGAGGGAACACAGGAGGGGTAGGGAGAGGGCAGTCGGTTCTCCTGCTCATGTTTGTGTTTTT

                3110      3120      3130      3140      3150      3160      3170      3180      3190      3200
          ....|....|....|....|....|....|....|....|....|....|....|....|....|....|....|....|....|....|....|....|
XTERV-S   ----------------------------------------------------------------------------------------------------
XLERV-S   TCTTCTGGCAGCGGTCTGCCCCTAGGAGCACTGGACGTCGGGGAGAAGGCGATGGCAGGAGTGACTCACATCAGCGGCCAGTGAGTCACGGCCGAGGAGT

                3210      3220      3230      3240      3250      3260      3270      3280      3290      3300
          ....|....|....|....|....|....|....|....|....|....|....|....|....|....|....|....|....|....|....|....|
XTERV-S   ----------------------------------------------------------------------------------------------------
XLERV-S   CGTCAGCATAGCGAGGAAAGGAGCACGGCCCGCAGGATGTGGCTTCAGGTGGGCCAGACGGTGAGTCCTCCAGCATGGTGTCAGTGTCACTCAATGCTAT

                3310      3320      3330      3340      3350      3360      3370      3380      3390      3400
          ....|....|....|....|....|....|....|....|....|....|....|....|....|....|....|....|....|....|....|....|
XTERV-S   ----------------------------------------------------------------------------------------------------
XLERV-S   GGGTTTGTTGCAAGGGCTGAAAGACTTTTTAGGGAAATGGGAATCGAGGGAGCCACGGGACAGGGTGGCAAGGGTGTGGGTCAGAAGTGAGGACAGAGGG

                3410      3420      3430      3440      3450      3460      3470      3480      3490      3500
          ....|....|....|....|....|....|....|....|....|....|....|....|....|....|....|....|....|....|....|....|
XTERV-S   ----------------------------------------------------------------------------------------------------
XLERV-S   ACTGGTGAGTCGATATGGTCGGAGTCCAAGGTTCCGGGATCGAATAGGCACGCCCCTGGGGCATCAGGAGGGGTGGACACGGGCAGGGTTGTCGCGACGG

                3510      3520      3530      3540      3550      3560      3570      3580      3590      3600
          ....|....|....|....|....|....|....|....|....|....|....|....|....|....|....|....|....|....|....|....|
XTERV-S   ----------------------------------------------------------------------------------------------------
XLERV-S   GCCAGGATGACCAAGGGGTTAAAGGGAGCAATGAGCAGCCAGTTCCGGGAAGCATTCCAGAGGCGGCACAGAGGGGGGCTGATGTGTCATTTGCAGGCCC

                3610      3620      3630      3640      3650      3660      3670      3680      3690      3700
          ....|....|....|....|....|....|....|....|....|....|....|....|....|....|....|....|....|....|....|....|
XTERV-S   ----------------------------------------------------------------------------------------------------
XLERV-S   CCTCGGATCACACGTAAAAAAGTAGGTTAAGGAAAAGATTTGGAAAGGGGAGTTTGTGGAAATTTTTTCCCTTCTTCCCCTGGAGGAATCGGTGGAGTTA

                3710      3720      3730      3740      3750      3760      3770      3780      3790      3800
          ....|....|....|....|....|....|....|....|....|....|....|....|....|....|....|....|....|....|....|....|
XTERV-S   ----------------------------------------------------------------------------------------------------
XLERV-S   AAGGATGAGGAAAAAAAGGACGGGAAGAAGGAGGAGGAGGAGGAGGAAAGAAACAAGCGTTATAGGAAAATCACCAAGACATTTGGCAGCTGGCTTAGGG
```

```
                3810      3820      3830      3840      3850      3860      3870      3880      3890      3900
          ....|....|....|....|....|....|....|....|....|....|....|....|....|....|....|....|....|....|....|....|
XTERV-S   ----------------------------------------------------------------------------------------------------
XLERV-S   CATTTTGCATTTTGGTAAGTATAATTGGCGAAAAGCGGCCAGAGCATTGTTCCTCCCTTTTTTGCTATGTGGATGGGATTTGGGAAGCATACAGGGTTTA

                3910      3920      3930      3940      3950      3960      3970      3980      3990      4000
          ....|....|....|....|....|....|....|....|....|....|....|....|....|....|....|....|....|....|....|....|
XTERV-S   ----------------------------------------------------------------------------------------------------
XLERV-S   TGGTGGCTTGGCTTGGAGAAATTTTGTTTGTTTCAGGAGTGGGTGGTGCGACAACGTGCCAATTCCAGAGGTTTGGTGCATTATCTTGATGATTTCTTGT

                4010      4020      4030      4040      4050      4060      4070      4080      4090      4100
          ....|....|....|....|....|....|....|....|....|....|....|....|....|....|....|....|....|....|....|....|
XTERV-S   ----------------------------------------------------------------------------------------------------
XLERV-S   GCGTGGGCCCGGCGATTTCTGATGAGTGCCTACATGTGCTGGAGACGCTACAAGAGGTGGCTAATGAATTCGGGGTAACGTTAGCTCCGGACAAGACGGT

                4110      4120      4130      4140      4150      4160      4170      4180      4190      4200
          ....|....|....|....|....|....|....|....|....|....|....|....|....|....|....|....|....|....|....|....|
XTERV-S   ----------------------------------------------------------------------------------------------------
XLERV-S   GGGTCCAGTGACCTGCCTTTGTTTTTTGGGGTTGGAAATCGATACAATGGCGGGTGAATGCCGCTTACCACTTGATAAGTTGTGTGATTTAAAGCAGTGG

                4210      4220      4230      4240      4250      4260      4270      4280      4290      4300
          ....|....|....|....|....|....|....|....|....|....|....|....|....|....|....|....|....|....|....|....|
XTERV-S   ----------------------------------------------------------------------------------------------------
XLERV-S   GTGGATACACTGGCACAGCGGTGAAAGGTAAAGGTTAGAGAGGTGCAGTCCCTTATGGGTAGACTAAACTTCGCGTGCCGGGTGATTGTGATGGGCAGGG

                4310      4320      4330      4340      4350      4360      4370      4380      4390      4400
          ....|....|....|....|....|....|....|....|....|....|....|....|....|....|....|....|....|....|....|....|
XTERV-S   ----------------------------------------------------------------------------------------------------
XLERV-S   TATTCTGCCGCAGGCTGGGCAAATTGTTGGCAGGGGCACCTCATCACCATGTCCGGTCGAATACAGAGGTCAGAGACGATTTGTTTTTGGAATCATTCAA

                4410      4420      4430      4440      4450      4460      4470      4480      4490      4500
          ....|....|....|....|....|....|....|....|....|....|....|....|....|....|....|....|....|....|....|....|
XTERV-S   ----------------------------------------------------------------------------------------------------
XLERV-S   TTGAAAATTTATTTTTCCCGGACGGGAAATAACTAGTATGGTGATGCAATTGTTTACGGACGCAGAGAGGAGTCTTGGTTTCGGCGCGTATTTAGAGGGG

                4510      4520      4530      4540      4550      4560      4570      4580      4590      4600
          ....|....|....|....|....|....|....|....|....|....|....|....|....|....|....|....|....|....|....|....|
XTERV-S   ----------------------------------------------------------------------------------------------------
XLERV-S   AGATGGTGCGCGGAGGTATGGTCGGGAGAGTGGGCCGGGTTAGGTCTGACGAGGAACCTTTGTTTTTTAGAGTTGTTTCCCATTGTTATTGCCCTGTTTA

                4610      4620      4630      4640      4650      4660      4670      4680      4690      4700
          ....|....|....|....|....|....|....|....|....|....|....|....|....|....|....|....|....|....|....|....|
XTERV-S   ----------------------------------------------------------------------------------------------------
XLERV-S   TTTGGGGGGACAGCTTATGCAACAGGAGAGTGATATTTGTATCGGACAATTGGGGGTTGTACAGGCAATAAACAGGCAGTCGGCTTCCTCGGCTGAGGTG

                4710      4720      4730      4740      4750      4760      4770      4780      4790      4800
          ....|....|....|....|....|....|....|....|....|....|....|....|....|....|....|....|....|....|....|....|
XTERV-S   ----------------------------------------------------------------------------------------------------
XLERV-S   GTGCGACTTTTGCGAGTTCTGGTTTTGCGCTGCTTGCAGCTTAATGTCAGTTTTCGAGCAGTGCACATTCCTGGGGTTCATAATGAGATTGCTCTTTGTC

                4810      4820      4830      4840      4850      4860      4870      4880      4890      4900
          ....|....|....|....|....|....|....|....|....|....|....|....|....|....|....|....|....|....|....|....|
XTERV-S   ----------------------------------------------------------------------------------------------------
XLERV-S   TCGTTTCCAGTGGGTTCATTTTCGGAAATTGGCTCCGTTGGCGCGGGAGGAAGGCCACCATTTCCCAGTGTGCCTGTGGAGACTCACACGGACGCACGGA

                4910      4920      4930      4940      4950      4960      4970      4980      4990      5000
          ....|....|....|....|....|....|....|....|....|....|....|....|....|....|....|....|....|....|....|....|
XTERV-S   ----------------------------------------------------------------------------------------------------
XLERV-S   TTGGCGGGTTGGTGAGATCATCGGTGGCCACGACCACGTGGGTGGCATATGGTAAGGTCTGGAAAGAATGGGAGCAGTTGGTTCATTGGGCCTCGGGTAC

                5010      5020      5030      5040      5050      5060      5070      5080      5090      5100
          ....|....|....|....|....|....|....|....|....|....|....|....|....|....|....|....|....|....|....|....|
XTERV-S   ----------------------------------------------------------------------------------------------------
XLERV-S   ACAGCTACTACAGGCCAGGCACGAACTCATGCTGTGGTGGGTATACTGGATGGCGGAGCAAGGTCGATCAGTAGCTCAGATTGAGAAGAGTAAGGCTGCA

                5110      5120      5130      5140      5150      5160      5170      5180      5190      5200
          ....|....|....|....|....|....|....|....|....|....|....|....|....|....|....|....|....|....|....|....|
XTERV-S   ----------------------------------------------------------------------------------------------------
XLERV-S   TTGGCCTTTTGGTTTAAAATACATGGTTGGGAGGATTTGTCGAAGGTTTTGTCATTGGACAGGCGATTAAAGGGTTGAAGAAGGGTCACGTACAGCAGGC

                5210      5220      5230      5240      5250      5260      5270      5280      5290      5300
          ....|....|....|....|....|....|....|....|....|....|....|....|....|....|....|....|....|....|....|....|
XTERV-S   ----------------------------------------------------------------------------------------------------
XLERV-S   CCATGTCTTTTAGAAAGTTTGGCTAGCGTTTTGATTAAAATTTGCTGTTCTTAATACGAATCTTTTTTATTCCGGGTGGCTTTCGTTTTGGCGTTTTTTG

                5310      5320      5330      5340      5350      5360      5370      5380      5390      5400
          ....|....|....|....|....|....|....|....|....|....|....|....|....|....|....|....|....|....|....|....|
XTERV-S   ----------------------------------------------------------------------------------------------------
XLERV-S   GCACGTTCAGGATCGGGGAACTGGTCAGCCGCAGTAAACGGGCGTACGGGGGGCTGCAGAGGTGGGACGTAGAATTGTGTCAGGAACACGTGGGGATATG

                5410      5420      5430      5440      5450      5460      5470      5480      5490      5500
          ....|....|....|....|....|....|....|....|....|....|....|....|....|....|....|....|....|....|....|....|
XTERV-S   ----------------------------------------------------------------------------------------------------
XLERV-S   GCTCCGCCGATCAAAGACCAATCAGTTAGGTAGAGGACGCCAGGTGGTGCTATATGCCTTGAAAGGGGGGCCAATTTGCCCTTTCAGTAATGTGCAACAA

                5510      5520      5530      5540      5550      5560      5570      5580      5590      5600
          ....|....|....|....|....|....|....|....|....|....|....|....|....|....|....|....|....|....|....|....|
XTERV-S   ----------------------------------------------------------------------------------------------------
XLERV-S   TTTTTGGTCGCTCTGCCTGGGCTGGACGGCCCCTTCTTAATTCATGAAGACGGCACGCCGCTGACCAGGTTTCAGTTTGTGAGGGTTTTTAGGATGGGGT

                5610      5620      5630      5640      5650      5660      5670      5680      5690      5700
          ....|....|....|....|....|....|....|....|....|....|....|....|....|....|....|....|....|....|....|....|
XTERV-S   ----------------------------------------------------------------------------------------------------
XLERV-S   TAGAGGCATTGGGCATGGTTCCCATGGAGTATGGATCGCATTCTCTTCGTATTGGGGCGGCTACTGAGGCGGCGCGGCTCGGTCTTGGTGAGAGGGTGGT
```

```
                  5710      5720      5730      5740      5750      5760      5770      5780      5790      5800
            ....|....|....|....|....|....|....|....|....|....|....|....|....|....|....|....|....|....|....|....|
XTERV-S     ----------------------------------------------------------------------------------------------------
XLERV-S     AAAGAGGATTGGGCGATGGGAATCGAGGAGATTTCGGAGTTATGTACGTCCAACTTTGTTACAATAATGTCTTACTAGTGGAGGGTTGTTATGTTTTGGG

                  5810      5820      5830      5840      5850      5860      5870      5880      5890      5900
            ....|....|....|....|....|....|....|....|....|....|....|....|....|....|....|....|....|....|....|
XTERV-S     ----------------------------------------------------------------------------------------------------
XLERV-S     AAGGGTTATGGAGTTAATTCACGGTTGGTTGTGGTGATTATTTGTTTAAGGTGATTGTCGGCGAGTGTGGATCGTTGGACATTCCTTTGTGACTTGGGCG

                  5910      5920      5930      5940      5950      5960      5970      5980      5990      6000
            ....|....|....|....|....|....|....|....|....|....|....|....|....|....|....|....|....|....|....|
XTERV-S     ----------------------------------------------------------------------------------------------------
XLERV-S     GAAAGGAGAGCTGCAGTTCGGAGGAGAGGTCAGCAGCTGGGCTTCCCGGACCGGCAGGTGGTGGTAAAATGGTTTGGTTTCCCAGGTCTACAATGGCCTG

                  6010      6020      6030      6040      6050      6060      6070      6080      6090      6100
            ....|....|....|....|....|....|....|....|....|....|....|....|....|....|....|....|....|....|....|
XTERV-S     ----------------------------------------------------------------------------------------------------
XLERV-S     GCTTATTTGACAGACTTACGGAAAAAGCTACGAAAGAAGAGCATCCCCACATTCTGGTTTTGCACGCGGGTGTAACGATATGGGAATTATGAGTCAAAAG

                  6110      6120      6130      6140      6150      6160      6170      6180      6190      6200
            ....|....|....|....|....|....|....|....|....|....|....|....|....|....|....|....|....|....|....|
XTERV-S     ----------------------------------------------------------------------------------------------------
XLERV-S     GATTTGGTAAGGTTGATTAAGTTTGATATTGATAAAATACGTTCATTGTTCACAGGAGTGGTGGTGGGAATGGTCCGAAATGGTGCCTCGGCTGGTATGG

                  6210      6220      6230      6240      6250      6260      6270      6280      6290      6300
            ....|....|....|....|....|....|....|....|....|....|....|....|....|....|....|....|....|....|....|
XTERV-S     ----------------------------------------------------------------------------------------------------
XLERV-S     CAGTGGGCAAGGGATTATTCTGCAATGGAACGGAGCAGGGTGAAGCTGAACAAGTTGCTTAGCACCTTCATTAGAAGGTCCGGTGGGGTTGTGGTAAGGC

                  6310      6320      6330      6340      6350      6360      6370      6380      6390      6400
            ....|....|....|....|....|....|....|....|....|....|....|....|....|....|....|....|....|....|....|
XTERV-S     ----------------------------------------------------------------------------------------------------
XLERV-S     ACAAGGAGTTGGAGAATGCTATGCCGAGTTATTACAGGAGGGATGGTGTTGATCTGTCAGATGTGGGCATGGATATATTTACTCTTAATTTGAAGGTGGG

                  6410      6420      6430      6440      6450      6460      6470      6480      6490      6500
            ....|....|....|....|....|....|....|....|....|....|....|....|....|....|....|....|....|....|....|
XTERV-S     ----------------------------------------------------------------------------------------------------
XLERV-S     GGTGGAAAGAGCCTTGCGGGTGCATGGTGGGGTGTCACAGCCAGCAGAAGGGGGTTACGCCGGCAGCTCAGTGGCGGTGTGGGGCTGCCCTAATGGGTAG

                  6510      6520      6530      6540      6550      6560      6570      6580      6590      6600
            ....|....|....|....|....|....|....|....|....|....|....|....|....|....|....|....|....|....|....|
XTERV-S     ----------------------------------------------------------------------------------------------------
XLERV-S     CCAAAAAGGGGGCTGGAGTGTTAAGGTGAGATTGGCCTGGGGCTAGGCTTAGGGGCAACGGGGCACACTAAGTGAGAAAGTAGGTGACGGAGGCTTGCTT

                  6610      6620      6630      6640      6650      6660      6670      6680      6690      6700
            ....|....|....|....|....|....|....|....|....|....|....|....|....|....|....|....|....|....|....|
XTERV-S     ----------------------------------------------------------------------------------------------------
XLERV-S     AGGCTGGAGAACGGGTTGGTTAGGAAGAGTAATATGAATTCATTATAAGCTACTGTTGAATGTTATGCAGCCTGAAGCGGCTATTGTTTTACAATGTCAC

                  6710      6720      6730      6740      6750      6760      6770      6780      6790      6800
            ....|....|....|....|....|....|....|....|....|....|....|....|....|....|....|....|....|....|....|
XTERV-S     ----------------------------------------------------------------------------------------------------
XLERV-S     ATCTTATTCATGTTATGCATTTGTTATAATAATAATGTGGGTATGTGGGTGGATCATTTTTTCTTAAGCTTGGTGAGTTTAATAAAAACAAGCTGTGGCC

                  6810      6820      6830      6840      6850      6860      6870      6880      6890      6900
            ....|....|....|....|....|....|....|....|....|....|....|....|....|....|....|....|....|....|....|
XTERV-S     --------------------------------------------------------------------------------ACAATTCAAGCTTTGCTAGA
XLERV-S     TTTTTCATCCAAAATGTTGTATTGTATTTTATTAAACGAAAGGGTCAATCAACGATATTGTGTCATACGTAAGTCATGAA.....A.....C........

                  6910      6920      6930      6940      6950      6960      6970      6980      6990      7000
            ....|....|....|....|....|....|....|....|....|....|....|....|....|....|....|....|....|....|....|
XTERV-S     GGTTGGAGTATTTCGGCCAGCAGTAAGTCCATTTAATGCTCCAGTATTTCCTGTAAAGAAAAAGGATGGAAGCTGGAGAATGACTGTGGACTATCGTGGG
XLERV-S     A.C..........TA-.........................T...T..............G.......................G.....T.....G...

                  7010      7020      7030      7040      7050      7060      7070      7080      7090      7100
            ....|....|....|....|....|....|....|....|....|....|....|....|....|....|....|....|....|....|....|
XTERV-S     TTAAACAAAGCAGCTCCACCTCTTGCAGCTGCTGTTCCAGATATAGTATCTATTGTGGAGGACATTGCACAAACTGCTGGAGATTGGCATGCAGTATTGG
XLERV-S     ..............C...........T.................G..T........C.........A.C............A................A.

                  7110      7120      7130      7140      7150      7160      7170      7180      7190      7200
            ....|....|....|....|....|....|....|....|....|....|....|....|....|....|....|....|....|....|....|
XTERV-S     ATTTAGCAAATGCTTTCTTTTCCATTCCTATTGCTGAGGAATCTCAAGATCAGTTTGCATTCACCTGGGAGGGAAAACAATACACATTAACTGTAGTACC
XLERV-S     .C..G.A.........A................A......G.......GC....................A..C........T...C.......G.....

                  7210      7220      7230      7240      7250      7260      7270      7280      7290      7300
            ....|....|....|....|....|....|....|....|....|....|....|....|....|....|....|....|....|....|....|
XTERV-S     ACAGGGGTACATGCATTCTCCCACATTATGTCATGGATTGGTGGCTAGGGATCTGGCCATGCTGCCTAACATGGACTGTAAGTTTTATCACTACATTGAT
XLERV-S     ............A............A.CA..T.....C....A..C.AT...................CAC.....A.....C.G...............

                  7310      7320      7330      7340      7350      7360      7370      7380      7390      7400
            ....|....|....|....|....|....|....|....|....|....|....|....|....|....|....|....|....|....|....|
XTERV-S     GATGTTATGATCTCAGGCAGCTCAGAGGAACAAGTGAGAAA-AGACTTACAAACAGTGGTGACATATATGCAGAAAAGAGGGTGGGCCATAAACCCAGAA
XLERV-S     ...A.................................A...TG..T............T......G.G.............A.....T.....T.T...C

                  7410      7420      7430      7440      7450      7460      7470      7480      7490      7500
            ....|....|....|....|....|....|....|....|....|....|....|....|....|....|....|....|....|....|....|
XTERV-S     AAAATCCAAGGACCAGCAACTAGTGTCAGATTTCTTGGAATGATCTGGGCTGGACCAGTCAAATCTATCCCACAGCCTGTGTTGGATTCAATTGCTGCCC
XLERV-S     .................T.A...............A.......................G..GA......................C........G..A.

                  7510      7520      7530      7540      7550      7560      7570      7580      7590      7600
            ....|....|....|....|....|....|....|....|....|....|....|....|....|....|....|....|....|....|....|
XTERV-S     TTAAACCACCAAAAAATGTGAAAGAAGCTCAGAGTTTGTTGGGTCTGCTTGGATTTTGGAGGCCTTTTATTCCACACCTGGGCCTCATTCTCAGACCTAT
XLERV-S     .C........C........T..G.....C...........AA.C.....G.......................G....................G.....
```

```
                 7610      7620      7630      7640      7650      7660      7670      7680      7690      7700
         ....|....|....|....|....|....|....|....|....|....|....|....|....|....|....|....|....|....|....|....|
XTERV-S  CTACAACATAACTAGGAAAAAGACTGAGTTTACCTGGGGGTCTGAGCAGCAATTGGCACTTGACACAGCCAAAGAAACTGTAAGAACCCATCATTCACTA
XLERV-S  T.....T..T..........G.G....A.....T.....A........A...........C...G...................G..T............

                 7710      7720      7730      7740      7750      7760      7770      7780      7790      7800
         ....|....|....|....|....|....|....|....|....|....|....|....|....|....|....|....|....|....|....|....|
XTERV-S  GGTCCTATACATCCAGATAAGCCATTTTTCTTAGATGTAGCAGTAACAGACCATGGAATGTCTTGGGGACTGTGGCAAAAAGGGCCTAATCCCAGGGATC
XLERV-S  ...T....T..A......C..T....C..TC.........T...G.....T..................T......................A..A....

                 7810      7820      7830      7840      7850      7860      7870      7880      7890      7900
         ....|....|....|....|....|....|....|....|....|....|....|....|....|....|....|....|....|....|....|....|
XTERV-S  GAAAAATACCTTTGGGTTTTTGGTCTAAGCAATTTTCAACTGCTCAGAAAAAATACAGCCCATTAGAGAAGCAATTGTTAGCTGCTTATACTGCCTTACA
XLERV-S  .G.....T...C.......C...........-C..........G........G....A...............G......A........TT...T..G..
                 7910      7920      7930      7940      7950      7960      7970      7980      7990      8000
         ....|....|....|....|....|....|....|....|....|....|....|....|....|....|....|....|....|....|....|....|
XTERV-S  ACATGTTGAGCCAACTACAAAGCAGCAATCTGTGACAGTAAGGACTGATTTACCTATTGCTGGATGGGTCAGACAAGAAGGGCTACAATCCCGCACAGGT
XLERV-S  ............T..C..C..A......C.......T..G.......................G.....G.....G...............T.T...A..

                 8010      8020      8030      8040      8050      8060      8070      8080      8090      8100
         ....|....|....|....|....|....|....|....|....|....|....|....|....|....|....|....|....|....|....|....|
XTERV-S  GTGGCCCAGAAACAGACTTTGCAGAAATGGAAATGGTATCTGAGTGAAAGAGGGGGAATATCTTCCCAAGACCCTAGTCAGCTATCGCAACAAATTGCTG
XLERV-S  .................C..------..A..........T.............................A.T..............A..........T..

                 8110      8120      8130      8140      8150      8160      8170      8180      8190      8200
         ....|....|....|....|....|....|....|....|....|....|....|....|....|....|....|....|....|....|....|....|
XTERV-S  GTTTTGTTAACTTTGAACCTAATGTAAATCAATTGCCCCCACTGCATGCAGAGGAGTCACTATTCAAGGAAGCCCCACCATGGATAGAGTTATCAGCAGA
XLERV-S  .C.......GT......T.A...TC.CC...........-.T....AAT.........T.C...........A...............A...........

                 8210      8220      8230      8240      8250      8260      8270      8280      8290      8300
         ....|....|....|....|....|....|....|....|....|....|....|....|....|....|....|....|....|....|....|....|
XTERV-S  AGATAAACTACGAGCATGGTTTACAGATGGCTCAGCTAAAGTGACTTACAAGGGGCGGATATGGACTGCAGCAGCTTTTCAGCCTAGTTCAGAGACTATA
XLERV-S  ...........A......T...........T.............T..C......CT...................C..C..................G.G

                 8310      8320      8330      8340      8350      8360      8370      8380      8390      8400
         ....|....|....|....|....|....|....|....|....|....|....|....|....|....|....|....|....|....|....|....|
XTERV-S  ATTCATCAGACGGGAGAAGGAGGTTCTAGTCAATATGCTGAACTACAAGCTGTTTATATGGTTGTCCAGGAGACCTCAGGCGACTTGATTATCTATACAG
XLERV-S  ........A..T........................A..........G..G.................A..A........G.G.........----..G.

                 8410      8420      8430      8440      8450      8460      8470      8480      8490      8500
         ....|....|....|....|....|....|....|....|....|....|....|....|....|....|....|....|....|....|....|....|
XTERV-S  ATAGCTGGGCTGTATTTAAAGGTCTCACAACATGGCTTTGTATTTGGAAAAAAGATAACTGGCAAGTAAATGGAAAAGAGTTATGGGGCGGGCCTCAAGT
XLERV-S  .C..T...T.............A......G............CC........-..C.GT.T...G...........G...C...................

                 8510      8520      8530      8540      8550      8560      8570      8580      8590      8600
         ....|....|....|....|....|....|....|....|....|....|....|....|....|....|....|....|....|....|....|....|
XTERV-S  ATGGGATTTCTTATGGTGTCAAGGAAAAAAGAGATGTATACAAGTAGGCCATGTTAATGCTCACACTGGAAATCTGAACAATGAAATAGTAGATGGTCTG
XLERV-S  ......C.AT......AA......C.....T.........A.G.......C.A............A.......G...............A.........A

                 8610      8620      8630      8640      8650      8660      8670      8680      8690      8700
         ....|....|....|....|....|....|....|....|....|....|....|....|....|....|....|....|....|....|....|....|
XTERV-S  GCACAAGTCTCTACTGCACAACAAGAGGAAACTACCTTGGAAGTGTTGGGCAAATGGGCCCACAGTCAAACTGGGCACAAAGGAGTTCAAGGTACCTGGC
XLERV-S  .....G......TA..T............G............T...............................A..T.G....A..........T....

                 8710      8720      8730      8740      8750      8760      8770      8780      8790      8800
         ....|....|....|....|....|....|....|....|....|....|....|....|....|....|....|....|....|....|....|....|
XTERV-S  AGTGGGCACAGCAGAGGGGAATACCTCTAACACAAATCCAAGTGAAGGACATAATCGCTAAATGTCCTGTATGTCAGGAAGCCAAGAAGTGGCCACCTTT
XLERV-S  ................A.................G...T....A........T...A..C........A.............T.....A........CA.

                 8810      8820      8830      8840      8850      8860      8870      8880      8890      8900
         ....|....|....|....|....|....|....|....|....|....|....|....|....|....|....|....|....|....|....|....|
XTERV-S  GACCCCATTGCCTGGAAAAATTCATCGAGGACAAAAGCCAGGACAAGTCTGGCAAGTGGATTATATTGGACCCTTGCCTGGAGGAAGGGGTGGTTTAAAA
XLERV-S  ......T.........G...---------------.......TT.....................................G.....A........G.G.

                 8910      8920      8930      8940      8950      8960      8970      8980      8990      9000
         ....|....|....|....|....|....|....|....|....|....|....|....|....|....|....|....|....|....|....|....|
XTERV-S  TATTGTGCGACTGCTGTGGACACATACAGTGGACTAGTACAAGTATTTCCAACAAAAAGTGCAGATCAAAAAACAACTCTAAGATTGATGCAACTGCTAA
XLERV-S  .T......A...................A....T.G.........................TG........G..T..CT..C.G.........T.AT.G.

                 9010      9020      9030      9040      9050      9060      9070      9080      9090      9100
         ....|....|....|....|....|....|....|....|....|....|....|....|....|....|....|....|....|....|....|....|
XTERV-S  TACAACATTATGGAATGCCTCAAGAAGTACAGTCAGATAATGGCACACATTTTACTGGACAGACCGTAAAGCAATGGGCGGAGGATAATGGTGTATACTG
XLERV-S  .........................................A..........C...A.......T............C.....A.A.........C..A.

                 9110      9120      9130      9140      9150      9160      9170      9180      9190      9200
         ....|....|....|....|....|....|....|....|....|....|....|....|....|....|....|....|....|....|....|....|
XTERV-S  GGTGTTCCACATTCCGTATTATCCTCAAGGGGCAGCGTTGATTGAGAGAATGAATGGTCTTTTAAAGGAACAAATGCGCAAACTTACACCTACTCACACA
XLERV-S  .........T.....C..C..C..C....CA....G...............A......A....T.....T.....TT..............G...G....

                 9210      9220      9230      9240      9250      9260      9270      9280      9290      9300
         ....|....|....|....|....|....|....|....|....|....|....|....|....|....|....|....|....|....|....|....|
XTERV-S  CTACGGGGATGGGATAAGGTGTTACAAGAGGCAGTATATTTATTAAATAATAGATCAGTTGGACATTTCACTCCTATTCAAAGAATGCTGGGAGAAAGTG
XLERV-S  ...A....G.............................C...C.G..............A......AG..........................GG....

                 9310      9320      9330      9340      9350      9360      9370      9380      9390      9400
         ....|....|....|....|....|....|....|....|....|....|....|....|....|....|....|....|....|....|....|....|
XTERV-S  GGGAGAATAGTTCAGATTGGGTAGTAACAGTTACTACAAAAGGTTCAACAGTCCCTTTATCTCAGTCTATTCCTGTATTTATACACAAAGATTTAGTTGT
XLERV-S  ...................A..G..T..T..G.........CAGC.....C.T...AC..T....C.A........GG.....TG......C....C...
                 9410      9420      9430      9440      9450      9460      9470      9480      9490      9500
         ....|....|....|....|....|....|....|....|....|....|....|....|....|....|....|....|....|....|....|....|
XTERV-S  AGGAGGTGAAGAAGAACATACTATGTTACAAATTACATCCATATCAGTACCAACAGGAATTTTAGACATAGATCCAACATGTGACTTGCAATTCATCCCA
XLERV-S  ...................TTC.-----------------.C.............T.....A....T.......T..........C.......T......
```

```
                  9510      9520      9530      9540      9550      9560      9570      9580      9590      9600
          ....|....|....|....|....|....|....|....|....|....|....|....|....|....|....|....|....|....|....|....|
XTERV-S   GATTTTGATTTAACCACTCAGTGTGACTGGGATGTTGACGTTAGAAAAGATGAATTGGGGGAATATTGTGTCACTATTGTTCCTTTTGGTCGGGCACAGT
XLERV-S   .G.......A................T......CCAA.G..G.....................CC....T.T.C.............A.A........
                  9610      9620      9630      9640      9650      9660      9670      9680      9690      9700
          ....|....|....|....|....|....|....|....|....|....|....|....|....|....|....|....|....|....|....|....|
XTERV-S   TTAAGAAAGGACAAAAAATAGGACAAATAGTGATTTTACCAAAACGTTTGCAAGTGAAGGGAAATATTTATTCCACACAGTTAGGAACAAAGGTATGGAT
XLERV-S   ....AG..A.G.-----------..............A.....T..-------....A......................G........A......G.

                  9710      9720      9730      9740      9750      9760      9770      9780      9790      9800
          ....|....|....|....|....|....|....|....|....|....|....|....|....|....|....|....|....|....|....|....|
XTERV-S   AGCTCCAAGTGTAACAGACAGGGGTAGAAAAACTGGTAGAAAAGGAGAAATTGTGGCCTGGGGTCCTGGGTCAACAGCATTAGTTTTAATAGATGCTGAA
XLERV-S   CT.C....A...............A......T.A.....G..G........CA..........A.........................GA..T.....

                  9810      9820      9830      9840      9850      9860      9870      9880      9890      9900
          ....|....|....|....|....|....|....|....|....|....|....|....|....|....|....|....|....|....|....|....|
XTERV-S   GATAAGCCTGTTTATGTTCCTCTTCACAGATTACTACCACTACCATGAGGAGTTTGCCTTGTCTACTGATGCTCGCGATATGTCTGATGAAAGTGACTGC
XLERV-S   .......................C..............TT.G..........CC.C.A..A.T..........T..A..T..C..AT...--...C....

                  9910      9920      9930      9940      9950      9960      9970      9980      9990     10000
          ....|....|....|....|....|....|....|....|....|....|....|....|....|....|....|....|....|....|....|....|
XTERV-S   GGAAAGGAATTTTTATCATGAAACGCTGAAAGCTACTGCTGCAGTATTTAATGTAACAAACTGTTGGATATGTGGCAAAATCCCACATGCGACTGAAGAA
XLERV-S   ......A..C..C...........A....GG..A..CA......A.......A....T........T..T.....A..............C.....G...

                 10010     10020     10030     10040     10050     10060     10070     10080     10090     10100
          ....|....|....|....|....|....|....|....|....|....|....|....|....|....|....|....|....|....|....|....|
XTERV-S   GGAATTCCACTTTATGGACTGCCATTTAATATGAGTTGGATAAACAGAACCGACCCTGAGTGGAACTTTGTTTTCAATATGACTACAAAACAATGTGCTA
XLERV-S   ..................T...........G........G.T........T........A......ACA................G..........CAA.-

                 10110     10120     10130     10140     10150     10160     10170     10180     10190     10200
          ....|....|....|....|....|....|....|....|....|....|....|....|....|....|....|....|....|....|....|....|
XTERV-S   TTGCTAGATATGCAGGTACAGAAAAAGAGCAACAGTTGAAGTTAACCCGGAAATCAACAGGGATTTTGTGTGTCCAGAAAAATCAAACCACTGAGACTGT
XLERV-S   --------..AA...T.T.TA...................C...A.G.A.G...................................A.G...T.....

                 10210     10220     10230     10240     10250     10260     10270     10280     10290     10300
          ....|....|....|....|....|....|....|....|....|....|....|....|....|....|....|....|....|....|....|....|
XTERV-S   GTGGTTAGGAAAAAGTCAATGTGACTATGTTGTCAACACAGTTAA-------------------------------------------------------
XLERV-S   ............T..C........T......T.A...........AGGAACAGTAACATCAAAAAATAAAAGTGTTTTAAAGTAATGAAAATATAATGTA

                 10310     10320     10330     10340     10350     10360     10370     10380     10390     10400
          ....|....|....|....|....|....|....|....|....|....|....|....|....|....|....|....|....|....|....|....|
XTERV-S   ----------------------------------------------------------------------------------------------------
XLERV-S   GCGTTGCCCTGCACTTGTAAAACTGCTGTGTTTACTTAAGAAACACTACTATTGTTTATATAAATAAGCTGCTGTGTAGCAATGGGGGCAGCCATTCAAA

                 10410     10420     10430     10440     10450     10460     10470     10480     10490     10500
          ....|....|....|....|....|....|....|....|....|....|....|....|....|....|....|....|....|....|....|....|
XTERV-S   ----------------------------------------------------------------------------------------------------
XLERV-S   GGAGAAAAGGCTCAGGTTACACAGCAGCTTGTTTATATGAACTATAGTAGTGTTTCTGAAGCAAACTGATCAGTTTTACCAGAGCAGGGCAAGACTACAT

                 10510     10520     10530     10540     10550     10560     10570     10580     10590     10600
          ....|....|....|....|....|....|....|....|....|....|....|....|....|....|....|....|....|....|....|....|
XTERV-S   ------------------------------------------------------CAACACACATGTAATGGTGACAGGAAAAGGGGAAATGTTTTATATG
XLERV-S   GATATTTTCATTACTTTAAAACACTTACATTTTTTGGTGTTACTGTTCCTTTAA..................A.A.........................A

                 10610     10620     10630     10640     10650     10660     10670     10680     10690     10700
          ....|....|....|....|....|....|....|....|....|....|....|....|....|....|....|....|....|....|....|....|
XTERV-S   TTTTATGTT--------AAGCAATGTTCATTTGATT--TTAGAGTTACTACAAATGATACGCATATTTGCGAAAGATCAA---GTACAAGC---------
XLERV-S   ......A.ATTCTGCCA....G.CCCC.CAGC...CAACC...CC.CT.TGC..A.T.GATGG.GACA.A....AC.TT.TTT.CTTT..TAGCCACAGA

                 10710     10720     10730     10740     10750     10760     10770     10780     10790     10800
          ....|....|....|....|....|....|....|....|....|....|....|....|....|....|....|....|....|....|....|....|
XTERV-S   ----------------------------------------------------------------------------------------------------
XLERV-S   ATGACACTGCCCAGAATTAAGATGGCCATAGATTTTTAAAAGATCCAATCCTCATCGTGAGACCAAGATTTTCTCAGAACGATTGTACGATAGTACGAAT

                 10810     10820     10830     10840     10850     10860     10870     10880     10890     10900
          ....|....|....|....|....|....|....|....|....|....|....|....|....|....|....|....|....|....|....|....|
XTERV-S   ----------------------------------------------------------------------------------------------------
XLERV-S   TGACCATCAACTAAAAAGACAAATGTGCCAGGAAAACAAAGGGGAGCTGCCTGCTTGGCCCTGCAAACATAGATAGATTGCACTGGGACCAACAAAGATT

                 10910     10920     10930     10940     10950     10960     10970     10980     10990     11000
          ....|....|....|....|....|....|....|....|....|....|....|....|....|....|....|....|....|....|....|....|
XTERV-S   ----------------------------------------------------------------------------------------------------
XLERV-S   TTTTGACCTGGCCGATCAATTTCCTGACAGATGTCGGCCGAAAAATTGTAAGATGTACGATTGTTCGAATGCCACTAACCGCACGATAATTTCGAAGGAT

                 11010     11020     11030     11040     11050     11060     11070     11080     11090     11100
          ....|....|....|....|....|....|....|....|....|....|....|....|....|....|....|....|....|....|....|....|
XTERV-S   ----------------------------------------------------------------------------------------------------
XLERV-S   TGGTCGGACTTCCCTAAAATCGGCCGTTCAGCATGAAGAATCGTCGCGTCTATGGGGAGCTTTAGACAACTTGTTATCTACAAAAACCCCTAGATCCTTA

                 11110     11120     11130     11140     11150     11160     11170     11180     11190     11200
          ....|....|....|....|....|....|....|....|....|....|....|....|....|....|....|....|....|....|....|....|
XTERV-S   ----------------------------------------------------------------------------------------------------
XLERV-S   TCCTTTAAGGAAAATCCCAACACACTGCTATTTGGTGTATAACTTGCATTTATATTATTTTTGCAAAATGCATAACCTTGCATTTATCAACATTGAACCT

                 11210     11220     11230     11240     11250     11260     11270     11280     11290     11300
          ....|....|....|....|....|....|....|....|....|....|....|....|....|....|....|....|....|....|....|....|
XTERV-S   ----------------------------------------------------------------------------------------------------
XLERV-S   CATTTTCCAGTTTGCTACCCAGTTCTCCAACTTAGACAAATCACTCTGCAAATCCTGCATGGAACCTATAGTTCTGCACAATTTAGTATCATCTGCAAAA

                 11310     11320     11330     11340     11350     11360     11370     11380     11390     11400
          ....|....|....|....|....|....|....|....|....|....|....|....|....|....|....|....|....|....|....|....|
XTERV-S   ----------------------------------------------------------------------------------------------------
XLERV-S   ATAGAAACAGTACTTTCAATGCCCACCTCCAGGTCATTAATAAAGGCACTAACAACACTGGTCCAATTAGAAAATGTTCCATTTACCACCACTCTTTGTA
```

```
               11410     11420     11430     11440     11450     11460     11470     11480     11490     11500
          ....|....|....|....|....|....|....|....|....|....|....|....|....|....|....|....|....|....|....|....|
XTERV-S   ----------------------------------------------------------------------------------------------------
XLERV-S   GTCTATCTTTTAGCCAGTTCTCTATACAGGTAACATTTCTTAATTTAACCAGTAACCTTTTGTGTGACACTGTATCAAATGCTTTAGCAAAGTCTAAGTA
               11510     11520     11530     11540     11550     11560     11570     11580     11590     11600
          ....|....|....|....|....|....|....|....|....|....|....|....|....|....|....|....|....|....|....|
XTERV-S   ----------------------------------------------------------------------------------------------------
XLERV-S   AATCACATCCACTGCCATCCCAGAATCGAGGTCTCTACTTACCTTCTCATAAAAATAAATTAAGTTTAAATAAAATCAAAGCCTTAACTCACCTATTTAA

               11610     11620     11630     11640     11650     11660     11670     11680     11690     11700
          ....|....|....|....|....|....|....|....|....|....|....|....|....|....|....|....|....|....|....|....|
XTERV-S   ----------------------------------------------------------------------------------------------------
XLERV-S   TCTTTCGCTCTCAACTGGACTGTTTCCTTCTCAACTAAAACATGCTCTTGTCACCCCCATTCTGAAAAAACCCTCTCTTGATCCCTCCAATCTTGACAAC
               11710     11720     11730     11740     11750     11760     11770     11780     11790     11800
          ....|....|....|....|....|....|....|....|....|....|....|....|....|....|....|....|....|....|....|....|
XTERV-S   ----------------------------------------------------------------------------------------------------
XLERV-S   CTCTGACCTATCTCTCTGCTACCTTTCATCTCTAAACTACTTGAGCGCCTAGTCTACAACCGACTAACCTCATTCCTCTCTGACAATAACCTGCTGGACC

               11810     11820     11830     11840     11850     11860     11870     11880     11890     11900
          ....|....|....|....|....|....|....|....|....|....|....|....|....|....|....|....|....|....|....|....|
XTERV-S   ----------------------------------------------------------------------------------------------------
XLERV-S   CCCTACAATCTGGTTTTAAGCCACAACACTCCACGGAAACTGCCCTGACTCGATTAACTAATGACCTTTTAACCGCTAAAGCCAACAATCATTTCTCACT

               11910     11920     11930     11940     11950     11960     11970     11980     11990     12000
          ....|....|....|....|....|....|....|....|....|....|....|....|....|....|....|....|....|....|....|....|
XTERV-S   ----------------------------------------------------------------------------------------------------
XLERV-S   CCTAATACTGCTTGATCTCTCAGCCGCATTTGACACTATAGATCACCTTCTCCTCCTCCAGTCCCTCCATTTGCTTGGCCTTCGTGACACAGCCCTGTGG

               12010     12020     12030     12040     12050     12060     12070     12080     12090     12100
          ....|....|....|....|....|....|....|....|....|....|....|....|....|....|....|....|....|....|....|....|
XTERV-S   ----------------------------------------------------------------------------------------------------
XLERV-S   GTCGAGGCAGGCAGAGAATAAACGTTATCAGGCAGGCAAGGGTCAACCAGGAAGTCAATCAGAAGAGGTAGTCTGTAATCAGGCAAGGGTCAGAATCCAG

               12110     12120     12130     12140     12150     12160     12170     12180     12190     12200
          ....|....|....|....|....|....|....|....|....|....|....|....|....|....|....|....|....|....|....|....|
XTERV-S   ----------------------------------------------------------------------------------------------------
XLERV-S   AAGTCAGAATAGTCAAAATAGCCAGGCAGGGGTCAAATCAAACAGGTTCAGAATATAGCAACGAATAGCACAAGGCTCAGAAGCACCAGGAAACAATATC

               12210     12220     12230     12240     12250     12260     12270     12280     12290     12300
          ....|....|....|....|....|....|....|....|....|....|....|....|....|....|....|....|....|....|....|....|
XTERV-S   ----------------------------------------------------------------------------------------------------
XLERV-S   CTATCACGGGCAATCCATAAGGTAAAACTGTACCTTTAAATACATTTGAAATTCGCACCTTTGCGCGCTGACGTCATACGTCAGCGAGCCTGCGCCTTTA

               12310     12320     12330     12340     12350     12360     12370     12380     12390     12400
          ....|....|....|....|....|....|....|....|....|....|....|....|....|....|....|....|....|....|....|....|
XTERV-S   ----------------------------------------------------------------------------------------------------
XLERV-S   AGACGCAACCAGACGCGGCACGCGCCGAAGAACCCTGCGGGCGTCCCCGCTTGGAGTTTGGCGGGCGTCCCCGCCGACCCCCAACACCCCTACTGGGGTA

               12410     12420     12430     12440     12450     12460     12470     12480     12490     12500
          ....|....|....|....|....|....|....|....|....|....|....|....|....|....|....|....|....|....|....|....|
XTERV-S   ----------------------------------------------------------------------------------------------------
XLERV-S   AGTTATTACAATGTTGCAGAGATGCCCTTATACTTTCAAATTTTGCACGTCTGAAATTTAGTGTTTTAGTTACTCCCTTATAGAATTGCTTCTGCAACAG

               12510     12520     12530     12540     12550     12560     12570     12580     12590     12600
          ....|....|....|....|....|....|....|....|....|....|....|....|....|....|....|....|....|....|....|....|
XTERV-S   -------------------------------CTCTCTGGTATGCTGTAAAATCTTACCATCTAATAGTACCAGGGTAGGAATATACTGGCAATATCAGTT
XLERV-S   AATCTCAAAGGAGACCATGTTATGATCACTAT........C.T.................T..............C......G.................

               12610     12620     12630     12640     12650     12660     12670     12680     12690     12700
          ....|....|....|....|....|....|....|....|....|....|....|....|....|....|....|....|....|....|....|....|
XTERV-S   ATATCTAGCAACTTGTTCCCCATTGCCCAATCAGTTGTACTTTATTTGTGGGAATAATGTTTATAAGTGGCTACCATATAATTGGGCAGGAAATTGTTAT
XLERV-S   .....A.T....C...G.T...C.....G.C........T...........A......ACC.........T.......A..C...T......G...C...

               12710     12720     12730     12740     12750     12760     12770     12780     12790     12800
          ....|....|....|....|....|....|....|....|....|....|....|....|....|....|....|....|....|....|....|....|
XTERV-S   ATAGGAAATATAGCACCAAAATTTAGAGTATTATTTGAGAACCCAAAAGGGCATGTGAGAAATTGGAAA-CGGCATTTTGCCTCTTCTCATGTTAATGAA
XLERV-S   .........G.......T...A.....A.............T................TG.........A.TTT.......T........G...G.....

               12810     12820     12830     12840     12850     12860     12870     12880     12890     12900
          ....|....|....|....|....|....|....|....|....|....|....|....|....|....|....|....|....|....|....|....|
XTERV-S   TTACCCGAAGAG-TGGGATACTTCAGAAGAAAACAGATTTGTTATGATTTTAATACCCCATTATGGAGTAG------CAAAAGCTGTGCAAATGATGAGA
XLERV-S   ..T.GT....G.G......G....C....G.G.......A.CA..T............T..........T.CAACTG...G.T....A..........AG

               12910     12920     12930     12940     12950     12960     12970     12980     12990     13000
          ....|....|....|....|....|....|....|....|....|....|....|....|....|....|....|....|....|....|....|....|
XTERV-S   AGAATGTCTAGAATAATTGAACATGCTTTAAACAACACATTAGATGGCTTGACTAGTCTTACCGAGGAAGTCAGACAAATGAGGTTAGTGGTTCTGCAAA
XLERV-S   ........C..........................T....A....C..C.............T.....C......A.....C.AC...............

               13010     13020     13030     13040     13050     13060     13070     13080     13090     13100
          ....|....|....|....|....|....|....|....|....|....|....|....|....|....|....|....|....|....|....|....|
XTERV-S   ATAGAGCATCGCTAGATTATATCCTAGCTTCAAAGGGAGGTGTATGTGCTCTGATTGGAGATGAATGTTGCACATATATCTCAGATAAAAGTCTGGAAGT
XLERV-S   .C............---.................................T.A..........................T.......G......A...A.

               13110     13120     13130     13140     13150     13160     13170     13180     13190     13200
          ....|....|....|....|....|....|....|....|....|....|....|....|....|....|....|....|....|....|....|....|
XTERV-S   AGAGGATGAC------ATCTCTGAAGCTCGAAAGTCAGTAGCCAAATTACATGCCCAAAATACTGGTAGTTGGTTATTTAGTTGGTTGGGGCAATGGGGA
XLERV-S   ..........TAGAGG...A......TG..T..........................G...G.....---.T...G.................G......

               13210     13220     13230     13240     13250     13260     13270     13280     13290     13300
          ....|....|....|....|....|....|....|....|....|....|....|....|....|....|....|....|....|....|....|....|
XTERV-S   CAACAATTGTTTATGTATATAACATCATTTATTATATTCATTCTAGGAATTTTTCTTGTGATAAAAATAATCAGTGTCTGTATCAGTAAGTATACTACAT
XLERV-S   ..G..---........TC...GTG.A......A...G...A.T..A...........T.T........G..T....CA.............C........
```

```
                  13310     13320     13330     13340     13350     13360     13370     13380     13390     13400
             ....|....|....|....|....|....|....|....|....|....|....|....|....|....|....|....|....|....|....|....|
XTERV-S      CTACAATAGAATCATTACCTATACGCAAAACTGGAATAATATATTAAATATTTCTCAATCAGTAT-TTTTGTAAATCAAAGGGTGGAATGTAGTGCAGTA
XLERV-S      ..GT......GT.....T...TT.........T..................ACACA.........A.............G........ ....A.A...GG

                  13410     13420     13430     13440     13450     13460     13470     13480     13490     13500
             ....|....|....|....|....|....|....|....|....|....|....|....|....|....|....|....|....|....|....|....|
XTERV-S      GGTTTCTTTTACTATGATTTGTATGATTTACAATTTATACATGC----TTTAATATGT-GTATGTATATATATATGTATATGGTTTAGTTAGTAAGTTTA
XLERV-S      .T.G.A.....------------................A...TCTGT........A.T.A..A..A......TA..TC....A.....C....C.G.A.

                  13510     13520     13530     13540     13550     13560     13570     13580     13590     13600
             ....|....|....|....|....|....|....|....|....|....|....|....|....|....|....|....|....|....|....|....|
XTERV-S      ATCAAGCAATTCTAGAGATAAGGGGAGTCCAGTGTCCTTGTACATATATTGTTGGGTAACAATACCTGCGTTTATTATGTAACAATAAACAATGACTATA
XLERV-S      ..AG.AACC.A..GAG....................T.....TG.GCC..TC.......A....GG.A---..G...CCC.C...------------...

                  13610     13620     13630     13640     13650     13660     13670     13680     13690     13700
             ....|....|....|....|....|....|....|....|....|....|....|....|....|....|....|....|....|....|....|....|
XTERV-S      GACTGTTTACCCAGCAAATACAACAAGCGGACGGCAGGCAGACAATGCCCAATGTATTTAAATAAGAACTTGAGGTATAAAGTTTGGTATCTGATCAGAC
XLERV-S      .--------...TAAT.....--..G..A...AAT..C.TC.TC.-.......T.....G.T.....C..G.CT.A.-..G.CA.AAAG.TC---.TA.A

                  13710     13720     13730     13740     13750     13760     13770     13780     13790     13800
             ....|....|....|....|....|....|....|....|....|....|....|....|....|....|....|....|....|....|....|....|
XTERV-S      ACAATGGCTGACCATTTG---------TTAAACAATATTGTTCAAGTCAATAGTTGGGCTTGGGGGTTTTTCCTAAGAACTTTTGGCATAAAAGAACGGC
XLERV-S      ..........GT......CTGTTTTTT.........T...CCT.T....GC.......G..........AA.--....CA...G.C........C..A..

                  13810     13820     13830     13840     13850     13860     13870     13880     13890     13900
             ....|....|....|....|....|....|....|....|....|....|....|....|....|....|....|....|....|....|....|....|
XTERV-S      CTCTGCCTTGGGTCAGGAGCTTCGCCTAGGACTCCTGAACGAGTGCCAGGATATTGGATCATCGCATGGTTATCGGGAACCTGAAGGTTTTGCACC-AAA
XLERV-S      ..T......A...A.........A...T...........A.......G...AC..........A.T..A...............G.T.CA..T...C...

                  13910     13920     13930     13940     13950     13960     13970     13980     13990     14000
             ....|....|....|....|....|....|....|....|....|....|....|....|....|....|....|....|....|....|....|....|
XTERV-S      GGTTGAGGGGCTCCCCGAGTTTGCTG---GATTAATGCTGAACTGGCTATCTTTGTAACCACAAAACCGTACGAAGCTAAGTAAATGTTT-TAAATTCTA
XLERV-S      ..C..C..A.........T.G.....ATT....G..A.............TCC.....TT..C.--T.A.....................C....A....

                  14010     14020     14030     14040     14050     14060     14070     14080     14090     14100
             ....|....|....|....|....|....|....|....|....|....|....|....|....|....|....|....|....|....|....|....|
XTERV-S      TTACTATGTGTGTGTGTAAGTTATTGC-TCATTTAATAGTTTTTCATCAGAAGGTTTAATCATTGATCCTGTTAATATAAATAATATTGATAAAGGTTAA
XLERV-S      CCTTC.CT.........GT..A.GG.TA.TGC.C.CA..CAG..T....A..........T...........

                  14110
             ....|....|...
XTERV-S      CCCCTTTATTACA
XLERV-S
```
