# Supplementary material for: A novel class III endogenous retrovirus with a class I envelope gene in African frogs with an intact genome and developmentally regulated transcripts in Xenopus tropicalis
Source: Retrovirology. 2021 Jul 14;18:20. doi: 10.1186/s12977-021-00564-2 (PMC8278194; doi:10.1186/s12977-021-00564-2)
Supplement: Supplementary file 5 — Additional file 5: Figure S5. XtERV-S like sequences in the African bull frog (P. adspersus) genome. [file 12977_2021_564_MOESM5_ESM.pdf]

>CM016419.1:110619505-110628308
CAGGTTGTTGGGTTAGCACAATTTATTTAATATCAATTATGTCTCACCAACAATAATGCTTTACCAGTATATA
CTGGTATATGTATACAATTTATAAGGTCATCCTAAAGGAATGCAGCATATCCTGTAAGGTTTAAGGATGATTA
TAGGTCAGTACTGGTCAAACCCGGCCATTGTCTTAGTTCCTTCCAGAGTAGTTAAAAAACACAGTGCCATACC
TAATGAGGGAGTCTTGACCAGAGCTTGCTGGATGCAACATGTTTCTTTGATCATCTGCCAAGTATTGATTTCC
CGCAGAATCTTTGGGTGAATTCAGTTTTCTTCCGCAGCTAAGTATCTTTTGTTACCGCTTAATAATTGTTATT
ACAGTATTGCATTGTTTTGTCTATCTNNNNNNNNNNNNNNNNNNNNNNNNNNNNNNNNNNNNNNNNNNNNNNN
NNNNNNNNNNNNNNNNNNNNNNNNNNNNNNNNNNNNNNNNNNNNNNNNNNNNNNNNNNNNNNNNNNNNNNNNN
NNNNNNNNNNNNNNNNNNNNNNNNNNNNNNNNNNNNNNNNNNNNNNNNNNNNNNNNNNNNNNNNNNNNNNNNN
NNNNNNNNNNNNNNNNNNNNNNNNNNNNNNNNNNNNNNNNNNNNNNNNNNNNNNNNNNNNNNNNNNNNNNNNN
NNNNNNNNNNNNNNNNNNNNNNNNNNNNNNNNNNNNNNNNNNNNNNNNNNNNNNNNNNNNNNNNNNNNNNNNN
NNNNNNNNNNNNNNNNNNNNNNNNNNNNNNNNNNNNNNNNNNNNNNNNNNNNNNNNNNNNNNNNNNNNNNNNN
NNNNNNNNNNNNNNNNNNNNNNNNNNNNNNNNNNNNNNNNNNNNNNNNNNNNNNNNNNNNNNNNNNNNNNNNN
NNNNNNNNNNNNNNNNNNNNAACTGCAGGTTGTTACAGAGCAAATATCTCTATTGAGAATGTGTTTGCGTGAT
ACTGATACGCAGAGAGAAGAAGTTGTTTCACAGTTTTCAAAGTATGTAGTTAATGCTGTGAAGAGAAAACAGT
GTCGTAAGAAACGGCCTCCTACAAGAGAAGGGGCTAAGTTGCATGTAAGAGCATTGATTTCAAGAGGAAAATG
GGAGGAGGTTGAACATTGGTCAGGGTCTTCTGAGTCAGATGAAGAAATTGACATTGAAGAGTCAGAGGAATCT
GTGAGACCAATAGTGACAACTAGACAGAGACGTGAAGTGAGGGAGAGATTGGGTGGAATGACTGGAACTAATG
GTGATACAGACATGAGACCACCACAGGGACTGTCTGCAGAACAGAATATGATAGAGGAGACTCGTTCATATAC
TGCAGGGGAGCTGACTGAGCTAGGACAGAAATATAGACAGATACCTGGAGAAGGAATTTTGACTTGGTTATTG
CGAATTTGGGATCAGGGTGGAGACGGTGTGATGCTAATACTGAAGCAATTGGACTGGGAAGTGTATCCTTTGA
CCCCAGAATCTGTCTTCAAATGCACAGGGCACGCAATACTGATCGCCCCTTTAGTTTGTTGCATTTGATTAGA
GACTCTGTGGCCCAAGTATATGAGACTCCAACAGAACTGATGGATGTATCTTTTTGGCATACATTGGGTGAGG
GGATTCAGAGAGTACGAGAGGCTGGGTGTATTACAGGATTAGTTAGGGATCCCCTTCCTATAGATGGACGTTA
TGGGATATCTACCACCTGGGTGGGGCCAGACATGTCCTGATTTACTCCTTTAATGAGACAGAAAATCCTTAAG
ACAGCGCCACAACATTTACGCCCTGCATTATTCACAGTATTAAGTGCTTTGGATGGGAGGCAAGGGAATGTTC
ATGAAGCAGCTACATTAATGGGTCAGTTGAAGGAGATTGAAAAAGGACCAGGACCAGGACATAAGCAAAGACC
AATACAGAAATTAGAAAAGGGAGATTTCATAAAGAGGTTTAAAATGACCAGAAAACAGTTGTTTCTTGATCTG
CTTAACAGTGGAGTTCCCAAAGCAGACATTGATGGGATTCCTACTATTGATCTGTTCAAGCGCTGGAAACAAC
TGAAGGAGAAAGGGAAGTTACAGCCAGGAAATAACCCCCCCTCCTACACCTCCTCAGAAAAGAGATAACCCAT
ACAGGGAGGCAAGAGCAGCCCTAGATGGTTATGTTACCACACAGACATCCTCCAAGGGAGAAAAAGGGTGGGG
GGTGCCACAGCCCATTTATGATTGACTAGAGGATAAATCCCCTGCTCCACTGATTAGAATTTGTACAATGAAA
CCAAGGGATAGGCCCATATGTATCAGTAGCAATTTATTGGCAGGGGCAAGTACAGCCACAAAGAGTTACAGCA
CTTTTGGACACTGAAGCTTCTTTGTTTCACAGCAATCGTAGACCCAAAGGGACAGACACTACATGGGTTGCAG
TAGAAGGTTTGGGAGGCGAGACTACATGGGCTGCTAAGGTTCACTGCAGACTACAGATAGGCAATGGCCCTGT
CTTTAATGCAAACATACTTGTATCTGAATTGCCAGACAACATATTGGGAATGGATATCTTACGGGGCCAGTCT
ATCCATACTATGACAGGAACATATGTGTTTGGCACTTCAGTTCGTTCTTTTGCTGTGTCTGCAGTCAAACCAA
TTGTGAGAAGTCATGCTAAATGGACGCCAGTGTATATTCCACCCCCGGATAACCCAGTACATTTAAAGCAATA
TCGTGTTCCAGGTGGACATAAGGAAATTACAGAGACTATCCAGGCCCTAATGGAAGCAGGTGTGTTCAGACCA
GCTGTTAGCCCTTTTAATGCGCCTGTCTTTCCAGTAAGGAAACCTGATGGATCTTACAGAATGACAATTGACT
ATCGAGGGTTAAATAAAGTGGCCCCTCCATTGGCCGCTGCTGTTCCTGATATGGTTACACTTGTAGAAACTAT
TGCTCAGACAGAAGGAGATTGGCATGCAGTACTTGATTTAGCAAATGCTTTCTTTTCAATACCAATTGATGAA
AAATCCCAGGATCAATTTGCATTTACCTGGGATGGAAGACAATAGGCTCCAACTGTGCTGCCACAAGGATATC
TGCATAGCCCCACAATCTGTCATGGACTGATTGCTAGAGATTTAGAAAAGATTCCACCAATTTCCTGCCAGAT
GTATCATTATATTGACGATAATATGATATCTGGAACAACTCAAGATGAAGTCCAAAAGGATTTACAAATTGTG
ATGGATTATATGAAAGAAAGAGTCTGGGCTATTAATCTATCTAAAGTACAAGGCCCAGCTCAGTCTGTGAAAA
TTCTGGGGATGATTTGGACTGGCTCTCAAAAGACAATTCCACAGCCTGTGTTAGACACAAATGCCAATCTGAA
AACCCCTACAACCATCAAAGAAGCTCAAAGTGTACTATGAGTGATGGGATTTTGGAGAGCTTTTGTACCACAC
CTGGGCCTAATTCTGAAGCCTATCTACAATGTTACCAGAAAAAAGTCTGTTTTCAGTTGGGGTCCAGAGCAAC
AGTGTGCCTTGGAGTTGGCTAAAGAGACTATACGGAGTCATAATTCCCTGGGGCCACTACAGCCAGATCAACC
TTTCTTTTTGGATGTTTTCACAACTGAACATGGAATGTCTTGGGGATTATGGCAAAAGGCTCCCTTGCCAGGG
GCCAGGAAAGTACCAATTGGATTCTGGTCAAAGCAGTATTTAGCTGCTCAAAGGAAGTACAGTCCACTGGAAA
AACAACTTTTAGCTGCGTACACTGCATTACAGCACGTGGAGTCTACAACTAAACAACAACCTGTGGTAGTACA
AACTGATTTACCTATTTCAGGTTGGGTCAGACAGGAAGGACTGGGGGCAAGGACTGCAGTAGCACAAAAGCCC

ACTCTTCAGAAATGGAAATGGTATTTAAGTGAAAGAGGTGGCATCTCTGCTCAGGAACCAGCTGCTTTGAATC
AACAATAAGCAGGACTTGTTACTTTTGAGTCTGTGGAACTAGATGCACCTGCAATCATGATTGATGGCAGTCC
ATTTCAGGAGGCATCAGCCTGGGAGCATTTGACAGTTGATGAATGTAAATGCGCATGGTACACTGATGGCTCA
GCTATGGTCACACTGAAAGGCCGAATTTGGACTGCTGCCGCTTTTCAACCCTCCACAGAGACAATGCTATATC
AGACTGGGGAGGGTGGGTCTAGCCAATATGCTGAATTACAAGCAGTGACTATGGTGATTGAAGAAACTACAGG
TCCTTTAATTATTTATACTGACAGTTGGACTGTTTTTAAGGGACTAACCACTTGGATTAAAACATGGCAAAAA
GATAACTGGACTGTGAATGGAAAACAAATATGGGGAGGCCCAGAACTTTGGAACAATATCTGGCATGCTGGTA
AAACTCGAATGATTAAAGCGGGTCAGGTTAATGCCCACTCTGGTAATAAAAACAATGAGTTTGTAGACCAACT
TGCCAGTATTTCCTCAATTGAACTTACTAGACTGGCAGAGGGACATGGACAATCAGGACATAGGGGTGCTAAA
GGCACGTGGAAATGGGCTAACACGAAAGGAATTCCCATCACACATACTCAAGCTCAAGATATTGCTGCCAAAT
GCCCTATATGCCAGGAAGTAAATCATCGTAAAACTTTGCCTGGTGAACCTGGACATATTTATTGTGGACAAGC
ATCTGGCCAAATTTGGCAAGTAGACTATATCGGACCTCTTCCTGTTGGGAGAAATGGATTCAAATACTGTGCA
ACAGCACTTGACACTTTTAGTGGAATACTAATTGTTTGTCCTACCAAATCAGCAGATCAAAATACCACTTTGA
AATTACTGAACTTGCTGCAACAGTATTATGGTCTGCCTAAAGAAGTCCAGTCTGACAATGGATCCCATTTCAC
TGGTTGTAAGATCAAAGAATGGGCAAAAAATAATGGAGTAAATTGGGTGTATCATATACCCCATTATCCACAA
GCATCTAGACTTATTGAAAGATATAATGGTTTACTCAAAGAACAAATAAAAAAGCTGGCCCCTACACATACAT
TAACCTCCTTGCAGTTAAGCCCGACCTTCACTCGGGCAAAAAAAAACTGCAAGGATGGTTAAGCCCGAGATTT
TGTACATGCTTACTTACCTGGTCCCCCTGTGCTCATCCAGCGTCGTCCTCCATCGATCATCGTCCGCCGATCT
CTCCNNNNNNNNNNNNNNNNNNNNNNNNNNNNNNNNNNNNNNNNNNNNNNNNNNNNNNNNNNNNNNNNNN
NNNNNNNNNNNNNNNNNNNNNNNNNNNNNNNNNNNNNNNNNNNNNNNNNNNNNNNNNNNNNNNNNNNNNN
NNNNNNNNNNNNNNNNNNNNNNNNNNNNNNNNNNNNNNNNNNNNNNNNNNNNNNNNNNNNNNNNNNNNNN
NNNNNNNNNNNNNNNNNNNNNNNNNNNNNNNNNNNNNNNNNNNNNNNNNNNNNNNNNNNNNNNNNNNNNN
NNNNNNNNNNNNNNNNNNNNNNNNNNNNNNNNNNNNNNNNNNNNNNNNNNNNNNNNNNNNNNNNNNNNNN
NNNNNNNNNNNNNNNNNNNNNNNNNNNNNNNNNNNNNNNNNNNNNNNNNNNNNNNNNNNNNNNNNNNNNN
NNNNNNNNNNNNNNNNNNNNNNNNNNNNNNNNNNNNNNNNNNNNNNNNNNNNNNNNNNNNNNNNNNNNNN
NNNNNNNNNNNNNNNNNNNNNNNNNNNNNNNNNNNNNNNNNNNNNNNNNNNNNNNNNNNNNNNNNNNNNN
NNNNNNNNNNNNNNNNNNNNNNNNNNNNNNNNNNNNNNNNNNNNNNNNNNNNNNNNNNNNNNNNNNNNNN
NNNNNNNNNNNNNNNNNNNNNNNNNNNNNNNNNNNNNNNNNNNNNNNNNNNNNNNNNNNNNNNNNNNNNN
NNNNNNNNNNNNNNNNNNNNNNNNNNNNNNNNNNNNNNNNNNNNNNNNNNNNNNNNNNNNNNNNNNNNNN
NNNNNNNNNNNNNNNNNNNNNNNNNNNNNNNNNNNNNNNNNNNNNNNNNNNNNNNNNNNNNNNNNNNNNN
NNNNNNNNNNNNNNNNNNNNNNNNNNNNNNNNNNNNNNNNNNNNNNNNNNNNNNNNNNNNNNNNNNNNNN
NNNNNNNNNNNNNNNNNNNNNNNNNNNNNNNNNNNNNNNNNNTTACAGGTCTACAATTTAAAAAAAAATT
TCATGAAAAACAGTGGATCACTTTTGGTACAGAAATCTAGACCTCAGTGTAACGCTTAGGTGGTTAAGAGGTT
GGGACCGGGTTGTGTTACAAGCTGTGTTTTTGTTAAATCAACGGGTGGTAGGGGGTAGTACACCATTCCAGCG
TATGCTAGGTCGGGGACAAGATAAAGTAATTCCTGAATTAGTTATCACTGTTAAAGAGAGAATTCAGGTACAG
GATAACAAAATAGAAATGAATGCCTATGAAGAAATTGAATTTGGGGATCCATTGCAAGAGGGAAATTGTGAGT
TGTTACAATTAGAATATAGTGTCCCAACAGGCATCCTCAGGATAGATGAAATGACTACATTACATTTCACTCC
ATTTTCTGACATTACTAATTGGTGTGACTGGGACAATGACTTGAGCCAAGACATTTTTCAAGAAAATTGTGTG
ACAATACGCCCATGGAAATTTGTAAAATTATGTAAGGGACAGTTAATTGGATATGTTACCCTAATACCAAAAA
GGGTGAAAGTGACAGTACAAACCTATGTAAATCAAACAGAGTAAAAAGTATGGATAATACCCAGTGCAGCGGA
TAGGAGGAGGGAAAGTAAAAAGCAAGGTGAAGTAATTGCTAGAGGACAGGGTAATACTTACCTAATACTCAGA
GATGGAGATGTTAACCCTGTTTATTTTCCTTCCCACAGACTGGTTCCAATTTTGAAGTAGAAGAATTTGGTGG
CGCTTGCTGGTATCATTATGGGCTGAAGTGGATTCTGATTATCAACGTCTTATACAAGACACTTTTGAAAATA
AACTATTATATATGCATCACCGTTTTGCCCAGCTGATGAATAAGACGGACTGCTGGATATGTACTCATAGTCC
ATTGTCTTCTAGAACTATGCCATTTATTGCCATTCCAGTACCAAAGGAGGAACTTTTAAACAGGACGCGGATT
GATTATGGGGAATTGACATTGGGGAAGTTTAATCACACAATTATGGGGCTGCCAATAATGGCTTGGTTATCAG
AACCTTGGTGGACTGCATTTAGAAATTATACATTTACAGGCCGTGCAGTAGTTTGCTCCAAAAAACGGCCAAA
GGTTTGCACAACAACTATTAGTTCACCAATTAATCTAGGACAAACACCGCTGGGAACCACCATTAATCTATAT
TATACAAAAACAGGAATCAAATATACTTTGATGGAAGATGGAAAAGAATTCAAAGAATTATATAAAGACACTA
TATATCGTGAACCTTTGTTTTCGTTTGCATATCAGATAAGCTATCTTGGACAATTTCAGTTGCCACATGATTG
GTATTATGCATGTGGGAATAATGCTTATAGGTTCCTACCTGTTGGGGGCACCGGTAGGTGTACCATTATAAGA
AAAGCTCCGTTTACTTGGTTTATTGATACAAATGATTTAGAGATTCAGAAAATGCTAAAACACCAATTATATA
AAAGAGAAACCCCCTAGAGTTAAAGCAAAACCCCTCATACACCTACCTTGGGAGAAAAAGTTTGGTTTATCCCT
CACTATTGTTGGATTGTCAGTTCAGAATGCACATACAATAGAGAAATTTGCTGAATTGTTTGACAATGTTACA

GATGAGATGTTTGACACATTAAACATTATCAATGATGCATTGAAGTGGCATACCAAAGTAATAGCTCAGTATG
GGCTTGTGCTAGACTATCTGACTGCCTCACAAGGAGGAATGTGTACAATCGTGGGTACGACTTGTTGTAATTA
CGTGTCACAAAATTATAGTGAGTTTATTGAACGGCACATTGAACATATGAAACAAATAAAAGATAAATTTAGA
GAAGAATATTCAAAAAGTGACTGGAATGTAAATGTGCCAATTTGGCTGTCCTGGTTGAATCATTCATATTGGT
TCTCAGGGGTTGGGGGTTGGATTGGAGGTATATTATATGTTTTATGGTATATAGTAATTGTTTTAGTAATTAT
ATATATATGTATTCGTCTGTTTTGCTGTTCTATATCGAAGTGTACTGCTTCAACAGTGGAAGTTCTGCCTTTG
CGTACAGTTGGTGTTTCCTTATGATGTTGGTGAGCCAGGGAGTGGAGTGTTGGGTTAGCACAAATTATTTAAT
ATCAATTATGTCTCACCAACAATAATGCTTTACCAGTATATACTGGTATATGTATACAATATATAAGATCATC
CTAAAGGAATGCAGCATATCCCGTAAAGTTTAAGAATGATTATGGGTCAGTACTGGTCAAACACCAGCCATTG
TCTTAGTTCCTTCCTGAGTAGTTAAAAACACAGTGCCATACCTAATGAAGGAGTCTTGACCAGAGCTTGCTGG
ATGCAACAGGTTTCTTTGATCATCTGCCAAGTTATTGATTTCCCGCAGAATCTTTGGGTGAATTCAGTTTTCT
TCCGCAGCTAAGTATCTTTTGTTACCGCTAAATAATTGTTATTACAGTATTGCATTGTTTTGTCTATCTATCC
TATTATTAAATAGTATAATCGTTCAAGTCTTTGTGTTATCTCTCTTTGTACACTTCAAATTGAACCATCAATA
TTTGCATAAACTGTCACAAATTATATCCGTGACATAACACAGGT

>MH186040.1__(reversed)_Provirus_1 Pyxicephalus adspersus clone BAC 10K01
genomic sequence
ATGTGTTGGGTTAGCACAATTTATTTAATATCAATCATGTCTCACCAACAATAATGCTTTGTCAGTATATACT
GGTATATGTATACCAGACATGAGGTCATCCTAAAGAAATGTAGCATATCCTGTAAGGGTTAAGAATGATTATG
GGTCAGTACTGGTCAAACACCAGCCATTGTCTTAGTTCCTTCCTGAGTAGTTAAAAACACAGTGCCATACCTA
ATGAAGGAGTCTTGACCAGAGCTTGCTGGATGCAACATGTTTCTTTGATCATCTGCCAAGTTATTGATTTCCA
GCAGAATCTTTGGGTGAATTCAGTTTTCTTCCGCAGCTAAGTATCTTTTGTTACCGTTTATTAATTATTATTA
CAGTATTGCATTGTTTTGTCTATCTATCTTATTAATAAATAGTATAATCATTCAAGTCTTTGTATTATCTCTC
TTTGTACACTTCAAATTGAATCCCATCAATATTTGCATAAACTGTCACAAATTATATCCGTGACATAACACTT
GGTACCAGGAGACGTGGGGTTTGATGTGTGCAAACTAGAGATAGTGATGATAAGATTGATAGCAGGAGAATTT
TGATGGTGTTTGTGCGGATTTATTATTTTAGTTTTTGTATTGTAAGTCATAGTGCAATCAGAATAAAATGCAG
AAGTTGTTTAGCTGGGGTAAAAGAAAGTTGCAGGATGATCAGAGTGCGAGTCCACTCCCAAGTTGGGTGTCCA
CATCTGGACAGTGGAACCCAATTGCTCGCGAGTGTAATAAATATGTTGCACCGATCCGATTGGCAGACCCCTG
CCAGTCGGTGAGGGAATTAGATCTGTCTCGGTATGACAAAAAGGAGCAGAAGGCCGCTGCACAGGTGGGATGG
CTGTGTTTTCAGGCGCTCCAAGCTGAGACAGAGAAAAGAGAGATATTGGAATCTGAACTGCAGGTTGTTACAG
AGCAGTTATCTCTATTGAGAATGTGTTTGCGTGATACTGATACGCAGAGAGAAGAAGTTGTGTCACAGTTTTC
AAAGTATGTAGTTAATGCTGTGAAGAGAAAACAGTGTCGTAAGAAACGGCCTCTTACAAGAGAAGGGGCTAAG
TTGCATGTTAGAGCATTGATTTCAAGAGGAAAATGGGGGGAGGTTGAACATTGGTCAGGGTCTTCTGAGTCAG
ATGAAGAAATTGAGATTGAAAAGACAGAAGAATTTATGAGACCAATAGTGACAACTAGACAGAGACGTGAAGT
AAGGGAGAGATTGGGTGGAATGACCGGAACTAATGGTGATACAGACATGAGACCACCACAGGGACCGTCTGCA
GAACAGAATATGACAGAGGAGACTCGTTCATATACTGTAGGGGAGCTGACTGAGCTAGGACAGAAATACAGAC
AGATACCTGGAGAAGGAATTTTGACATGGTTATTGCGAATTTGGGATCAGGGTGGAGACGGTGTGATGCTGAC
TAATACTGAAGCAATTGGACTGGGAAGTGTATCCTTTGACCCCAGAGTCCGTCTTCAAATGCACAGGGCACGC
AATACTGATCGCCCCTTTAGTTTGTTGCATTTGATTAGAGACTCTGTGGCCCAAGTATATGAGACTCCAACAG
AACTGATGGATGTATCTTTTTGGCATACATTGGGCGAGGGCATTCAGAGAGTACGAGAAGCTGGGTGTATCAC
AGGATTAGTTAGGGATCCCGTTCCTATAGATGGACGTTATGGGGTATCTACCACCTGGGTGGGGCCAAACATG
TCCCGATTTACTCCTTTAATGAGACAGAAAATCCTTAAGACAGCACCACAAAATTTACGCCCTGCATTATTCA
CAGTATTAAGTGCTTTGGATGGGAGGCAAGGGAATGTGCATGAAGCAGCAGCATTATTGGGTCAGCTAGAGGA
GATTGAAAAAGGACCAGGACATAAGCAAAGACCAATACAGAAAACAGAAAAGGGAGATTTCATAAAGAGGTTT
AAAATGACAAGAAAACAGTTGTTTCTTGATTTGCTTAGCAGTGGTGTTCCCAAAGCAGACATTGATGGGATTC
CTACTACTGATCTGTTCAAGCGCTGGAAACAACTGAAGGATAGAGGGAAGTTACAGCCAGCCAGACCCAAGAA
GGTGTCAGCTAGTGATGTACAAAAAATAACCCCTCCTTCTACACCTCCCCAGAAAAGAAACAACCCGTATAGG
GAGGCACCAACAGCTTTATATGAATATGTTACAACACATACACCCTCCAAGAGAGAAAAAGGGTGGGGGGTAC
CACAGTCTATATTTCATTGACTAAAGGATAACTCCCCTGCTCCACTGATCAGAATACAGACACTACATGGGTG
ACAGTGGAGGGTTTGGGAGGTCAGATTACATGGATTTCTAAGGTTCACTGCAGACTACAAATAGGCAATGGTC
CTGTCTTTAATGCCAACATATTTGCATCTGAATTGCCAAACAACATAACAGAAATGGATATCTTACGGGGCCA
GACTATCCATACAATGACAGGGATGTATGTGTTTGGCACTTCAGTCTGTTTTTTCTCTGTGTTTGCAGTCAAA
CCCATTGTGAGGGGTCATGCCAAATGGACATCAGTGTACATTCTACCCCTGAAGCACCCAGCTTATTTGAAGC

AATATTGGGGTACATACAAAAATTACAGAGACTGTTCAGGCCATGTTGGAAGCTGGAGTGTTCAGACCATCAG
TCAGTCCTTTTATTGCTCATGTTTTTCCAGTAAATAAACTTGATGGATACTACAAGATGACCACTGACTATCA
AGAGTTTAAAAAAAATGGTCCATATATTGGCTGCGGCTGATTCGGATATGGTGACACTTGTACAGACTATTGC
TCAGACAGTGGGGGAGTGGCATATGGTTCTTGATTTATAAAAAAATTTCTTTCCAAAGACCATTGATGGAGAG
TCTCAGGACCAATTTGTGTTTACCTTGGATGGAAAACAACATGCCACAATTGGATTATTACAAGGTTACCCAC
ACAGCCCAATGATTTGTCATGGATTGGTTGCTAAAGATCTAGAAAAGCTTCCACCTATTTCTTATCTGGAACA
GCTCTAGATGAAGTACCAAACGACAACCAGTGGTAAAACAGACAGAGTTAAGTAAAAGTGGTGGTATTTCTGC
TCGGGAATCAACCATTTTGAATCAACAGTTAGCGGGACTGGTAACTGCACCTGTTGAACTGGAAGCACCTGTA
ATCATGATTGATGGGAAACTTTGCCAAGAGGCTTCGGATTGGGAGCATTGGACAGCTGATGAATATATAATTG
TGTGATACACTGATGGTTGAGCTATGGACAAATTTGGACTAATACTGCTTTTCAACCCTCCACAGGGACAATG
TTTTATCAGACTGAGAGGGAGGGTTTAGCTGATATGCTGAAATACATGCAGTGGATATGAAGATTGAAGATAC
TGCAAGTTCATCTGGACTTAATGAAAGTTACAATGGCCTGCTCAAACAAAAAAGAGCTGACCCCCACAAATAC
ATTGATAGATTGGGTTTGGGTTGTGTCACAAGCCTCATTTTTGTTAAATCAGTGGGTGGTGGGGGTAGTACAC
CATTCCTTCACATGTTAGGTTAGGGACAACAAAGAATTTTCTGAACTAGTTTTTATTGTTAAAAAGTGAACTC
AGTTACAGGACAAGATAGATGTTTTTGGGGGGTCCATTGTGGGAGAAAAACTGTGAGTTTGTATAAACTGGAC
TACCGTATTCTATCAGGCGTACATAAAAAGATGATGTGACTATGACCCATTGCCTGACTGATATGACAGTGAC
TTGACCCAAGACATTTTTCAATAATGTTATATGATTATTTGCATGGATATGGATAATATCGAGTATATTAGAT
AGAGGAAGGGAAAGTAAGAAGAAAGATGAAGTATTTGCTAGTAAATGCTAATACTAATGTAAAACTCAGAGAT
GTTGATGTTCACCCTGTTTATTTTCCTTCCCACAGACTAGTTCCAATCTTGGTGGCTCTAATTGGTTTCACTA
TGACCTGGAGTGGATTCTGATTATCAACATCTTATACAGGACACGCTTGAAAAAAAATTATTGTATATGAATC
ACCGTTATGCCCAGCTGATGAATAAGACGGACTACTAAATATGTTCTCATAGTCCATTGTCATCTAGAACTAT
GCCATGTATTATCATTCTAGTACTACCGGAAGAACTTTTAAAAAGGACATGGATTGTTTATGGGGACCTGACT
TTGGGAACATTATATTATACAAAAACAGGAATCAAATATACTCCGATGGAAGAAAAGAGTGACTGAAATCTAA
AATGGCCAAATTGCTAATCCTGGTTAAATCCTGTCATTTGATTCTCAGGGGTTGGGGGTTGGATTGGAGGTAT
ATTACATGTTTTATGGTATATAGTAATTGTTTTAGTAATTATATATATATGTATTCGTCTATTTTGCTGTTCT
ATATCAAAATGTACTGCTTCAACAGTGGAAGTTCTGCCTTTGCGTACAGTTGGTGTTTCATTATGATGTTGGT
GAGCCAGGGAGTGGAGTGTTGGGTTAGCACAATTTATTTAATATCAATCATGTCTCACCAACAATAATGCTTT
GTCAGTATATACTGGTATATGTATACCAGACATGAGGTCATCCTAAAGAAATGTAGCATATCCTGTAAGGGTT
AAGAATGATTATGGGTCAGTACTGGTCAAACACCAGCCATTGTCTTAGTTCCTTCCTGAGTAGTTAAAAACAC
AGTGCCATACCTAATGAAGGAGTCTTGACCAGAGCTTGCTGGATGCAACATGTTTCTTTGATCATCTGCCAAG
TTATTGATTTCCAGCAGAATCTTTGGGTGAATTCAGTTTTCTTCCGCAGCTAAGTATCTTTTGTTACCGTTTA
TTAATTATTATTACAGTATTGCATTGTTTTGTCTATCTATCTTATTAATAAATAGTATAATCATTCAAGTCTT
TGTATTATCTCTCTTTGTACACTTCAAATTGAATCCCATCAATATTTGCATAAACTGTCACAAATTATATCCG
TGACATAACAGTA
