# Supplementary material for: A novel class III endogenous retrovirus with a class I envelope gene in African frogs with an intact genome and developmentally regulated transcripts in Xenopus tropicalis
Source: Retrovirology. 2021 Jul 14;18:20. doi: 10.1186/s12977-021-00564-2 (PMC8278194; doi:10.1186/s12977-021-00564-2)
Supplement: Supplementary file 6 — Additional file 6: Figure S6. Alignment of RV MHR sequences including XtERV-S and mouse ERV-L. MHR is defined by three conserved residues and a fourth site occupied by a hydrophobic residue, all with conserved spacing as shown in the consensus sequence. [file 12977_2021_564_MOESM6_ESM.pdf]

```
                           ** **   ** ***** *
          XtERV-S   FAQKGGETILEWVLRVWDLG
African bullfrog ERV   YR.IP..G..T.L..I..Q.
          MuERV-L   .K..P..YVW..I.....K.
           HERV-L   YK..S..QAW..I.....N.

         Ruff ERV   .GRLP..H.VT.L..C..N.
  Turtle dove ERV   .SRRE..N..T.L..C..N.
           HERV-S   CQ.LP..PLPA.L.CL..E.

           PERV-C   VM.GPN.PPSVFLE.LLEAF
             GALV   VL.GPA.PPSVFI..LMEAY
             BAEV   IT.GKD.SPAAFME.LLEGF
             FeLV   VV.GKE..PAAFLE.LKEAY
              AKV   IT.GPN.SPSAFLE.LKEAY
             EIAV   IR.GAK.PYP.F.D.LLSQI
              FIV   LR.GAK.DYSSFID.LFAQI
            VISNA   VK..NT.SYEDFIA.LLEAI
            HIV-1   IR.GPK.PFRDY.D.FYKTL
           HTLV-1   IL.GLE.PYHAF...LNIAL
              IAP   II.GPQ.SFSDF.A.MTEAA
             MMTV   LK.GNE.SYETFIS.LEEAV
             JSRV   IR.GPD.PYSDFIS.LQE.A
             SMRV   IR.GPD.SYSDF.S.LQETA
            MgLDV   VT.GL..PF.SFAE.LLNAY
              ALV   IT.GPS.SFVDFAN.LIKAV
        Consensus   xxQxxxExxxxφxxRxxxxx
```

Figure 6
