# Supplementary material for: A novel class III endogenous retrovirus with a class I envelope gene in African frogs with an intact genome and developmentally regulated transcripts in Xenopus tropicalis
Source: Retrovirology. 2021 Jul 14;18:20. doi: 10.1186/s12977-021-00564-2 (PMC8278194; doi:10.1186/s12977-021-00564-2)
Supplement: Supplementary file 7 — Additional file 7: Figure S7. Alignment of the TM regions of the ERV and RV envelopes. Some of TMenv genes in the Fig. 6C tree have a heptad repeat stutter marked by N-linked glycosylation site (shaded in grey). The ISD region is shown in red and the CX6CC motif is highlighted in green. [file 12977_2021_564_MOESM7_ESM.pdf]

```
                                    10        20        30        40        50        60        70
                           ....|....|....|....|....|....|....|....|....|....|....|....|....|....|....|.
             XtERV-S       VAKAVQMMRRMSRIIEHALNNT-------LDGLTSLTEEVRQMRLVVLQNRASLDYILASKG--GVCALIG-DECC
             XlERV-S       -.RS....K.............-------*.R......D..K............-.......--......-....
       Alligator ERV       IGRTI.KI.LLAN.LKQVN.D.-------AES.DH..Q.MIAL.TM.....IA..FT.....--.I.....-K..Y
            Coel-ERV       T.RSI.EI.AL.T.V.QLA.K.-------SGS.LA.ST.ETAT.A.A....MA..F......--.....V.-K...
          Turtle ERV       ..Y..REI..LEVAF.KVA.A.-------TKA.RAINM.LGEV.QM.....MA...V.....--.....V.-K...
                 RSV       A.Q.LREIE.LACWSVKQA.L.-------TSL.GD.LDD.TSI.HA......AI.FL.LAH.--HG.EDVA-GM..
               ALV-J       A.Q.LKEIE.LACWSVKQA.L.-------SLI.NAIL.DTNSI.HA......AI.FL.LAQ.--HG.QDVE-GM..
African bullfrog ERV       ---S..NAHTIEKFA.LFD.V.----DEMF.T.NIINDALKWHTK.IA.YGLV...LT..Q.--.M.TIV.-TT..
            Bos-Rum1       -------TADIMVKV.ALN.F.KQALLDSKKAIEA.N..QS...KA.M...MA..ILT.AQ.--.T..I.K-V...
        Cat-SynCar1        -------IEDIIWHV.ALT.Y.TKALNDSMR..SL.NS..TL..KA.....MA..ILT.AQ.--.T..IVK-T...
              HERV-S       .ITVEMQVTAL.EHRAQN..C.-------HVI.LLF.D..DHI.KA.....M...IVTTAQ.--.-...V.-TR..
         Chimp ERV-S       FITVEMQITALAEHTVQV..Y.-------RVA.LL.ID..D.T.K.....WMA..IVT.DQ.--.T...T.-SQ..
           Platyfish       .MSSLDQIADL.KA..VIA.E.-------GKA.ILMSN.LASV..LA.....A..FL..AQ.--.T..I..-S...
     Giant mudskipper      .MS.LDQISDL.HA..TIA.E.-------GRA.QLMSS.LASV..LA.....A..FL..AQ.--.T..I..-Q...
               ZFERV       PN.N.DRINYIHYNVQRLS.L.-------R.AVSG.K.QLAATS.MAI...LA..ML.SER.--...SMFL-.T..
            Syn-Mar1       PIYMLNRII.LQAVL.IIT.Q.-------ATAFDL.ATQQT...AAIY...LA...L..EE.--...GKFNSSD..
            Syn-Cav1       SIYMLNRII.LQDAL.ITA.ES-------AYA.NP.ANQQTNIQNA.Y...LA...L..KE.--...GKFNLTS..
            Syn-Ten1       F.YHEATL.NLTQVVRNIAMS.-------GKAIE.QQRSLDSLAN...D..IA..FL..EQ.--....IAN-TS..
                 AKV       ALV.T.QFQQLQAAMHDD.KEV-------EKSI.N.EKSLTSLSE......RG..LLFLKE.--.L..ALK-E...
                FeLV       ALLETAQF.QLQMAMHTDIQAL-------EESISA.EKSLTSLSE......RG..ILFLQE.--.L..ALK-E...
                GALV       PIDLQ.GLTSLQIA.DAD.RAL-------Q.SVSK.EDSLTSLSE......RG..LLFLKE.--.L..ALK-E...
              PERV-C       PQQLETGLSNLH..VTED.QAL-------EKSVSN.E.SLTSLSE......RG..LLFLKE.--.L.VALK-E...
              HTLV-1       GSMSLASGKSLLHEVDKDISQL-------TQAIVKNHKNLLKIAQYAA...RG..LLFWEQ.--.L.KALQ-EQ..
                 BLV       S.LSH.RLTSLIHVL.QDQQRL-------ITAINQTHYNLLNVAS..A...RG..WLYIRL.FQSL.PT.N-EP..
          mouse-SynA       ITTSTVYFQQL.KALSDS.DEI-------ATSII..QDQIDSLAG......RA..L.V.ER.--.T.LFLQ-E...
          mouse-SynB       L.TSTLYFQQL.KVLSET.EEI-------AASI.T.QNQIDSLAG......RA..L.T.E..--.T.LFLQ-E...
          Syncytin-1       ITTST.FYYKL.QELNGDMERV-------A.S.VT.QDQLNSLAA......RA..LLT.ER.--.T.LFL.-E...
          Syncytin-2       IT..SLTYSQL.KE.ANNIDTM-------AKA..TMQ.QIDSLAA......RG..MLT.AQ.--.I.LALD-EK..
             Syn-ory       LGI..HKY.EL.QQLINDVQTL-------STTIQD.QDQIDSLAE......RG..LLT.ER.--.I.LALQ-EK..
         Dasy-Env1-1       LGLSLTQYTQI.NQLVSDVRAL-------SNTIKEIQDQIDSLAD......RG..LLT.ER.--.I.LALQ-EQ..
```
